# Supplementary material for: Combinatorial expression motifs in signaling pathways
Source: Cell Genom. 2024 Jan 10;4(1):100463. doi: 10.1016/j.xgen.2023.100463 (PMC10794782; doi:10.1016/j.xgen.2023.100463)

Cell Genomics, Volume *4*

Supplemental information

Combinatorial expression motifs in signaling pathways

Alejandro A. Granados, Nivedita Kanrar, and Michael B. Elowitz

Table S1. List of single-cell RNA seq datasets used in this study (related to main figures 1 and 2)

Dataset	Time points	Reference	Cells	Mice sampled	Technology	No. of Cell States in Integrated Atlas
Forelimb atlas (The changing mouse embryo transcriptome at whole tissue and single-cell resolution)	E10.5, E11.0, E11.5, E12.0, E13.0, E13.5, E14.0, E15.0	[28]	90,637	Pair of forelimbs per time point	10X	90
A single-cell molecular map of mouse gastrulation and early organogenesis	E6.5, E6.75, E7.0, E7.25, E7.5, E7.75, E8.0, E8.25, E8.5	[63]	116,312	411 mouse embryos	10X	134
The emergent landscape of the mouse gut endoderm at single-cell resolution	E5.5	[30]	-	-	10X	20
Single-cell RNA-seq analysis unveils a prevalent epithelial/mesenchymal hybrid state during mouse organogenesis	E9.5, E10.5, E11.5	[31]	1916	7 embryos	Smart-seq2	28
Molecular recording of mammalian embryogenesis	E6.5, E7.0, E7.5, E8.0, E8.5	[27]	88,779	50 embryos	10X	181
Tabula Muris, Tabula Muris Senis	1mo, 3mo, 18mo, 21mo, 24mo, 30mo	[25,26]	450,000+	-	10X, Smart-seq2	753

Supplementary Figure 1

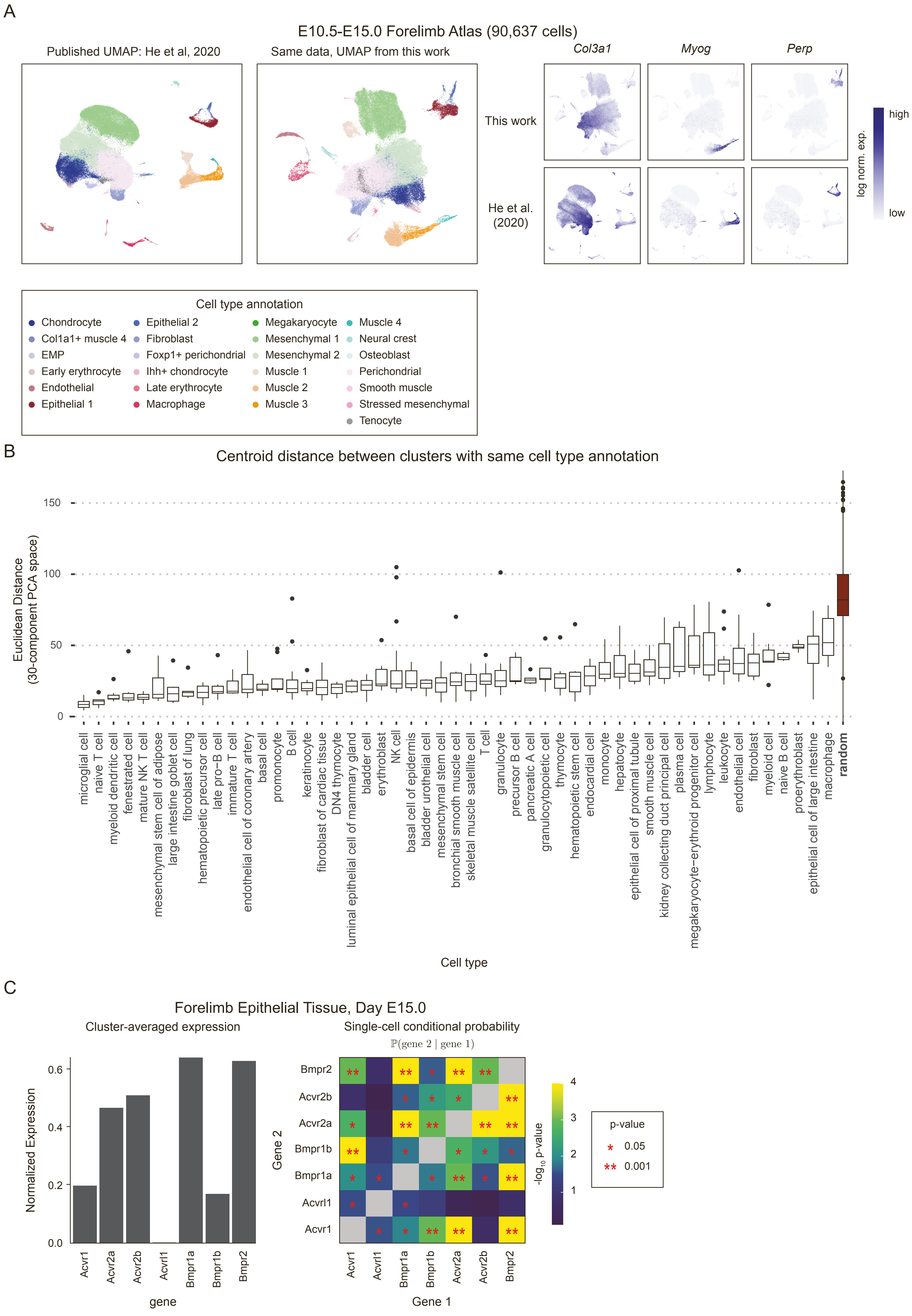

Figure S1. Single-cell RNA seq analysis and expression of TGF-β receptors (related to main figure 2)

A. Analysis of scRNA-seq datasets using the standard Scanpy pipeline recapitulates published analyses, including [28]. Independent analysis of mouse forelimb over days E10.5-E15.0 captures similarity in cell types (colors, left) and gene expression (right).
B. The integrated atlas captures cell type similarity across datasets. Cell clusters with similar annotations in different data sets remain similar to each other in the integrated atlas.
C. Cluster-averaged profiles reflect co-expression in single-cells. Shown is an example of a single cluster from the forelimb epithelial tissue data set at day E15. Left, expression of TGF-β receptor genes averaged over all cells in the cluster corresponding to forelimb epithelial tissue at day E15.0. Right, pairwise conditional probability in single cells of gene 2 expression conditioned on gene 1 expression. Pairs of genes with significant entries (**) are co-expressed in the cluster-averaged profile. Higher-order conditional probabilities were not computed due to dropout effects in scRNA-seq data.

Supplementary Figure 2

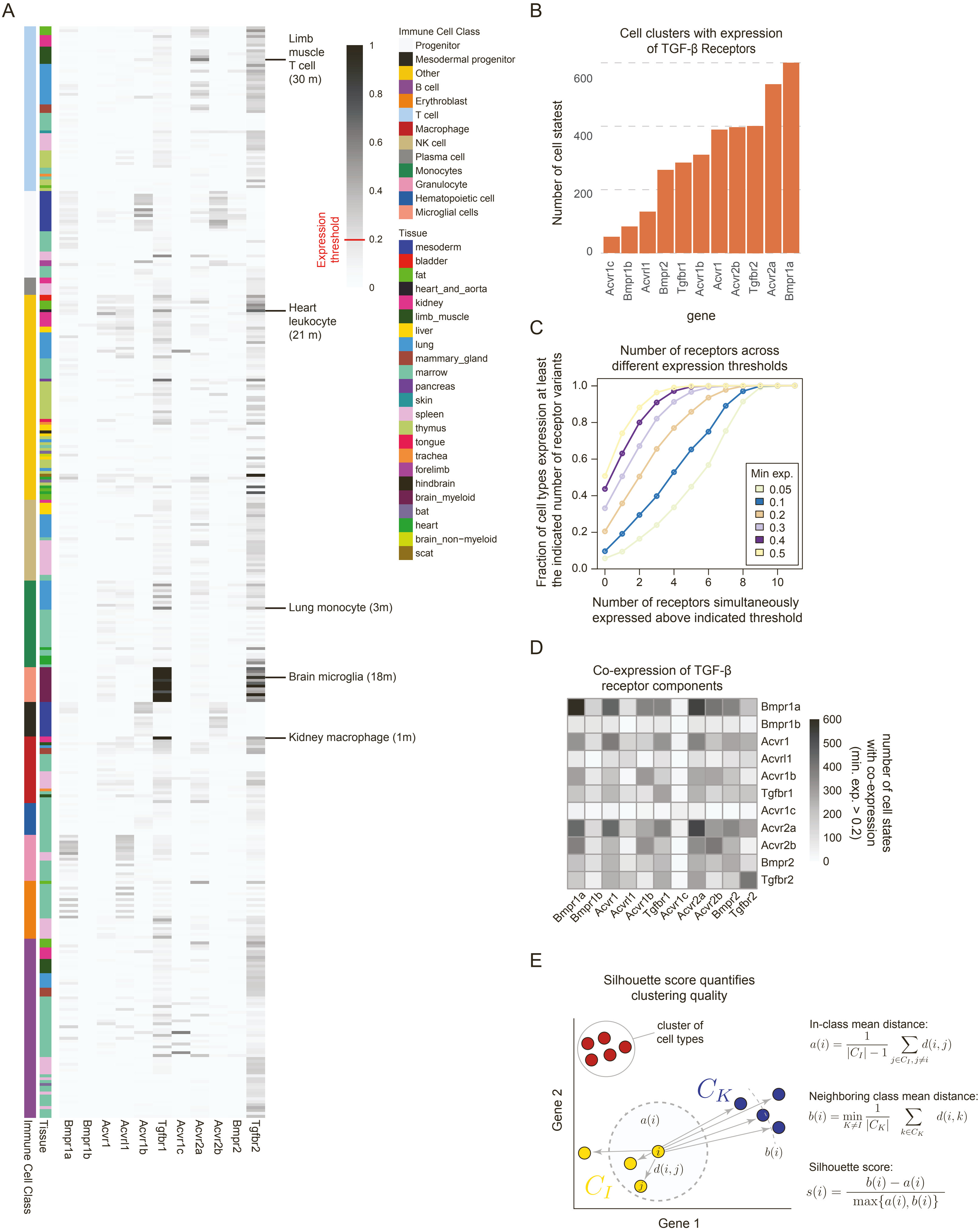

Figure S2. Co-expression statistics of TGF-β receptors across all cell states (related to main figure 3).

A. A heatmap showing the average normalized expression of TGF-β receptors for the immune cell types not included in the clustering analysis due to low mRNA expression levels. Each gene's average expression level was normalized from 0 to 1 relative to the minimum and maximum values observed across all 1206 cell types (Figure 3B, Methods). A cell type was included if it showed normalized expression above 0.2 for more than two receptors. The color side bards indicate the tissue of origin and the broad immune cell class.
B. Histogram showing the number of cell types in the integrated atlas with normalized expression of TGF-β receptors above a threshold of 0.2 in standardized expression units.
C. The number of TGF-β receptor components simultaneously expressed for different values of the minimum expression threshold (colors).
D. Pairwise co-expression of TGF-β receptor expression reveals broad receptor co-expression patterns. Off-diagonal elements indicate the number of cell states co-expressing, above threshold, the indicated pair of components. Diagonal elements indicate the number of cell states expressing the corresponding individual gene (as shown in A).
E. The silhouette score quantifies clustering quality (schematic). For a given clustering, we compute the silhouette score on every data point i. We compute $a(i)$, the mean distance between i and every other point in the same cluster, and $b(i)$, the mean distance between i and the nearest neighboring cluster. The silhouette score for the data point i is then defined as the difference between the inter- and intra-cluster distances, normalized to the maximum of the two (equations). A silhouette score value close to 1 corresponds to well-defined clusters, where the data point i is similar to other members of its cluster and dissimilar to others. In contrast, a value close to -1 suggests poor cluster assignment. Finally, the silhouette score for a given clustering, is taken as the average of the individual scores for all data points. In our analysis, a data point corresponds to a vector of the scaled, normalized counts of pathway gene expression of a single cell state.

Supplementary Figure 3

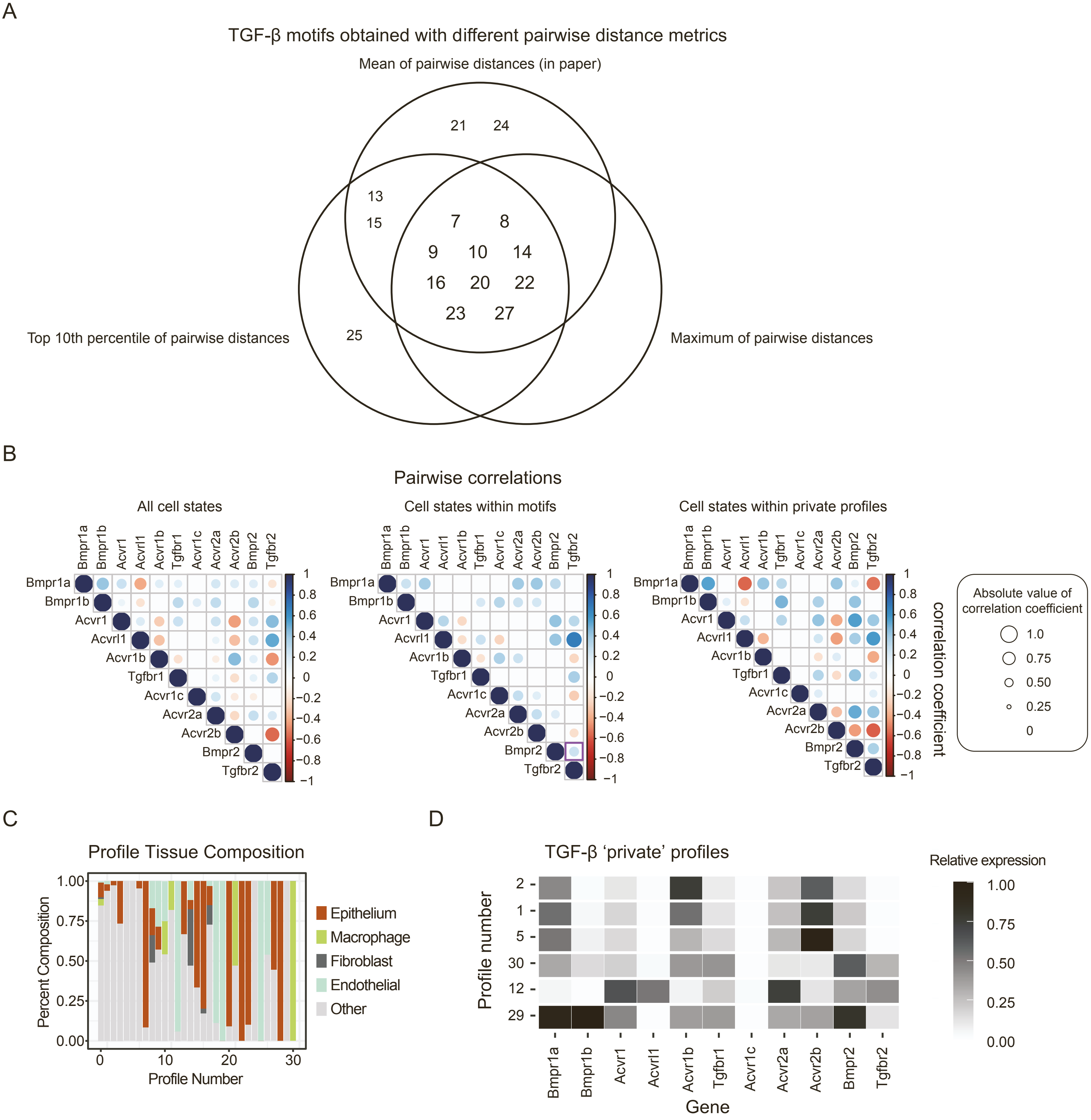

Figure S3. Dispersion metrics and private profiles for TGF-β (related to main figures 3 and 4).

A. Alternative definitions of the dispersion metric recover similar sets of motifs. The mean of intra-class pairwise distances was used as the dispersion metric throughout this work. Still, we tested two additional dispersion metrics, one that uses the maximum of intra-class pairwise distances, the second that uses the top 10th percentile. The Venn diagram shows profiles identified as motifs from these three distinct definitions of the dispersion metric. Most profiles (shown in the intersection of the three circles) are robust to the definition of dispersion. Notably, the dispersion metric that utilizes the maximum pairwise distances only captures profiles in this intersection. The mean pairwise distance, however, captures two additional profiles as motifs, profiles 21 and 24. Profile 24 contains only two cell states, liver B cells and bone marrow NK cells. The top 10th percentile of pairwise distances captures the adult endothelium-specific profile, 25, as a motif. However, the maximum metric omits profiles 13 and 15, even though they appear to be motifs since they are both dispersed across the adult smooth muscle and adult kidney epithelium.
B. TGF-β profiles exhibit unique pairwise receptor correlations. Each matrix represents the correlation coefficient for each pair of receptors across all cell states (left), cell states associated with motifs (middle), and cell states associated with private profiles (right).
C. Cell class composition of each TGF-β profile, matching classes depicted in Figure 4F. “Other” includes all cell states in the atlas that do not fall into the epithelial, macrophage, fibroblast, or endothelial cell classes.
D. TGF-β profiles with average dispersion less than the 50 percentile from the expected distribution were classified as *private profiles*.

Supplementary Figure 4

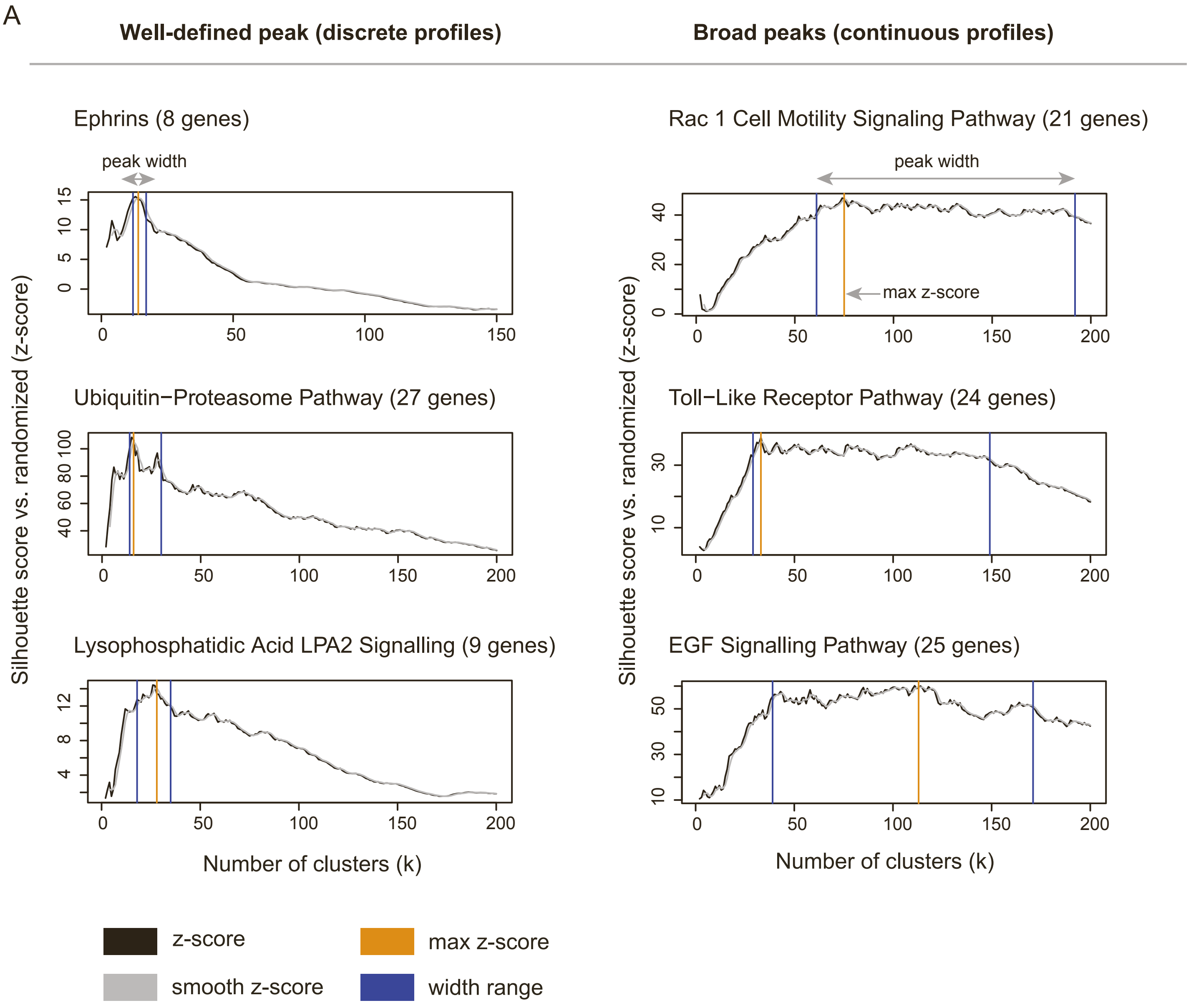

Figure S4. Silhouette analysis for other pathways (related to main figures 3 and 5).

Silhouette analysis of indicated pathways, as in Figure 3A and Figure 5A. A vertical yellow line indicates the value of k (the proposed number of clusters) with the maximum silhouette z-score. The peak width is shown with two blue vertical lines (the minimum and maximum values of *k* with a 10% difference from the maximum value). Finally, the relative peak width is calculated as the ratio of the peak width divided by the maximum number of *k* considered. We show three pathways with the lowest (left) and highest (right) relative width.

Supplementary Figure 5

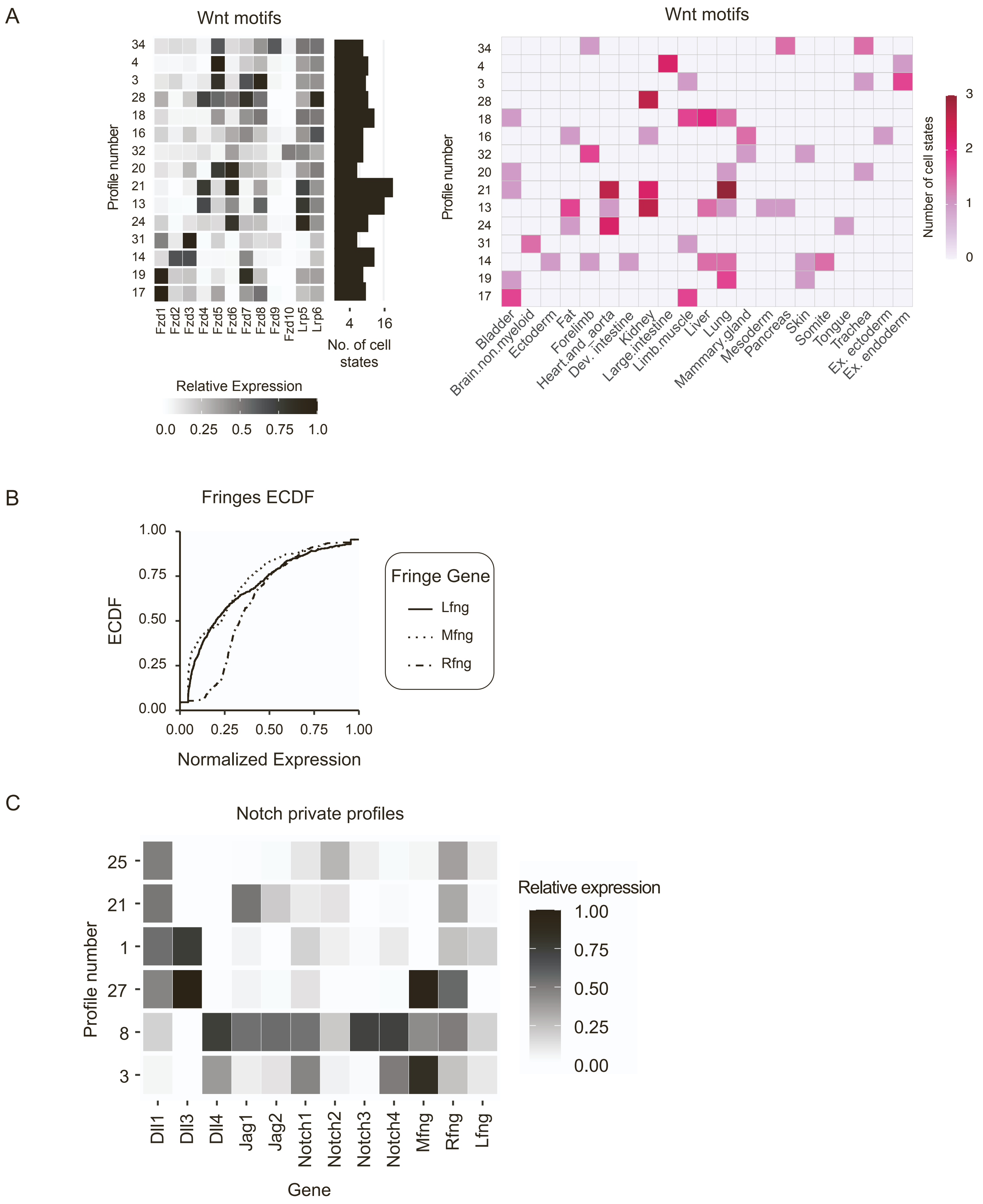

Figure S5. Motifs for Wnt and Notch pathways (related to main figures 4 and 5).

A. Motifs in the Wnt pathway and their distribution across tissues and organs, similar to Figure 4D, E.
B. Cumulative distribution histogram of Fringe gene expression across all cell states.
C. Notch pathway private profiles.

Supplementary Figure 6

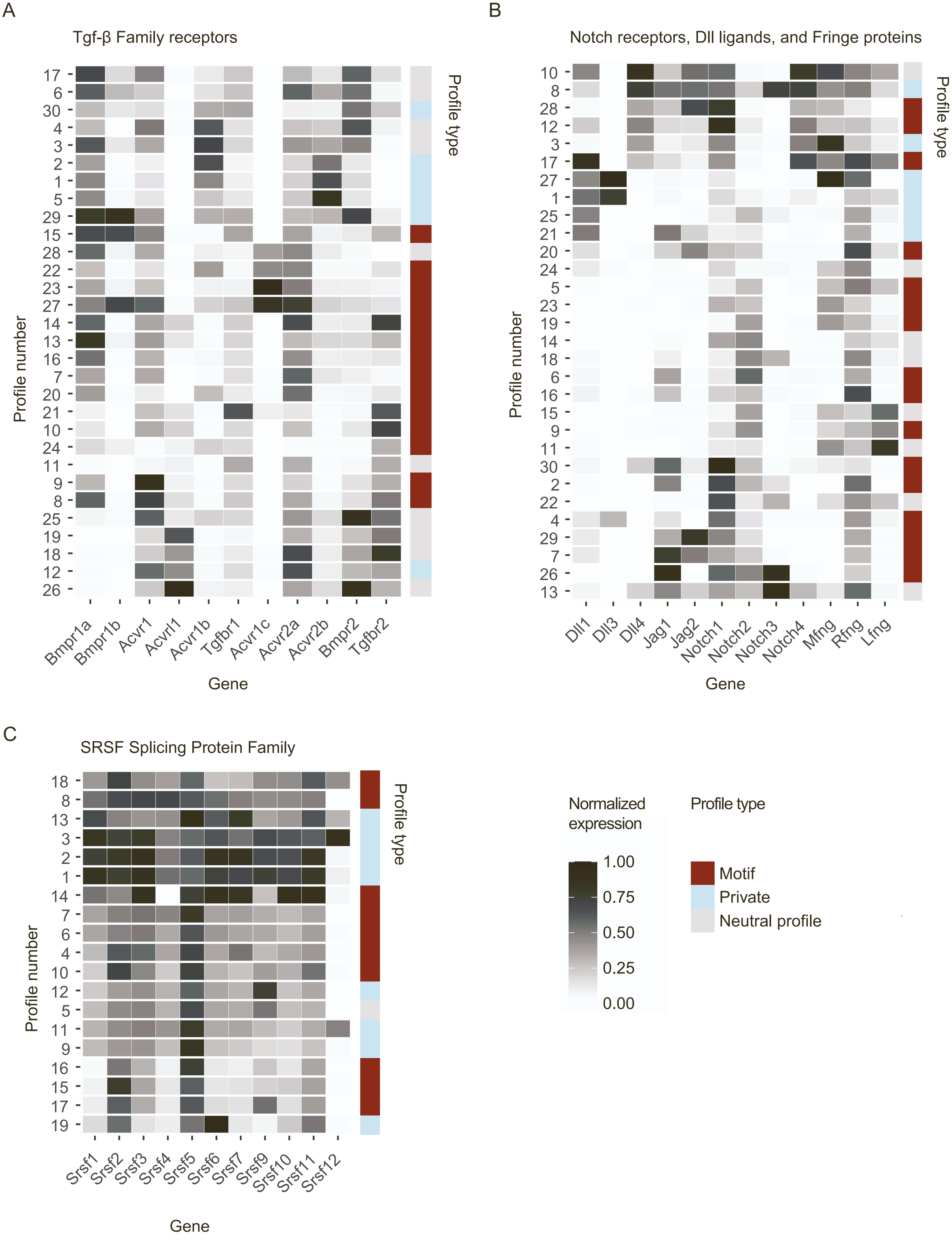

Figure S6. All profile classes for TGF-β, Notch, and SRSF (related to main figures 5 and 6).

All profiles are organized by similarity and annotated by their profile number and profile type (motif, private, or neutral) for TGF-β (A), Notch (B), and SRSF (C).

Supplemental data S1

Global cell type dendrogram, as shown in Figure 3C, along with a heatmap of the projection of each cell type on the top 20 principal components computed from the whole-transcriptome space.

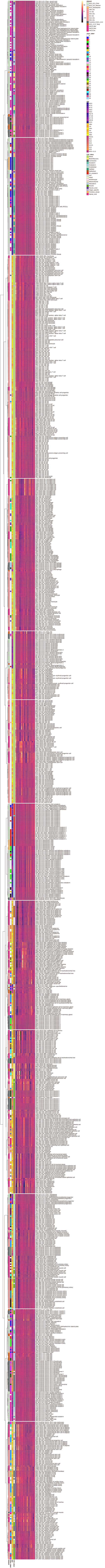

Supplemental data S2

Analysis of pathways shown in Figure 5C. Each pathway has plots displayed on two pages. The first page has plots displayed for pathways as done for TGF-beta in Figures 3 and 4. The second page shows UMAPs highlighting each pathway motif (colored dots) and displays the dispersion score for the motif.

BCR Signaling Pathway (k_opt = 110)

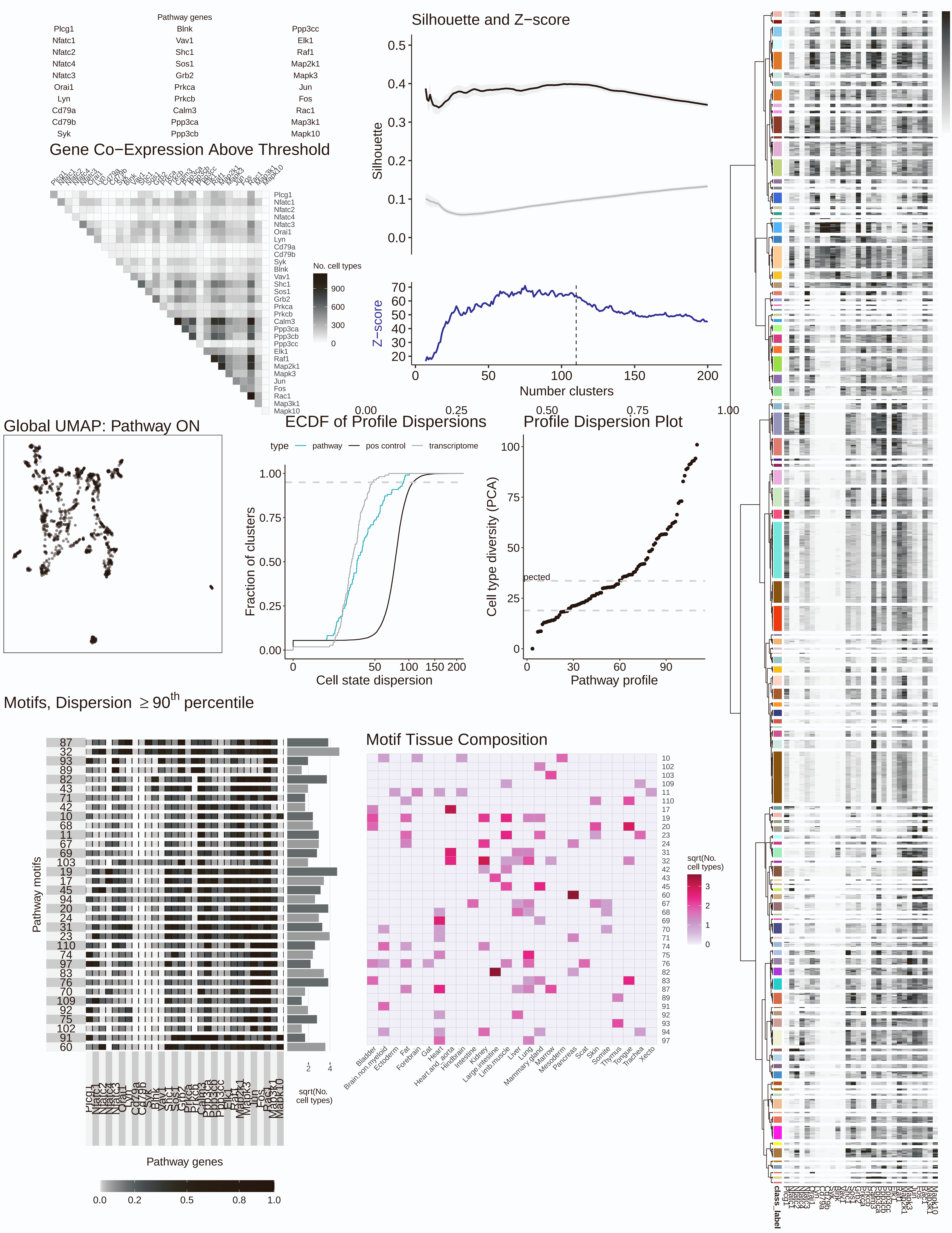

BCR Signaling Pathway (k_opt = 110)

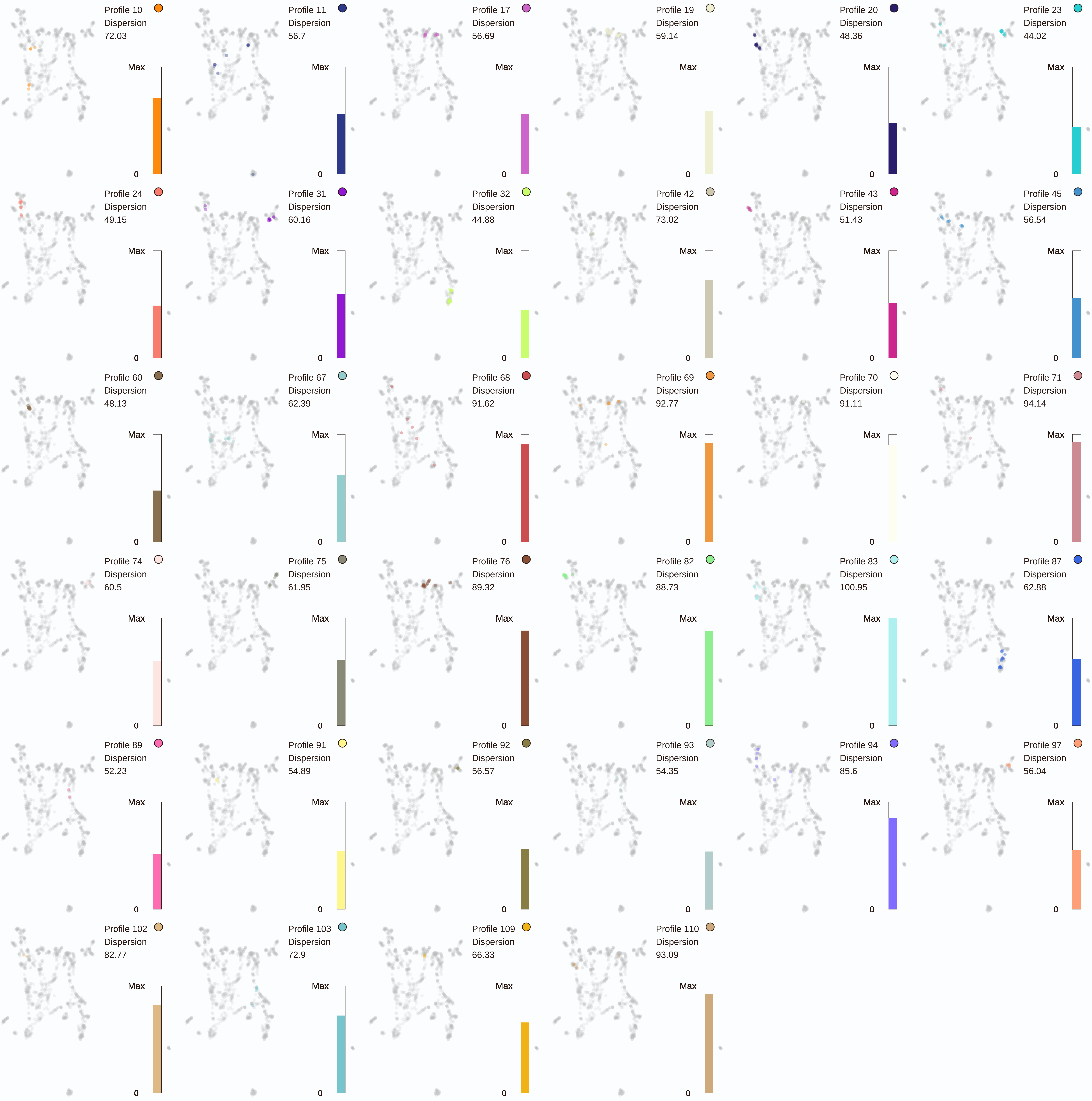

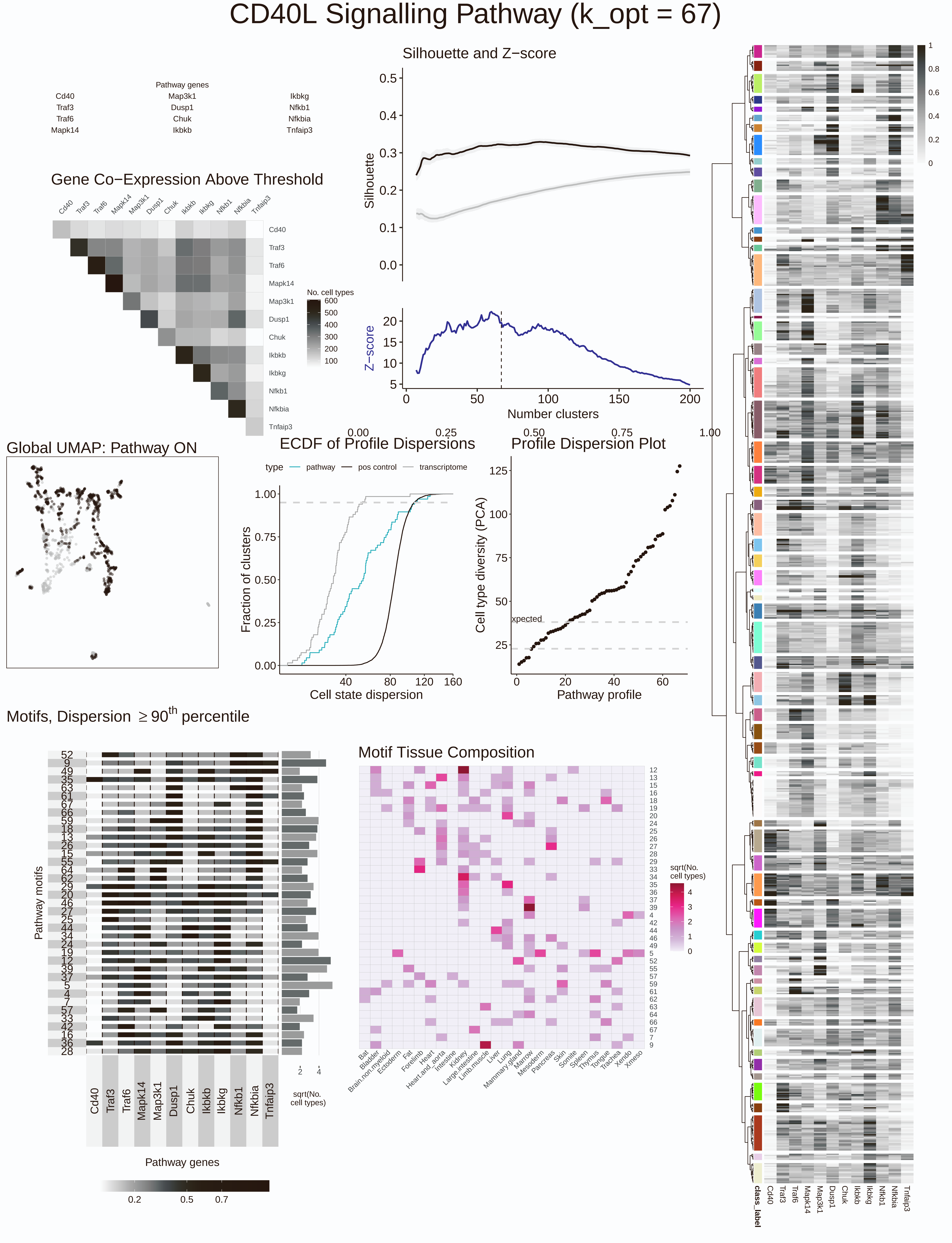
CD40L Signalling Pathway (k_opt = 67)
Pathway genes
Cd40
Traf3
Traf6
Mapk14
Map3k1
Dusp1
Chuk
Ikbkb
Ikbkg
Nfkb1
Nfkbia
Tnfaip3
Gene Co−Expression Above Threshold
No. cell types
Silhouette and Z−score
Silhouette
Z−score
Number clusters
Global UMAP: Pathway ON
ECDF of Profile Dispersions
type
pathway
pos control
transcriptome
Fraction of clusters
Cell state dispersion
Profile Dispersion Plot
Cell type diversity (PCA)
Pathway profile
xpected
Motifs, Dispersion ≥ 90th percentile
Pathway motifs
sqrt(No. cell types)
Motif Tissue Composition
Bat
Bladder
Brain.non.myeloid
Ectoderm
Fat
Forelimb
Heart
Heart.and.aorta
Intestine
Kidney
Large.intestine
Limb.muscle
Liver
Lung
Mammary.gland
Marrow
Mesoderm
Pancreas
Skin
Somite
Spleen
Thymus
Tongue
Trachea
Xendo
Xmeso
class_label

CD40L Signalling Pathway (k_opt = 67)

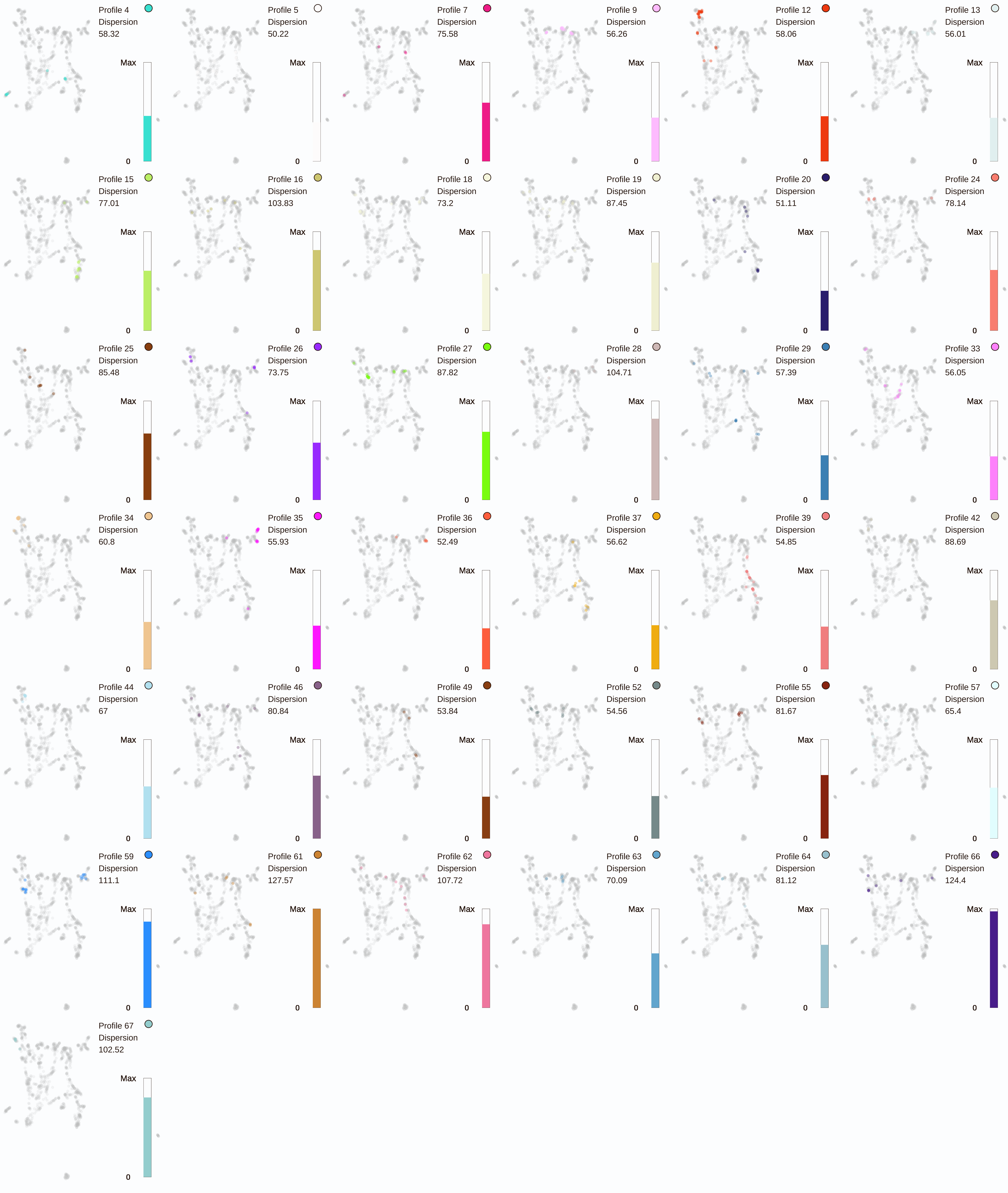

D4−GDI Signaling Pathway (k_opt = 77)

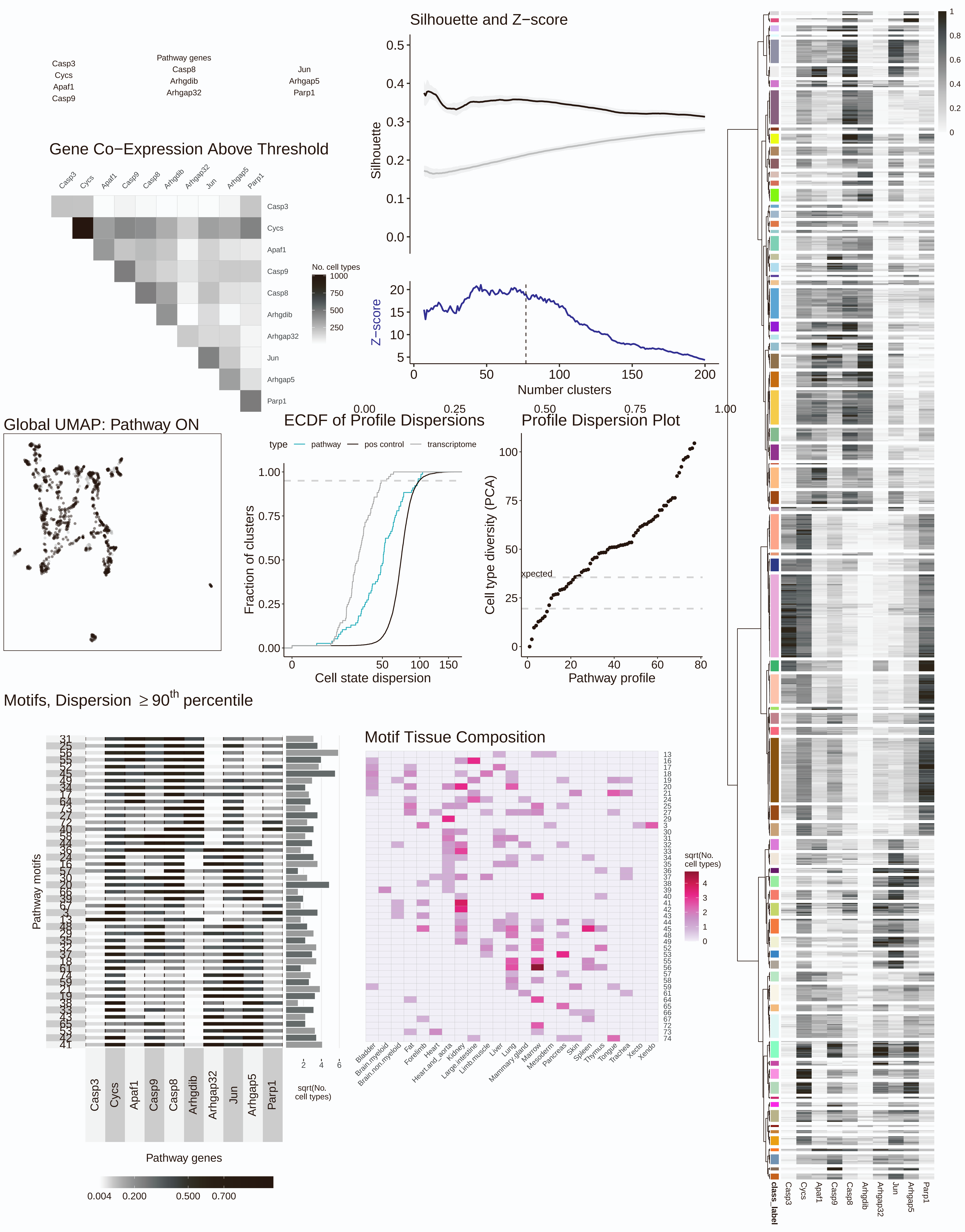

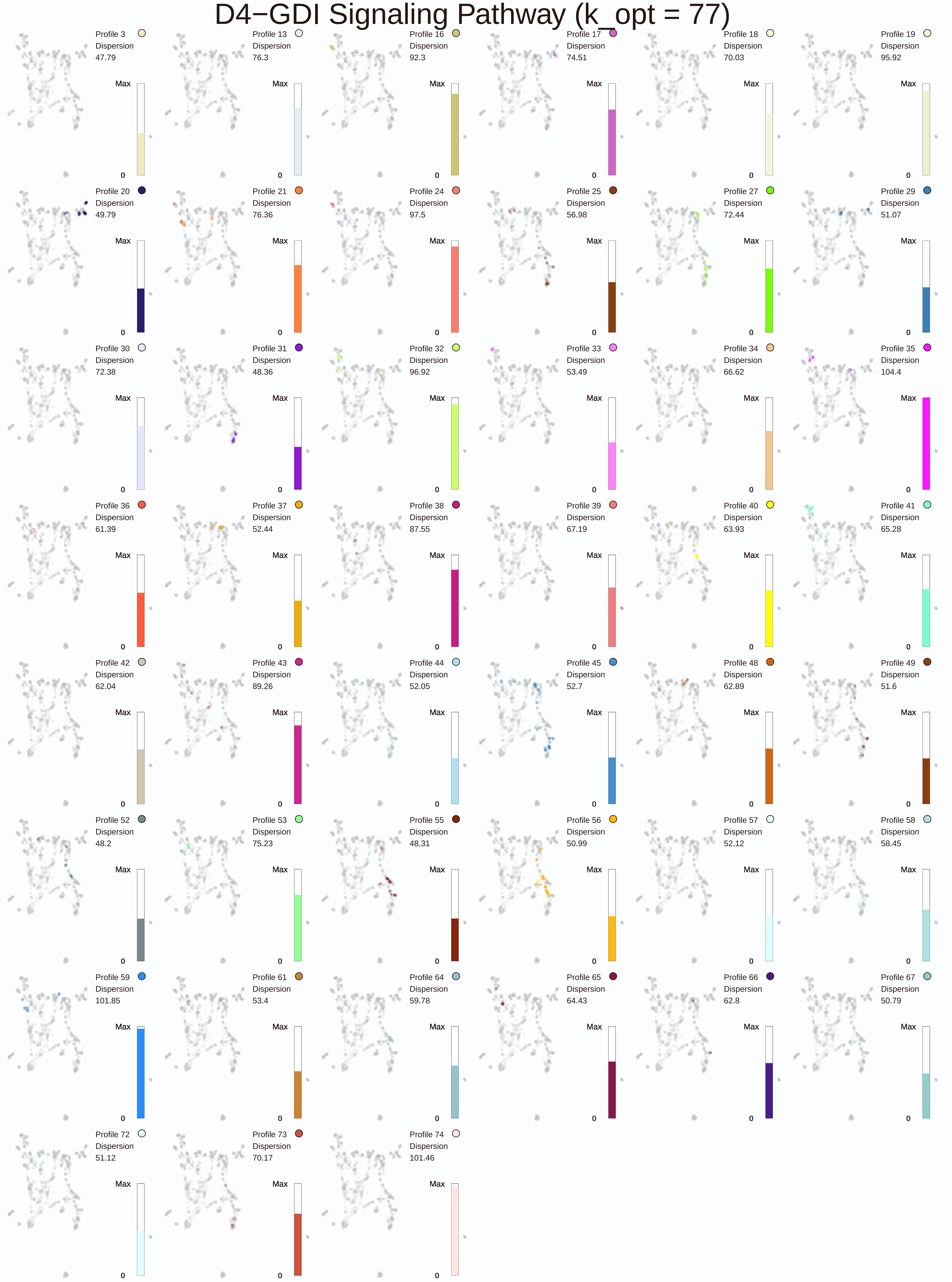
D4−GDI Signaling Pathway (k_opt = 77)
Profile 3
Dispersion
47.79
Profile 13
Dispersion
76.3
Profile 16
Dispersion
92.3
Profile 17
Dispersion
74.51
Profile 18
Dispersion
70.03
Profile 19
Dispersion
95.92
Profile 20
Dispersion
49.79
Profile 21
Dispersion
76.36
Profile 24
Dispersion
97.5
Profile 25
Dispersion
56.98
Profile 27
Dispersion
72.44
Profile 29
Dispersion
51.07
Profile 30
Dispersion
72.38
Profile 31
Dispersion
48.36
Profile 32
Dispersion
96.92
Profile 33
Dispersion
53.49
Profile 34
Dispersion
66.62
Profile 35
Dispersion
104.4
Profile 36
Dispersion
61.39
Profile 37
Dispersion
52.44
Profile 38
Dispersion
87.55
Profile 39
Dispersion
67.19
Profile 40
Dispersion
63.93
Profile 41
Dispersion
65.28
Profile 42
Dispersion
62.04
Profile 43
Dispersion
89.26
Profile 44
Dispersion
52.05
Profile 45
Dispersion
52.7
Profile 48
Dispersion
62.89
Profile 49
Dispersion
51.6
Profile 52
Dispersion
48.2
Profile 53
Dispersion
75.23
Profile 55
Dispersion
48.31
Profile 56
Dispersion
50.99
Profile 57
Dispersion
52.12
Profile 58
Dispersion
58.45
Profile 59
Dispersion
101.85
Profile 61
Dispersion
53.4
Profile 64
Dispersion
59.78
Profile 65
Dispersion
64.43
Profile 66
Dispersion
62.8
Profile 67
Dispersion
50.79
Profile 72
Dispersion
51.12
Profile 73
Dispersion
70.17
Profile 74
Dispersion
101.46
Max
0

Eph A−B receptors (k_opt = 31)

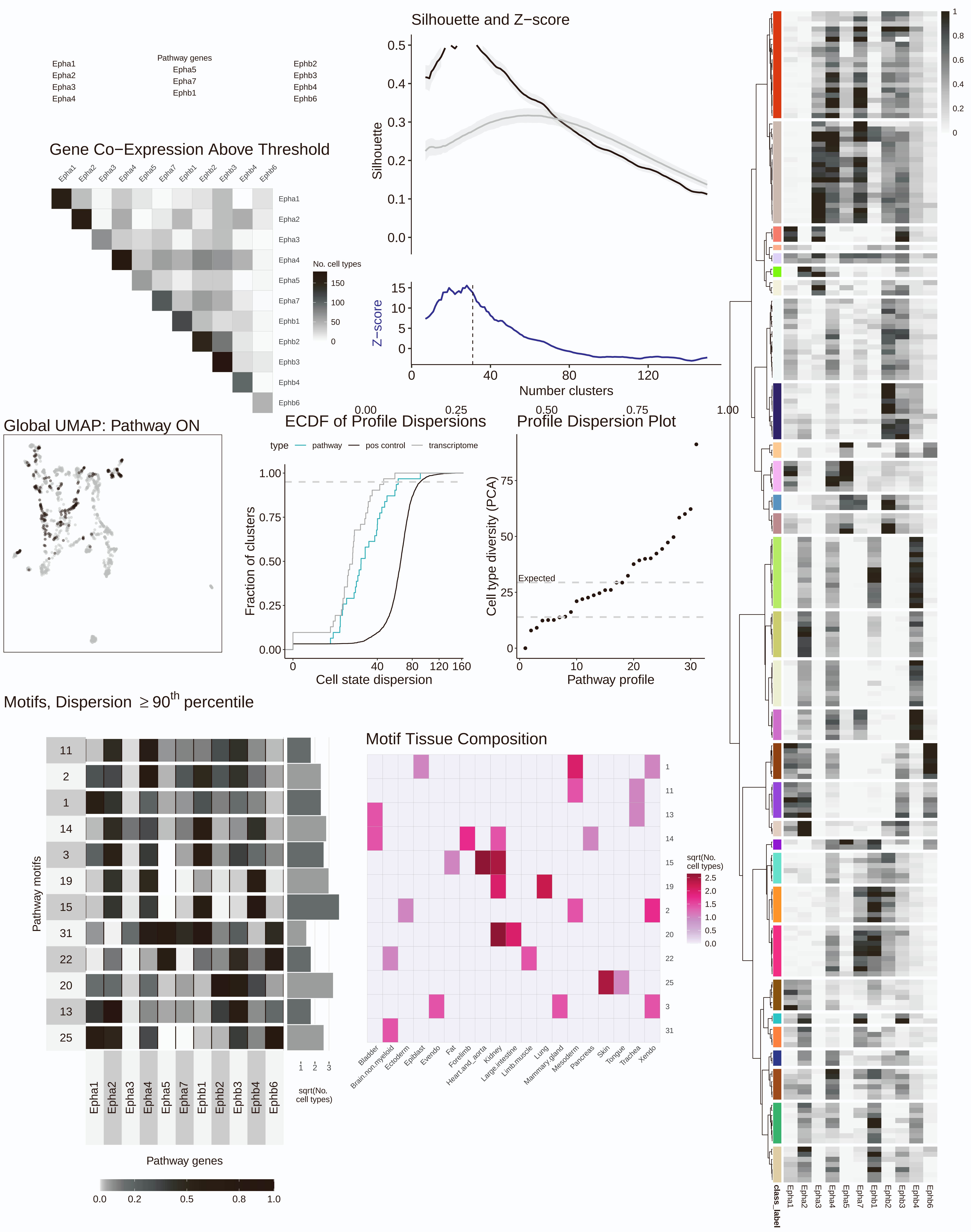

Eph A−B receptors (k_opt = 31)

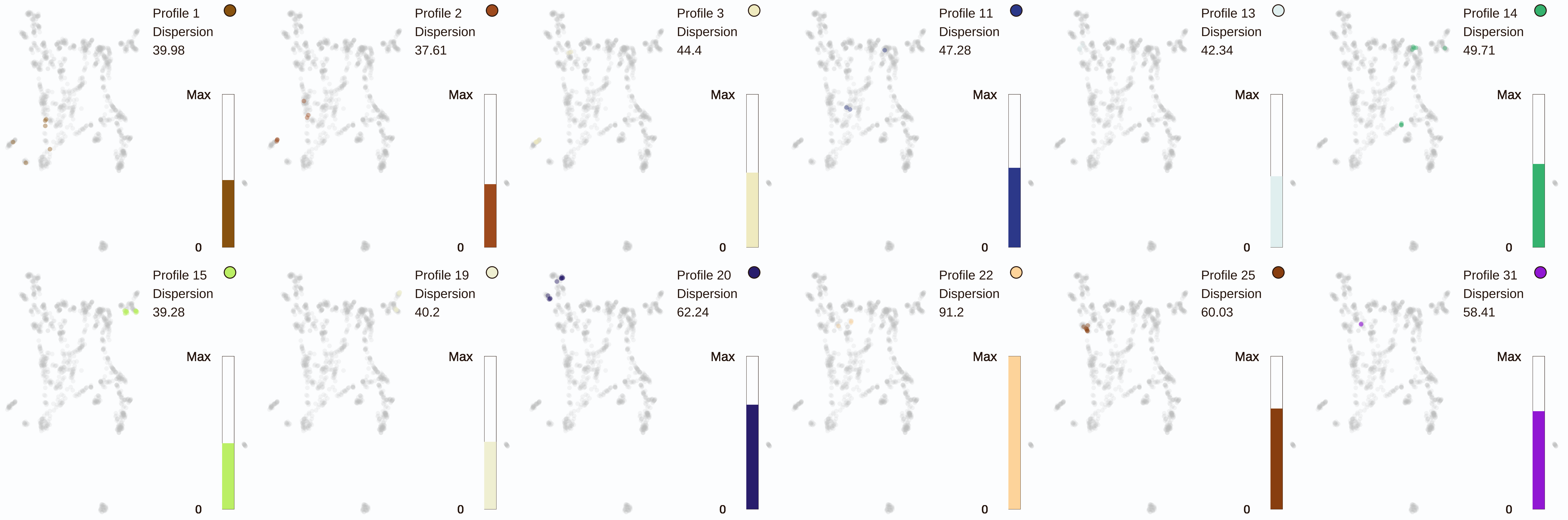

Eph receptors and ligands (k_opt = 52)

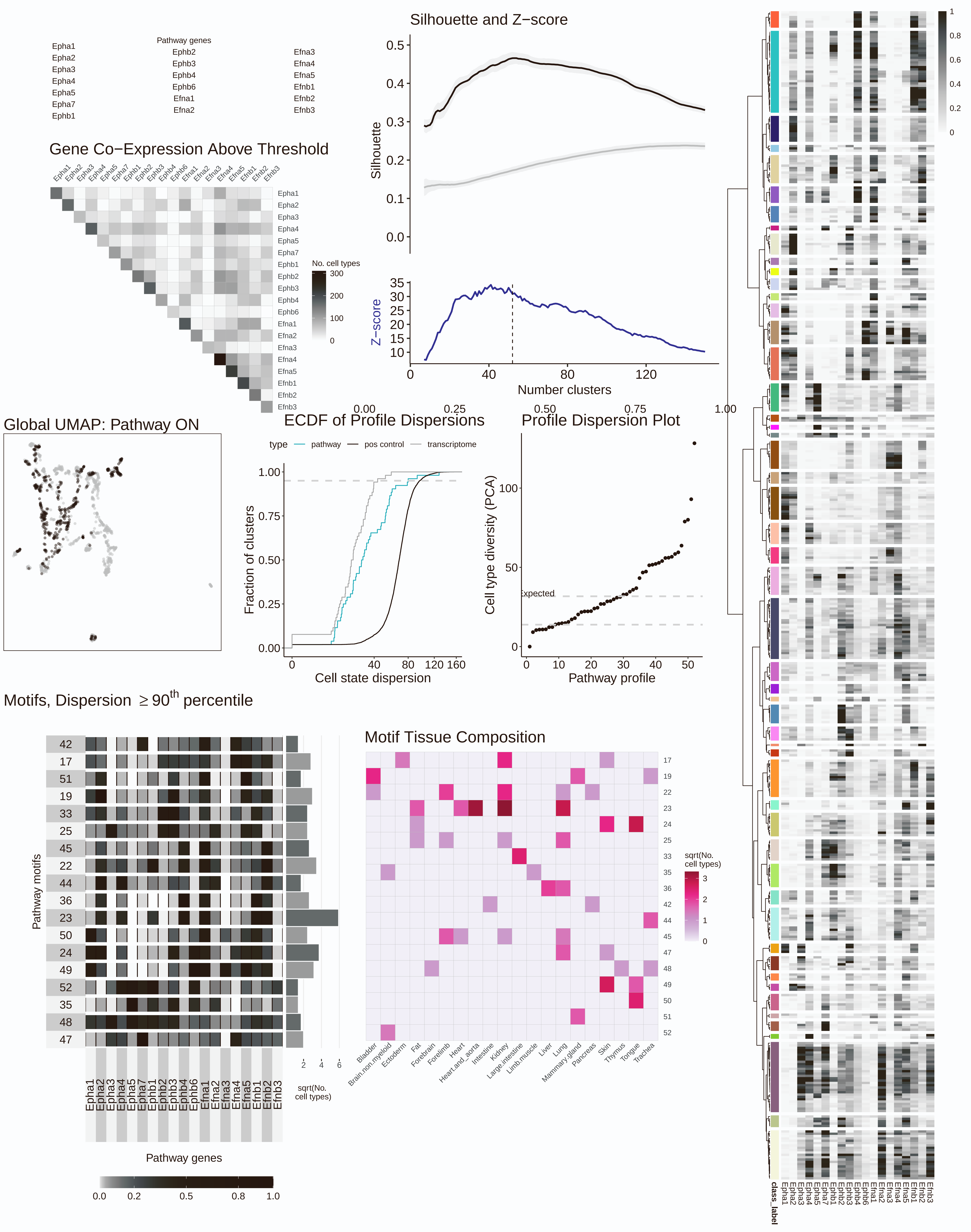

Eph receptors and ligands (k_opt = 52)

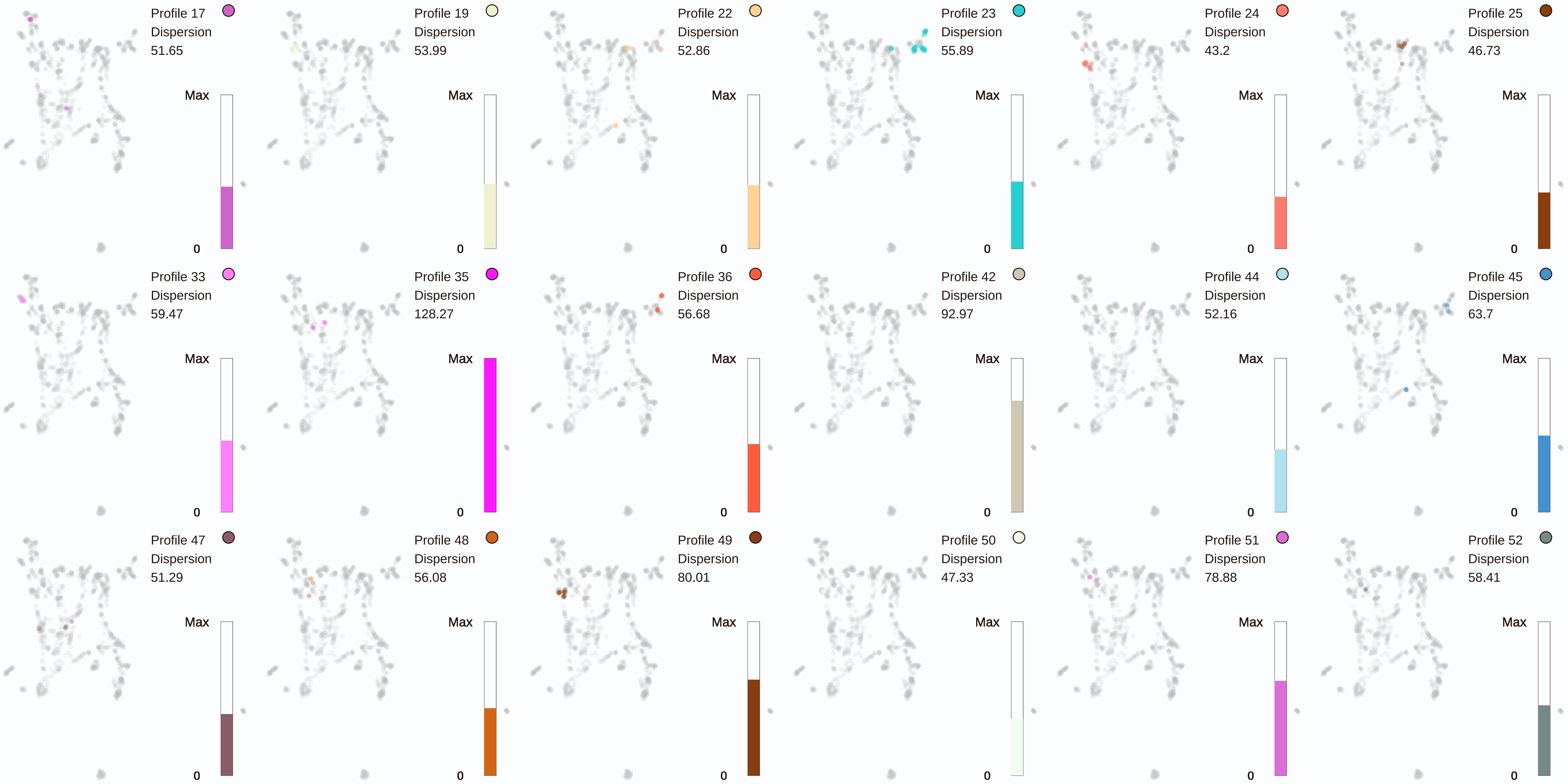

Eph_I (k_opt = 16)

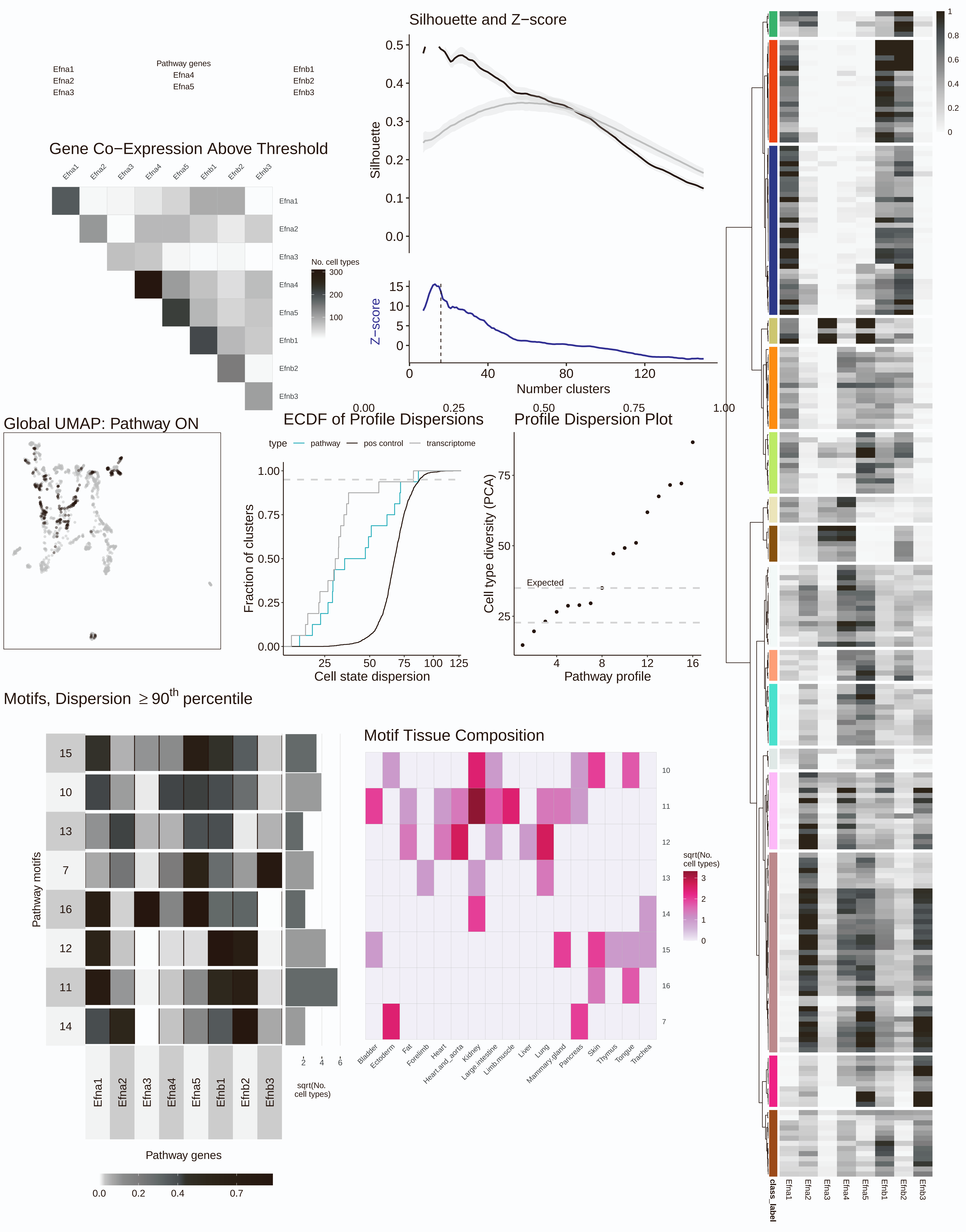
Silhouette and Z−score
Silhouette
Z−score
Number clusters
Pathway genes
Efna1
Efna2
Efna3
Efna4
Efna5
Efnb1
Efnb2
Efnb3
Gene Co−Expression Above Threshold
No. cell types
Global UMAP: Pathway ON
ECDF of Profile Dispersions
type
pathway
pos control
transcriptome
Fraction of clusters
Cell state dispersion
Profile Dispersion Plot
Expected
Cell type diversity (PCA)
Pathway profile
Motifs, Dispersion ≥ 90th percentile
Pathway motifs
sqrt(No. cell types)
Motif Tissue Composition
Bladder
Ectoderm
Fat
Forelimb
Heart
Heart_and_aorta
Kidney
Large.intestine
Limb.muscle
Liver
Lung
Mammary.gland
Pancreas
Skin
Thymus
Tongue
Trachea
class_label

Eph_I (k_opt = 16)

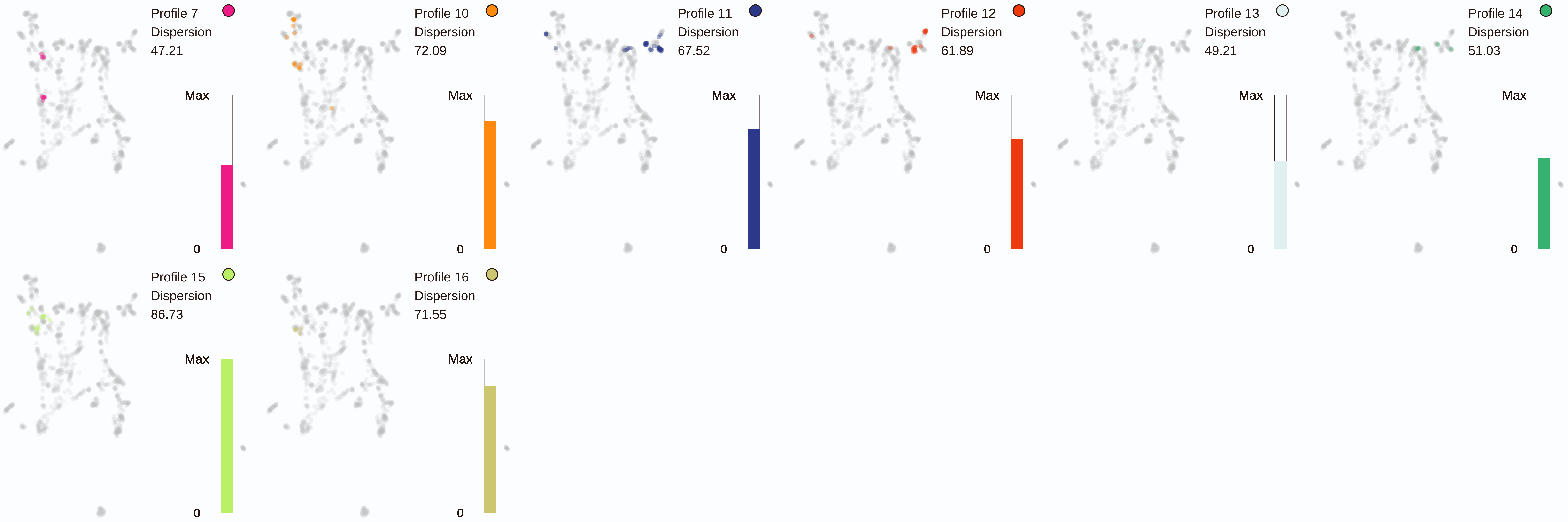
Profile 7
Dispersion
47.21
Max
0
Profile 10
Dispersion
72.09
Max
0
Profile 11
Dispersion
67.52
Max
0
Profile 12
Dispersion
61.89
Max
0
Profile 13
Dispersion
49.21
Max
0
Profile 14
Dispersion
51.03
Max
0
Profile 15
Dispersion
86.73
Max
0
Profile 16
Dispersion
71.55
Max
0

FGF cell signaling proteins (k_opt = 80)

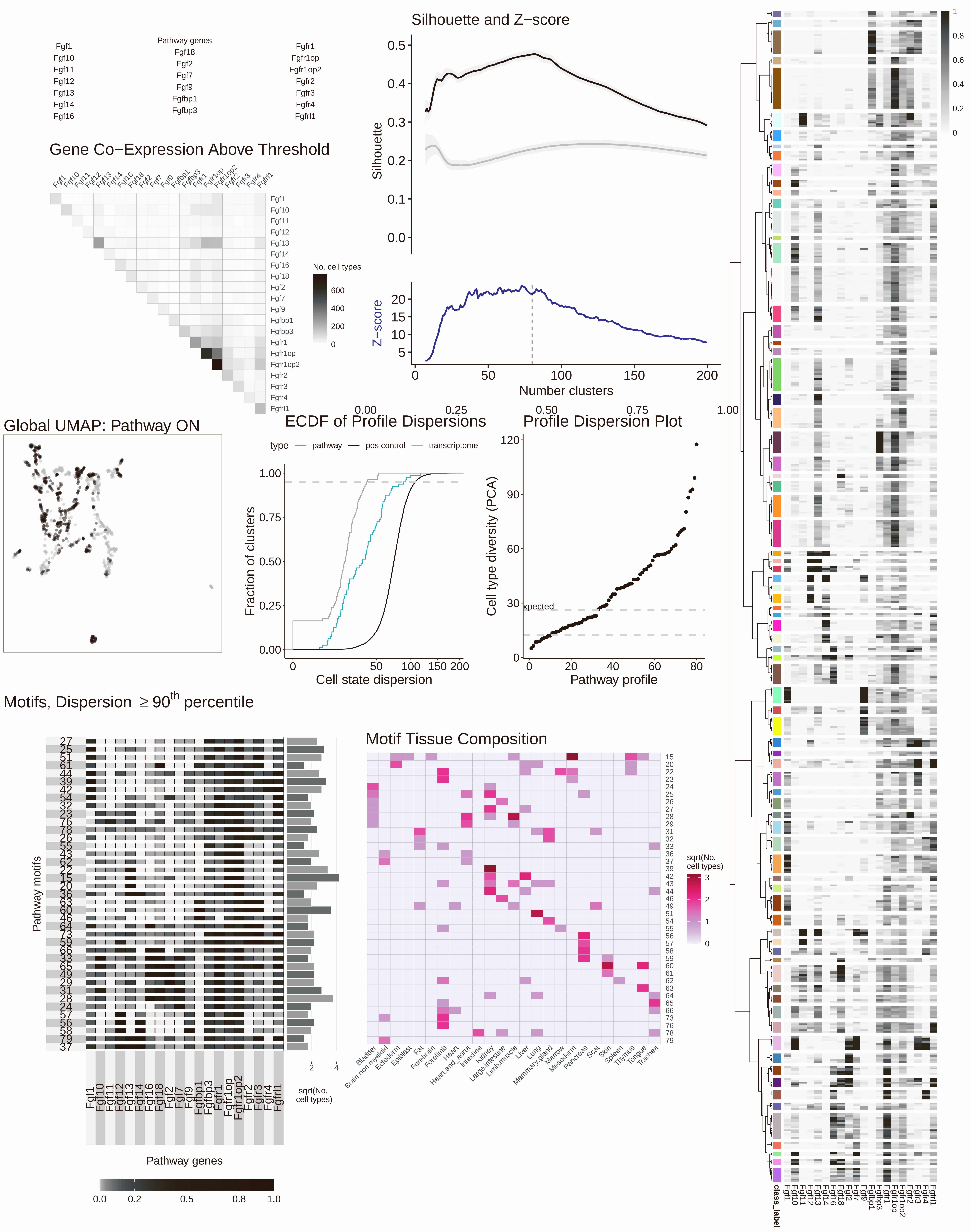

FGF cell signaling proteins (k_opt = 80)

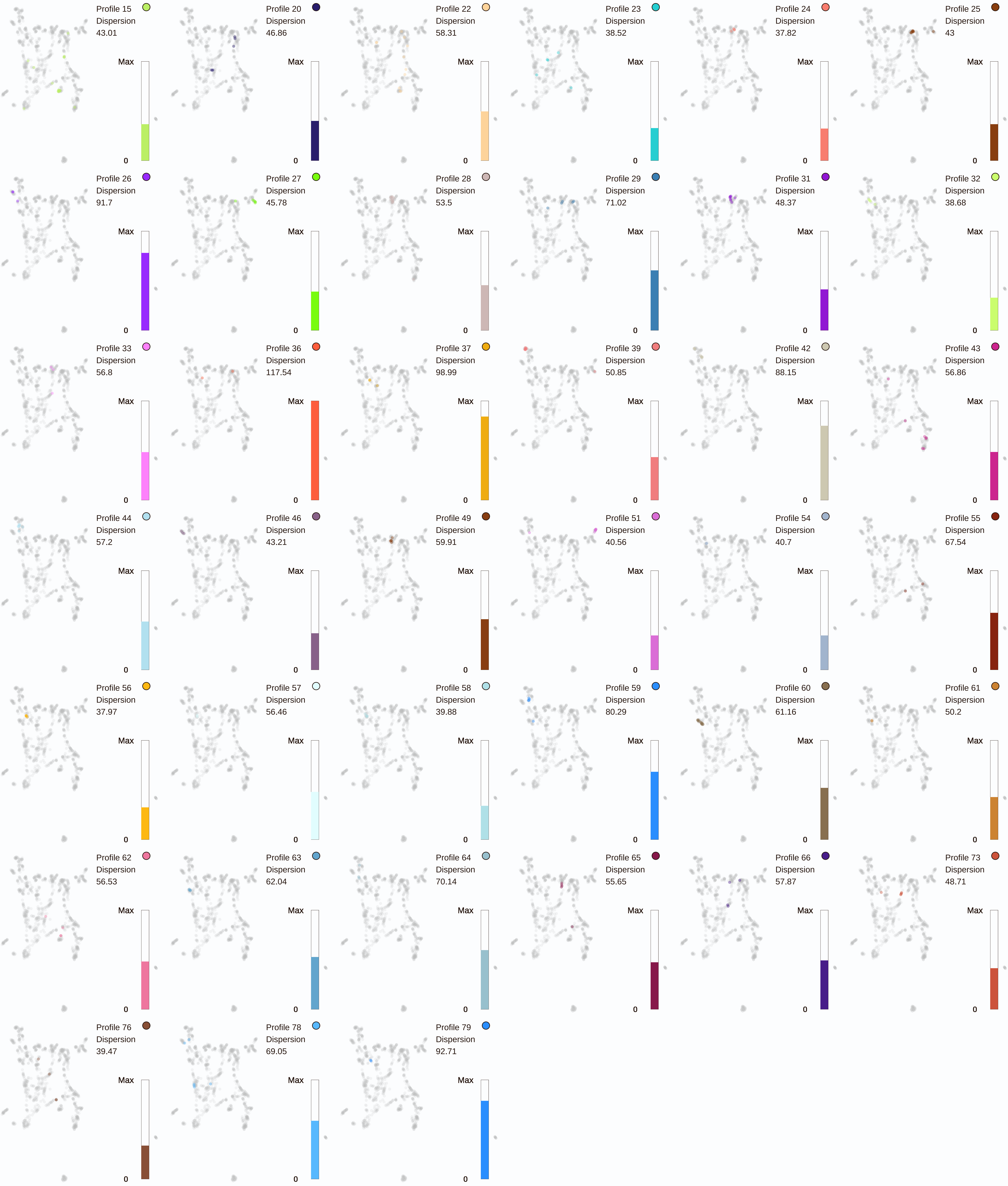

Frizzled and Lrp5 6 receptors for Wnt B Catenin Signaling (k_opt = 37)

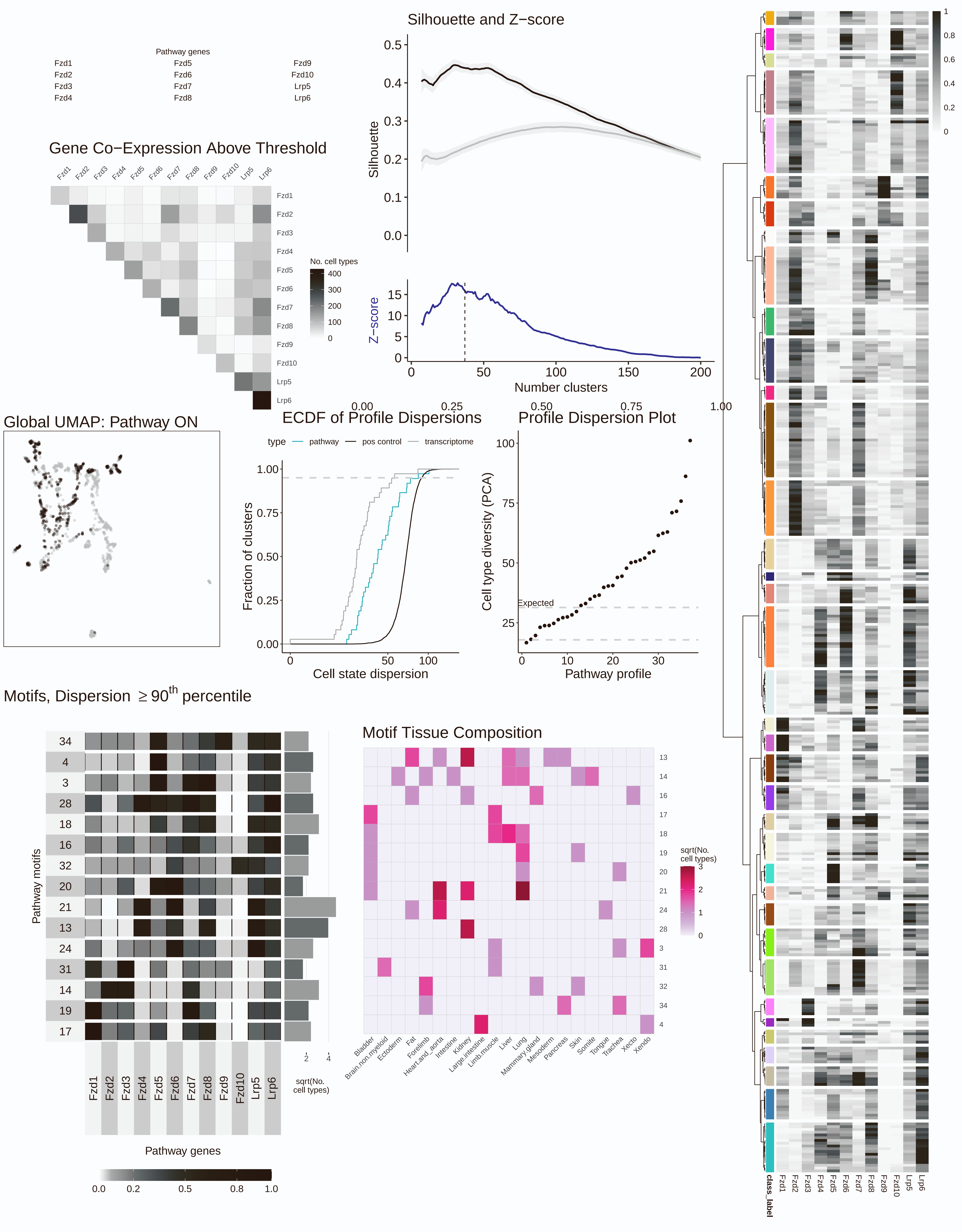

Frizzled and Lrp5 6 receptors for Wnt B Catenin Signaling (k_opt = 37)

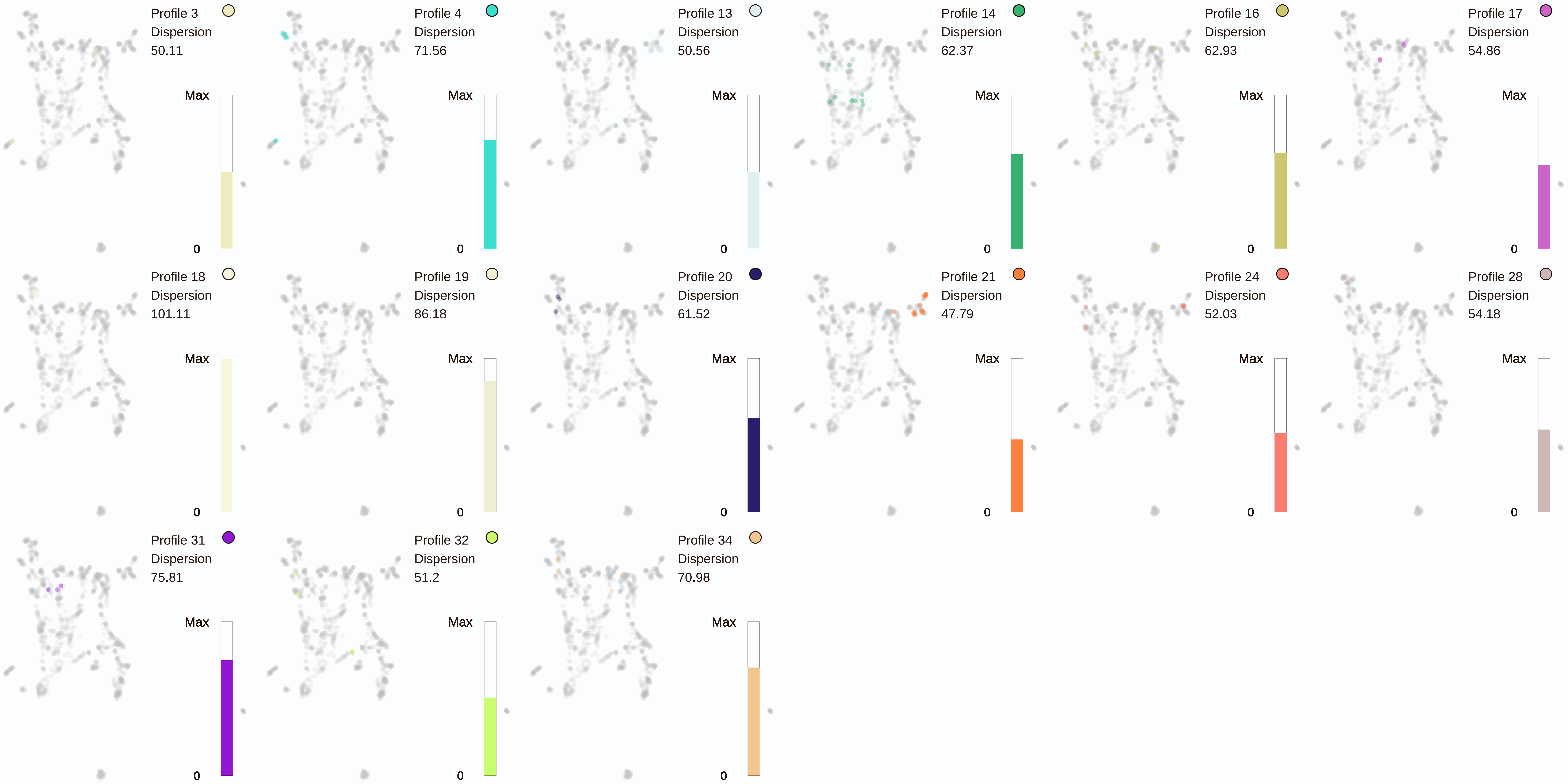

acellular Signalling Through Adenosine Receptor A2a and Adenosine (k_opt =

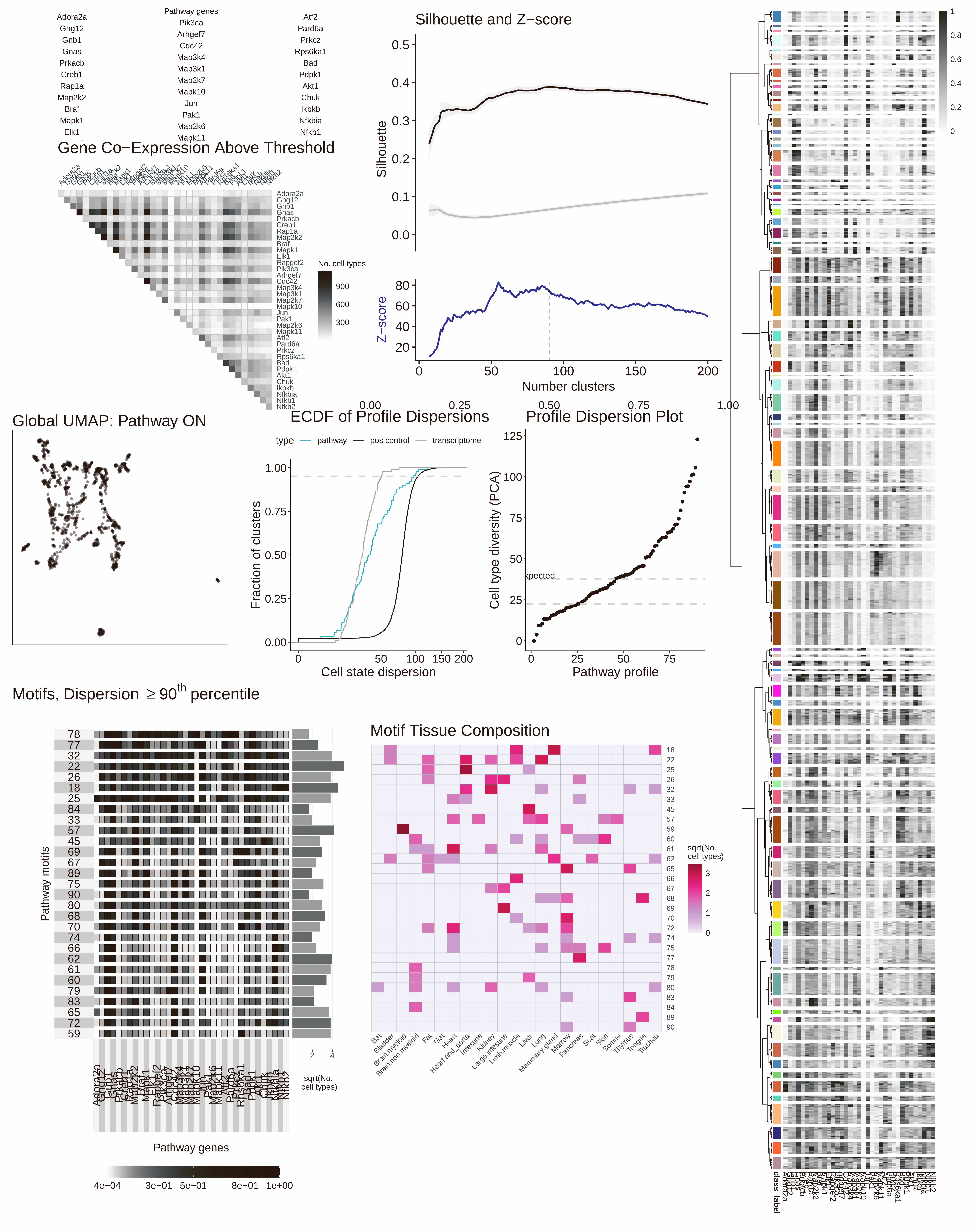

acellular Signalling Through Adenosine Receptor A2a and Adenosine (k_opt =

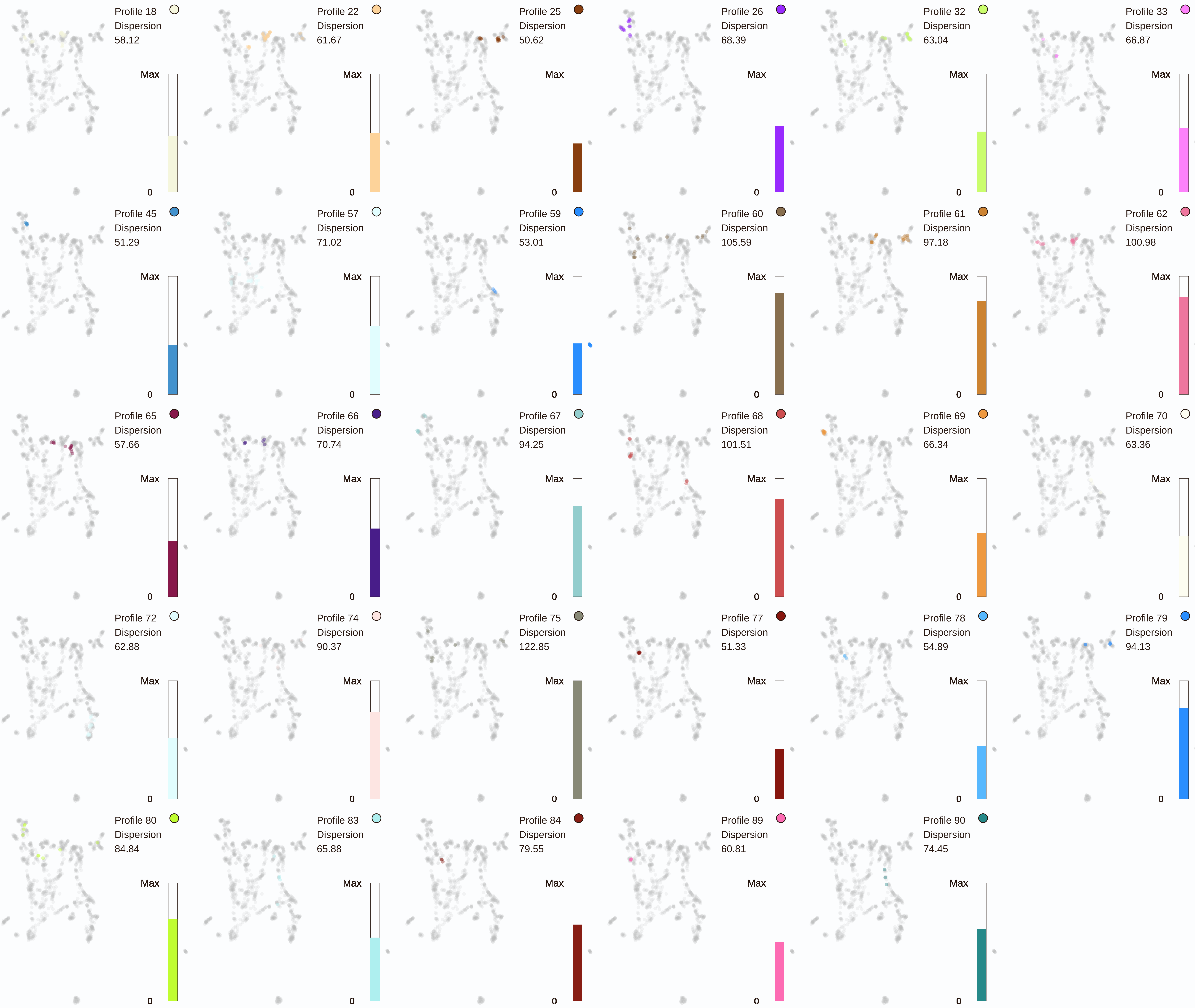

Ion Channels and Their Functional Role in Vascular Endothelium (k_opt = 32)

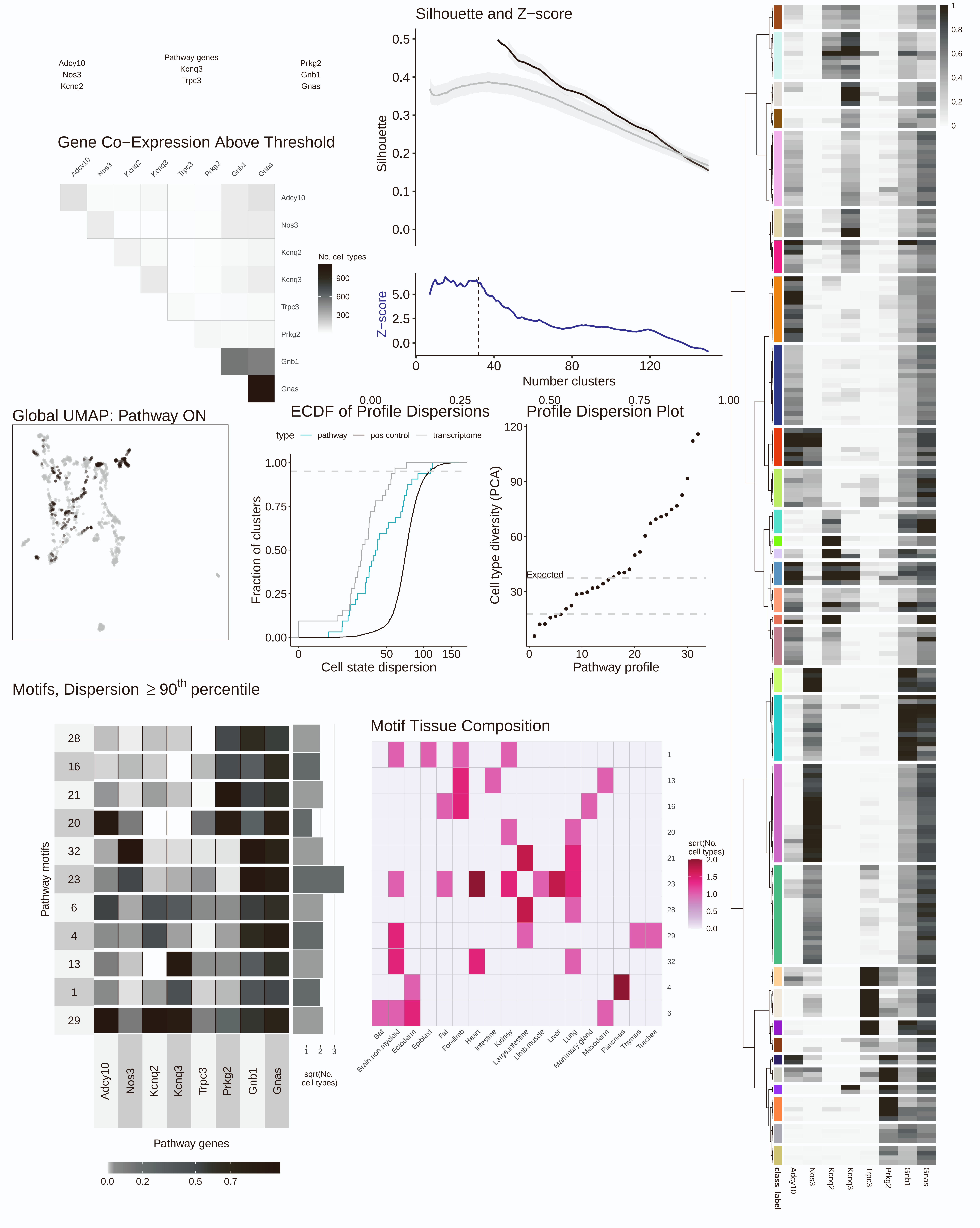

Ion Channels and Their Functional Role in Vascular Endothelium (k_opt = 32)

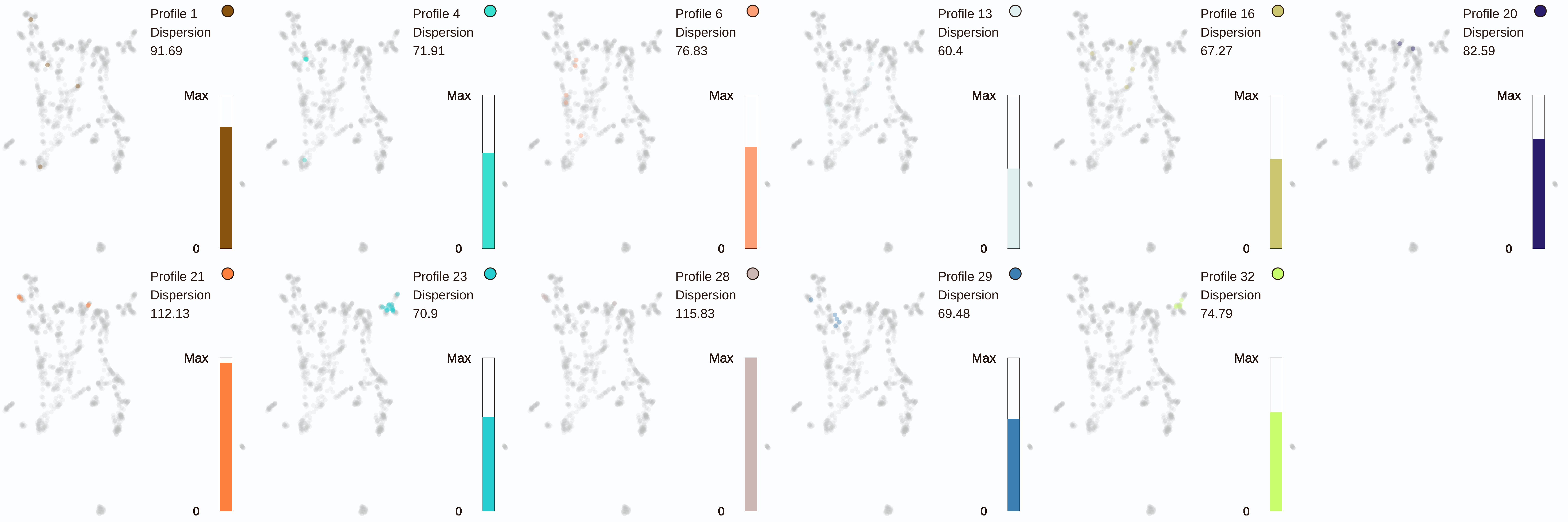

Leucine Stimulation on Insulin Signaling (k_opt = 34)

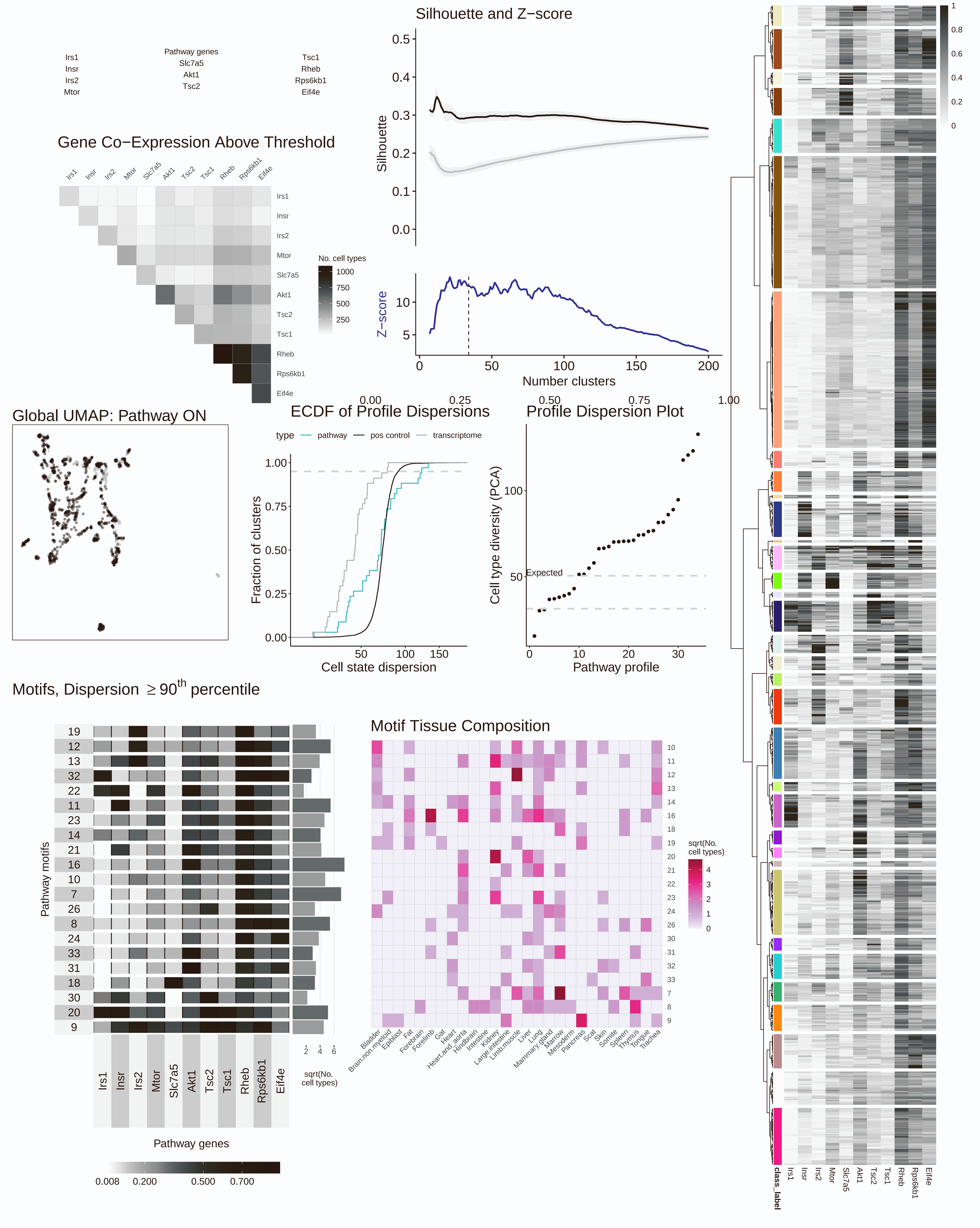

Leucine Stimulation on Insulin Signaling (k_opt = 34)

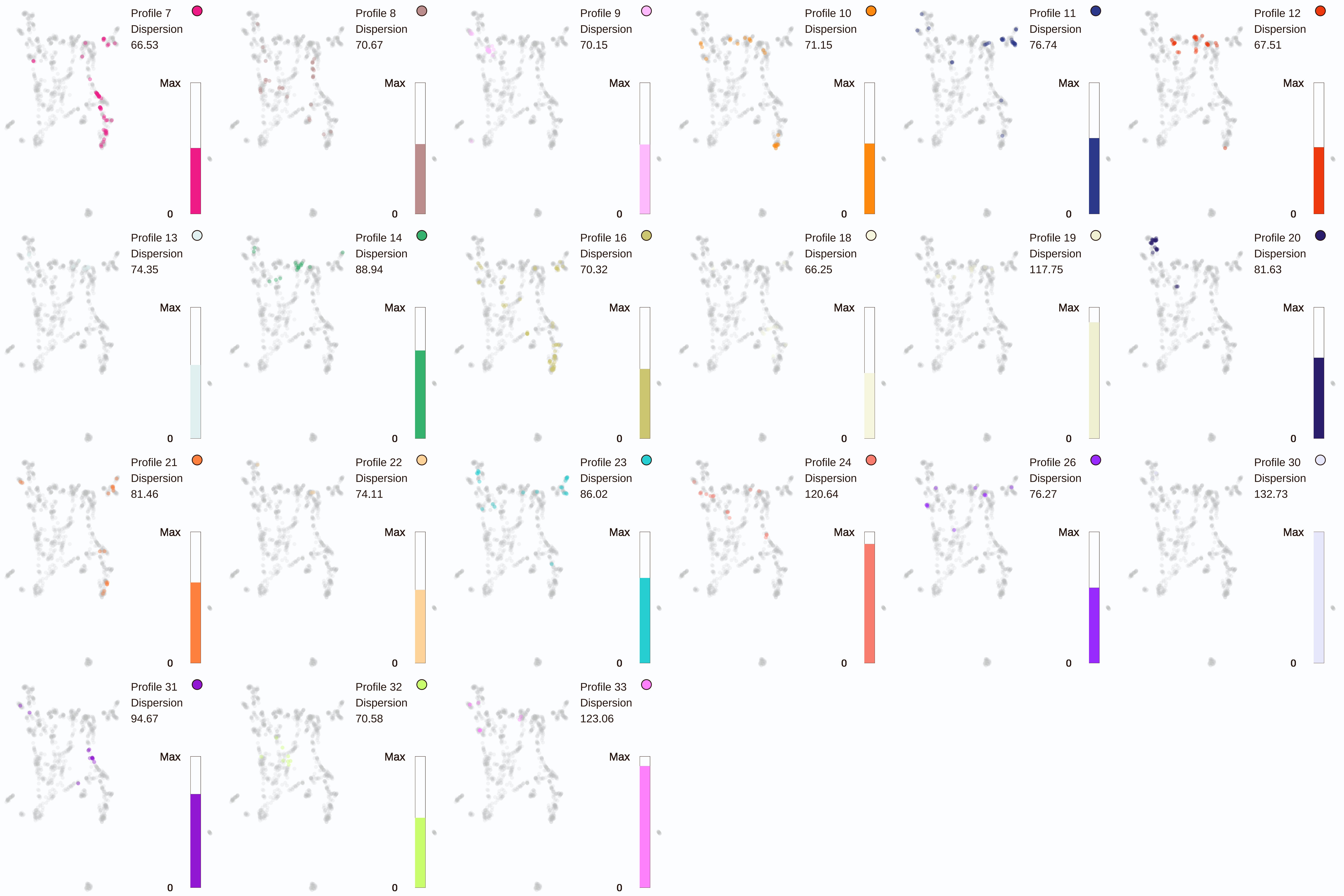

LPS and Citrate Signaling and Inflammation (k_opt = 112)

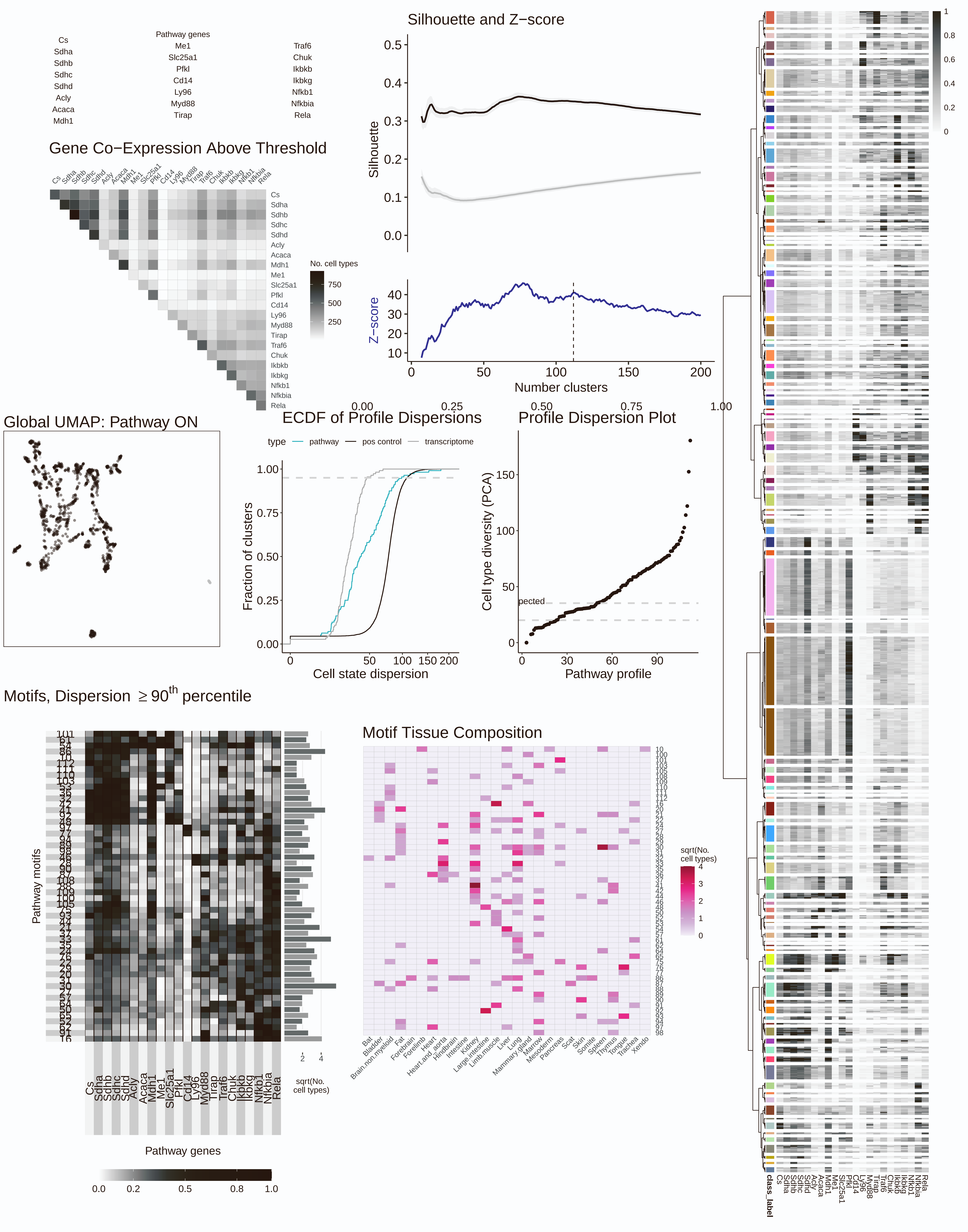

LPS and Citrate Signaling and Inflammation (k_opt = 112)

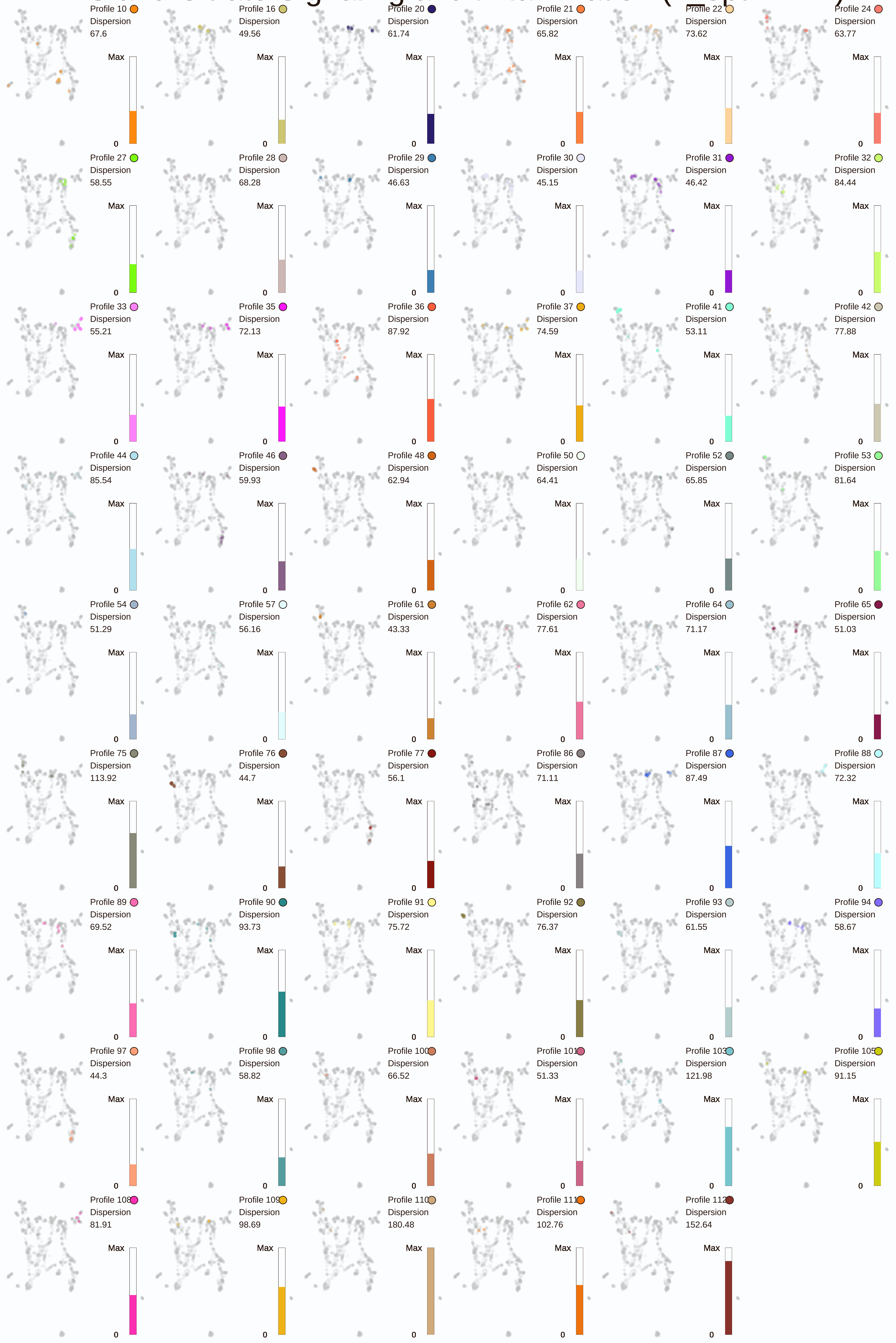

Lysophosphatidic Acid LPA2 Signalling (k_opt = 31)

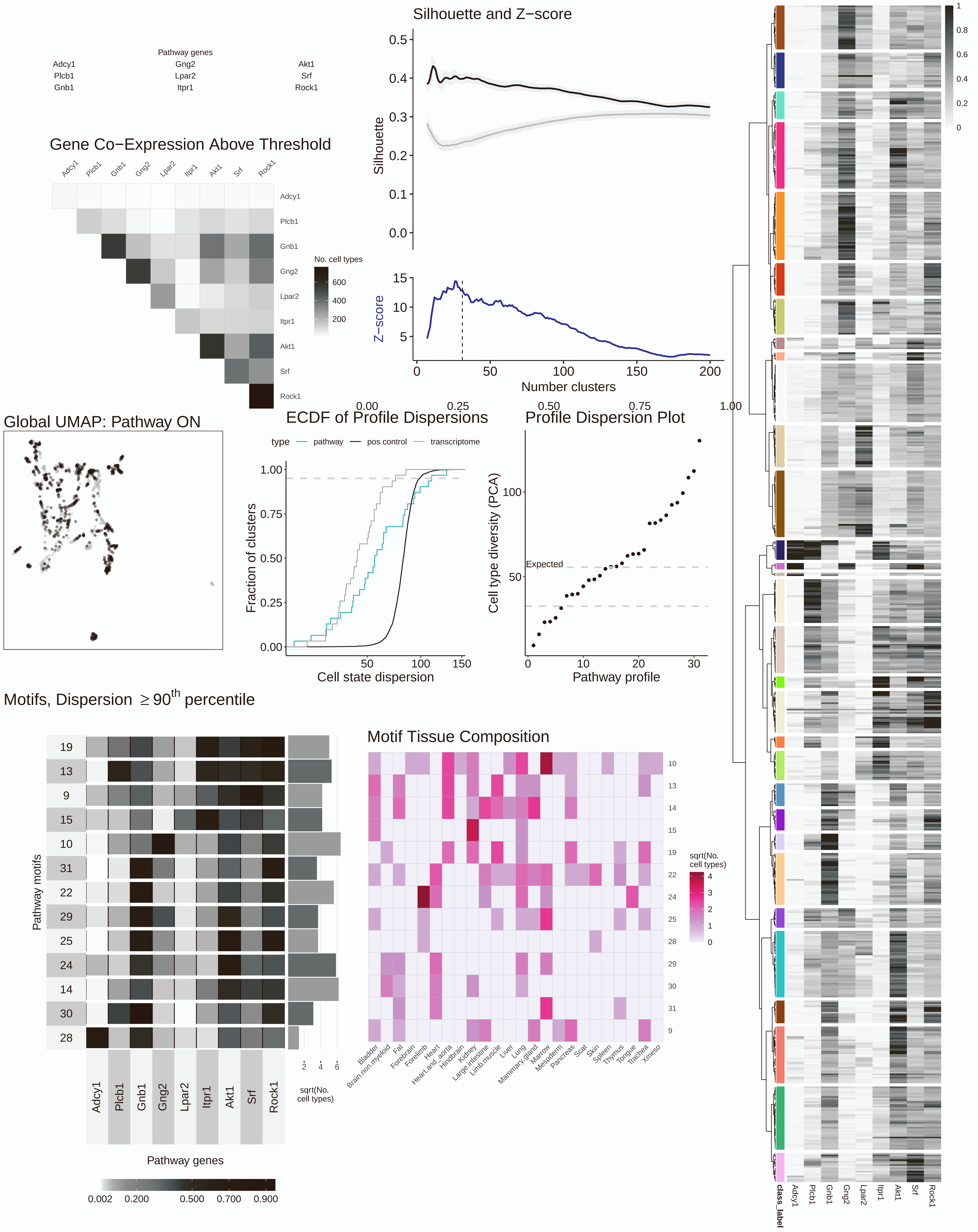

Lysophosphatidic Acid LPA2 Signalling (k_opt = 31)

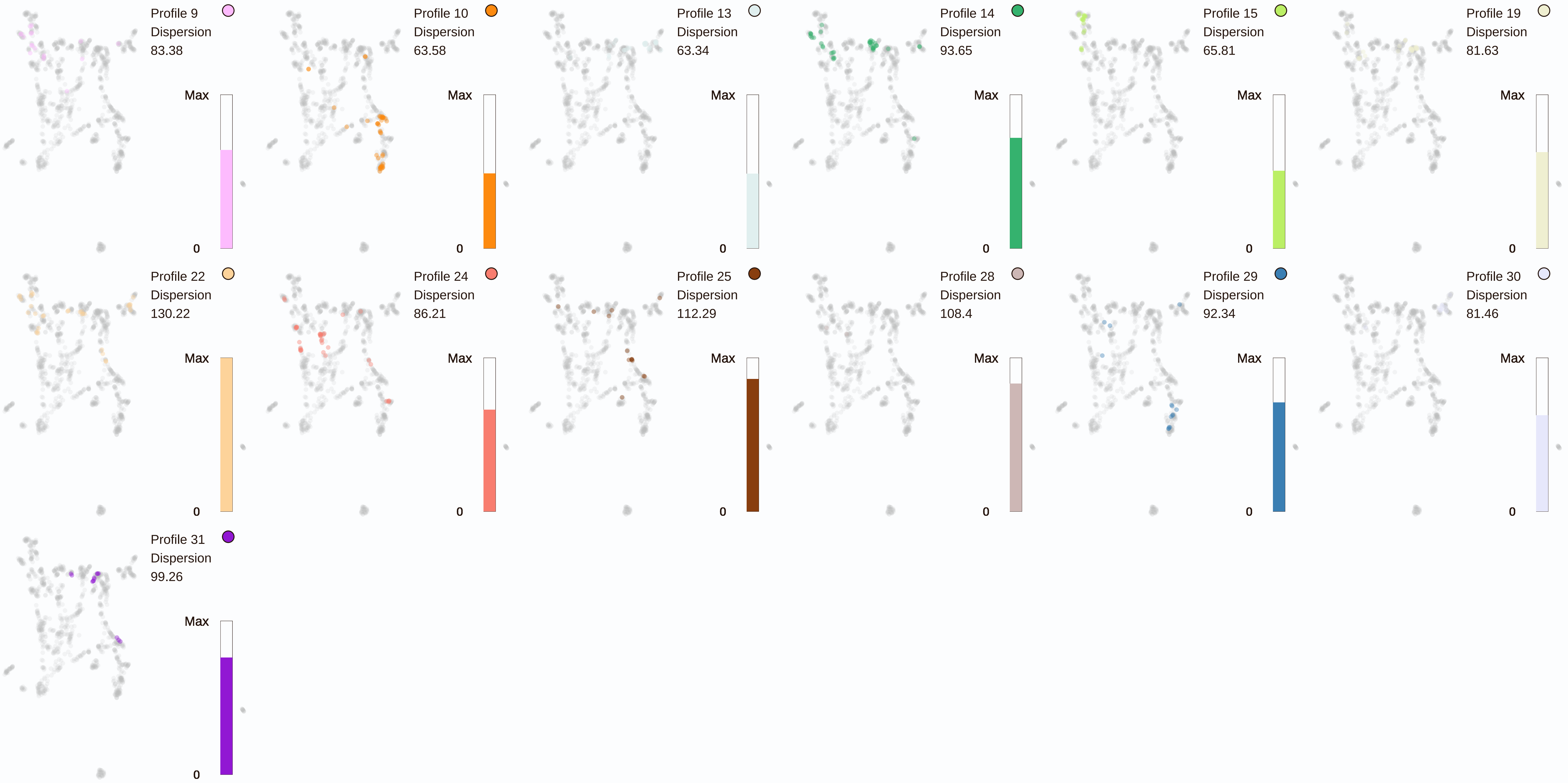

Mitochondrial Electron Transport Chain (k_opt = 43)

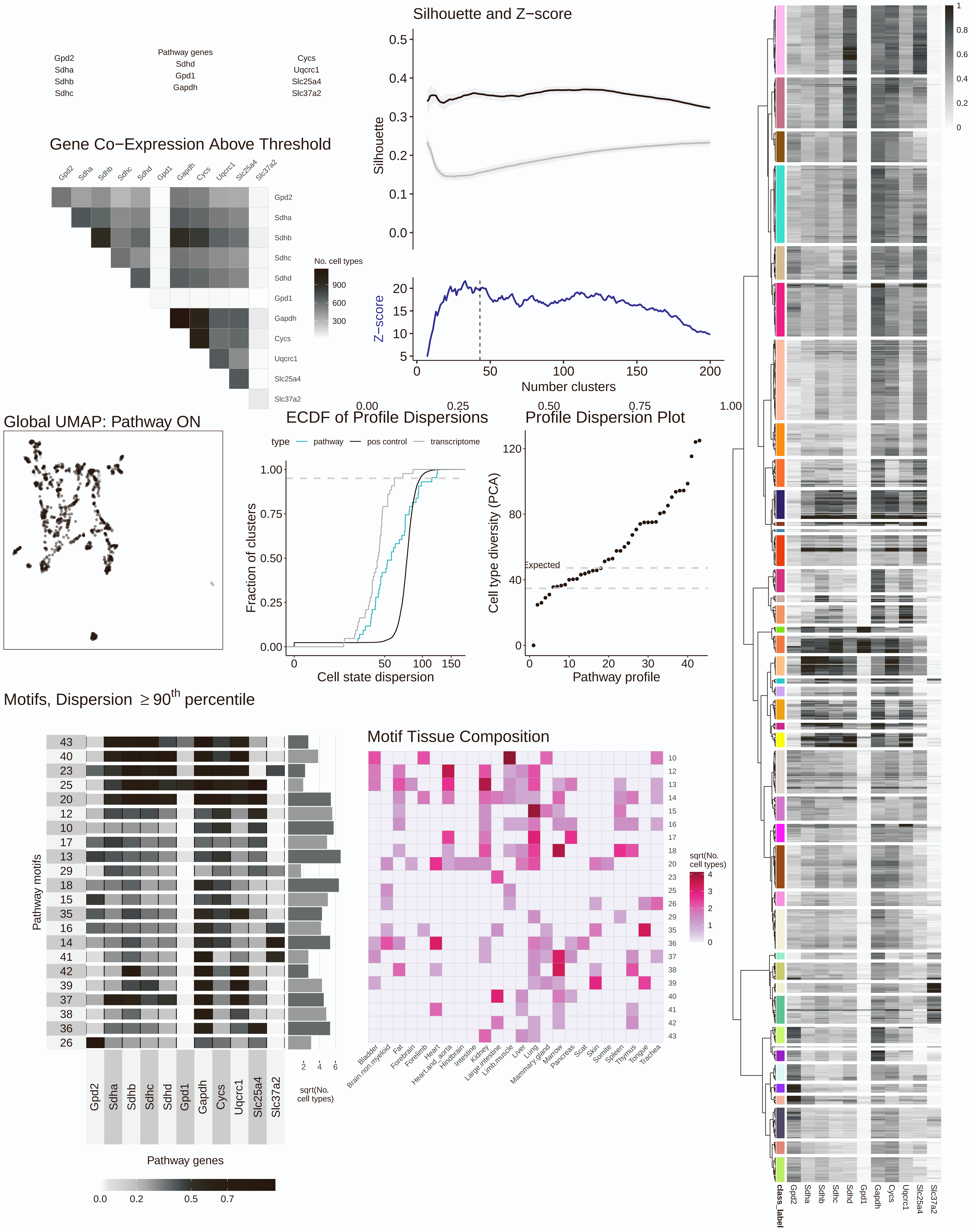

Mitochondrial Electron Transport Chain (k_opt = 43)

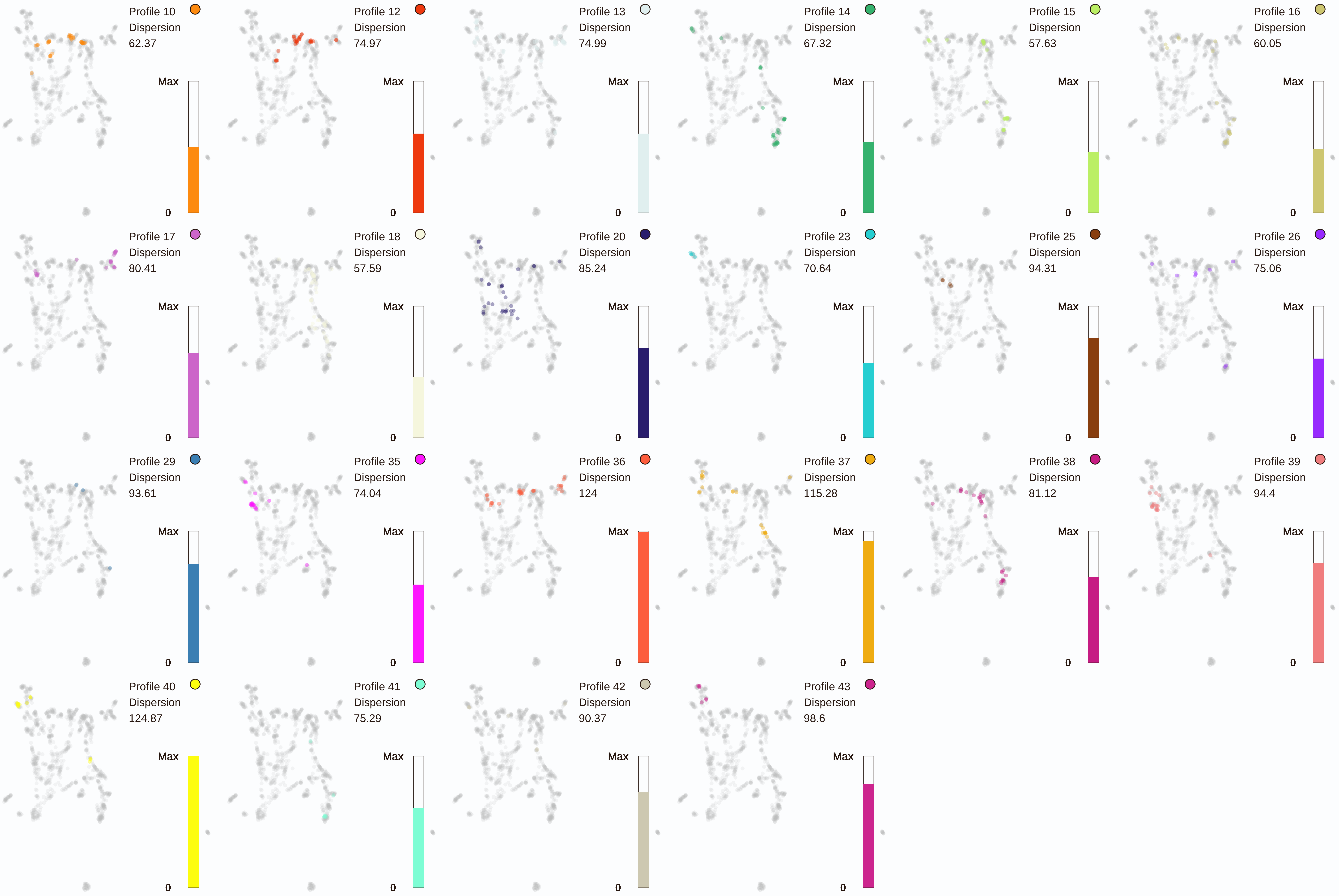

NF−kB Signaling Pathway (k_opt = 113)

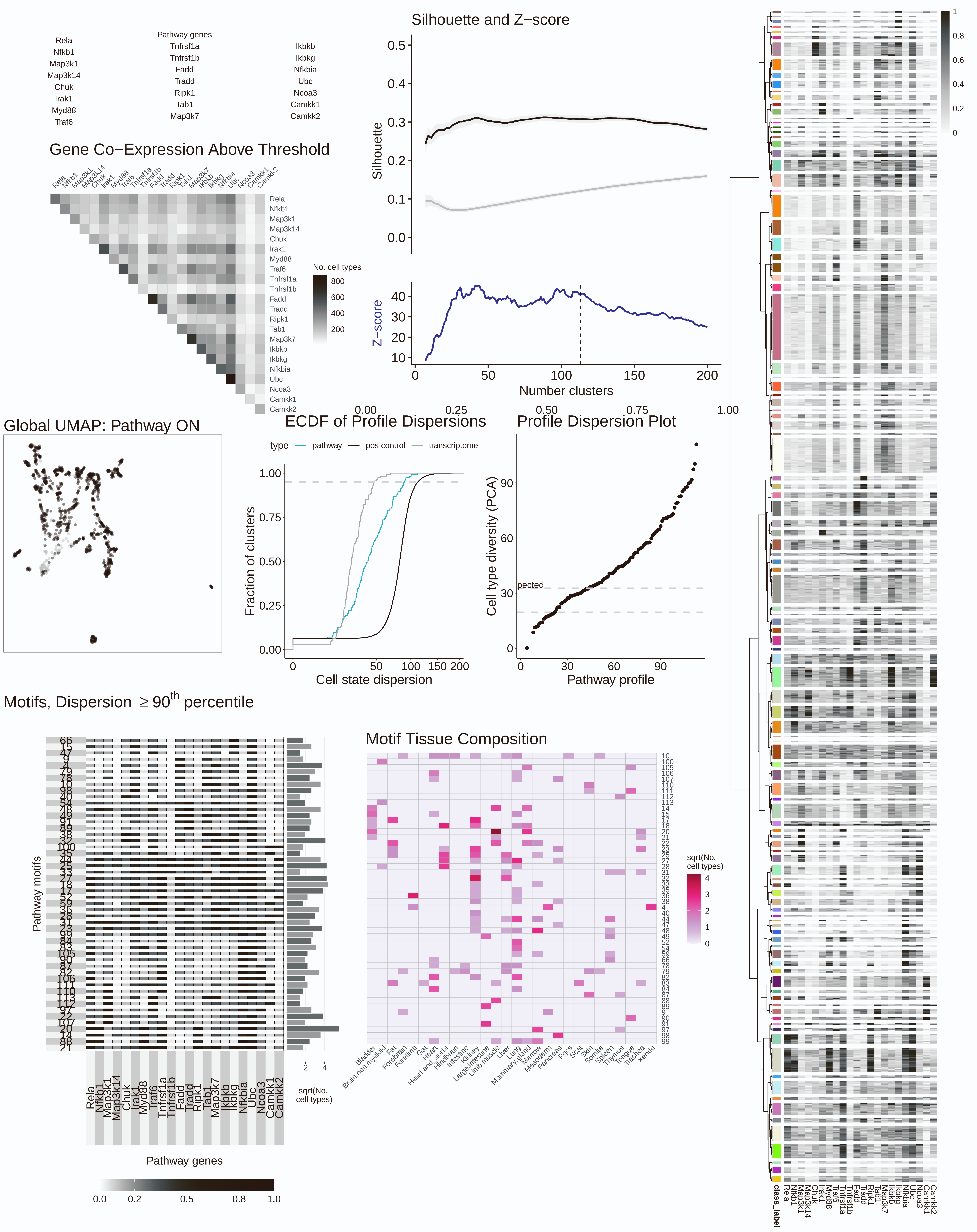

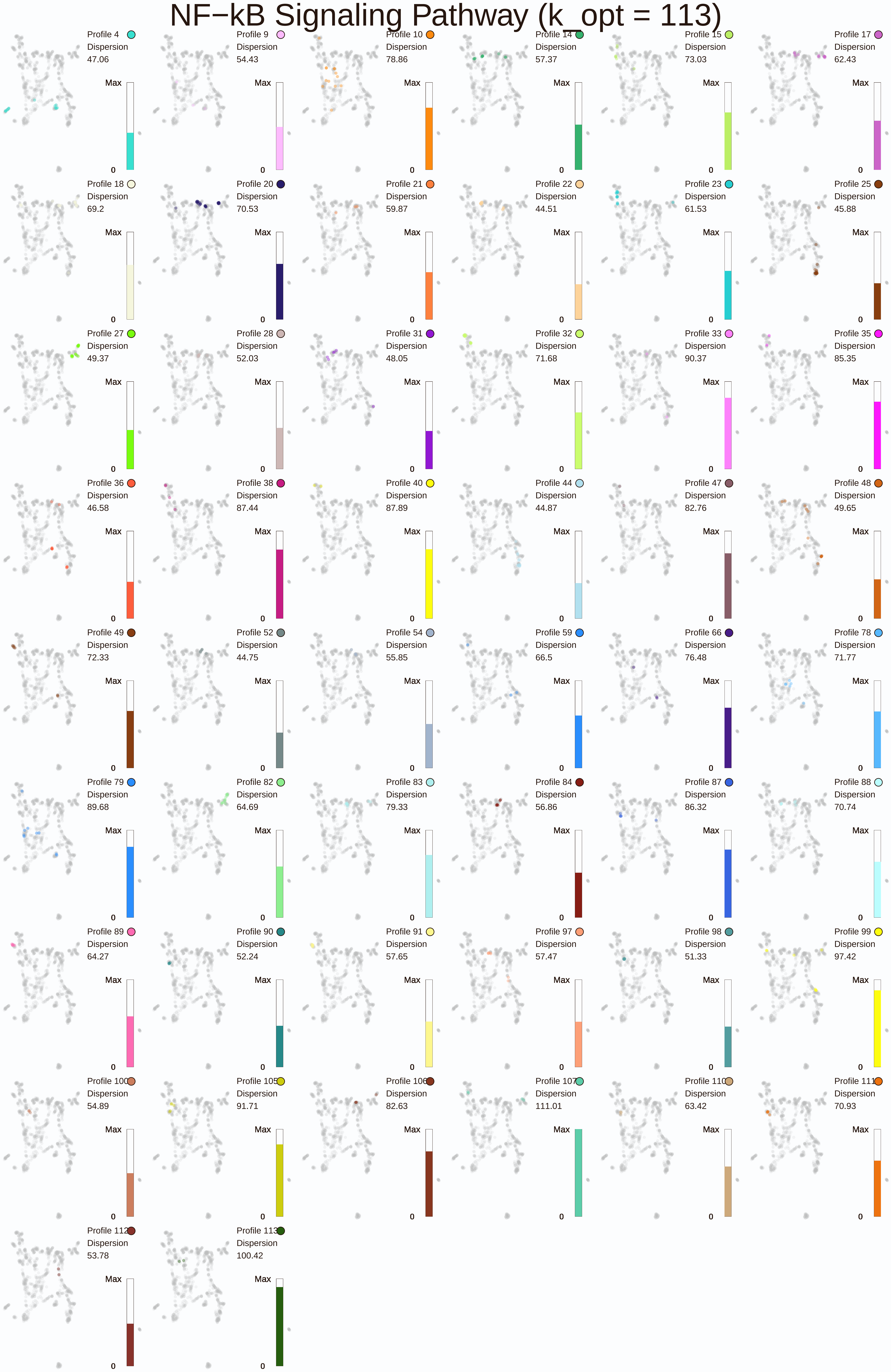
NF−kB Signaling Pathway (k_opt = 113)
Profile 4 Dispersion 47.06 Max 0
Profile 9 Dispersion 54.43 Max 0
Profile 10 Dispersion 78.86 Max 0
Profile 14 Dispersion 57.37 Max 0
Profile 15 Dispersion 73.03 Max 0
Profile 17 Dispersion 62.43 Max 0
Profile 18 Dispersion 69.2 Max 0
Profile 20 Dispersion 70.53 Max 0
Profile 21 Dispersion 59.87 Max 0
Profile 22 Dispersion 44.51 Max 0
Profile 23 Dispersion 61.53 Max 0
Profile 25 Dispersion 45.88 Max 0
Profile 27 Dispersion 49.37 Max 0
Profile 28 Dispersion 52.03 Max 0
Profile 31 Dispersion 48.05 Max 0
Profile 32 Dispersion 71.68 Max 0
Profile 33 Dispersion 90.37 Max 0
Profile 35 Dispersion 85.35 Max 0
Profile 36 Dispersion 46.58 Max 0
Profile 38 Dispersion 87.44 Max 0
Profile 40 Dispersion 87.89 Max 0
Profile 44 Dispersion 44.87 Max 0
Profile 47 Dispersion 82.76 Max 0
Profile 48 Dispersion 49.65 Max 0
Profile 49 Dispersion 72.33 Max 0
Profile 52 Dispersion 44.75 Max 0
Profile 54 Dispersion 55.85 Max 0
Profile 59 Dispersion 66.5 Max 0
Profile 66 Dispersion 76.48 Max 0
Profile 78 Dispersion 71.77 Max 0
Profile 79 Dispersion 89.68 Max 0
Profile 82 Dispersion 64.69 Max 0
Profile 83 Dispersion 79.33 Max 0
Profile 84 Dispersion 56.86 Max 0
Profile 87 Dispersion 86.32 Max 0
Profile 88 Dispersion 70.74 Max 0
Profile 89 Dispersion 64.27 Max 0
Profile 90 Dispersion 52.24 Max 0
Profile 91 Dispersion 57.65 Max 0
Profile 97 Dispersion 57.47 Max 0
Profile 98 Dispersion 51.33 Max 0
Profile 99 Dispersion 97.42 Max 0
Profile 100 Dispersion 54.89 Max 0
Profile 105 Dispersion 91.71 Max 0
Profile 106 Dispersion 82.63 Max 0
Profile 107 Dispersion 111.01 Max 0
Profile 110 Dispersion 63.42 Max 0
Profile 111 Dispersion 70.93 Max 0
Profile 112 Dispersion 53.78 Max 0
Profile 113 Dispersion 100.42 Max 0

Nitric Oxide Signaling Pathway (k_opt = 28)

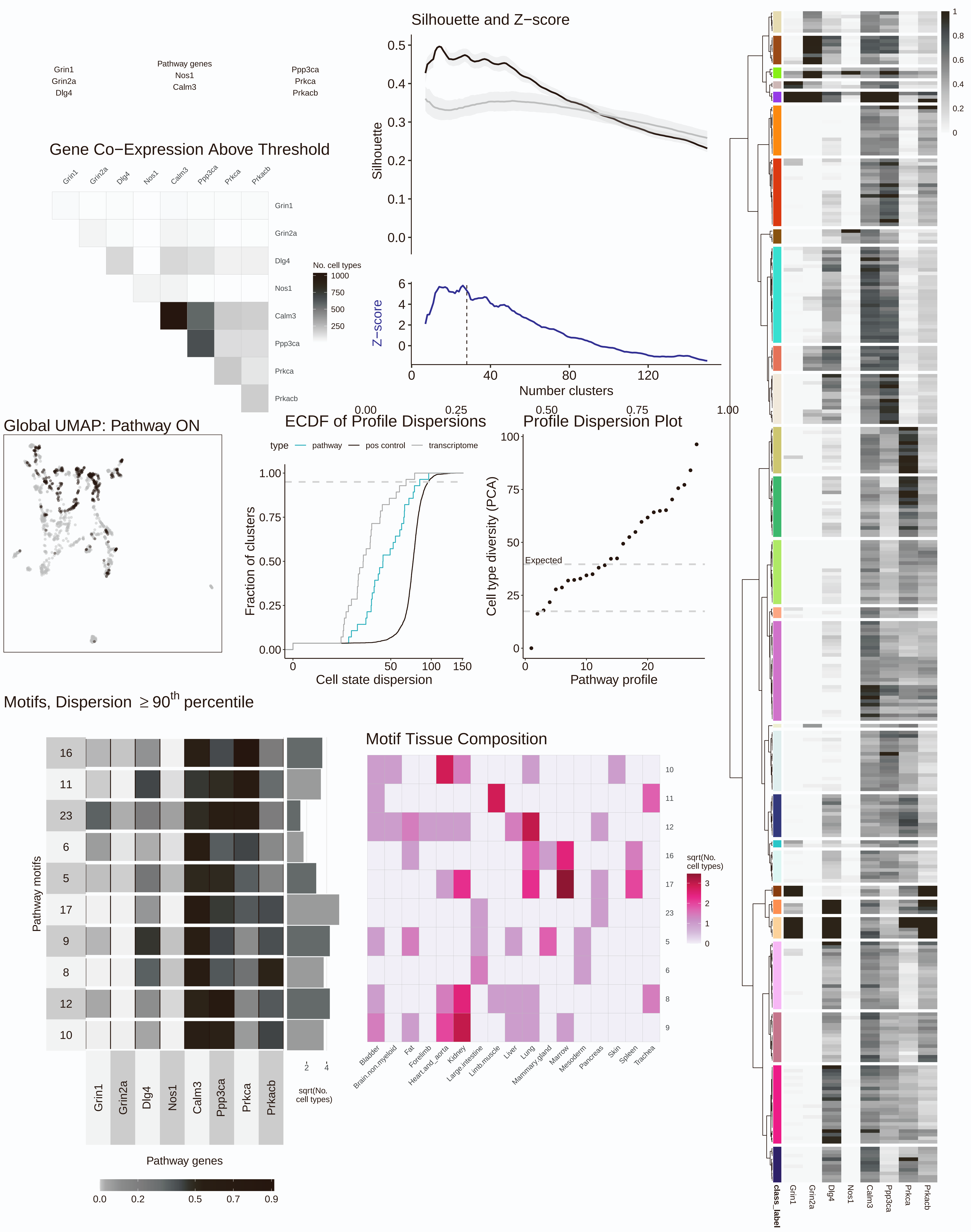

Nitric Oxide Signaling Pathway (k_opt = 28)

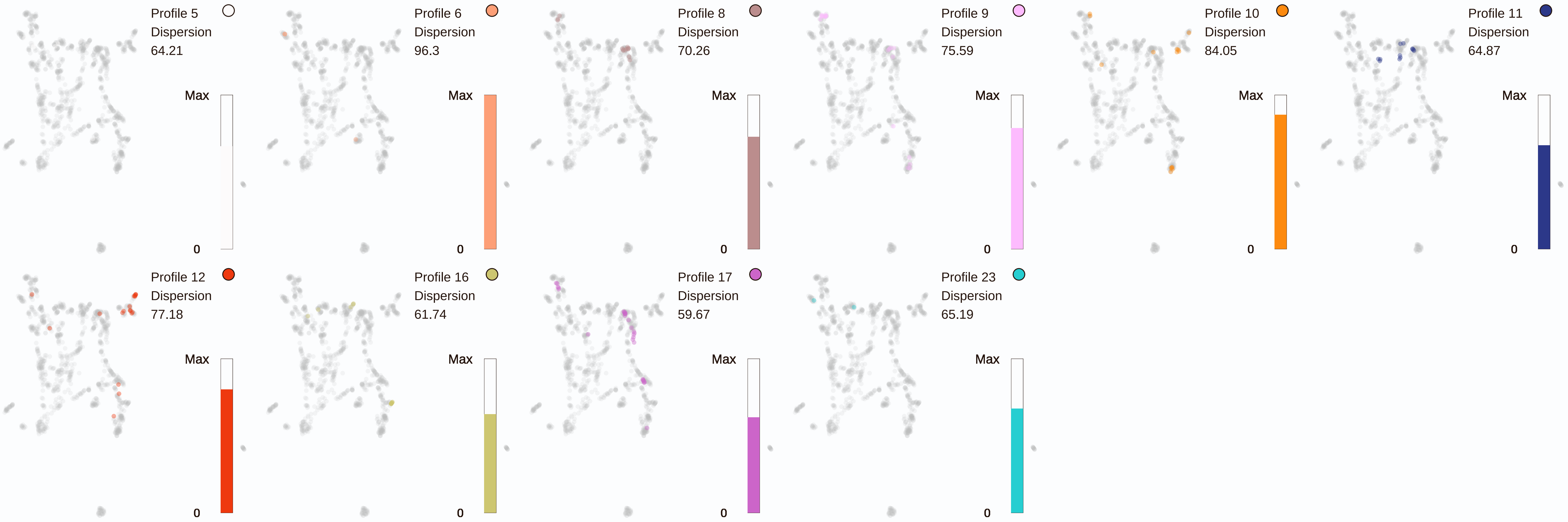

Notch receptors, Dll ligands and Fringe proteins (k_opt = 30)

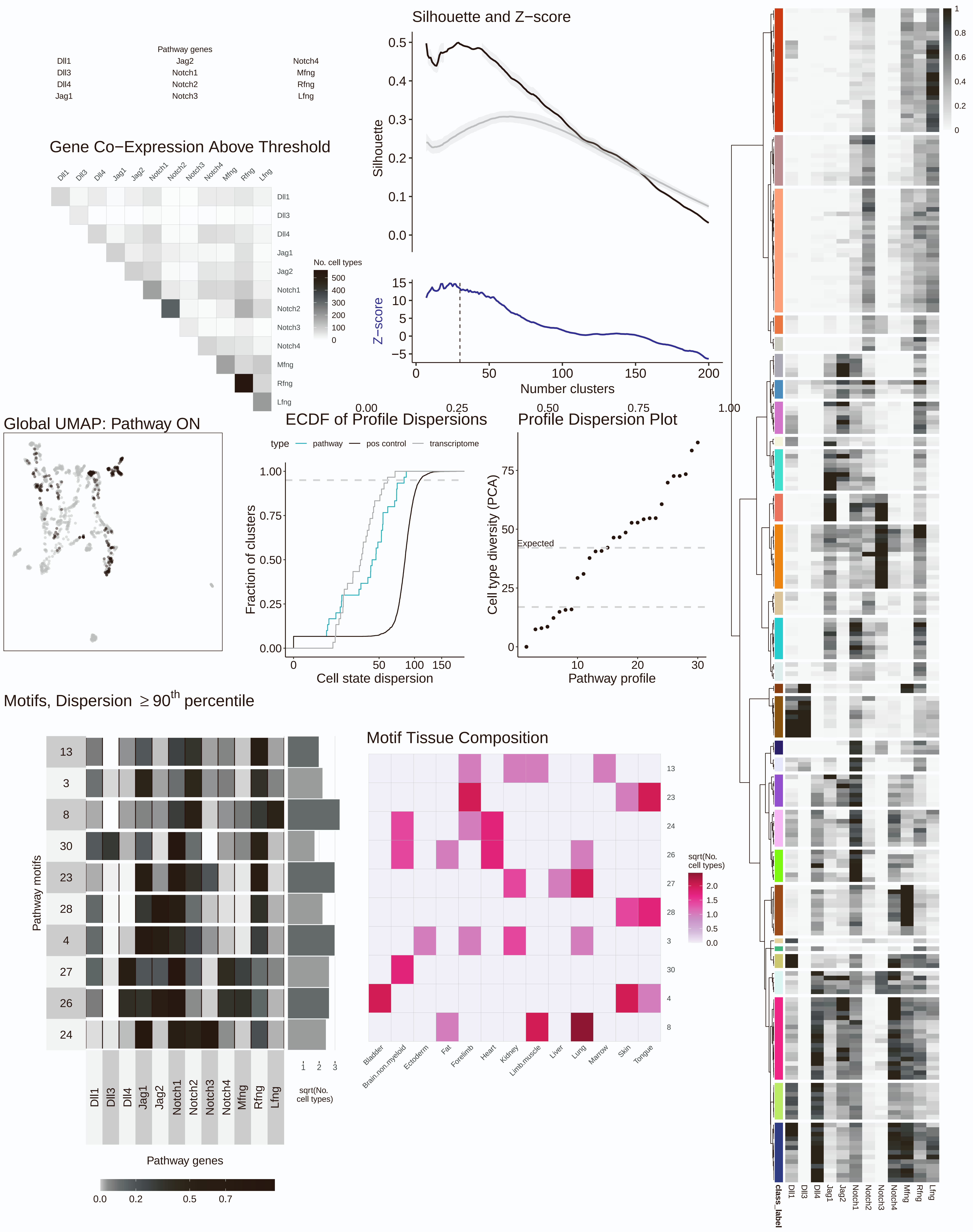

Notch receptors, Dll ligands and Fringe proteins (k_opt = 30)

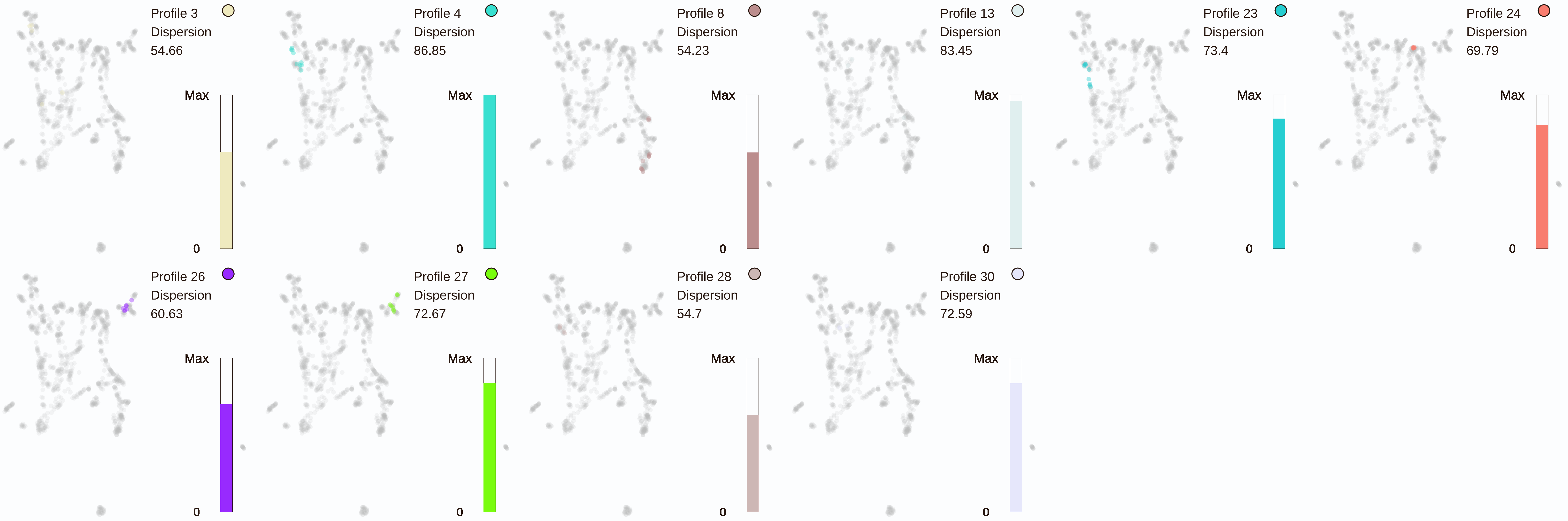

Ras Signaling Pathway (k_opt = 83)

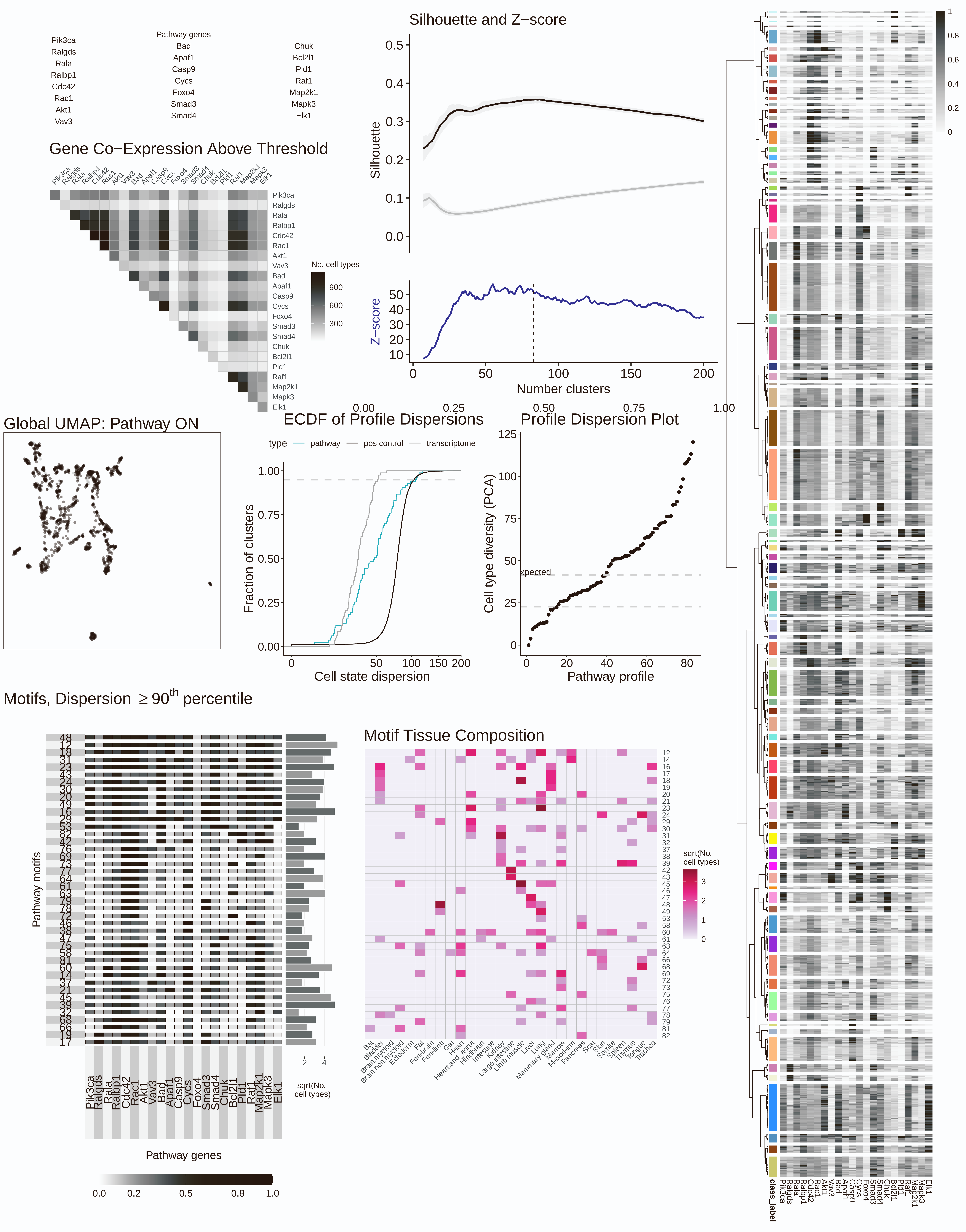

Ras Signaling Pathway (k_opt = 83)

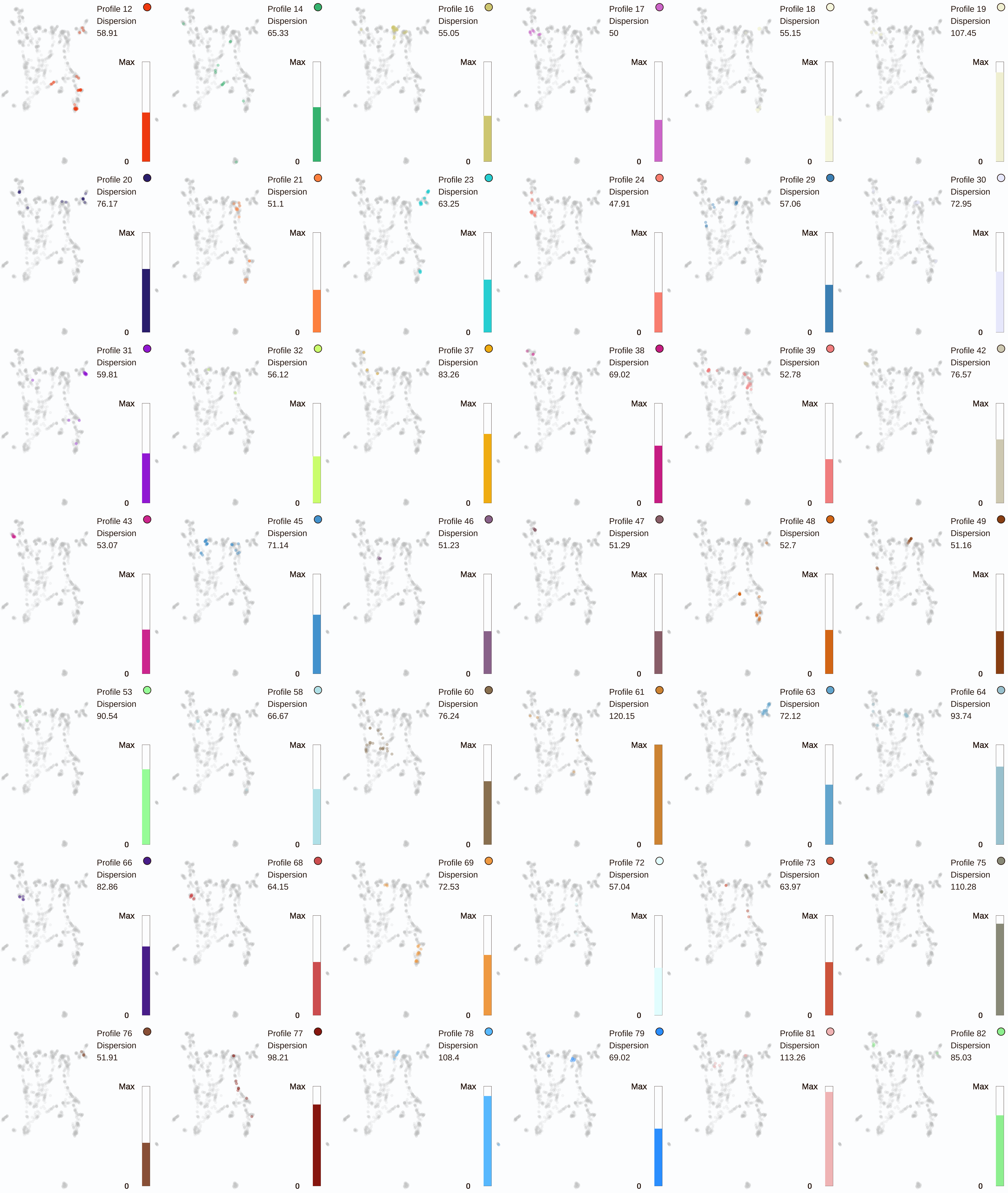

RNA−splicing by SR protein family (k_opt = 19)

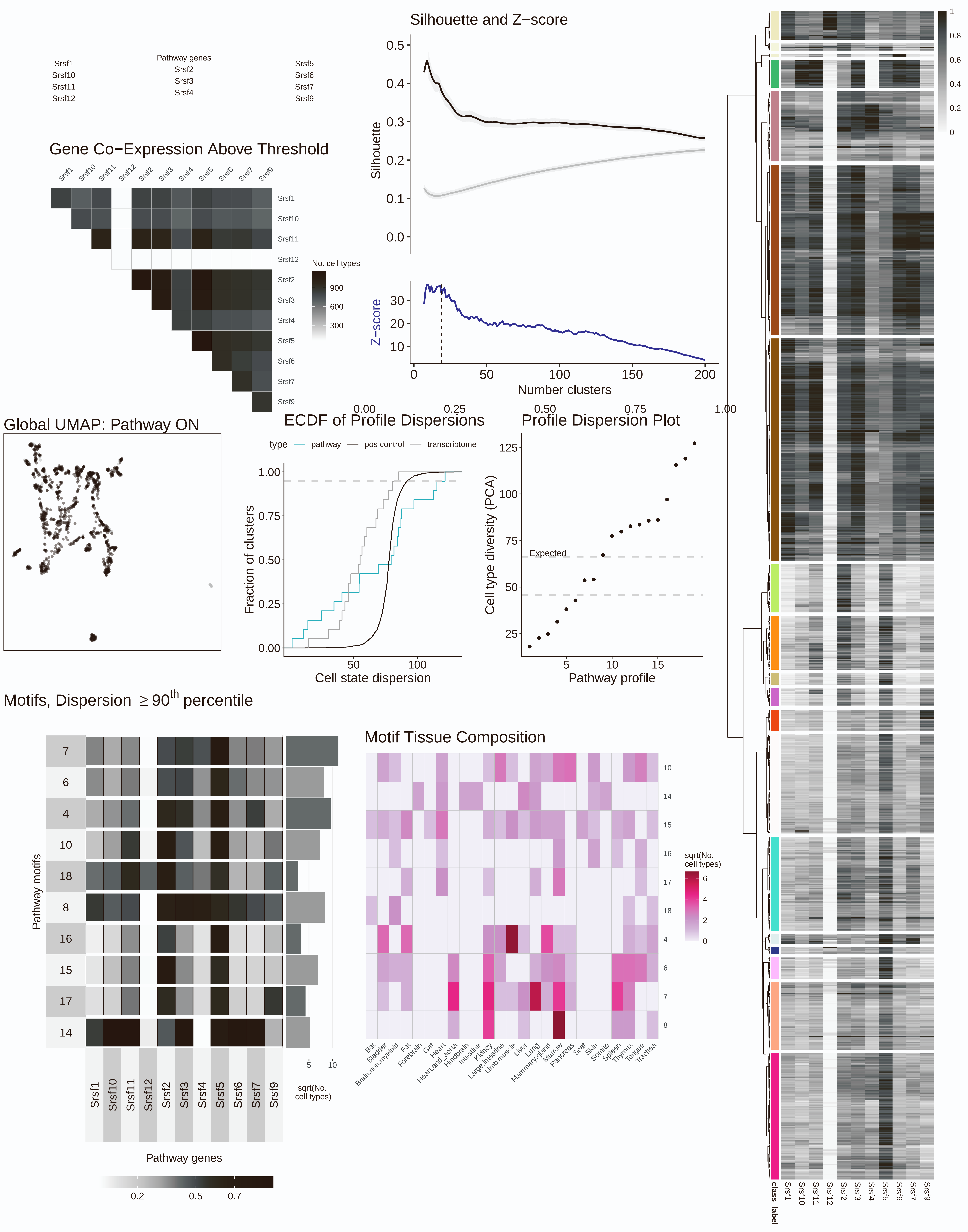

RNA−splicing by SR protein family (k_opt = 19)

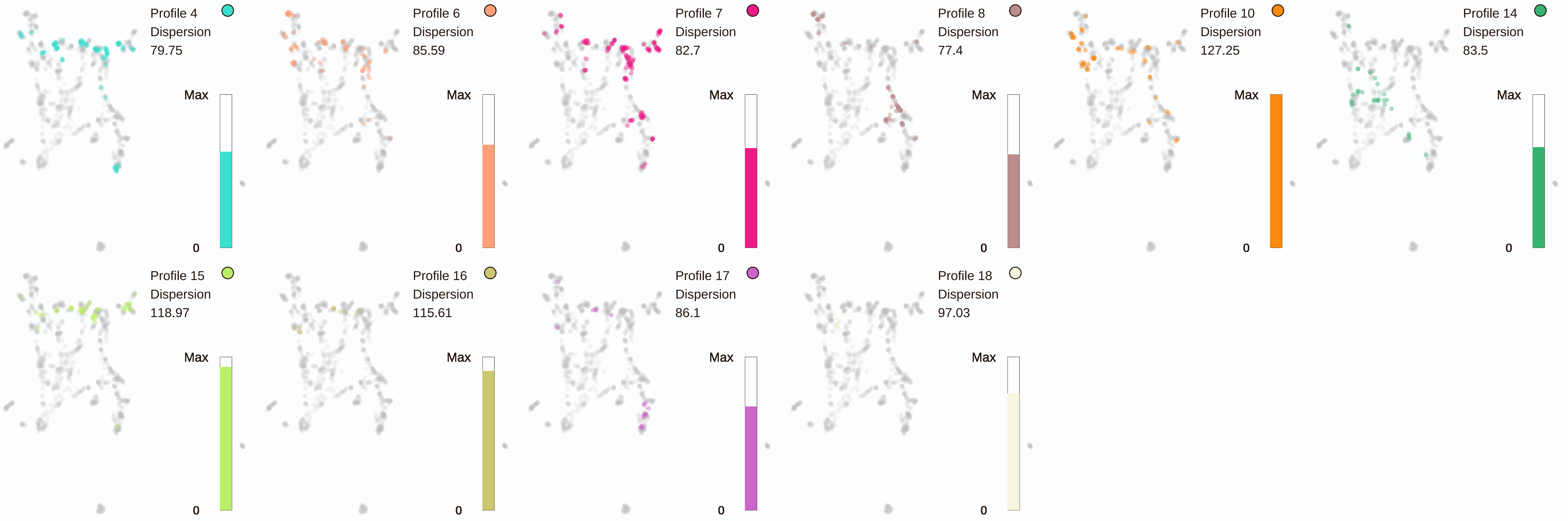

Tgf−beta family receptors (k_opt = 25)

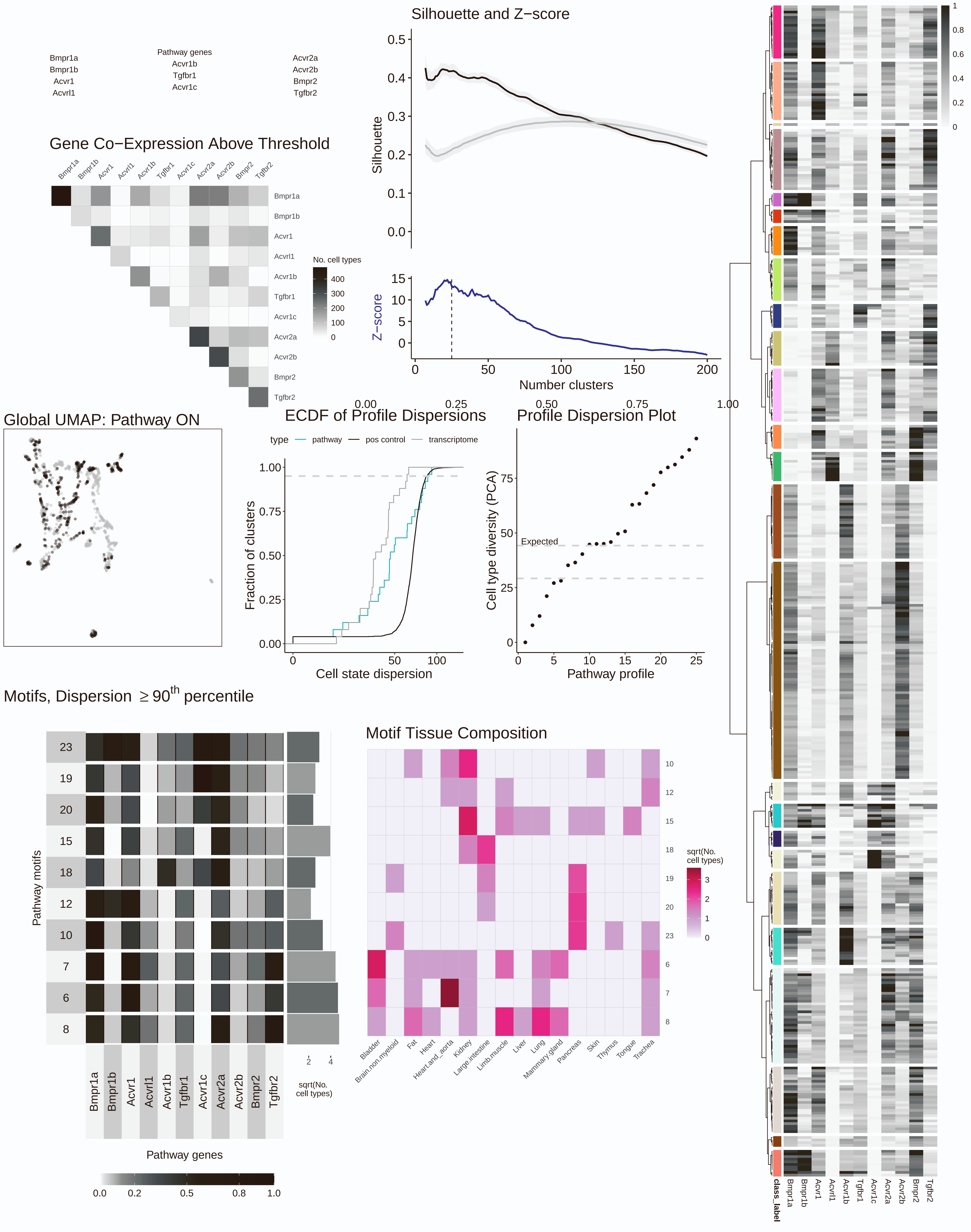

Tgf−beta family receptors (k_opt = 25)

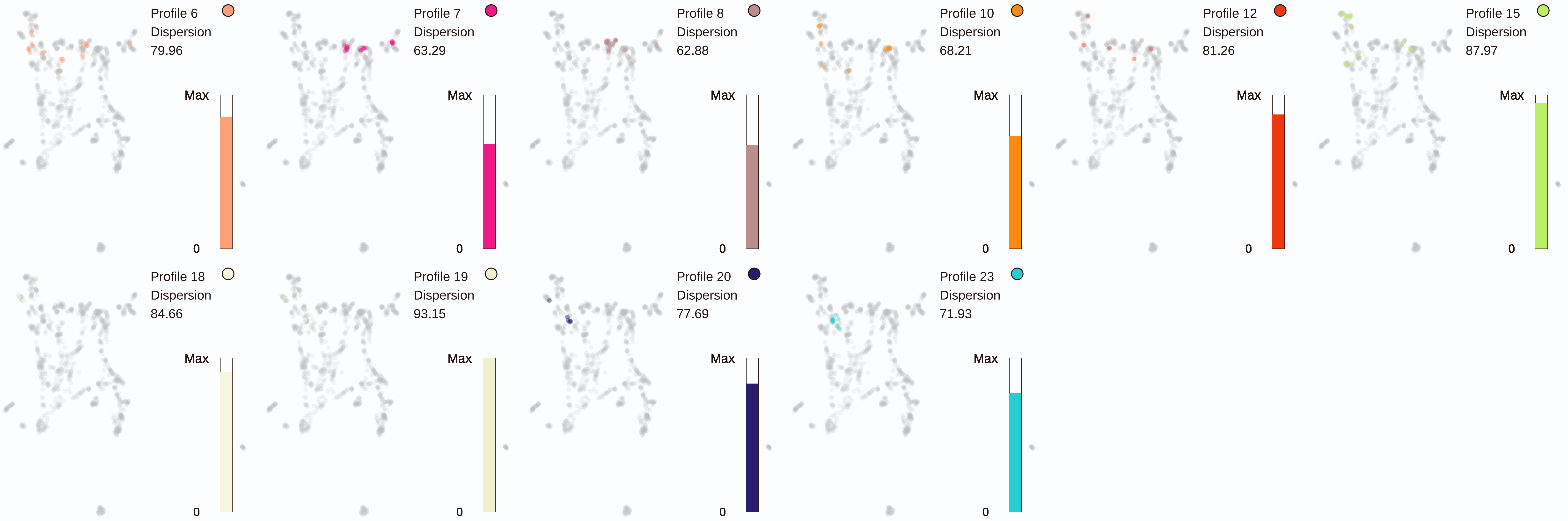

Supplement: Document S1. Figures S1–S6 and Data S1 and S2 [file mmc1.pdf]
