# Supplementary material for: Combinatorial expression motifs in signaling pathways
Source: Cell Genom. 2024 Jan 10;4(1):100463. doi: 10.1016/j.xgen.2023.100463 (PMC10794782; doi:10.1016/j.xgen.2023.100463)

Cell Genomics

Article

Combinatorial expression motifs in signaling pathways

Graphical abstract

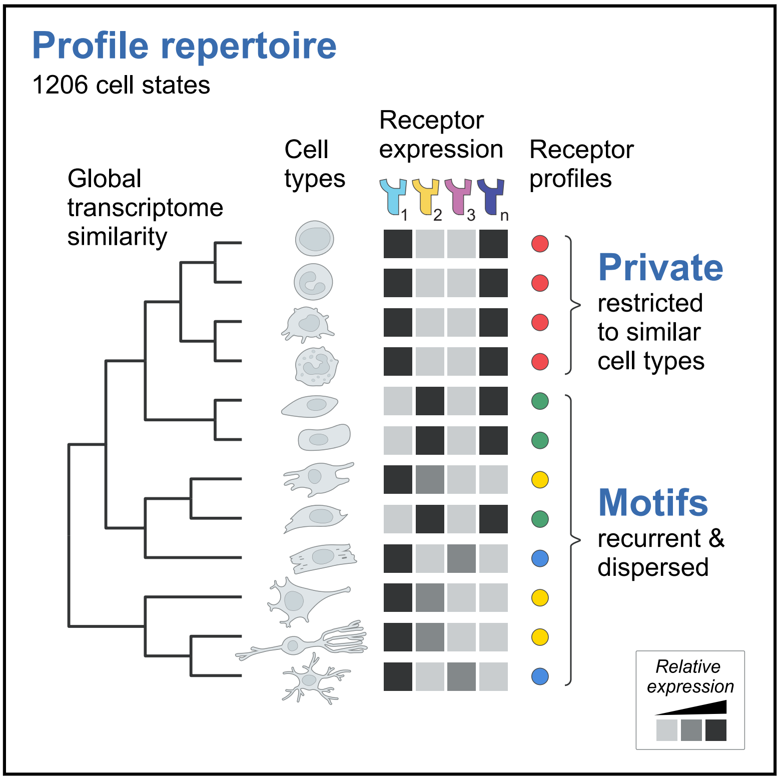

Highlights

- Core pathways exhibit combinatorial component expression profiles in atlas datasets
- Some profiles, termed motifs, recur across diverse organs and cell types
- Motifs reduce expression complexity and could represent distinct functional modes
- Motif usage is largely independent among pathways, suggesting a mosaic view of cell types

Authors

Alejandro A. Granados, Nivedita Kanrar, Michael B. Elowitz

Correspondence

melowitz@caltech.edu

In brief

Granados et al. identify pathway expression motifs from single-cell gene expression data, which are found across core cell signaling pathways. Different cell types were found to operate the same pathways but with distinct functional modes that can remain stable or change dynamically during biological processes.

Granados et al., 2024, Cell Genomics *4*, 100463
January 10, 2024 © 2023 The Authors.
https://doi.org/10.1016/j.xgen.2023.100463

CellPress

Cell Genomics

CellPress
OPEN ACCESS

Article

Combinatorial expression motifs in signaling pathways

Alejandro A. Granados,[1,2,3] Nivedita Kanrar,[1,2,4] and Michael B. Elowitz[1,2,5,*]
[1]Division of Biology and Biological Engineering, California Institute of Technology, Pasadena, CA 91125, USA
[2]Howard Hughes Medical Institute and Department of Applied Physics, California Institute of Technology, Pasadena, CA 91125, USA
[3]Present address: Chan-Zuckerberg Biohub, San Francisco, CA 94158, USA
[4]Present address: Department of Medicine and Life Sciences, Pompeu Fabra University, Barcelona, 08009 Catalonia, Spain
[5]Lead contact
*Correspondence: melowitz@caltech.edu
https://doi.org/10.1016/j.xgen.2023.100463

SUMMARY

In animal cells, molecular pathways often comprise families of variant components, such as ligands or receptors. These pathway components are differentially expressed by different cell types, potentially tailoring pathway function to cell context. However, it has remained unclear how pathway expression profiles are distributed across cell types and whether similar profiles can occur in dissimilar cell types. Here, using single-cell gene expression datasets, we identified pathway expression motifs, defined as recurrent expression profiles that are broadly distributed across diverse cell types. Motifs appeared in core pathways, including TGF-β, Notch, Wnt, and the SRSF splice factors, and involved combinatorial co-expression of multiple components. Motif usage was weakly correlated between pathways in adult cell types and during dynamic developmental transitions. Together, these results suggest a mosaic view of cell type organization, in which different cell types operate many of the same pathways in distinct modes.

INTRODUCTION

In metazoans, a handful of core cell-cell communication pathways such as TGF-β, Notch, Eph-ephrin, and Wnt play critical roles in diverse developmental and physiological processes.[1–4] Each of these pathways includes multiple, partly redundant, receptor variants that are expressed in distinct combinations in different cell types and interact in a many-to-many, or promiscuous, manner with corresponding sets of ligand variants (Figure 1A).[5–10] Within a given cell, the function of the pathway—which ligands it responds to or which intracellular targets it activates—in general depends on which combination of components a cell expresses.[11]

For example, the TGF-β pathway, which plays pivotal roles in diverse developmental and physiological processes,[12] comprises seven type I and five type II receptor subunits that combine to form heterotetrameric receptors composed of two type I and two type II subunits.[13] Cell types with distinct receptor expression profiles preferentially respond to distinct combinations of BMP ligands,[14,15] suggesting that different receptor combinations could provide distinct ligand specificities. Similarly, in mice, the Wnt pathway comprises a set of 10 Frizzled receptor variants that interact with two different LRP co-receptors, all of which are expressed in different combinations, and collectively control the cell's response to combinations of Wnt ligand variants.[16–18] The theme continues in the juxtacrine Notch and Eph-ephrin pathways where different membrane-bound ligand and receptor variants are expressed in diverse combinations and interact promiscuously to control which cells can signal to which others.[19–24] Similar families of gene variants are also found in non-signaling pathways as well. Despite the prevalence of these many-to-many architectures, it has generally remained unclear what expression profiles exist for a given pathway within an organism and how those profiles are distributed across cell types and tissues.

In principle, pathway expression profiles could be distributed across cell types in three qualitatively different ways. At one extreme, each cell type could express its own, completely unique, profile of pathway components (Figure 1B, left). In this case, one would observe as many distinct pathway profiles as cell types. Alternatively, sets of closely related (transcriptionally similar) cell types could share the same pathway expression profile (Figure 1B, center). This would result in fewer pathway profiles than cell types and a correlation between the similarity of pathway profiles and the similarity of the overall transcriptomes of the cells in which they appear. Finally, a limited number of recurrent pathway profiles could exist (as in the second case) but with individual profiles dispersed across multiple, distantly related cell types, rather than confined to sets of closely related cell types (Figure 1B, right). In this regime, otherwise similar cell types could exhibit divergent profiles for the pathway of interest, while, conversely, more distantly related cell types would converge on similar pathway profiles. In this regime, a limited repertoire of profiles, which we term "pathway expression motifs," are re-used in diverse cell contexts. Assuming that differences in pathway profile confer corresponding differences in

This is an open access article under the CC BY license (http://creativecommons.org/licenses/by/4.0/).

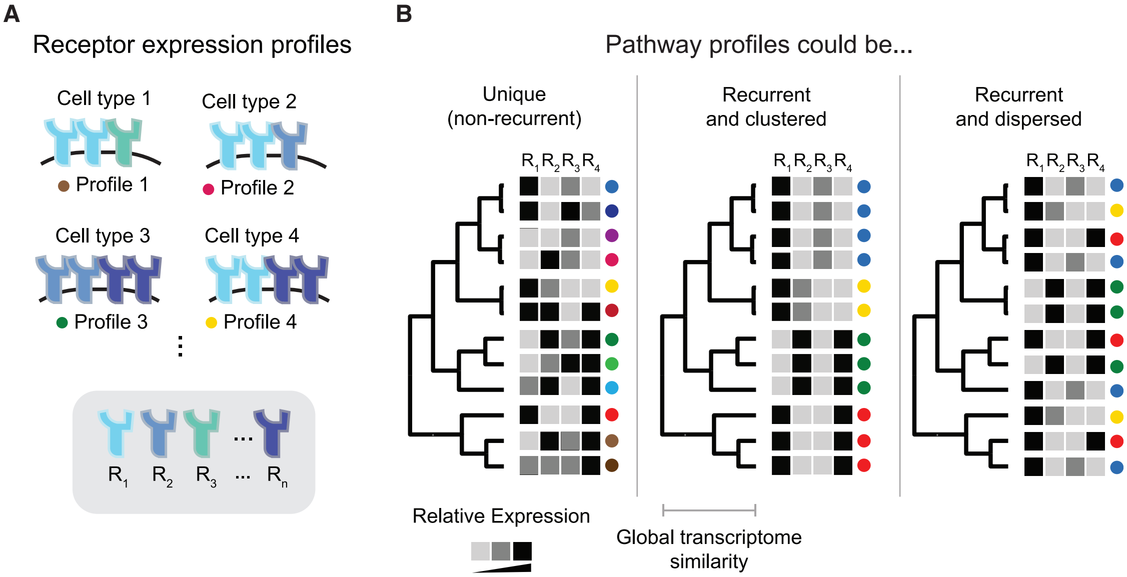

Figure 1. Pathway expression profiles could be distributed across cell types in different ways (schematic)
(A) Cell-cell signaling pathways comprise multiple variants of key components such as receptors (cartoons, R_n). These variants can be expressed in different combinations in different cell types. Colored dots identify receptor profiles for comparison with (B).
(B) Cell types can be arranged hierarchically based on similarities among their global (genome-wide) gene expression profiles (dendrogram). A hypothetical signaling pathway profile for each cell type is indicated by the gray intensity in the corresponding row of squares. In principle, each cell type could have a unique signaling pathway profile (unique, left); exhibit a smaller set of recurrent profiles, each used by a set of related cell types (recurrent and clustered, middle); or exhibit signaling pathway profiles that recur even among otherwise distantly related cell types (recurrent and dispersed, right). These possibilities are not exclusive, and it is possible that some pathways or subsets of cell types might operate in different regimes.

ligand responsiveness or other properties, each of these regimes implies something different about the number and distribution of functionally distinct signaling modes for a pathway of interest.

Previously, a lack of data precluded researchers from systematically distinguishing among these three behavior classes. Recently, however, single-cell RNA sequencing (scRNA-seq) cell atlases have begun to provide comprehensive gene expression profiles across most or all cell types in embryos and adult organisms. For example, one of the first efforts, the Tabula Muris project, provided expression profiles for ∼100,000 cells across 20 organs in adult mice.[25] This dataset was later augmented with studies of mice at additional ages.[26] In parallel, scRNA-seq studies of embryonic development have similarly provided transcriptional profiles for the cell states in the early embryo[27] and specific organs later in organogenesis.[28] Collectively, these data provide an opportunity to determine the combinatorial expression structure of many individual pathways.

Here, we introduce a statistical framework to identify pathway expression profiles and characterize their distribution across cell types in an aggregated dataset spanning multiple atlases. This approach allowed us to identify the pathway expression motifs described above (Figure 1B, right) as well as "private" profiles that are limited to sets of closely related cell types (Figure 1B, middle) in core communication pathways including TGF-β, Notch, and Wnt, as well as other pathways, such as the SRSF (serine/arginine-rich splicing factor) family of splice regulators. This analysis revealed that each pathway can operate in a handful of distinct "modes." Further, the mode used by one pathway appears to be independent of those used by other signaling pathways. Dynamically, pathway modes can remain remarkably stable or change suddenly as cells progressively differentiate during development. Together, these results provide a combinatorial view of signaling pathway states and suggest that many of the most central pathways can exist in a handful of different modes, which, in the future, may be studied independently of the cell types in which they appear.

RESULTS

Integration of cell atlas datasets

To analyze pathway expression profiles across a broad diversity of cell types, we first compiled data from multiple adult and developmental cell atlas datasets (Figure 2A; Table S1). These included the 10x 3' Tabula Muris cell atlas,[25] which comprises 45,000 cells distributed across 18 organs from a 3-month-old mouse, as well as 10x 3' Tabula Senis,[26] which augmented these data with ∼200,000 additional cells from mice aged 1, 18, 21, 24, and 30 months. We also included three early developmental whole-embryo atlases from embryonic day (E)5.5 and E6.5 to E8.5[27,29,30] as well as two organogenesis datasets, a whole-embryo[31] and a forelimb atlas,[28] that together span developmental days E9.5 to E15. Each of these datasets contained a cell type annotation for each cell based on expression of known markers. Altogether, the aggregated dataset included expression profiles and cell type annotations for ∼700,000 individual cells.

To allow a unified analysis of these data, we clustered the global transcriptional profiles from each dataset independently. This procedure resulted in 1,206 clusters, spanning 917 unique cell type

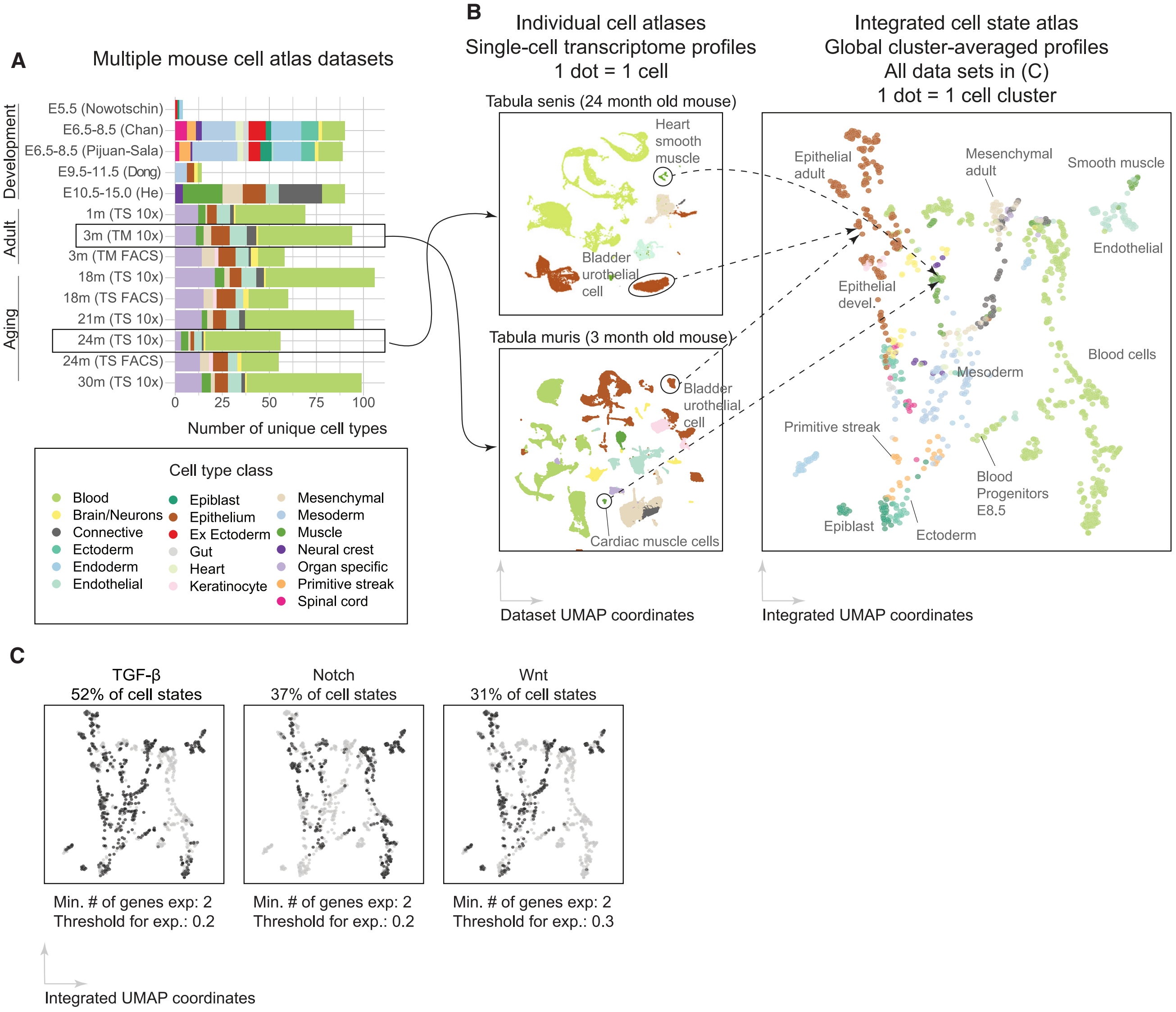

Figure 2. Integration of scRNA-seq atlas data reveals widespread expression of signaling pathway components

(A) We integrated seven published developmental and adult scRNA-seq datasets spanning 14 different stages in the mouse lifespan from embryonic development to old age. These datasets differ in their representation of organs and cell type classes (colors). The name of the dataset indicates the developmental stage and the first author's name. For the Tabula Muris and Tabula Muris Senis datasets, we used the abbreviations TM and TS, accordingly.

(B) To generate an integrated cell state atlas, we first independently clustered each scRNA-seq dataset, treating distinct time points in each dataset separately (UMAPs, left; STAR Methods). We then averaged expression over all cells in each cluster to yield a "cell state" profile for that cluster, and we represented each cluster by a single dot in an integrated cell state atlas dataset (UMAP, right). Colors are consistent with the legend in (A).

(C) Components of core signaling pathways are broadly expressed. Black or gray dots show clusters whose pathway components are expressed above or below threshold, respectively.

annotations (e.g., "Organ: Lung, cell type:endothelial, age: 3m"), providing a unified dataset for further analysis (Figure 2B, STAR Methods). For simplicity, in this work, we will refer to each global gene expression cluster as a "cell state" and not distinguish between formal "cell types" and other levels of variation. This clustering procedure and the cell states recovered from each dataset matched previous published analyses (Figure S1A).

To focus on expression differences between cell states, to reduce the complexity of the dataset, and to minimize the impact of measurement noise, we computed the average transcriptome profile of each one of the 1,206 clusters (STAR Methods), similar to other recent integration approaches.[32] A uniform manifold approximation and projection (UMAP) displays the variety of cell classes comprising the integrated atlas (Figure 2B, right). We note that cluster averaging potentially eliminates biologically meaningful gene expression variability within a cluster. However, pairs of genes that were highly expressed within a cluster average also showed significant co-expression in single cells

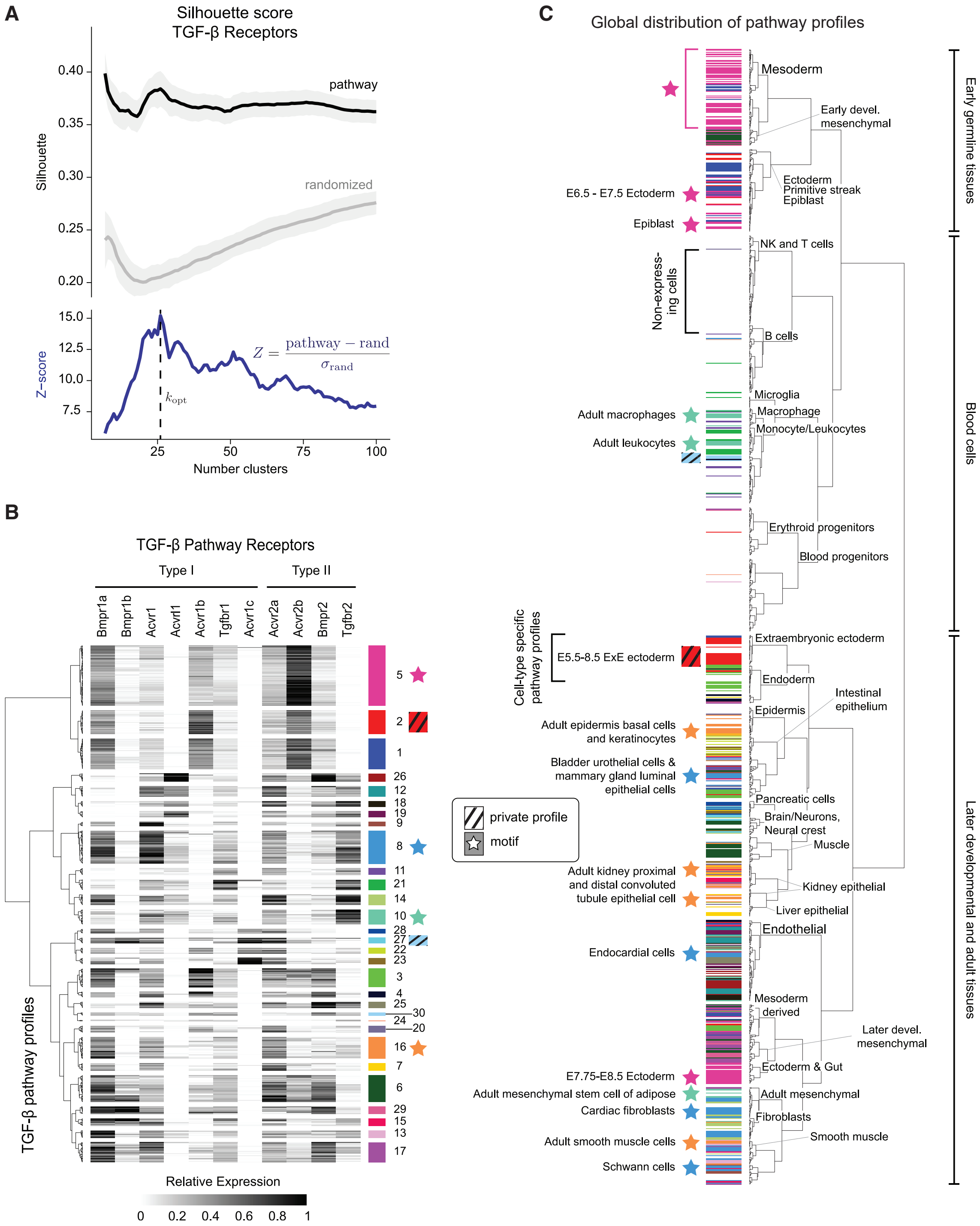

Figure 3. TGF-β receptors exhibit recurrent and dispersed pathway expression profiles

(A) The silhouette score identifies the approximate number of unique TGF-β receptor expression profiles. We computed the silhouette score across expression values of the pathway genes (black), as well as for 100 random gene sets (gray) where pathway gene expression was independently scrambled for each gene. We then computed the *Z* score (blue), defined as the silhouette score for pathway genes normalized to the silhouette score for randomized gene sets. We defined the optimal number of receptor profiles k_{opt} as the number of clusters that produced the peak *Z* score value, in this case, approximately 30 TGF-β receptor expression profiles (dashed line).

(B) Heatmap indicates gene expression of TGF-β receptor components in the 622 cell types expressing the pathway (Figure 2C). The identified ∼30 TGF-β receptor expression profiles are indicated as color-labeled groups of rows. Colored stars indicate examples of dispersed profiles highlighted on the global cell

(legend continued on next page)

($p < 0.001$, ** heatmap entries; Figure S1C). The integrated, cluster-averaged dataset provides a basis for analyzing systematic changes in pathway gene expression between cell states in embryonic and adult contexts.

TGF-β receptors exhibit recurrent expression profiles

TGF-β is among the most important and pleiotropic pathways in multicellular organisms, making it an ideal target for motif analysis.[33] A functional TGF-β pathway requires expression of at least one type I and one type II receptor subunit. Across the 1,206 cell states, approximately half met this criterion, expressing at least one receptor of each type above a minimum threshold (Figure 2C, STAR Methods). This criterion excluded some cell types—notably blood and immune cells—that functionally respond to TGF-β signaling but exhibit lower mRNA expression levels for receptors compared to most cell types (Figure S2A). Among the cell types that passed the filter criterion, the most prevalent receptors, Bmpr1a and Acvr2a, were expressed in ~10 times more cell types than the least prevalent, Acvr1c and Bmpr1b (Figure S2B). Nearly every receptor subunit was co-expressed with each other receptor subunit in at least some cell types (Figure S2D). Even Acvrl1 and Bmpr1a, which were mainly expressed in endothelial and epithelial cells, respectively, were also co-expressed in mesenchymal cells (Table S2). Exceptions included Bmpr1b and Acvr1c, which were less prevalent overall and were co-expressed with a more limited set of other subunits (Figure S2D). Overall, these results provided TGF-β transcriptional expression profiles across cell types and revealed that they were strongly combinatorial.

To test whether certain receptor profiles recurred across cell types (Figure 1B, middle and right panels), we clustered cell states based only on their TGF-β pathway expression profiles. To detect recurrent profiles, we computed the silhouette score, which compares the separation of points between clusters to the proximity of points within a cluster and penalizes for both over- and under-clustering (Figure S2E).[34] The silhouette score provides a metric to quantify the approximate number of distinct clusters in a dataset. However, any set of random variables can be clustered. We therefore compared the silhouette scores of the real data (Figure 3A, black line) to those obtained from randomized control datasets. More specifically, we constructed a set of randomized datasets, in each of which we randomly permuted the expression levels of each gene across all cell types, maintaining the distribution of expression values for each gene while eliminating gene-gene correlations. We note that this procedure exactly preserves the quantitative distributions of each gene and therefore is preferred over an indiscriminate randomization over the whole matrix. We then performed silhouette analysis on each randomized dataset to obtain a null distribution of silhouette scores (Figure 3A, gray lines). Finally, we computed a silhouette Z score that compares the silhouette score of the real data against the distribution of scores obtained from randomized datasets (Figure 3A, blue line). Using this Z score, we selected an optimal number of clusters, k_{opt}, defined as the largest value of k that reached at least 90% of the maximum Z score (Figure 3A, dashed line; STAR Methods). To normalize for the number of genes in the pathway, we also defined a recurrence score for the pathway as $r = k_{opt}/N_g$, where N_g denotes the number of genes included in the pathway definition. Altogether, this analysis revealed that 622 cell states expressing TGF-β receptors collectively exhibit only about ~30 distinct, recurrent pathway expression profiles, generating a recurrence score of $r = 30/11 = 2.7$ (Figure 3B). Critically, every receptor subunit was expressed in at least one of these profiles, consistent with a combinatorial view of receptor utilization.

TGF-β pathway expression motifs appeared in diverse cell types

Having identified recurrent pathway expression profiles, we next asked how they were distributed across cell types, as in Figure 1B. To answer this question, we first visualized TGF-β pathway expression profiles on the dendrogram of global cell types (Figure 3C; Data S1). We color-coded each profile in Figure 3B and then annotated each cell state on the global dendrogram with the color corresponding to its TGF-β profile (Figure 3C). Strikingly, many profiles were broadly distributed over diverse cell types (Figure 3C, colored stars). For example, profile 10 (mint green) appeared in adult macrophages and leukocytes as well as mesenchymal adipose stem cells. On the other hand, a smaller number of pathway profiles showed the opposite behavior. They were restricted exclusively to a particular clade of closely related cell states (Figure 3C, colored shaded boxes). These results suggest that TGF-β could exhibit both pathway motifs and private profiles.

To more systematically and quantitatively characterize the distribution of each pathway profile across cell types, we defined the "dispersion" of a given TGF-β profile as the mean value of the pairwise Euclidean transcriptome distances among all cell types that express that profile, computed in the space of the 100 most significant principal components (Figure 4A). We compared each profile's dispersion to two hypothetical limiting cases: a low dispersion limit, in which individual profiles were restricted to sets of closely related cell types (Figure 4C, gray line), and a high dispersion limit, in which the profile was randomly assigned to different cell types (Figure 4C, black line). For the lower limit, we applied a threshold on the dendrogram of global transcriptome similarity (Figure 3C) to obtain the same number of clusters as the pathway. We then computed the distribution of mean pairwise distances in principal component space among the cluster-averaged global expression profiles (Figure 4C, gray line). For the upper limit, we randomly reassigned pathway profiles to cell types and then computed the resulting dispersion distribution (Figure 4C, black line).

fate dendrogram in (C). Colored shaded boxes indicate private profiles, also shown in (C). Dendrogram at left represents similarity among different profiles. Each gene is standardized to a range of 0–1 across all cell types (grayscale) as described in the STAR Methods.

(C) Distribution of TGF-β receptor expression profiles across cell types. The global cell type dendrogram was computed using a cosine distance metric applied to the integrated transcriptome dataset in a 20-component principal-component analysis (PCA) space constructed from 4,000 highly variable genes (HVGs). Stars indicate featured TGF-β profiles that are broadly dispersed across cell types, while colored shaded boxes indicate examples of private profiles. Cell types that do not express TGF-β receptors have no color (white). Colors match those in (B). Note that blood cell types are relatively lacking in expression of TGF-β receptors.

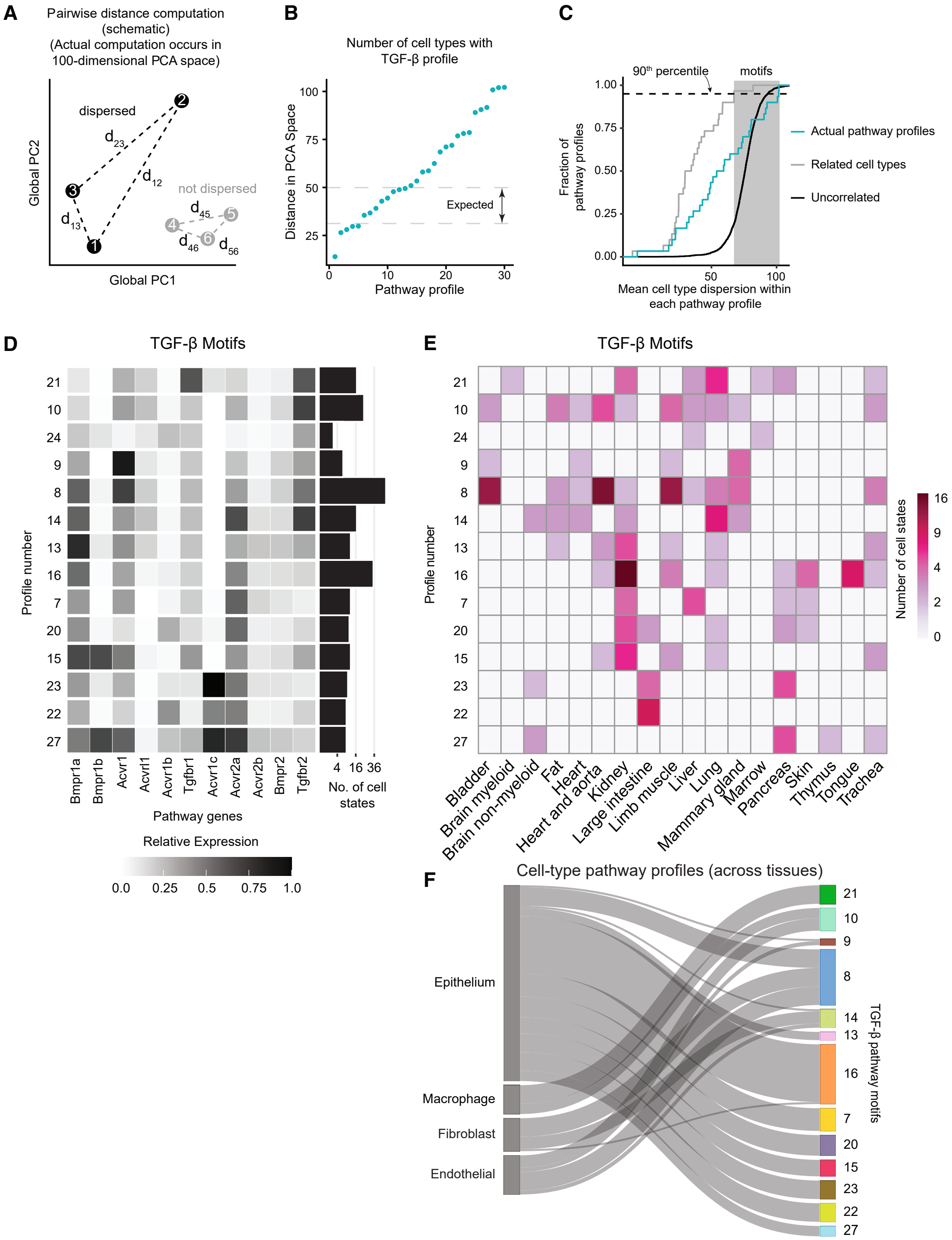

Figure 4. TGF-β expression motifs are dispersed across cell types and organs

(A) We defined the dispersion of a receptor expression profile to be the within-class pairwise distance computed in a 100-dimensional PCA space constructed from the top 4,000 highly variable genes (HVGs) (left). Dispersed profiles (black) show high cell type diversity, whereas non-dispersed profiles (gray) are closer together in PCA space.

(B) The dispersion of actual TGF-β expression profiles. Dashed lines indicate the expected range (25th and 75th percentiles, respectively) of dispersions obtained by clustering similar cell types using the whole transcriptome. Note the large number of profiles with larger dispersions than expected in similar cell types.

(legend continued on next page)

This analysis revealed that many profiles were broadly dispersed. ∼44% of TGF-β profiles were predominantly observed in specific sets of closely related cell types (Figure 4B, points below the lower-limit distribution's 75th percentile, top dashed line). By contrast, 56% of TGF-β profiles were dispersed more broadly, often spanning distantly related cell types (Figure 4B, points above the 75th percentile). In fact, among this subset of TGF-β profiles, dispersion levels approached those produced by random reassignment (Figure 4C, turquois line and shaded region). For example, profile 16 was observed in cardiomyocytes, kidney podocytes, and keratinocytes from the tongue. Similarly, profile 8 was observed in bladder endothelial cells, type II pneumocytes, and epithelial cells from the mammary gland. Based on this analysis, we defined pathway expression motifs as profiles whose mean cell type dispersion exceeded the 90th percentile of the lower bound distribution (Figure 4C; STAR Methods).

We note that this definition of motifs is sensitive to an arbitrary percentile cutoff and could be biased by over-representation of cell types due to integration of overlapping datasets or under-representation due to missing cell types. Further, alternative dispersion metrics could be used, although these produced broadly similar motif sets (Figure S3A).

TGF-β pathway motifs exhibited several interesting features. First, they were enriched for expression of the type I receptors Bmpr1a and Acvr1, as well as the type II receptor Acvr2a. In fact, almost all motifs co-expressed all three of these receptor subunits (Figure 4D). On the other hand, Bmpr1b, Acvrl1, and Acvr1c were the least represented receptor subunits, appearing in only three, three, or four of the motifs, respectively. The most prevalent motif, 8, was expressed in nine different mouse organs and is similar to the profile of NMuMG mammary epithelial cells, which were shown to compute complex responses to ligand combinations[15,35] (Figure 4D, rows). Motif 8 included the type 1 subunits Bmpr1a, Acvr1, and Tgfbr1, as well as the type II subunits Acvr2a, and Tgfbr2. Motif 15, which is similar to motif 8 but with more Bmpr1b, was shown to exhibit reduced complexity of combinatorial ligand responsiveness,[35] suggesting that even a change in a single receptor between profiles could be functionally significant.

We also examined expression correlations among individual TGF-β receptors. Among cell states expressing pathway motifs, almost half of the receptor pairs (25/55) showed no significant correlation, with the remaining pairs exhibiting a mix of positive and negative pairwise correlations (Figure S3B, middle). For example, Bmpr1a was positively correlated with Acvr1 and Acvr2a, while Acvrl1 and Tgfbr2 were strongly correlated, with Acvrl1 expressed in a subset of cell types that expressed Tgfbr2. Acvrl1 and Tgfbr2, which were previously shown to mediate signaling by BMP9, could also function together as a module in this context.[36]

Motifs were broadly distributed across the organism, with some appearing in as many as 10 different mouse organs (Figure 4E, rows). Conversely, multiple motifs appeared in the same organ. For example, the adult kidney included cell states with nine different TGF-β receptor expression motifs (Figure 4E, columns). These results underscore the breadth of the dispersion of the pathway motifs.

Cell types can be grouped into more general, higher-level classes such as macrophages, fibroblasts, epithelial, or endothelial cells, each of which comprises a diverse set of cell types across multiple organs. In principle, a motif could be restricted to a single cell type class and still be dispersed across transcriptome states. Alternatively, it could recur in multiple cell type classes. To gain insight into the distribution of motifs across cell type classes, we tabulated the distribution of TGF-β profiles across cell type classes, based on cell type annotations in the atlas (Figure S3C). We then constructed a Sankey diagram to visualize the relationship between motifs and cell type classes (Figure 4F). Each cell type class included multiple motifs, with different degrees of diversity, ranging from just two motifs for macrophages to 11 different motifs for epithelial cells. Macrophages included just two motifs, 10 and 21. A set of seven motifs each appeared only among epithelial cells, while motif 21 was similarly restricted only to macrophages. The remaining five motifs each appeared in at least two different cell type classes. (We note that motif 24 did not appear in any cell types annotated for one of these four classes.) These results suggest that TGF-β receptors motifs show partial but not complete preferences for certain cell type classes.

In contrast to motifs, which were by definition dispersed, other TGF-β profiles recurred in multiple cell types but exhibited low dispersion, as in Figure 1B, middle panel (Figure S3D). One of these groups, consisting of profiles 1, 2, and 5, was in fact dispersed among diverse developmental cell types, including the primitive streak, ectoderm derivatives, and mesodermal tissues. However, it received a lower dispersion score due to the relative similarity of early embryonic cell types compared to adult cell types. We therefore classified these profiles as a developmental motif (Figure 3B, hot pink). These three profiles expressed a combination of Bmpr1a and Acvr2b and resembled the BMP receptor profile previously identified in mouse embryonic stem cells, suggesting that the early embryonic receptor profile is stably maintained during early germ layer cell fate diversification.[35]

(C) Empirical cumulative distribution functions of TGF-β profile dispersion. The observed dispersion distribution (turquoise) lies between the extremes of cell-type-specific profiles (gray) and profiles obtained by randomizing cell type distances by shuffling cell type labels (black). We classified motifs in the shaded region, defined as being in at least the 90th percentile of the related cell type dispersion distribution (gray) as motifs.

(D) We identified 14 TGF-β motifs, arranged by gene expression similarity. For each motif, the number of cell states in which it appears is indicated by the histogram at right.

(E) TGF-β motifs (rows) are broadly distributed across different tissues and organs (columns). Each matrix element represents the number of cell states in the indicated tissue or organ expressing the corresponding motif. Note that most motifs are expressed in multiple tissues or organs, and most tissues or organs contain multiple motifs.

(F) Key cell type classes, including epithelial, macrophage, fibroblast, and endothelial cell types, each span multiple TGF-β motifs. Motifs are ordered to match the ordering in (D) and (E). Each cell class mapped to multiple distinct pathway motifs yet differed in their motif diversity. For example, epithelial cells comprise a broad spectrum of 11 distinct motifs, whereas macrophages and endothelial cells are primarily restricted to smaller subsets of more closely related motifs.

By contrast, profiles 29 and 30 were each confined to a single set of closely related cell types: chondrocytes (E13.5–E15.0) and macrophages, respectively (Figure 3B; Table S2). Because they were tightly associated with a particular set of cell types, these profiles are effectively the opposite of a motif, and we refer to them as "private," or cell-type-specific, profiles. Notably, these private profiles both expressed Bmpr2, which is less prevalent compared to other receptors. Nevertheless, Bmpr2 is not an exclusive marker of private profiles, as it is also expressed in dispersed motifs, such as motifs 8, 9, 10, 13, and 27 (Figure 4D). Together, these results suggest that the TGF-β pathway exhibits a set of recurrent and dispersed expression motifs, as well as a smaller number of private profiles.

Additional signaling pathways also exhibit pathway expression motifs

Pathway motifs could in principle occur in many other pathways. To assess how general this type of organization is, we used the PathBank database of biological pathways[37] to identify 40 different annotated biological pathways involved in signaling and other functions (Table S3). For each pathway, we assembled a corresponding list of genes, normalized their expression, clustered the resulting profiles, computed silhouette scores, and compared them to a null hypothesis in which the expression levels of each gene were independently and randomly reassigned to different cell types as described previously (Figure S4A). Pathways differed in the width of their silhouette profiles. For example, the SRSF splicing protein family exhibited a narrow silhouette peak similar to that of TGF-β receptors, indicating a well-defined number of distinct profiles (SRSF, Figure 5A, upper panel; TGF-β, Figure 3A; Figure S4A, left column). By contrast, other pathways, such as the Ras signaling pathway, exhibited a broad silhouette profile, with no clear optimum (Figure 5A, lower panel; Figure S4A, right column). Across all pathways surveyed, we observed a bimodal distribution of silhouette profile widths (Figure 5B).

To identify pathways with strong motif structure, we then computed the dispersion score for pathways with well-defined silhouette peaks (STAR Methods). Finally, to visualize the two key motif metrics together, we plotted dispersion versus recurrence score for these pathways (Figure 5C). Among the pathways with the strongest motif signatures (Figure 5C, upper-left corner), we observed core cell-cell communication pathways such as Notch, Wnt, and ephrin; the SRSF splicing protein family, which includes all 11 SR family splice regulatory proteins; and a protein degradation pathway defined by PathBank, consisting predominantly of different proteasome subunits.[37]

Notably, not all pathways exhibited strong motif signatures. In fact, some pathways displayed recurrent but weakly dispersed profiles that were more confined to related cell types (Figure 5C), similar to Figure 1B, middle. These pathways included NF-kB signaling, Ras signaling, and lipopolysaccharide (LPS) signaling in inflammation (Figure 5C; Table S3).

Notch, SRSF, and Wnt pathways exhibit dispersed expression motifs

In addition to TGF-β, the developmental signaling pathways Notch, SRSF, and Wnt all also exhibited strong motif signatures (Figure 5C). We therefore analyzed their motifs in more detail.

Notch signaling

The Notch pathway involves juxtacrine interactions between a set of membrane anchored ligands, including Dll1, Dll4, Jag1, Jag2, and the *cis*-inhibitor Dll3, and a set of four Notch receptors, Notch1-4.[38–40] Further, a set of three Fringe proteins (M-, R-, and L-fng) modulates *cis* and *trans* ligand-receptor interaction strengths, both between adjacent cells (*trans*) as well as within the same cell (*cis*).[41,42] We therefore defined a minimal Notch pathway comprising 11 ligands, receptors, and Fringe proteins (Data S2). This definition excludes ADAM family metalloproteases, γ-secretase, the CSL (CBF1, Suppressor of Hairless, Lag-1) complex, and other components, in order to focus specifically on ligands, receptors, and the Fringe proteins that directly modulate their interactions, all of which exist in multiple variants. We classified pathway expression as "on" if at least two of these genes were expressed above a minimum threshold of 20% of the maximum observed expression level across all cell types. With these criteria, the Notch pathway was "on" in 37% of cell states (450 out of 1,200) (Figure 2C).

As with TGF-β, the Notch pathway exhibited combinations of co-expressed components, including receptors, ligands, and Fringe proteins. The pathway exhibited a peak silhouette score at ∼30 cell clusters (Data S2), 16 of which qualified as motifs based on their dispersion scores (Data S2; Figure 6A).

The Notch motifs were largely consistent with previous observations. For example, B cells (Notch motif 19) are known to express the Notch2 receptor and no ligands.[43,44] Among the three Delta ligands, it was notable that Dll3, which inhibits, but does not activate, Notch signaling, was strongly expressed only in motif 4, whereas expression of the activating ligands Dll1 and Dll4 was more widespread. This is consistent with previous observations of Dll3 expression in brain and bladder epithelial tissues, where motif 4 appears.[45]

Most motifs co-expressed both ligands and receptors. For example, the combination of Notch1, Notch2, and Jag1 occurred in most motifs, which were distinguished by expression of other components (Figure 6A). Nevertheless, even among motifs expressing both Notch1 and Notch2, the expression ratio of the two receptors varied (compare Notch motifs 19 and 28, Figure 6A).

Among the Fringe proteins, R-fng was expressed in all motifs (Figure 6A). While the expression distributions of the three Fringe proteins across cell types differed from one another, L-fng and M-fng both exhibited lower median expression levels compared to R-fng (Figure S5B). In particular, R-fng was broadly expressed at levels ≥0.25 on the normalized expression scale in most motifs, while less than half of the cell types exceeded this threshold for L-fng and R-fng (Figure S5B).

In addition to its expression motifs, Notch also exhibited a smaller set of "private" expression profiles limited to closely related cell types (Figure S5C). Private motifs were used by muscle cells during forelimb development (profile 25), basal cells of the mammary gland (profile 21), mesodermal lineages at E7.0–E8.0, and the adult endothelium (profile 8). The private profiles exhibited greater expression of M-fng and the Delta family ligands Dll1, -3, and -4 compared to the motifs (Data S2).

Taken together, these results reveal that the Notch pathway uses a set of recurrent and dispersed combinatorial expression

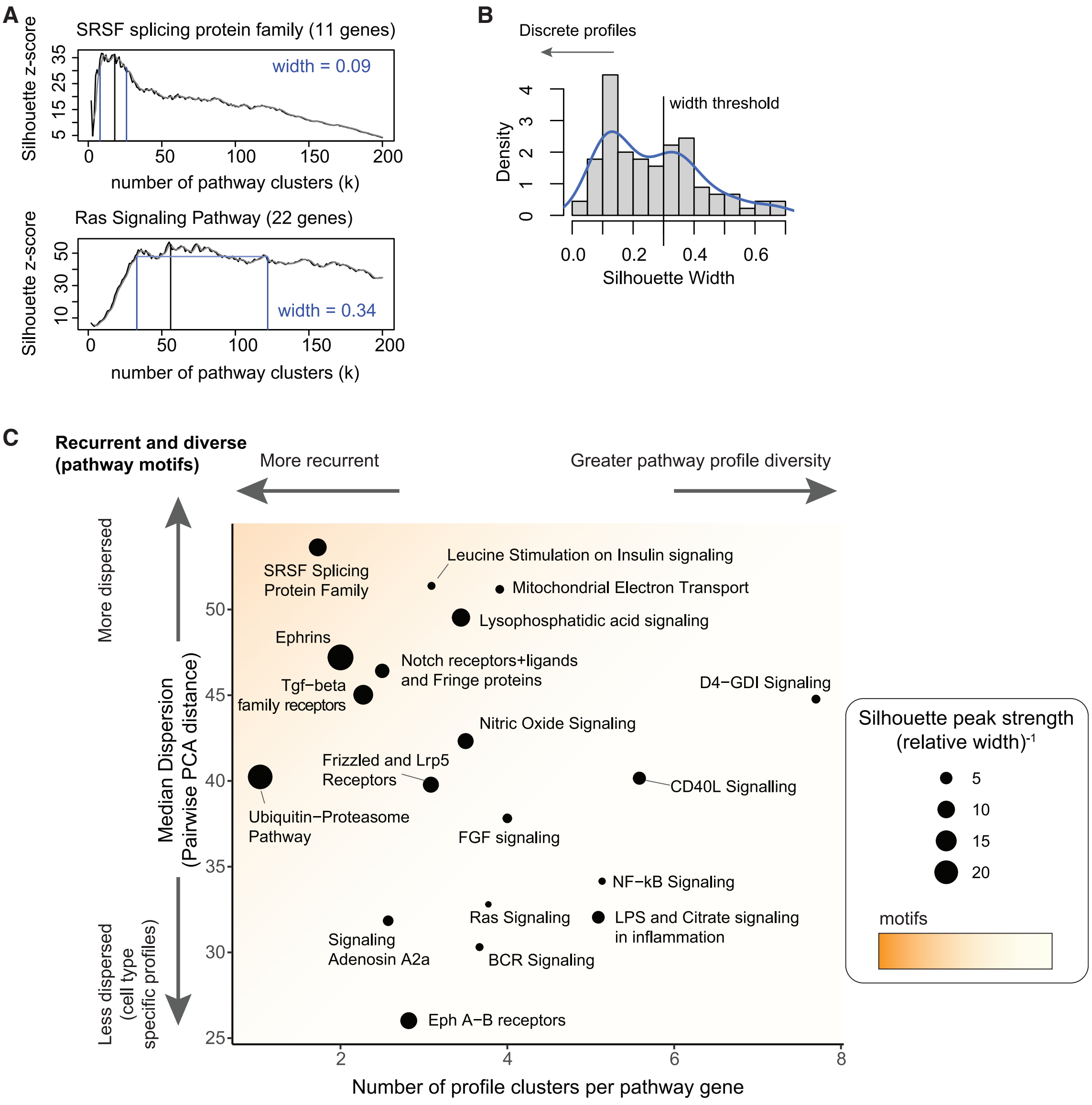

Figure 5. Expression motifs occur in multiple pathways

(A) The silhouette score calculated for different numbers of clusters (*k*) normalized as a *Z* score compared to randomized profiles. The peak width is defined as the number of *k* values with a *Z* score within 10% of the maximum *Z* score (blue lines), relative to the total number of *k* values evaluated (200). Pathways with a well-defined peak (SRSF, top) display a well-defined number of profiles around the peak. On the other hand, a broad range of *k* with high silhouette scores indicates higher order structure as for increasing number of clusters (Ras signaling pathway, bottom).

(B) The distribution of width scores for selected pathways (from PathBank database, Table S3).

(C) Dispersion and recurrence metrics for multiple pathways with well-defined peaks (relative width < 0.35). Based on the silhouette *Z* score, we identified the optimal number of clusters and computed the dispersion for different pathways (y axis). The optimal value of *k* is normalized by the number of genes in the pathway (x axis). We defined the silhouette peak strength as the inverse of the peak width (dot size). Pathways including motifs appear in the upper-left corner: they display a few discrete profiles that are expressed across multiple cell types.

motifs as well as private expression profiles in some lineages. Notch ligands and receptors are known to exhibit inhibitory (*cis*-inhibition[19,46]) and activating (*cis*-activation[47]) same-cell interactions that can generate complex interaction specificities with other cell types expressing similar or different ligand and receptor combinations. The prevalence of multi-component Notch motifs could help explain complex Notch behaviors with the potential to send or receive signals to or from specific cell types.[4,20,46]

SRSF splicing proteins

Among the most recurrent and dispersed pathways in our panel was the SRSF family of splicing regulators (Figure 5C, top left).

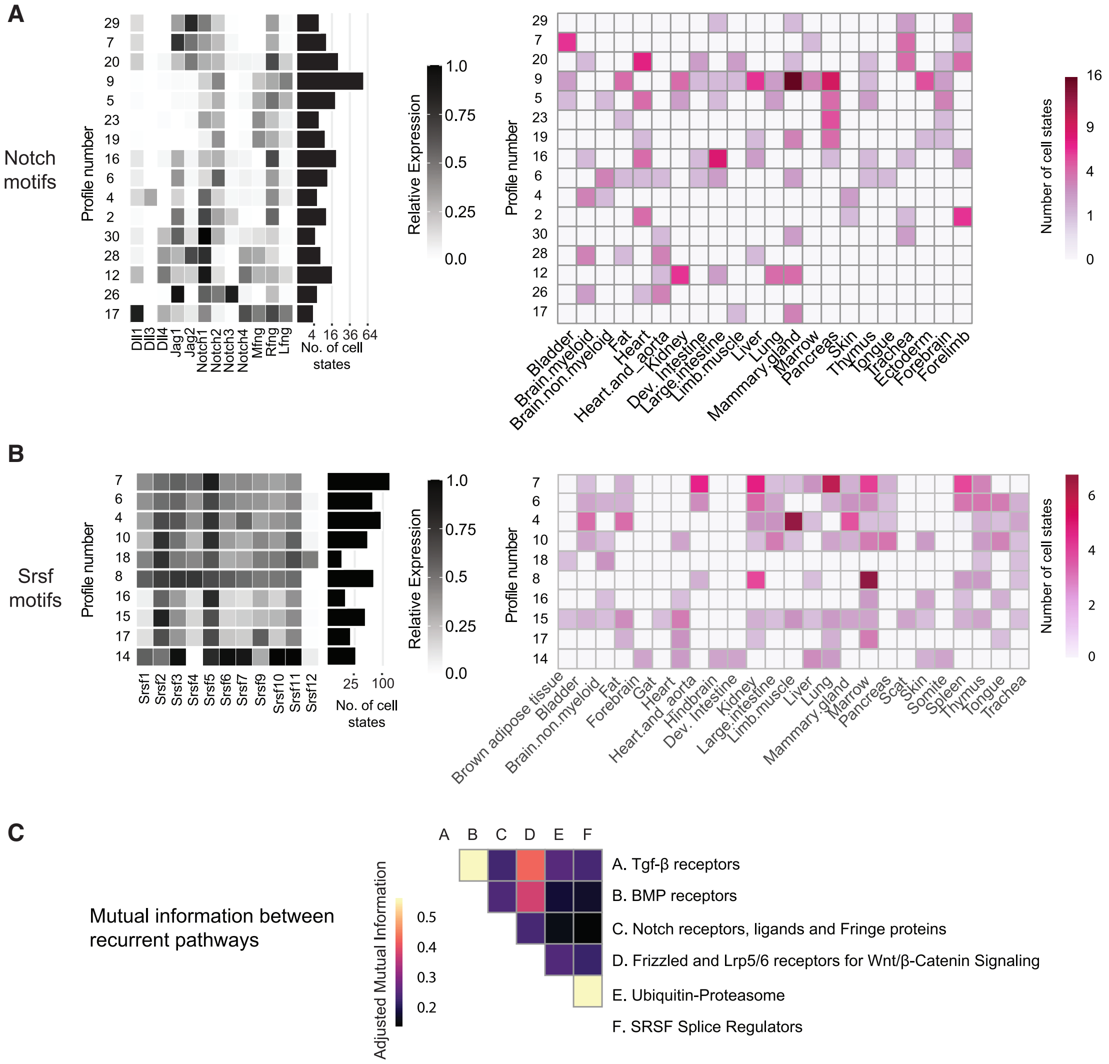

Figure 6. Expression motifs occur in multiple pathways and are largely uncorrelated

(A) Motifs in the Notch pathway and their distribution across tissues and organs, similar to Figures 4D and 4E.

(B) Motifs in the SRSF pathway and their distribution across tissues and organs, similar to Figures 4D and 4E.

(C) Pairwise correlations in profile usage between pathways, quantified by the adjusted mutual information between their respective profile labels.

SRSF proteins play crucial roles in alternative splicing, generating diversity in the transcriptome,[48] by modulating the recognition of exon-intron boundaries and interacting with other components of the spliceosome to promote the selection of specific splice sites.[49] Diverse SRSF proteins play partially overlapping but distinct roles in transcription-coupled splicing and mRNA processing in the nucleus,[50,51] and they have varying abilities to "shuttle" between nucleus and cytoplasm.[52] Some variants promote exon inclusion while others promote exon skipping,[49] with the balance of SRSF factors in the cell influencing the final composition of mature transcripts.

We tabulated the expression of the 11 SRSF mouse variants across all global clusters. The recurrence score for SRSF was lower (more recurrent) than that of TGF-β and Notch, and its silhouette score peaked at ∼19 clusters (Data S2). Strikingly, all of these clusters exhibited co-expression of multiple SRSF variants (Figure 6B). Some SRSF proteins were broadly expressed (SRSF2 and SRSF5) across all tissues, whereas others showed more motif-specific expression. For example, SRSF12 appeared predominantly in only a single motif. At the same time, that motif was distributed across multiple tissues, including thymus, trachea, brain, and fat (Figure 6B, right). More generally, all motifs were combinatorial, including multiple SRSF variants, and broadly distributed across tissues and organs (Figure 6B, right). Conversely, most tissue and organ types contained multiple SRSF motifs. Because of the high level of recurrence, the two largest motifs (numbers 4 and 7) were expressed in more than 100 distinct cell states (Figure 6B, left, histogram), with early developmental cell states exhibiting elevated SRSF expression levels overall (Data S2).

CellPress
OPEN ACCESS

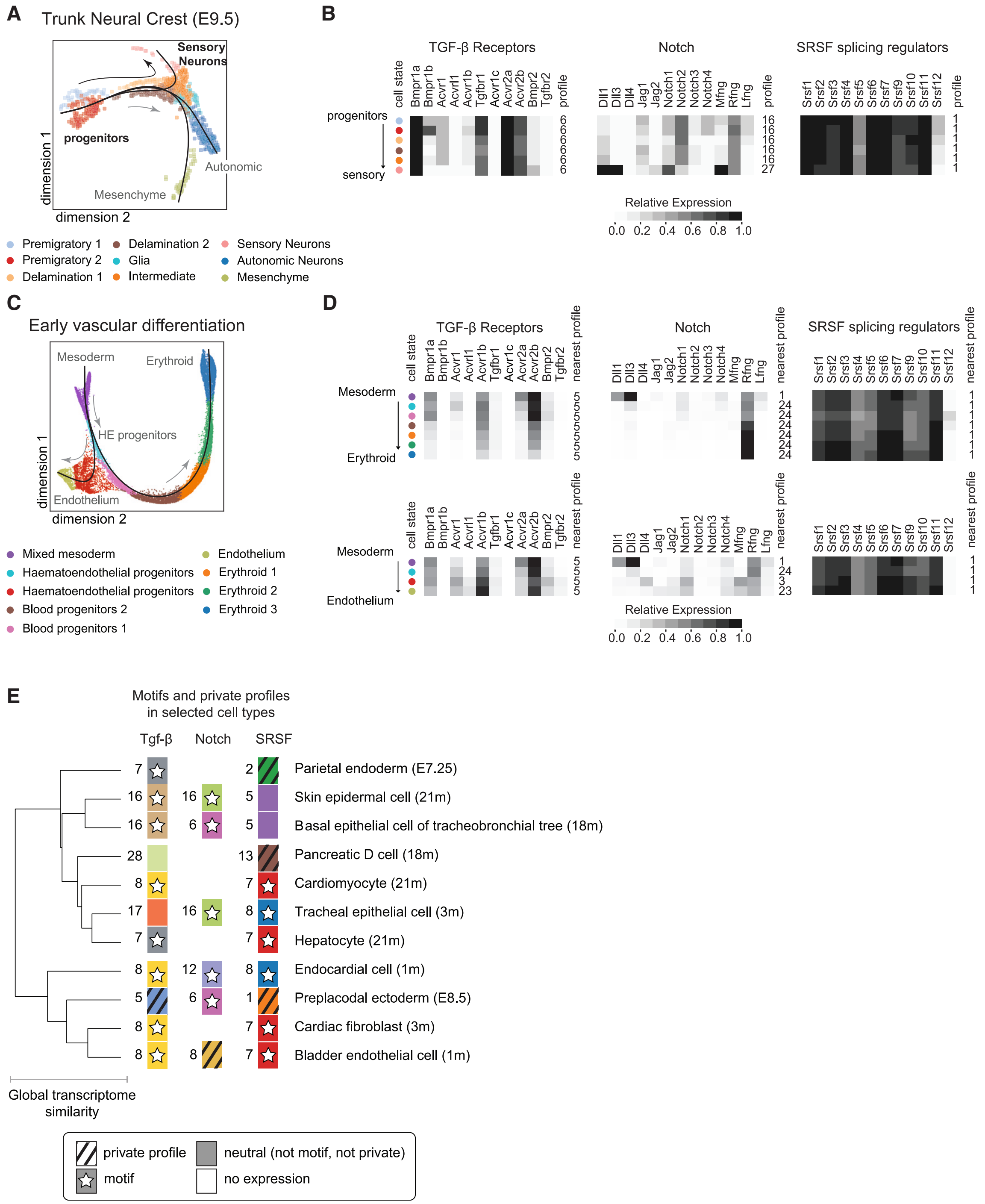

Figure 7. Developmental transitions of pathway profiles and summary

(A) Pseudotime trajectory analysis of the trunk neural crest[60] captures delamination of progenitors into three distinct cell fates in a ForceAtlas projection: sensory neurons, autonomic neurons, and the mesenchyme. Here, we follow the sensory neuron trajectory (black arrow).

(B) Developmental pathways expression dynamics in neural crest differentiation. For each pathway, corresponding mean expression profiles are shown in grayscale for each of the cell states indicated in (A), as indicated by the colored dots. Profile numbers indicate the closest match (nearest neighbor) to one of the reference pathway profiles in Figure 3B for TGF-β and Data S2 for Notch and SRSF.

(legend continued on next page)

Wnt signaling

Finally, as a fourth signaling pathway, we also analyzed Wnt, which plays critical roles in a vast range of developmental and physiological processes. Wnts can function as morphogens and are involved in regeneration, cancer, and disease.[53] Extracellular interactions between Wnt ligand and receptor variants exhibit promiscuity, with each ligand typically interacting with many receptor variants.[16] Signaling involves Wnt ligands binding to Frizzled (Fzd1-10) receptors and low-density lipoprotein-related co-receptors 5/6 (LRP5/6) to stabilize β-catenin, allowing it to activate transcription of target genes.[18,54,55] Wnt signaling has also been shown to have combinatorial features.[56]

The recurrence score for Wnt was slightly less than that of TGF-β and SRSF (Data S2). Nonetheless, the pathway exhibited recurrent profiles. Silhouette score analysis showed a peak elevation at $k_{opt} = 37$ profiles and was elevated compared to a null model of randomly scrambled pathways constructed from the same genes (Data S2). Strikingly, these profiles all exhibited co-expression of multiple Fzd variants, and all but two co-expressed both the Lrp5 and Lrp6 co-receptors (Figure S5A).

A subset of Wnt pathway expression profiles were broadly dispersed (Figure S5A). All of these high dispersion profiles co-expressed multiple Frizzled variants. Conversely, most Frizzled variants were expressed in multiple high dispersion profiles. The exceptions were Fzd9 and Fzd10, which were expressed at much lower levels in most cell types, although Fzd9 was highly expressed in profile 34, along with other receptors. These results show that the Wnt pathway also exhibits combinatorial expression motifs.

Inter-pathway correlations reveal independent profile usage

Identifying combinatorial expression profiles in multiple pathways provokes the question of whether component configurations are correlated between pathways. For example, in the limit of tight coordination, cells expressing one TGF-β profile might always express a corresponding Notch profile. In the opposite limit, profiles from one pathway might be used independently of those from another pathway, suggesting a more mosaic cellular organization.

To quantify the correlation between expression profiles of different pathways, we computed the pairwise adjusted mutual information (AMI) between the profile labels of each pair of pathways across all cell types (clusters on heatmaps in Data S2). The AMI metric quantifies the degree of statistical dependence between the two clusterings, controlling for correlations expected in a null, or completely independent, model. The full dataset of 1,206 cell states was used for computing the pairwise AMI, assigning the profile label "0" to cell states that do not express a given pathway. We visualized the results with a heatmap showing the pairwise AMI values across the main recurrent pathways (Figure 6C).

In general, most pathway-pathway correlations were weak (AMI < 0.4) (Figure 6C). To ensure that the AMI was indeed capable of capturing correlations, we included a subset of the TGF-β receptors (the seven BMP receptors: Acvr1, Acvrl1, Bmpr1a, Bmpr1b, Acvr2a, Acvr2b, and Bmpr2) as a separate pathway ("BMP receptors"). Given their overlapping components, TGF-β and BMP showed elevated AMI values of $\sim$0.6, as expected (Figure 6C). A notable exception was the strong correlation between the ubiquitin-proteasome pathway and SRSF splice regulators, which arose predominantly from developmental cell states expressing ubiquitin-proteasome profile 1 with SRSF profiles 1 and 2 (Table S4). Other pathway pairs, consisting of TGF-β and Wnt exhibited weaker relationships, whereas the Notch pathway showed little correlation with almost all other pathways. These results suggest that, at least for the limited set of components considered here, different pathways seem to adopt profiles largely independently of one another.

Pathway profiles exhibit distinct dynamic behaviors during differentiation

The relative independence of profile usage between pathways provokes the dynamic question of when and how pathways switch profiles during development. At one extreme, profiles could switch in a stepwise fashion, changing one component at a time. At the opposite extreme, they could change multiple components simultaneously, directly switching from one profile to another. Further, either type of change could occur gradually or suddenly and could be temporally synchronized or unsynchronized between different pathways. As an initial step in addressing these questions, we explored pathway profile usage in two well-studied processes: neural crest and blood cell differentiation.

The neural crest is responsible for diverse cell types, including sensory neurons, autonomic cell types, and mesenchymal stem cells.[57,58] Further, TGF-β, Notch, Eph-ephrin, and Wnt all play key roles in its differentiation.[59] Soldatov et al. performed SMART-seq2 scRNA-seq analysis of neural crest development from E9.5.[60] Using the Slingshot package,[61] we constructed pseudotime trajectories from these data, and we identified seven distinct pseudotemporal stages (Figure 7A). All expression counts were scaled to match the normalization used in the integrated atlas (Figure 2; STAR Methods). This reconstruction recapitulated known cell fate trajectories, with neural crest progenitors differentiating into sensory neurons, autonomic neurons, and mesenchymal cells (Figure 7A). Except for a transient upregulation of Bmpr1b early on, the TGF-β profile was remarkably stable during the trajectory from progenitors to more differentiated cell types. The profile was dominated by Bmpr1a, Tgfbr1, Acvr2a, and Acvr2b (Figure 7B, first panel). Its closest match in the integrated atlas was profile 6, which exhibited a roughly similar composition but with higher relative Bmpr2 expression

(C) ForceAtlas projection and pseudotime reconstruction of early vascular differentiation.[29] Mesodermal progenitors differentiate into endothelial and erythroid cell fates (gray arrows).

(D) Dynamics of three core pathways for each of the two trajectories in (C): erythroid differentiation (upper row of heat maps) and endothelial differentiation (lower row). Colored dots indicate cell states in (C). Profile numbers indicate closest matches in reference profiles (Figure 3B for TGF-β and Data S2 for Notch and SRSF).

(E) Mosaic view of profile usage (schematic). Cell states can express each of their pathways, using any of the distinct available profiles (indicated schematically by profile ticks). In this way, cell states can be thought of, in part, as mosaics built from combinations of available pathway profiles.

(Figures 3B and S6A). Profile 6 occurs in the developing forebrain and spinal cord, adult mesenchymal, and adult podocyte cell types. The expression of TGF-β receptors is consistent with previous observations that TGF-β inhibition in neural crest stem cells leads to cardiovascular defects.[62] These results indicate that a developmental pathway can retain a stable profile along a differentiation trajectory.

In contrast to the stability of TGF-β along this trajectory, Notch components exhibited a step-like transition at the end of the pseudotime trajectory (Figure 7B, second panel). Progenitors predominantly express the receptors Notch1 and Notch2, the ligands Dll1 and Jag1, and high levels of R-fng. This closest match to this profile was Notch motif 16 (Figures 6A and S6B). Upon differentiation into sensory neurons, cells switched on expression of Notch1, Dll3, and M-fng, as well as a lower level of Jag2, while downregulating Notch2, thus changing to private profile 27 (Figure S5C). In contrast to the signaling pathways, the SRSF splicing regulators showed stable co-expression of almost all components, except for Srsf5 and Srsf12, at high levels, most similar to profile 1 (Figure 7B, third panel; Figure S6C). Thus, the transition to the sensory neural fate involves both maintenance of stable profiles, as observed for TGF-β and SRSF, as well as abrupt multi-gene alterations, as observed for Notch.

Next, as a second example, we analyzed hematopoiesis, which occurs in temporally and spatially overlapping waves in close proximity to blood vascular endothelial cells.[63] Mesodermal hematoendothelial progenitors differentiate into both endothelium and erythroid cells (E7.5–E8.5), allowing analysis of how pathway profiles change during a branched differentiation trajectory (Figure 7C). Endothelial cells exhibit "private" TGF-β profiles, characterized by expression of Acvrl1. Thus, they provide an opportunity to analyze how pathway profiles change during a branched transition and how private profiles are acquired dynamically.

We clustered the subset of hematoendothelial lineages from Pijuan-Sala et al.[29] (15,645 single cells), applied Slingshot to reconstruct branching pseudotime trajectories (Figure 7C), and then analyzed changes in TGF-β receptor expression profiles over these trajectories. The TGF-β profiles expressed multiple receptor variants, including the developmental receptor Acvr2b, most closely matching profile 5 from the integrated dataset (Figure S6A). The amplitude of profile expression decayed during the transition to erythroid fate, but the relative expression levels of different components were preserved. By contrast, cells differentiating into endothelial fates maintained expression of the core profile genes. These dynamics thus reveal that profiles can vary gradually in amplitude during differentiation.

By contrast, the Notch pathway showed an abrupt profile change during erythroid differentiation. The initial mesodermal cells expressed a profile resembling profile 1 (cf. Figures 7D and S6B). However, differentiation coincided with a reduction of expression of ligands and receptors to a profile resembling profile 24. Similar to the TGF-β dynamics, expression of most components faded out in the erythroid lineage, except for R-fng whose expression increased. By contrast, the endothelial lineage exhibited more complex expression dynamics, including a sequential switch from Dll1 and Dll3 ligands to Dll4 and Jag2 ligands, coinciding with activation of M-fng (Figure 7D). Finally, SRSF showed stable expression of a profile closely resembling that observed in the neural crest, across both lineages (Figure 7D, right). Taken together, these results show that pathways can exhibit stable expression states, dynamic multi-component changes, and gradual variation in amplitude during differentiation trajectories.

DISCUSSION

In multicellular organisms, a core set of molecular signaling pathways mediate a huge variety of developmental and physiological events. How can such a limited set of pathways play such a broad range of different roles? At a coarse level, each pathway may be considered competent for signaling in a given cell type if its receptors and other components are expressed and not inhibited by other cellular components. However, examining pathway expression patterns globally, as we did here, reveals a more subtle situation, in which pathways can be expressed in a finite number of distinct configurations, characterized by different expression levels for their components, all potentially competent to signal in response to suitable inputs. Each configuration could be functional in some contexts but nevertheless differ from other configurations in the specific input ligands it senses or the downstream effectors it activates within the cell.[3,4,6,8,20,35,56,64]

To find out what configurations exist, we focused on cell-cell signaling pathways known to use sets of partially redundant component variants. Each of these pathways was already known to adopt multiple expression configurations in specific biological contexts. However, cell atlas data permit a systematic analysis of expression profiles in a broad set of cell and tissue contexts (Figures 2, 3, 4, and 5), revealing what pathway profiles are expressed, how they correlate with one another between pathways (Figure 6C), and how they change dynamically during development (Figure 7).

The expression profiles of pathways are strikingly combinatorial. Across each of the four major pathways studied here (TGF-β, Notch, Wnt, and SRSF), no two components exhibited identical expression patterns, and all were differentially regulated in some cell types. Further, almost all motifs comprised multiple receptor and/or ligand variants. The number of distinct expression profiles for each pathway was much smaller than one would expect if individual components varied independently. For instance, the SRSF pathway with 11 components exhibits ~19 profiles, which is almost 2-fold smaller than the ~30 profiles observed for the 11 TGF-β receptors and far less than the 2^{11} = 2,048 pathway profiles one would expect if each of its 11 genes could independently vary between low and high expression states.

Expression profiles for different pathways appeared to vary independently across cell types (Figure 6C). This observation argues against tight coupling of specific expression receptor profiles in one pathway with those in another. However, it does not rule out the possibility that signaling through combinations of pathways could play special roles in some cases.[65] Comparison of pseudotime trajectories from two developmental contexts revealed both stability and dynamic change in pathway profiles. Specifically, TGF-β and SRSF exhibited relatively stable profiles during these trajectories. By contrast, the Notch pathway exhibited dynamic

multi-component expression changes (Figures 7B and 7D). In the future, it will be interesting to comprehensively analyze pathway expression dynamics from the point of view of pathway motifs.

While we focused on the pathways that show strong motif signatures, it is equally important to note that other pathways predominantly used cell-type-specific, or private, profiles (Figure 5C), and even the pathways that we focused on here also contained some private profiles (Figures S3D and S5C). Nevertheless, these results suggest a "mosaic" view of cells, in which each cell type adopts a particular motif or private profile for each of its general-purpose pathways (Figure 7E).

Why use motifs? Motifs could provide a rich but limited repertoire of distinct functional behaviors for each pathway.[64] One appealing possibility is that each motif provides a distinct but related function. Many-to-many protein interaction systems can in fact "compute" complex functions and change the function they compute by altering component expression levels.[11] For example, in a "combinatorial addressing" system, cell types that express different receptor profiles can respond to different ligand combinations, allowing increased cell type specificity in signaling.[4,35,64] A similar principle could apply to juxtacrine signaling pathways such as Notch and Eph-ephrin, where the combination of components expressed in a given cell type could control which other cell types it can communicate with, based on their own pathway expression profiles. In the case of SRSF, otherwise diverse cell types expressing the same motif might generate similar splicing patterns. In the future, it will be interesting to experimentally test whether individual motifs indeed confer distinct functional behaviors across the cell types in which they appear. If so, a more complete functional understanding of pathway motifs could contribute to allowing researchers to predict and control pathway activities in diverse cell types based on their gene expression profiles.

Limitations of the study

Several limitations apply to the findings reported here. First, pathway definition starts with a human-curated list of receptors, ligands, or other components or previously annotated pathway definitions. Different pathway definitions could potentially alter these results. Second, while comprehensive, the datasets used here are likely incomplete, and they could miss profiles used only by rare cell types or could inaccurately estimate dispersion scores if some cell types appear over- or under-represented. Note also that cell types with lower overall expression of pathway components, such as immune cells for TGF-β, were filtered out. Alternative approaches could account for these issues and retain a larger subset of cell types. Third, clustering is an imperfect representation of expression variation, potentially averaging over subtle quantitative differences in individual component levels between cells. In particular, unsynchronized single-cell dynamics, such as those that occur during Notch-dependent fate determination,[66] could therefore be missed. Moreover, we explored signaling dynamics in only a few developmental trajectories. A broader exploration of more developmental processes could potentially reveal other types of dynamic behaviors beyond those shown here. Finally, subcellular localization patterns, post-translational modifications, alternative splice forms, and other types of regulation could diversify the functional modes of the pathway beyond what can be detected by scRNA-seq. For example, TGF-β pathway receptors are regulated through post-translational modifications and recycling at the membrane.[67] On the other hand, alterations in transcription of individual receptor subunits can quantitatively and qualitatively alter the specificity with which the BMP pathway responds to different ligand combinations.[11,15] With improving single-cell technologies, we anticipate that it should eventually become possible to extend pathway motif analysis to the protein level.

STAR★METHODS

Detailed methods are provided in the online version of this paper and include the following:

- KEY RESOURCES TABLE
- RESOURCE AVAILABILITY
  - Lead contact
  - Materials availability
  - Data and code availability
- METHOD DETAILS
  - Clustering cells and defining cell states
  - Integration of multiple datasets
  - Clustering pathway profiles
  - Defining motifs and private profiles
  - Interpathway correlations
  - Pseudotime trajectory analysis

SUPPLEMENTAL INFORMATION

Supplemental information can be found online at https://doi.org/10.1016/j.xgen.2023.100463.

ACKNOWLEDGMENTS

We would like to thank Benjamin Emert, Heidi Klumpe, Rachael Kuintzle, Matthew Langley, James Linton, Nicolas Pelaez-Restrapo, Judy Shon, and other members of the Elowitz lab for suggestions and critical feedback on this work, as well as critical feedback from Miri Adler, Yaron Antebi, Jialong Jiang, Matt Thomson, and Kai Zinn. This research was supported by the Allen Discovery Center program under award no. UWSC10142, a Paul G. Allen Frontiers Group advised program of the Paul G. Allen Family Foundation, and by the National Institutes of Health grant R01 HD075335A. N.K. was supported in the summer of 2020 by the Samuel N. Vodopia and Carol J. Hasson SURF Endowment.

DECLARATION OF INTERESTS

The authors declare no competing interests.

Received: February 22, 2023
Revised: August 2, 2023
Accepted: November 15, 2023
Published: January 10, 2024

REFERENCES

1. Gerhart, J. (1999). 1998 Warkany lecture: signaling pathways in development. Teratology *60*, 226–239.
2. Lim, W., Mayer, B., and Pawson, T. (2015). Cell Signaling: Principles and Mechanisms (Garland Science).

CellPress
OPEN ACCESS

3. Antebi, Y.E., Nandagopal, N., and Elowitz, M.B. (2017). An operational view of intercellular signaling pathways. Curr. Opin. Struct. Biol. *1*, 16–24.
4. Li, P., and Elowitz, M.B. (2019). Communication codes in developmental signaling pathways. Development *146*, dev170977.
5. Massagué, J. (2012). TGFβ signalling in context. Nat. Rev. Mol. Cell Biol. *13*, 616–630.
6. Verkaar, F., and Zaman, G.J.R. (2010). A model for signaling specificity of Wnt/Frizzled combinations through co-receptor recruitment. FEBS Lett. *584*, 3850–3854.
7. Okigawa, S., Mizoguchi, T., Okano, M., Tanaka, H., Isoda, M., Jiang, Y.-J., Suster, M., Higashijima, S.-I., Kawakami, K., and Itoh, M. (2014). Different combinations of Notch ligands and receptors regulate V2 interneuron progenitor proliferation and V2a/V2b cell fate determination. Dev. Biol. *391*, 196–206.
8. Rohani, N., Parmeggiani, A., Winklbauer, R., and Fagotto, F. (2014). Variable combinations of specific ephrin ligand/Eph receptor pairs control embryonic tissue separation. PLoS Biol. *12*, e1001955.
9. Wang, Y., Chang, H., Rattner, A., and Nathans, J. (2016). Frizzled Receptors in Development and Disease. Curr. Top. Dev. Biol. *117*, 113–139.
10. Derynck, R., and Budi, E.H. (2019). Specificity, versatility, and control of TGF-β family signaling. Sci. Signal. *12*, eaav5183.
11. Klumpe, H.E., Garcia-Ojalvo, J., Elowitz, M.B., and Antebi, Y.E. (2023). The computational capabilities of many-to-many protein interaction networks. Cell Syst. *14*, 430–446.
12. David, C.J., and Massagué, J. (2018). Contextual determinants of TGFβ action in development, immunity and cancer. Nat. Rev. Mol. Cell Biol. *19*, 419–435.
13. Wrana, J.L., Attisano, L., Cárcamo, J., Zentella, A., Doody, J., Laiho, M., Wang, X.-F., and Massagué, J. (1992). TGFβ signals through a heteromeric protein kinase receptor complex. Cell *71*, 1003–1014.
14. Vilar, J.M.G., Jansen, R., and Sander, C. (2006). Signal processing in the TGF-beta superfamily ligand-receptor network. PLoS Comput. Biol. *2*, e3.
15. Antebi, Y.E., Linton, J.M., Klumpe, H., Bintu, B., Gong, M., Su, C., McCardell, R., and Elowitz, M.B. (2017). Combinatorial Signal Perception in the BMP Pathway. Cell *170*, 1184–1196.e24.
16. Voloshanenko, O., Gmach, P., Winter, J., Kranz, D., and Boutros, M. (2017). Mapping of Wnt-Frizzled interactions by multiplex CRISPR targeting of receptor gene families. Faseb. J. *31*, 4832–4844.
17. Eubelen, M., Bostaille, N., Cabochette, P., Gauquier, A., Tebabi, P., Dumitru, A.C., Koehler, M., Gut, P., Alsteens, D., Stainier, D.Y.R., et al. (2018). A molecular mechanism for Wnt ligand-specific signaling. Science *361*, eaat1178.
18. Goentoro, L., and Kirschner, M.W. (2009). Evidence that fold-change, and not absolute level, of beta-catenin dictates Wnt signaling. Mol. Cell *36*, 872–884.
19. Sprinzak, D., Lakhanpal, A., Lebon, L., Santat, L.A., Fontes, M.E., Anderson, G.A., Garcia-Ojalvo, J., and Elowitz, M.B. (2010). Cis-interactions between Notch and Delta generate mutually exclusive signalling states. Nature *465*, 86–90.
20. LeBon, L., Lee, T.V., Sprinzak, D., Jafar-Nejad, H., and Elowitz, M.B. (2014). Fringe proteins modulate Notch-ligand cis and trans interactions to specify signaling states. Elife *3*, e02950.
21. Groot, A.J., Habets, R., Yahyanejad, S., Hodin, C.M., Reiss, K., Saftig, P., Theys, J., and Vooijs, M. (2014). Regulated proteolysis of NOTCH2 and NOTCH3 receptors by ADAM10 and presenilins. Mol. Cell Biol. *34*, 2822–2832.
22. Lafkas, D., Shelton, A., Chiu, C., de Leon Boenig, G., Chen, Y., Stawicki, S.S., Siltanen, C., Reichelt, M., Zhou, M., Wu, X., et al. (2015). Therapeutic antibodies reveal Notch control of transdifferentiation in the adult lung. Nature *528*, 127–131.
23. Kania, A., and Klein, R. (2016). Mechanisms of ephrin–Eph signalling in development, physiology and disease. Nat. Rev. Mol. Cell Biol. *17*, 240–256.
24. Klein, R. (2012). Eph/ephrin signalling during development. Development *139*, 4105–4109.
25. Tabula Muris Consortium; Overall coordination; Logistical coordination; Organ collection and processing; Library preparation and sequencing; Computational data analysis; Cell type annotation; Writing group; Supplemental text writing group; Principal investigators (2018). Single-cell transcriptomics of 20 mouse organs creates a Tabula Muris. Nature *562*, 367–372.
26. Tabula Muris Consortium (2020). A single-cell transcriptomic atlas characterizes ageing tissues in the mouse. Nature *583*, 590–595.
27. Chan, M.M., Smith, Z.D., Grosswendt, S., Kretzmer, H., Norman, T.M., Adamson, B., Jost, M., Quinn, J.J., Yang, D., Jones, M.G., et al. (2019). Molecular recording of mammalian embryogenesis. Nature *570*, 77–82.
28. He, P., Williams, B.A., Trout, D., Marinov, G.K., Amrhein, H., Berghella, L., Goh, S.-T., Plajzer-Frick, I., Afzal, V., Pennacchio, L.A., et al. (2020). The changing mouse embryo transcriptome at whole tissue and single-cell resolution. Nature *583*, 760–767.
29. Pijuan-Sala, B., Griffiths, J.A., Guibentif, C., Hiscock, T.W., Jawaid, W., Calero-Nieto, F.J., Mulas, C., Ibarra-Soria, X., Tyser, R.C.V., Ho, D.L.L., et al. (2019). A single-cell molecular map of mouse gastrulation and early organogenesis. Nature *566*, 490–495.
30. Nowotschin, S., Setty, M., Kuo, Y.-Y., Liu, V., Garg, V., Sharma, R., Simon, C.S., Saiz, N., Gardner, R., Boutet, S.C., et al. (2019). The emergent landscape of the mouse gut endoderm at single-cell resolution. Nature *569*, 361–367.
31. Dong, J., Hu, Y., Fan, X., Wu, X., Mao, Y., Hu, B., Guo, H., Wen, L., and Tang, F. (2018). Single-cell RNA-seq analysis unveils a prevalent epithelial/mesenchymal hybrid state during mouse organogenesis. Genome Biol. *19*, 31.
32. Qiu, C., Cao, J., Martin, B.K., Li, T., Welsh, I.C., Srivatsan, S., Huang, X., Calderon, D., Noble, W.S., Disteche, C.M., et al. (2022). Systematic reconstruction of cellular trajectories across mouse embryogenesis. Nat. Genet. *54*, 328–341.
33. Massagué, J., and Sheppard, D. (2023). TGF-β signaling in health and disease. Cell *186*, 4007–4037.
34. Rousseeuw, P.J. (1987). Silhouettes: A graphical aid to the interpretation and validation of cluster analysis. J. Comput. Appl. Math. *20*, 53–65.
35. Klumpe, H., Langley, M.A., Linton, J.M., Su, C.J., Antebi, Y.E., and Elowitz, M.B. (2020). The context-dependent, combinatorial logic of BMP signaling. Preprint at bioRxiv.
36. Chen, H., Brady Ridgway, J., Sai, T., Lai, J., Warming, S., Chen, H., Roose-Girma, M., Zhang, G., Shou, W., and Yan, M. (2013). Context-dependent signaling defines roles of BMP9 and BMP10 in embryonic and postnatal development. Proc. Natl. Acad. Sci. USA *110*, 11887–11892.
37. Wishart, D.S., Li, C., Marcu, A., Badran, H., Pon, A., Budinski, Z., Patron, J., Lipton, D., Cao, X., Oler, E., et al. (2020). PathBank: a comprehensive pathway database for model organisms. Nucleic Acids Res. *48*, D470–D478.
38. Artavanis-Tsakonas, S., Rand, M.D., and Lake, R.J. (1999). Notch signaling: cell fate control and signal integration in development. Science *284*, 770–776.
39. D'Souza, B., Miyamoto, A., and Weinmaster, G. (2008). The many facets of Notch ligands. Oncogene *27*, 5148–5167.
40. Siebel, C., and Lendahl, U. (2017). Notch Signaling in Development, Tissue Homeostasis, and Disease. Physiol. Rev. *97*, 1235–1294.
41. Kakuda, S., and Haltiwanger, R.S. (2017). Deciphering the Fringe-Mediated Notch Code: Identification of Activating and Inhibiting Sites Allowing Discrimination between Ligands. Dev. Cell *40*, 193–201.

42. Kakuda, S., LoPilato, R.K., Ito, A., and Haltiwanger, R.S. (2020). Canonical Notch ligands and Fringes have distinct effects on NOTCH1 and NOTCH2. J. Biol. Chem. *295*, 14710–14722.
43. Yoon, S.-O., Zhang, X., Berner, P., Blom, B., and Choi, Y.S. (2009). Notch ligands expressed by follicular dendritic cells protect germinal center B cells from apoptosis. J. Immunol. *183*, 352–358.
44. Saito, T., Chiba, S., Ichikawa, M., Kunisato, A., Asai, T., Shimizu, K., Yamaguchi, T., Yamamoto, G., Seo, S., Kumano, K., et al. (2003). Notch2 is preferentially expressed in mature B cells and indispensable for marginal zone B lineage development. Immunity *18*, 675–685.
45. Tissue Expression of DLL3 - Summary - the Human Protein Atlas https://www.proteinatlas.org/ENSG00000090932-DLL3/tissue.
46. del Álamo, D., Rouault, H., and Schweisguth, F. (2011). Mechanism and significance of cis-inhibition in Notch signalling. Curr. Biol. *21*, R40–R47.
47. Nandagopal, N., Santat, L.A., and Elowitz, M.B. (2019). activation in the Notch signaling pathway. Elife *8*, e37880.
48. Long, J.C., and Caceres, J.F. (2009). The SR protein family of splicing factors: master regulators of gene expression. Biochem. J. *417*, 15–27.
49. Zhou, Z., and Fu, X.-D. (2013). Regulation of splicing by SR proteins and SR protein-specific kinases. Chromosoma *122*, 191–207.
50. Jeong, S. (2017). SR Proteins: Binders, Regulators, and Connectors of RNA. Mol. Cells *40*, 1–9.
51. Zahler, A.M., Neugebauer, K.M., Lane, W.S., and Roth, M.B. (1993). Distinct functions of SR proteins in alternative pre-mRNA splicing. Science *260*, 219–222.
52. Hammarskjold, M.-L., and Rekosh, D. (2017). SR proteins: To shuttle or not to shuttle, that is the question. J. Cell Biol. *216*, 1875–1877.
53. Grigoryan, T., Wend, P., Klaus, A., and Birchmeier, W. (2008). Deciphering the function of canonical Wnt signals in development and disease: conditional loss- and gain-of-function mutations of beta-catenin in mice. Genes Dev. *22*, 2308–2341.
54. Mikels, A.J., and Nusse, R. (2006). Wnts as ligands: processing, secretion and reception. Oncogene *25*, 7461–7468.
55. MacDonald, B.T., and He, X. (2012). Frizzled and LRP5/6 receptors for Wnt/β-catenin signaling. Cold Spring Harbor Perspect. Biol. *4*, a007880.
56. Buckles, G.R., Thorpe, C.J., Ramel, M.-C., and Lekven, A.C. (2004). Combinatorial Wnt control of zebrafish midbrain-hindbrain boundary formation. Mech. Dev. *121*, 437–447.
57. Simões-Costa, M., and Bronner, M.E. (2015). Establishing neural crest identity: a gene regulatory recipe. Development *142*, 242–257.
58. Kléber, M., Lee, H.-Y., Wurdak, H., Buchstaller, J., Riccomagno, M.M., Ittner, L.M., Suter, U., Epstein, D.J., and Sommer, L. (2005). Neural crest stem cell maintenance by combinatorial Wnt and BMP signaling. J. Cell Biol. *169*, 309–320.
59. Bhatt, S., Diaz, R., and Trainor, P.A. (2013). Signals and switches in Mammalian neural crest cell differentiation. Cold Spring Harbor Perspect. Biol. *5*, a008326.
60. Soldatov, R., Kaucka, M., Kastriti, M.E., Petersen, J., Chontorotzea, T., Englmaier, L., Akkuratova, N., Yang, Y., Häring, M., Dyachuk, V., et al. (2019). Spatiotemporal structure of cell fate decisions in murine neural crest. Science *364*, eaas9536.
61. Street, K., Risso, D., Fletcher, R.B., Das, D., Ngai, J., Yosef, N., Purdom, E., and Dudoit, S. (2018). Slingshot: cell lineage and pseudotime inference for single-cell transcriptomics. BMC Genom. *19*, 477.
62. Wurdak, H., Ittner, L.M., Lang, K.S., Leveen, P., Suter, U., Fischer, J.A., Karlsson, S., Born, W., and Sommer, L. (2005). Inactivation of TGF signaling in neural crest stem cells leads to multiple defects reminiscent of DiGeorge syndrome. Genes Dev. *19*, 530–535.
63. Canu, G., and Ruhrberg, C. (2021). First blood: the endothelial origins of hematopoietic progenitors. Angiogenesis *24*, 199–211.
64. Su, C.J., Murugan, A., Linton, J.M., Yeluri, A., Bois, J., Klumpe, H., Antebi, Y.E., and Elowitz, M.B. (2020). Ligand-receptor promiscuity enables cellular addressing. Preprint at bioRxiv.
65. Muñoz Descalzo, S., and Martinez Arias, A. (2012). The structure of Wntch signalling and the resolution of transition states in development. Semin. Cell Dev. Biol. *23*, 443–449.
66. Kageyama, R., Shimojo, H., and Isomura, A. (2018). Oscillatory Control of Notch Signaling in Development. Adv. Exp. Med. Biol. *1066*, 265–277.
67. Yakymovych, I., Yakymovych, M., and Heldin, C.-H. (2018). Intracellular trafficking of transforming growth factor β receptors. Acta Biochim. Biophys. Sin. *50*, 3–11.
68. Wolf, F.A., Angerer, P., and Theis, F.J. (2018). SCANPY: large-scale single-cell gene expression data analysis. Genome Biol. *19*, 15.
69. Traag, V.A., Waltman, L., and van Eck, N.J. (2019). From Louvain to Leiden: guaranteeing well-connected communities. Sci. Rep. *9*, 5233.
70. Jacomy, M. (2011). Force Atlas 2 Layout.

STAR★METHODS

KEY RESOURCES TABLE

REAGENT or RESOURCE	SOURCE	IDENTIFIER
Deposited data		
All raw and processed data	This paper	https://doi.org/10.22002/hf6zq-zmg82
Forelimb mouse atlas	He et al.[28]	ENCODE: ENCSR713GIS
Early gastrulation mouse atlas	Pijuan-Sala et al.[29]	https://github.com/MarioniLab/EmbryoTimecourse2018
Early gastrulation mouse atlas (endoderm)	Nowotschin et al.[30]	https://endoderm-explorer.com/
Epithelial/mesenchymal transition atlas	Dong et al.[31]	GEO: GSE87038
Mammalian embryogenesis atlas	Chang et al.[27]	GEO: GSE122187
Tabula Muris and Tabula Muris Senis	Tabula Muris Consortium[25,26]	tabula-muris-senis.ds.czbiohub.org
Software and algorithms		
Code for all analyses and figures generated in this paper	This paper	Release version: https://doi.org/10.22002/37gwp-bjg24 and Development version: https://github.com/labowitz/motifs
Scanpy	Wolf et al.[68]	https://scanpy.readthedocs.io/en/stable/
Motif finder	This study	https://github.com/labowitz/motifs
PathBank	Wishart et al.[40]	https://www.pathbank.org/

RESOURCE AVAILABILITY

Lead contact

Further information and requests for resources should be directed to Michael Elowitz (melowitz@caltech.edu).

Materials availability

This study did not generate new reagents.

Data and code availability

Raw data were obtained from indicated authors' works, but we include these files, along with the processed data at https://doi.org/10.22002/hf6zq-zmg82. Released versions of the code can be found at https://doi.org/10.22002/37gwp-bjg24 and code in development at https://github.com/labowitz/motifs.

METHOD DETAILS

Clustering cells and defining cell states

We obtained raw scRNA-seq matrices directly from the GEO repositories or specific locations indicated by the authors for the datasets appearing in Table S1. Clustering of single cells started from the count matrices of single cells vs. genes. First, we applied quality control (when needed, since some datasets were already filtered) by filtering out cells with high mitochondrial RNA content, a low number of detected transcripts or a low number of detected counts (at least 2,000 counts per cell). We then applied a standard pipeline for clustering scRNA-seq data. Briefly, we applied principal component analysis and used the first 50 principal components as input for graph-based (Leiden) clustering using Scanpy.[68,69] Finally, we labeled the resulting clusters using the cell type annotations provided by the authors. All datasets analyzed in this study included ground truth cell type annotations that we use throughout the manuscript.

Integration of multiple datasets

To integrate the 7 datasets in Table S1, spanning 14 different timepoints, into a single matrix of gene expression, we first generated a pseudo-bulk expression matrix for each dataset by averaging the log-normalized gene expression values of individual cells in a cluster. The resulting matrix has dimensions N x M, where N is the number of cell states in the dataset and M is the number of distinct genes. To account for differences in gene detection across datasets, we found the intersection of detected genes across all datasets and subsampled each matrix to include only genes that appeared in all datasets. The intersection of detected genes across all

datasets comprised ~11,000 genes that we then used for all downstream analysis. Having defined the intersection gene set, we concatenated individual datasets into a global average expression matrix containing 1206 clusters and ~11,000 genes.

To normalize gene expression values from different datasets to a common scale, we applied a second round of normalization to the global expression matrix. First, we transformed the log-normalized matrix M using the exponential function to obtain a matrix M_{ij} of "counts" per gene: $M'_{ij} = exp(M_{ij}) + 1$. We then normalized, scaled and clustered the resulting matrix following the standard methods from Seurat v3 (total RNA counts per cell state = 1e4, 4,000 highly-variable genes, and 50 principal components), which resulted in the clustering and UMAP shown in Figure 2B. We verified that cell states from different datasets and sequencing technologies clustered together (Figure S1B), as an indication that the integrated and normalized atlas recovers the biological diversity across development, adult, and aging datasets.

Clustering pathway profiles

All downstream analysis on pathway genes starts from a matrix of normalized pathway gene counts subsetted from the matrix M' described above. We noticed that pathway genes showed different dynamic ranges in their expression across cell states. To give each gene equal weight during clustering of pathway profiles, we applied a MinMax scaling for each pathway gene, using the 95% percentile observed across all 1206 cell states as the maximum value. After scaling, each gene in the pathway had a dynamic range from 0 to 1, corresponding to the range of 0–95% of the maximum value in the dataset for that gene. For each cell state, we classified a pathway as being "on"' if at least two of the pathway genes showed expression above a threshold of 0.3 on this scale, meaning that the gene is expressed at a level of at least 30% of its maximum observed value. This threshold allowed us to filter out cell states in which most genes in the pathway are zero or showed low expression compared to most other cell states, and focus instead on the cell states showing combinatorial expression of multiple genes (Figure S2C). This pre-processing step resulted in a matrix P of scaled pathway gene expression counts of cell states with an "on" pathway profile. Using this matrix P, we computed pairwise cosine distances on cell states and applied hierarchical clustering to the resulting distance matrix (Figure 3A). Finally, we applied the same pre-processing steps and obtained the pathway profiles for 55 pathways from the PathBank[37] database annotated as 'Signaling' or 'Protein' in PathBank, excluding pathways with less than 7 genes (Table S3; Data S2).

For each pathway, we found the approximate optimal number of clusters, k_{opt}, using the silhouette score metric. First, we applied hierarchical clustering to the pathway expression matrix P, and defined the number of clusters, k, by setting a depth cut-off and splitting the associated dendrogram (Figure 3A). We then bootstrapped the average silhouette score on the pathway expression matrix for a range of k values (from 3 to 100). To account for potential clustering artifacts, we normalized to a null distribution and randomized the pathway gene expression matrix, shuffling the expression values for each gene independently across cell states, and repeated the clustering procedure. By independently scrambling P, the pathway expression matrix, we could generate a sample from the null distribution for the expected silhouette score at different values of k (Figure 3A, gray). We found that after ~100 randomizations, the silhouette score distribution converged and the Z score calculations were not significantly affected by increasing the number of random datasets beyond that value. Using this null distribution, we computed z-scores (Figure 3A, blue) for the silhouette scores observed in the real pathway expression matrix. Finally, we defined the optimal number of clusters, k_{opt}, to be the largest value of k for which the smoothed Z score dropped below 90% of its maximum value. To normalize for number of genes N_g in the pathway definition, we defined the recurrence score of pathways to be $r = k_{opt}/N_g$.

Some pathways did not show a clear peak in the Z score (Figure 5A, bottom; Figure S4A, right) meaning an optimal number of clusters can't be reliably found. To focus on pathways with a well-defined peak, we computed the range of k values with a Z score within 90% of the maximum. We therefore defined the peak width as the fraction of k values within 90% of the maximum Z score divided by the total number of k values considered (200) and excluded pathways with peak width greater than 0.35 (Figure 5B).

Defining motifs and private profiles

Having defined the k_{opt} clusters, or pathway profiles, we computed the diversity of cell states expressing each profile based on their transcriptome similarity. In principle, pathway profiles might comprise similar cell types (high transcriptome similarity) or sets of diverse cell types (low transcriptome similarity). We calculated their pairwise Euclidean distances in the PCA projection constructed from the top 4,000 highly variable genes (100 principal components) to measure transcriptome similarity in a subset of cell states. We first verified that this metric was low for closely related cell states (as defined by their cell type annotation) and largest for randomly selected cell states (Figures 4B and 4C). We then defined *dispersion* as the average pairwise PCA distance among a subset of cell states (Figure 4A).

To find the lower bound of dispersion, we computed the expected dispersion for related cell states by clustering their transcriptomes using the first 100 principal components, resulting in a global dendrogram of cell states (Figure 3C). We then identified the clustering threshold for the global dendrogram to obtain the same number of clusters k as observed for the pathway in question, therefore generating k groups of cell states that are each closely related. We then compared the distribution of dispersions for clusters of related cell states and the dispersions for cell states within the pathway profiles (Figure 4C). The dispersion distribution observed for related cell states (Figure 4C, gray) defines an approximate lower bound for the expected dispersion (Figure 4C, turquoise). Conversely, we also computed dispersion values for randomly selected groups of cell states (Figure 4C, black). Random groups of cell states provide the dispersion expected if pathway expression states were completely uncorrelated with the overall expression similarity of the cells in which they appear. Finally, we defined a pathway profile as a *motif* if the cell states expressing

it showed dispersion values higher than the 90% percentile value expected for related cell states (Figure 4C, shaded area). The 90% percentile threshold in dispersion identified pathway profiles expressed in the most diverse set of cell states. However, we observed additional pathway states that appeared dispersed among cell types but did not pass the 90% threshold. Therefore, this method could underestimate the number of dispersed pathway profiles and the threshold can be adjusted to allow a more flexible definition of pathway motifs.

In contrast to pathway motifs, "private" profiles are cell-state specific, effectively the opposite of motifs (Figure S3D). By definition, private profiles are confined to sets of similar cell states and therefore show low dispersion values. To classify private profiles, we identified those profiles whose cell state dispersion overlapped with the expectation for highly-related cell states. Specifically, we considered profiles with dispersion <50% percentile of the lower-bound distribution as "private." For a pathway to be cell-state specific we expected the dispersion to be similar to that observed in closely related cell states. The threshold can be increased to allow for identification of other pathway profiles with dispersion values comparable to related cell states.

Interpathway correlations

To detect potential statistical dependence between pathway states from different signaling pathways, we computed a pairwise Adjusted Mutual Information (AMI) for each pair of pathways (labels in Table S4). The AMI quantifies statistical dependencies between categorical features in a dataset. In this case, each cell state has two different categorical labels, one for each pathway. The AMI accounts for the expected correlations if the two labels are assigned at random. An AMI value of 0 represents the expected co-occurrence of labels due to chance, while a value of 1 represents perfect statistical dependence between the two clusterings.

Pseudotime trajectory analysis

To study transitions in pathway signaling profiles through the course of developmental processes, we performed pseudotime trajectory analysis on two developmental datasets that were not included in the main integrated dataset (Figure 2): the neural crest developmental lineage from embryonic day 9.5,[60] and the haemato-endothelial lineages from embryonic development days 7.5–8.5 subsetted from a scRNA-seq atlas of early organogenesis.[29] We clustered single-cell data as described above (*Clustering cells and defining cell states*) and constructed a force-directed projection using the ForceAtlas2 algorithm.[70] We used cluster annotations and the ForceAtlas2 reduced dimensional space as input to the Slingshot algorithm[61] to obtain a global lineage structure. We then placed cell states in the ordering given by the resulting pseudotime coordinates (Figures 7A and 7C). We scaled the gene expression values of the developmental dataset using min-max scaling after log-normalization. This scaling was performed to align each gene's expression distribution with the 0 and 0.95 quantiles of the corresponding gene in the integrated dataset. This allowed for direct comparability of developmental profiles with the integrated dataset, as depicted in Figures 7B and 7D. Finally, we used the k-nearest neighbors algorithm to obtain the profile numbers which closest match a given cell state along a developmental trajectory (Figures 7B and 7D, numbers).

Cell Genomics, Volume *4*

Supplemental information

Combinatorial expression motifs in signaling pathways

Alejandro A. Granados, Nivedita Kanrar, and Michael B. Elowitz

Table S1. List of single-cell RNA seq datasets used in this study (related to main figures 1 and 2)

Dataset	Time points	Reference	Cells	Mice sampled	Technology	No. of Cell States in Integrated Atlas
Forelimb atlas (The changing mouse embryo transcriptome at whole tissue and single-cell resolution)	E10.5, E11.0, E11.5, E12.0, E13.0, E13.5, E14.0, E15.0	[28]	90,637	Pair of forelimbs per time point	10X	90
A single-cell molecular map of mouse gastrulation and early organogenesis	E6.5, E6.75, E7.0, E7.25, E7.5, E7.75, E8.0, E8.25, E8.5	[63]	116,312	411 mouse embryos	10X	134
The emergent landscape of the mouse gut endoderm at single-cell resolution	E5.5	[30]	-	-	10X	20
Single-cell RNA-seq analysis unveils a prevalent epithelial/mesenchymal hybrid state during mouse organogenesis	E9.5, E10.5, E11.5	[31]	1916	7 embryos	Smart-seq2	28
Molecular recording of mammalian embryogenesis	E6.5, E7.0, E7.5, E8.0, E8.5	[27]	88,779	50 embryos	10X	181
Tabula Muris, Tabula Muris Senis	1mo, 3mo, 18mo, 21mo, 24mo, 30mo	[25,26]	450,000+	-	10X, Smart-seq2	753

Supplementary Figure 1

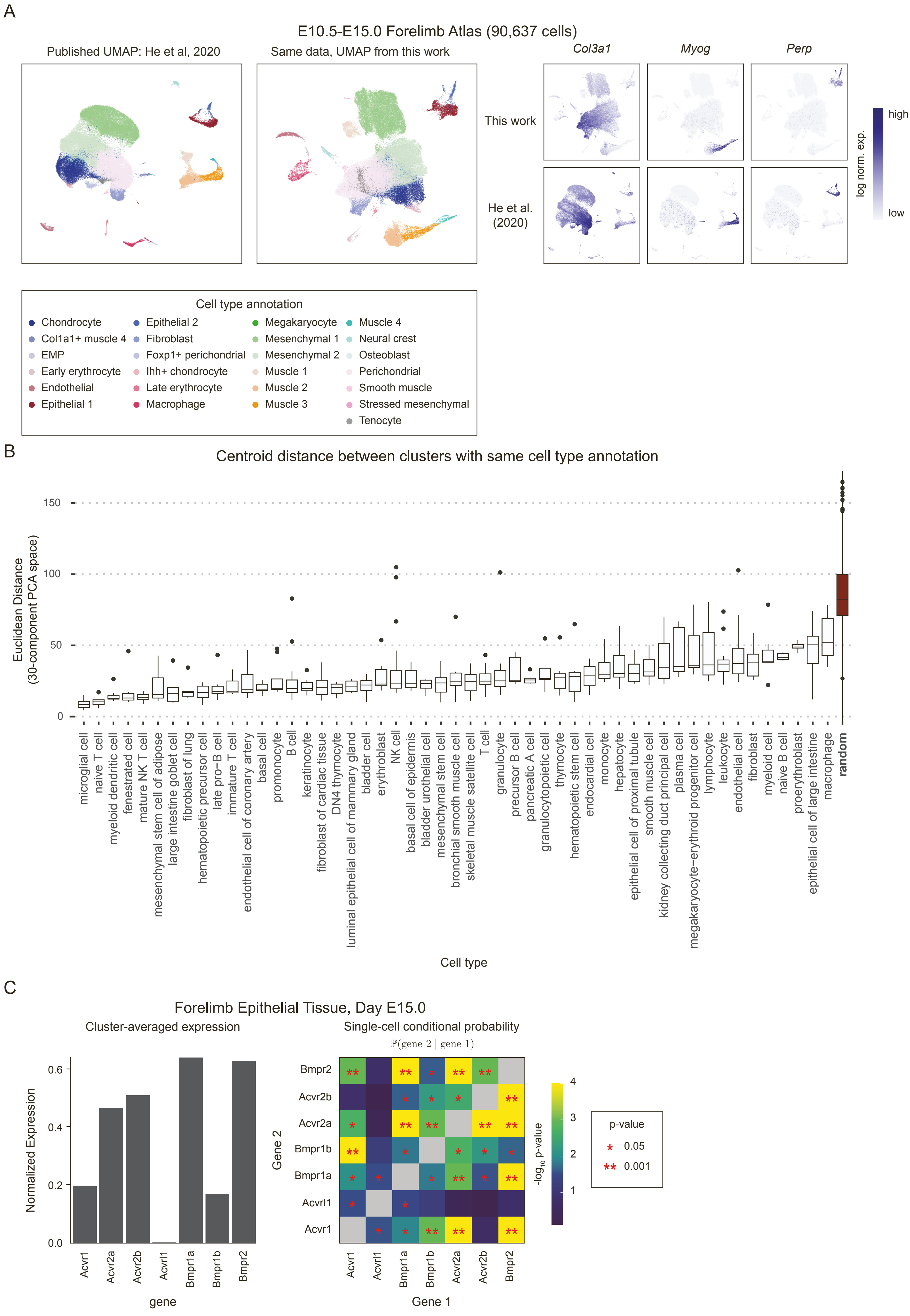

Figure S1. Single-cell RNA seq analysis and expression of TGF-β receptors (related to main figure 2)

A. Analysis of scRNA-seq datasets using the standard Scanpy pipeline recapitulates published analyses, including [28]. Independent analysis of mouse forelimb over days E10.5-E15.0 captures similarity in cell types (colors, left) and gene expression (right).
B. The integrated atlas captures cell type similarity across datasets. Cell clusters with similar annotations in different data sets remain similar to each other in the integrated atlas.
C. Cluster-averaged profiles reflect co-expression in single-cells. Shown is an example of a single cluster from the forelimb epithelial tissue data set at day E15. Left, expression of TGF-β receptor genes averaged over all cells in the cluster corresponding to forelimb epithelial tissue at day E15.0. Right, pairwise conditional probability in single cells of gene 2 expression conditioned on gene 1 expression. Pairs of genes with significant entries (**) are co-expressed in the cluster-averaged profile. Higher-order conditional probabilities were not computed due to dropout effects in scRNA-seq data.

Supplementary Figure 2

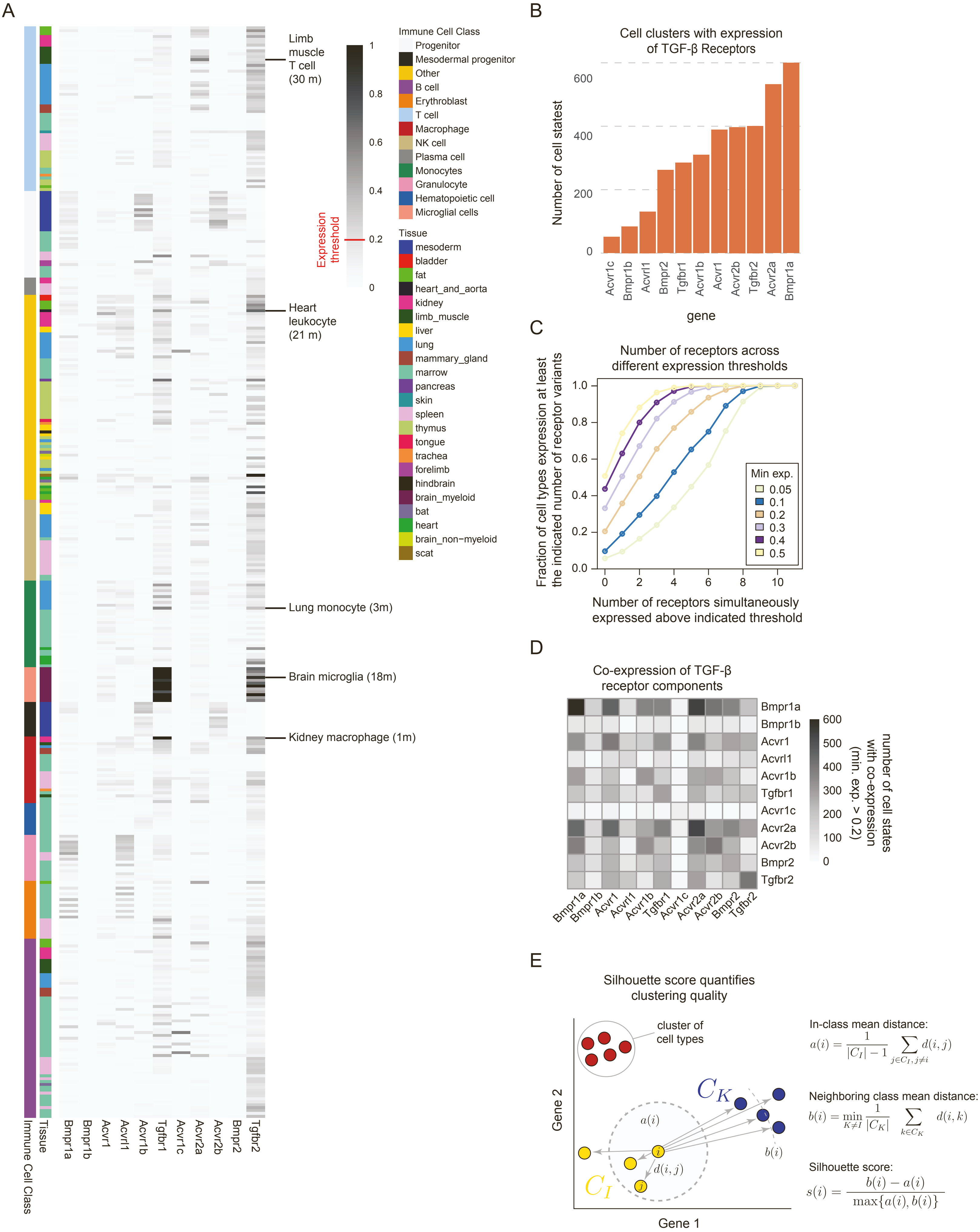

Figure S2. Co-expression statistics of TGF-β receptors across all cell states (related to main figure 3).

A. A heatmap showing the average normalized expression of TGF-β receptors for the immune cell types not included in the clustering analysis due to low mRNA expression levels. Each gene's average expression level was normalized from 0 to 1 relative to the minimum and maximum values observed across all 1206 cell types (Figure 3B, Methods). A cell type was included if it showed normalized expression above 0.2 for more than two receptors. The color side bards indicate the tissue of origin and the broad immune cell class.
B. Histogram showing the number of cell types in the integrated atlas with normalized expression of TGF-β receptors above a threshold of 0.2 in standardized expression units.
C. The number of TGF-β receptor components simultaneously expressed for different values of the minimum expression threshold (colors).
D. Pairwise co-expression of TGF-β receptor expression reveals broad receptor co-expression patterns. Off-diagonal elements indicate the number of cell states co-expressing, above threshold, the indicated pair of components. Diagonal elements indicate the number of cell states expressing the corresponding individual gene (as shown in A).
E. The silhouette score quantifies clustering quality (schematic). For a given clustering, we compute the silhouette score on every data point i. We compute $a(i)$, the mean distance between i and every other point in the same cluster, and $b(i)$, the mean distance between i and the nearest neighboring cluster. The silhouette score for the data point i is then defined as the difference between the inter- and intra-cluster distances, normalized to the maximum of the two (equations). A silhouette score value close to 1 corresponds to well-defined clusters, where the data point i is similar to other members of its cluster and dissimilar to others. In contrast, a value close to -1 suggests poor cluster assignment. Finally, the silhouette score for a given clustering, is taken as the average of the individual scores for all data points. In our analysis, a data point corresponds to a vector of the scaled, normalized counts of pathway gene expression of a single cell state.

Supplementary Figure 3

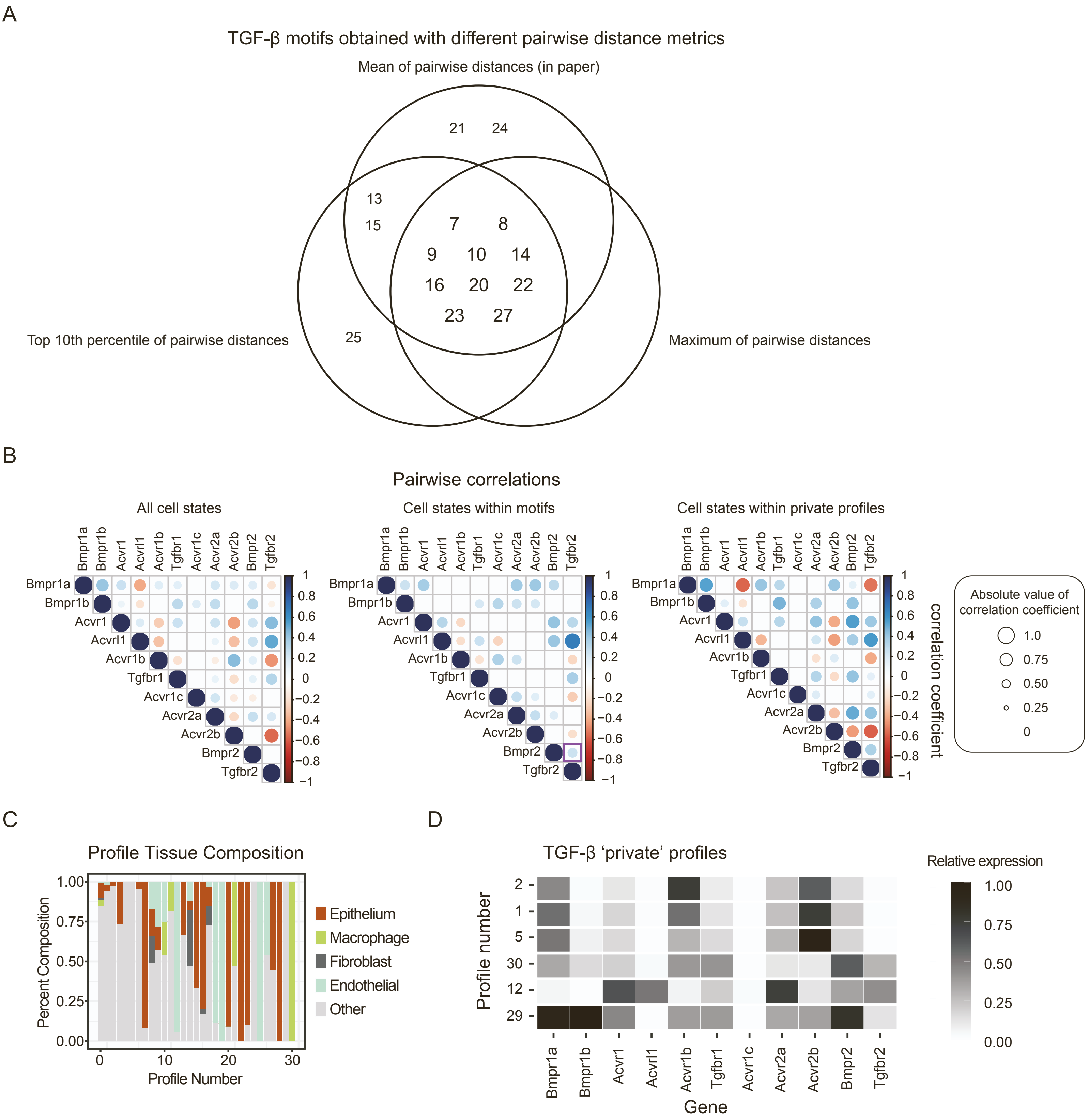

Figure S3. Dispersion metrics and private profiles for TGF-β (related to main figures 3 and 4).

A. Alternative definitions of the dispersion metric recover similar sets of motifs. The mean of intra-class pairwise distances was used as the dispersion metric throughout this work. Still, we tested two additional dispersion metrics, one that uses the maximum of intra-class pairwise distances, the second that uses the top 10th percentile. The Venn diagram shows profiles identified as motifs from these three distinct definitions of the dispersion metric. Most profiles (shown in the intersection of the three circles) are robust to the definition of dispersion. Notably, the dispersion metric that utilizes the maximum pairwise distances only captures profiles in this intersection. The mean pairwise distance, however, captures two additional profiles as motifs, profiles 21 and 24. Profile 24 contains only two cell states, liver B cells and bone marrow NK cells. The top 10th percentile of pairwise distances captures the adult endothelium-specific profile, 25, as a motif. However, the maximum metric omits profiles 13 and 15, even though they appear to be motifs since they are both dispersed across the adult smooth muscle and adult kidney epithelium.
B. TGF-β profiles exhibit unique pairwise receptor correlations. Each matrix represents the correlation coefficient for each pair of receptors across all cell states (left), cell states associated with motifs (middle), and cell states associated with private profiles (right).
C. Cell class composition of each TGF-β profile, matching classes depicted in Figure 4F. “Other” includes all cell states in the atlas that do not fall into the epithelial, macrophage, fibroblast, or endothelial cell classes.
D. TGF-β profiles with average dispersion less than the 50 percentile from the expected distribution were classified as *private profiles*.

Supplementary Figure 4

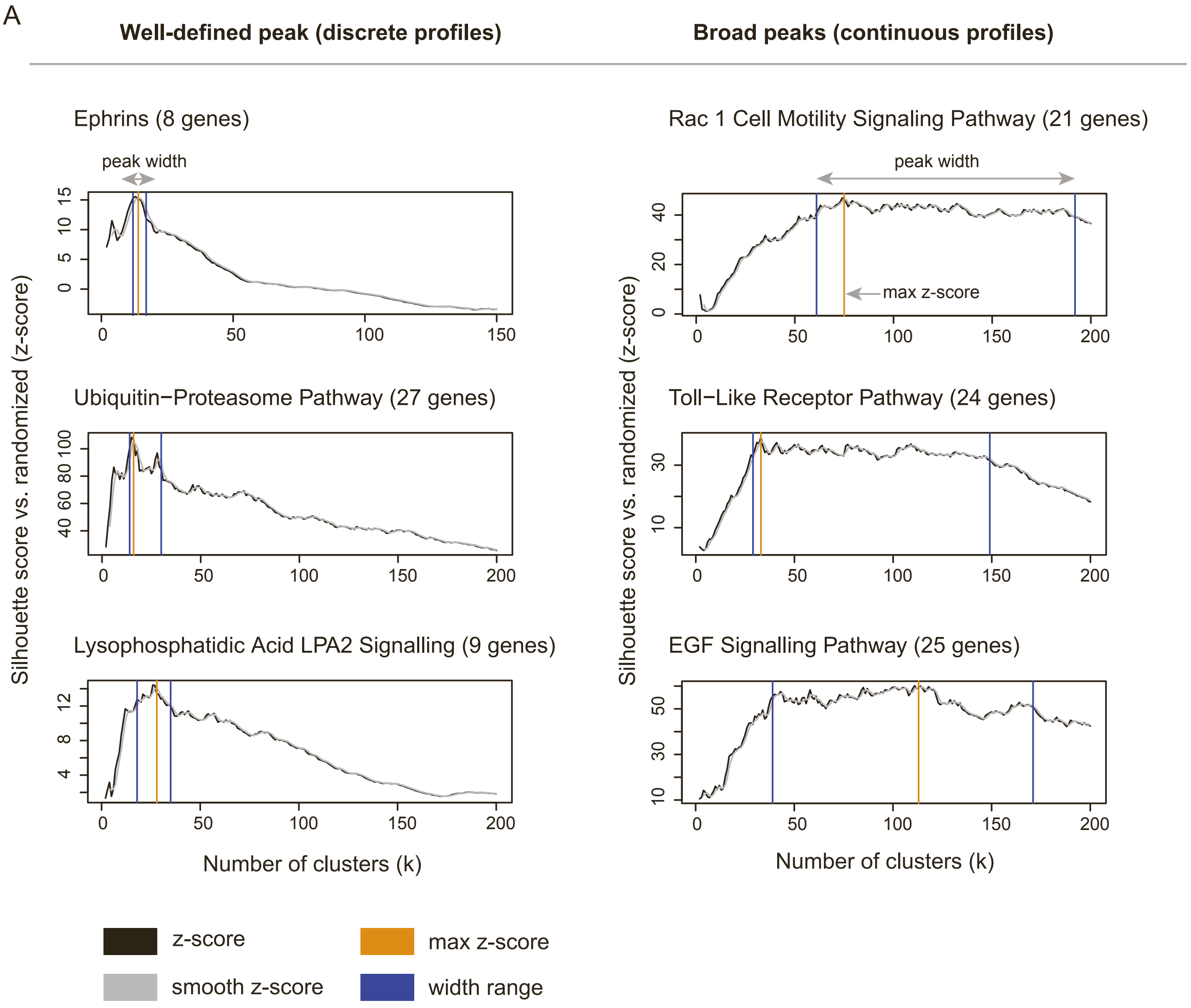

Figure S4. Silhouette analysis for other pathways (related to main figures 3 and 5).

Silhouette analysis of indicated pathways, as in Figure 3A and Figure 5A. A vertical yellow line indicates the value of k (the proposed number of clusters) with the maximum silhouette z-score. The peak width is shown with two blue vertical lines (the minimum and maximum values of *k* with a 10% difference from the maximum value). Finally, the relative peak width is calculated as the ratio of the peak width divided by the maximum number of *k* considered. We show three pathways with the lowest (left) and highest (right) relative width.

Supplementary Figure 5

A

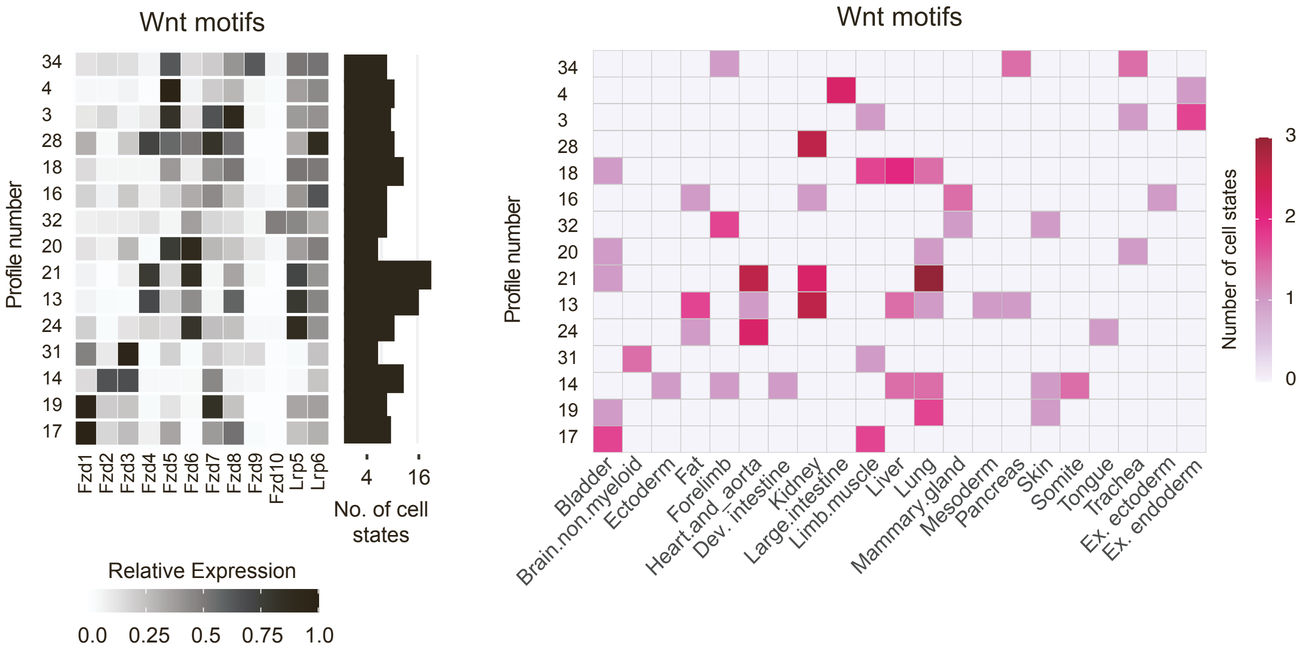

B

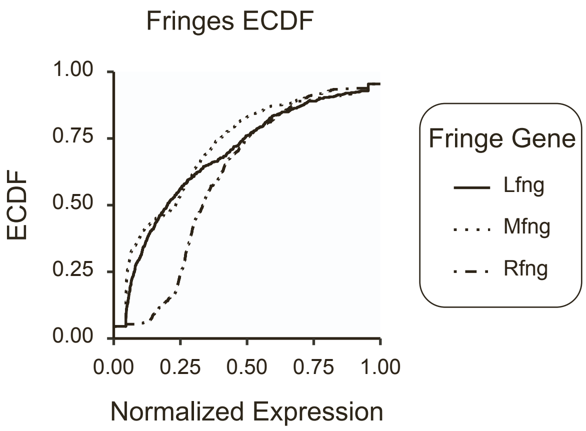

C

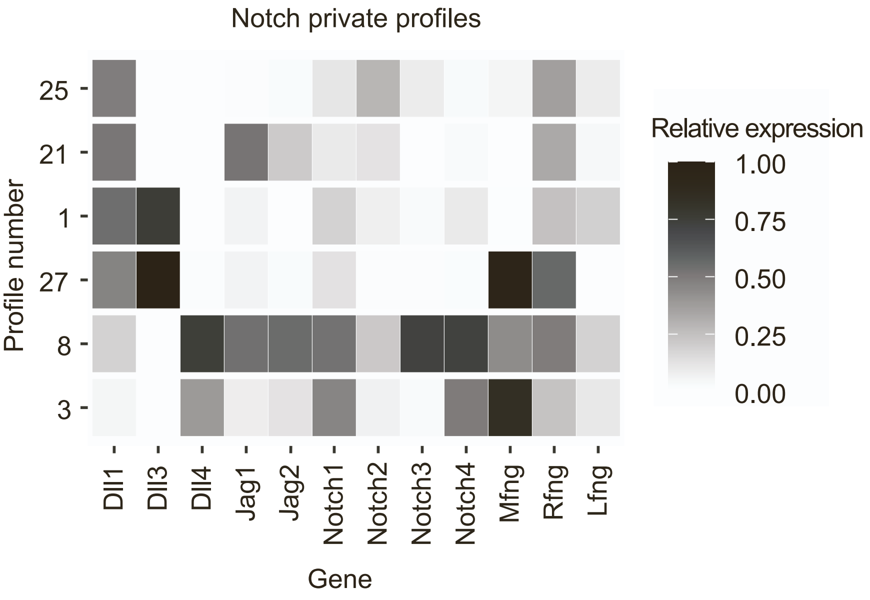

Figure S5. Motifs for Wnt and Notch pathways (related to main figures 4 and 5).

A. Motifs in the Wnt pathway and their distribution across tissues and organs, similar to Figure 4D, E.
B. Cumulative distribution histogram of Fringe gene expression across all cell states.
C. Notch pathway private profiles.

Supplementary Figure 6

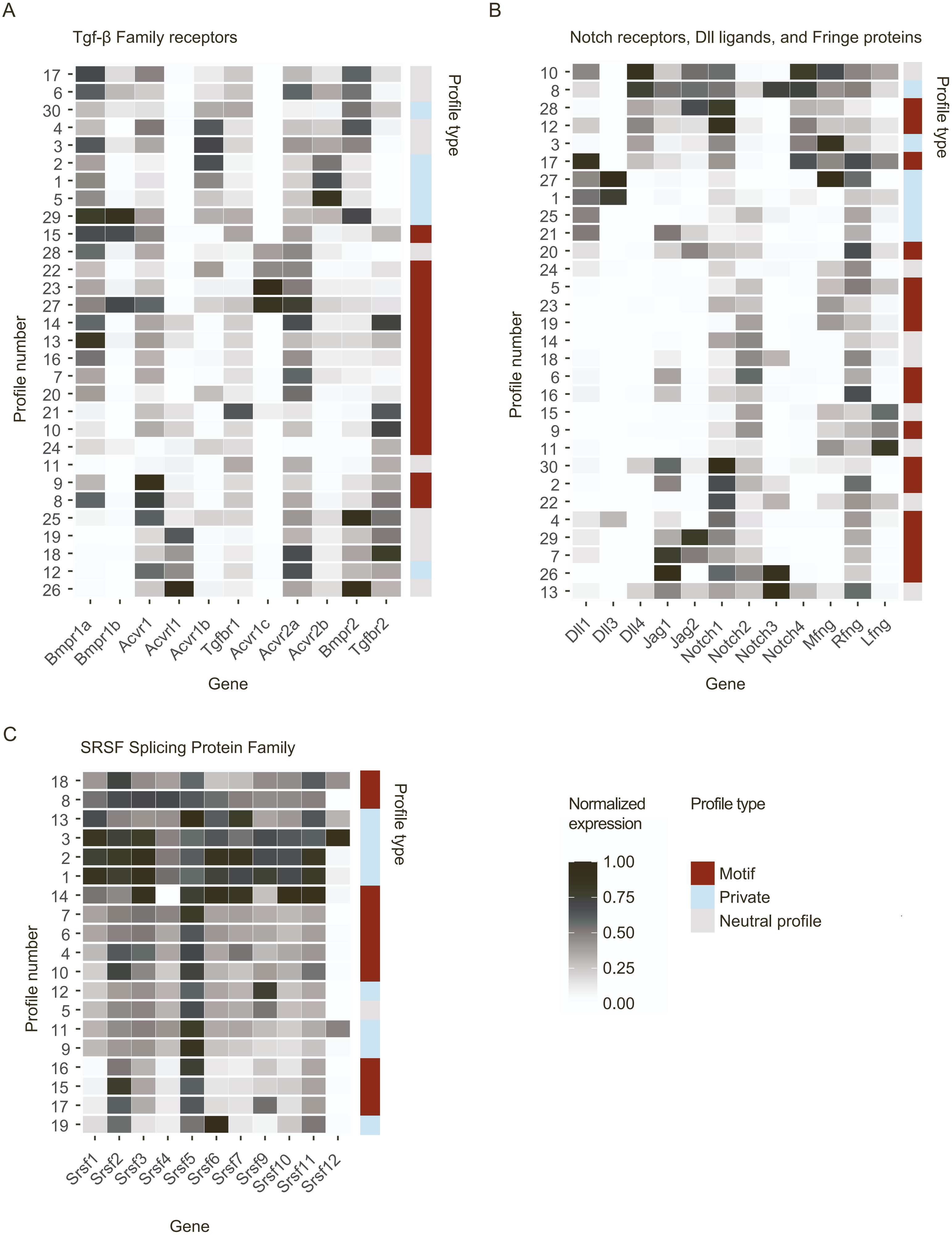

Figure S6. All profile classes for TGF-β, Notch, and SRSF (related to main figures 5 and 6).

All profiles are organized by similarity and annotated by their profile number and profile type (motif, private, or neutral) for TGF-β (A), Notch (B), and SRSF (C).

Supplemental data S1

Global cell type dendrogram, as shown in Figure 3C, along with a heatmap of the projection of each cell type on the top 20 principal components computed from the whole-transcriptome space.

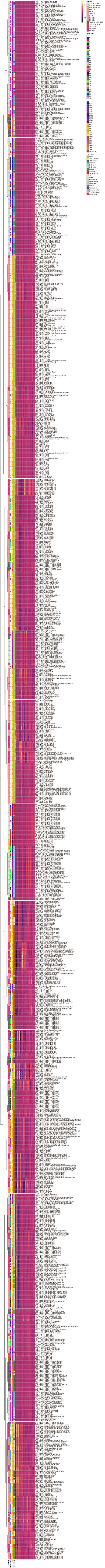

Supplemental data S2

Analysis of pathways shown in Figure 5C. Each pathway has plots displayed on two pages. The first page has plots displayed for pathways as done for TGF-beta in Figures 3 and 4. The second page shows UMAPs highlighting each pathway motif (colored dots) and displays the dispersion score for the motif.

BCR Signaling Pathway (k_opt = 110)

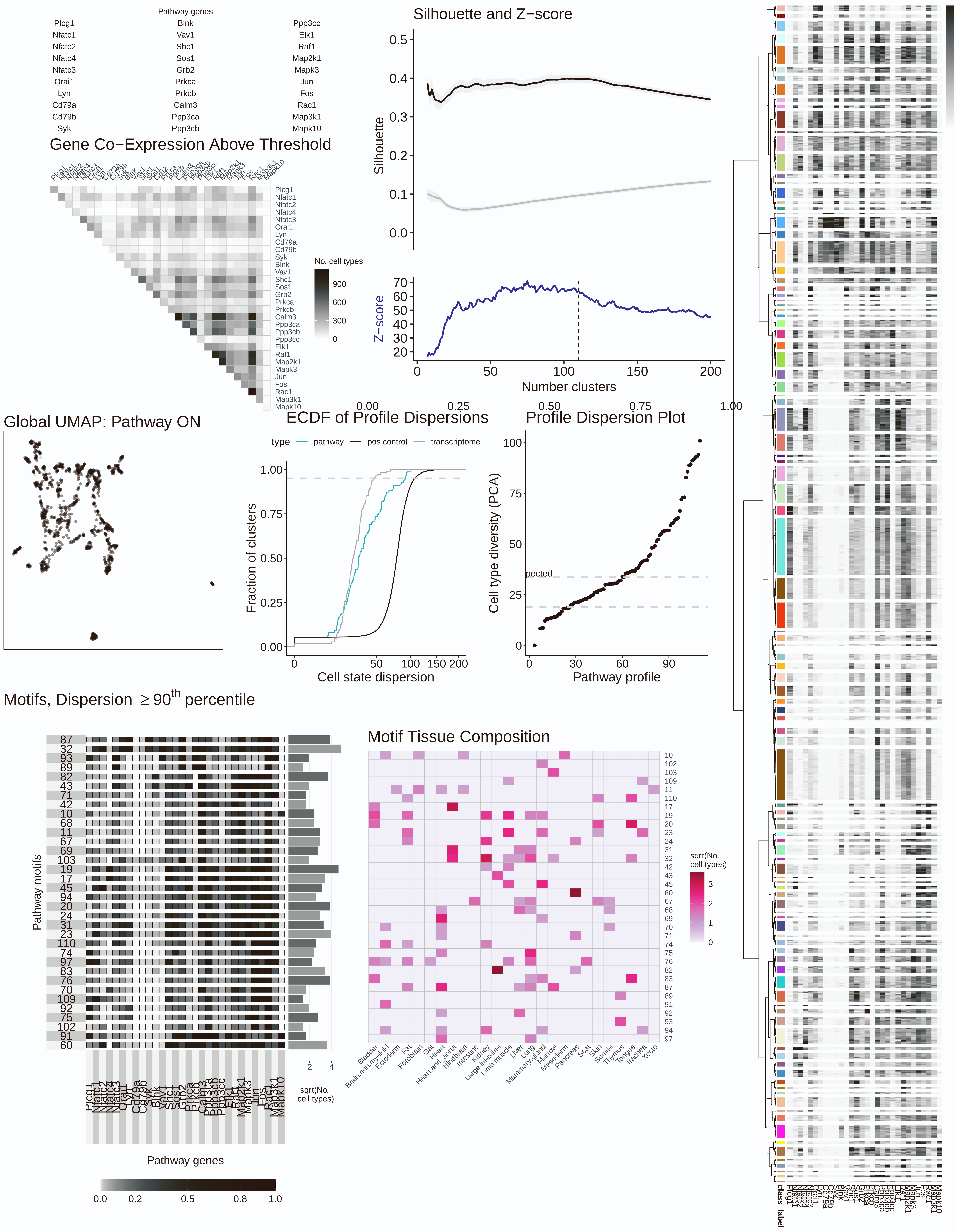

BCR Signaling Pathway (k_opt = 110)

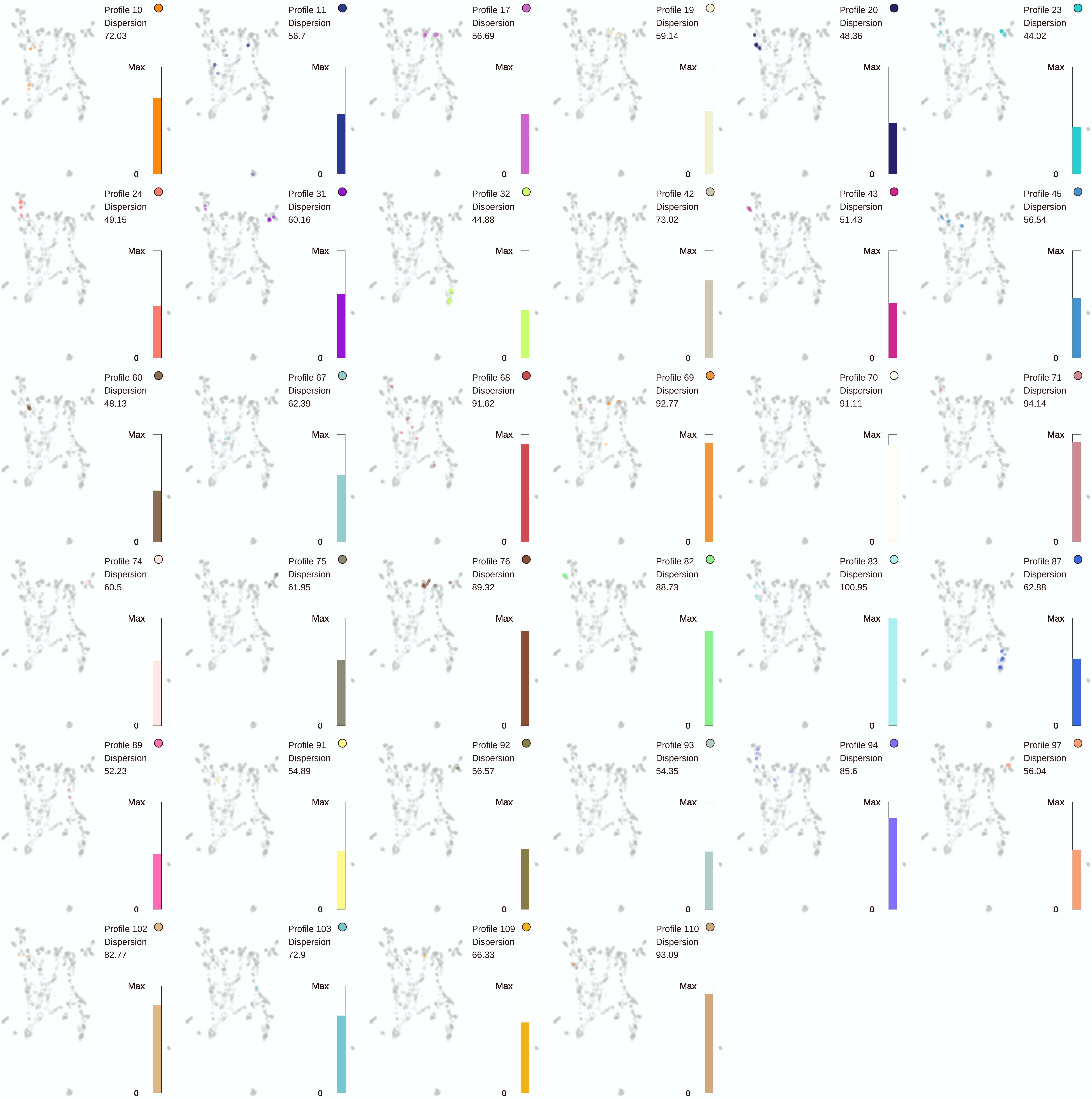

CD40L Signalling Pathway (k_opt = 67)

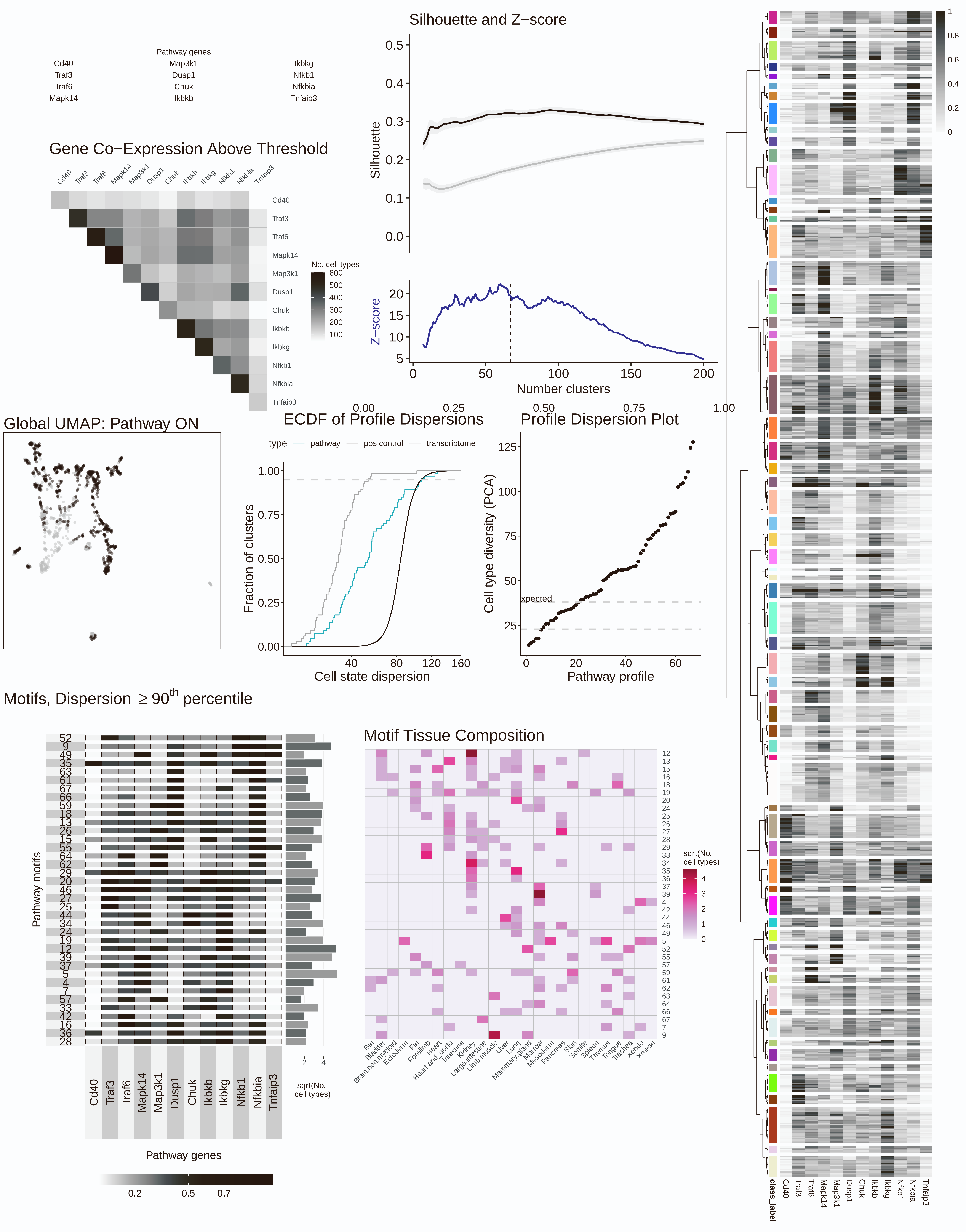

CD40L Signalling Pathway (k_opt = 67)

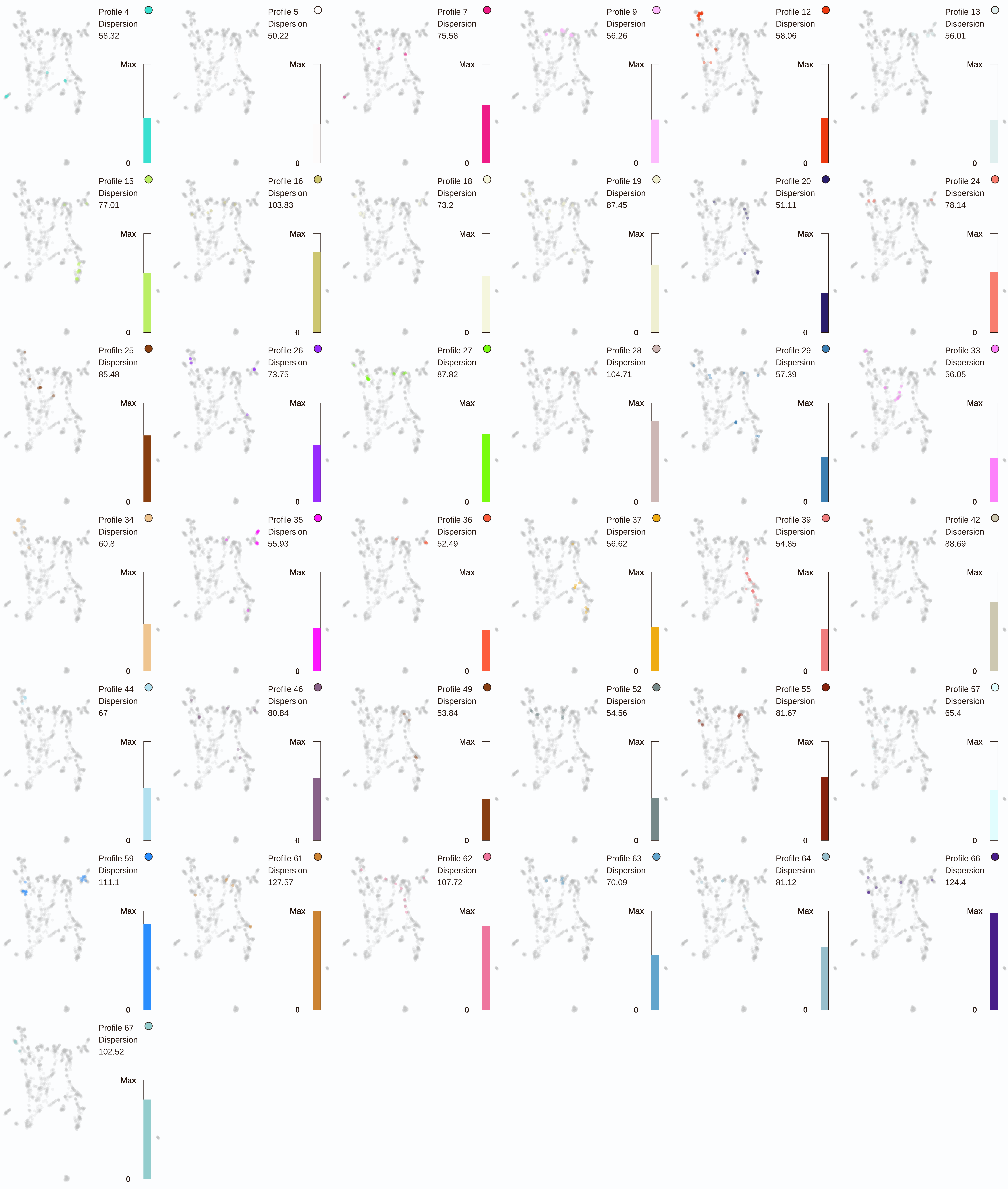

D4−GDI Signaling Pathway (k_opt = 77)

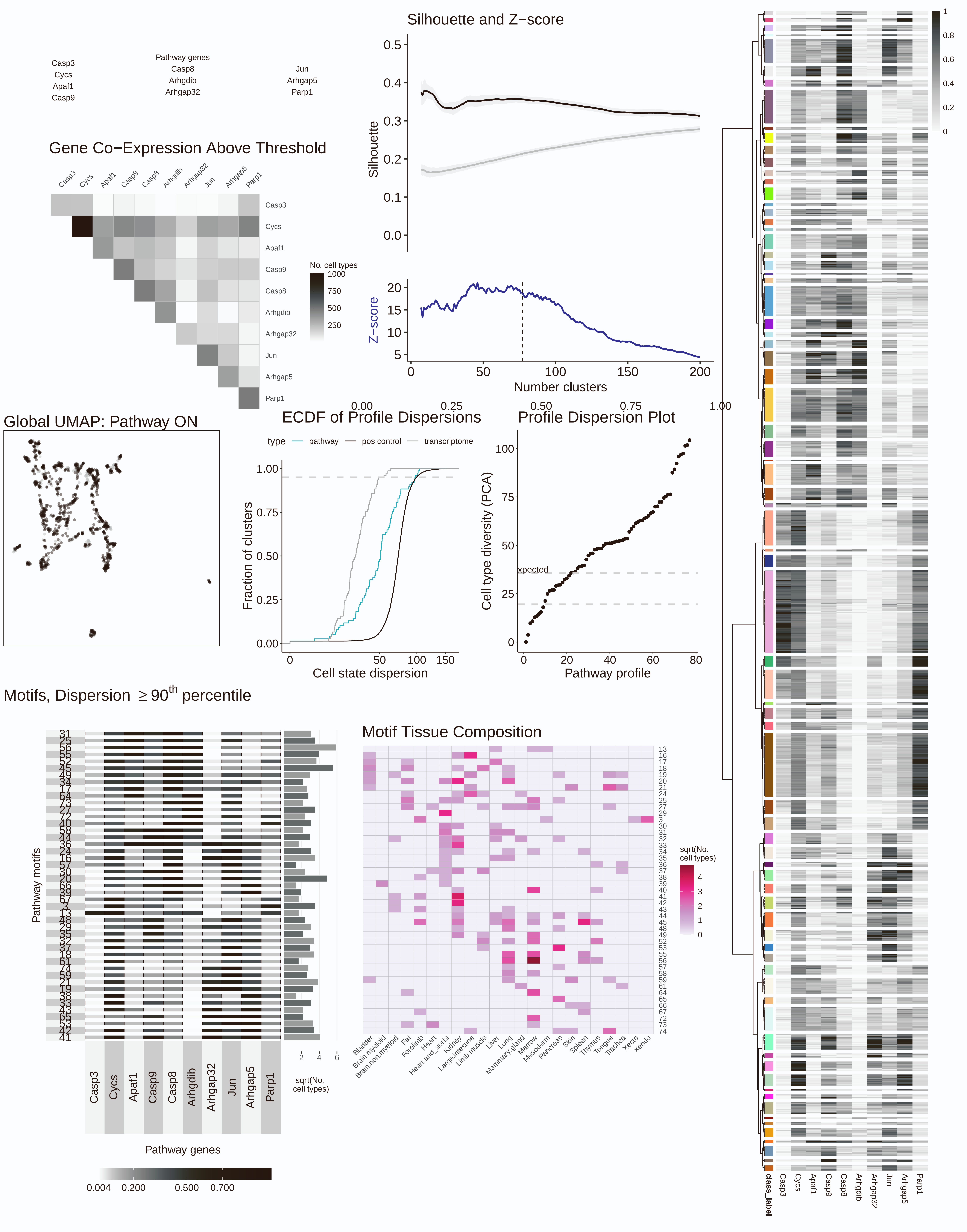

D4−GDI Signaling Pathway (k_opt = 77)

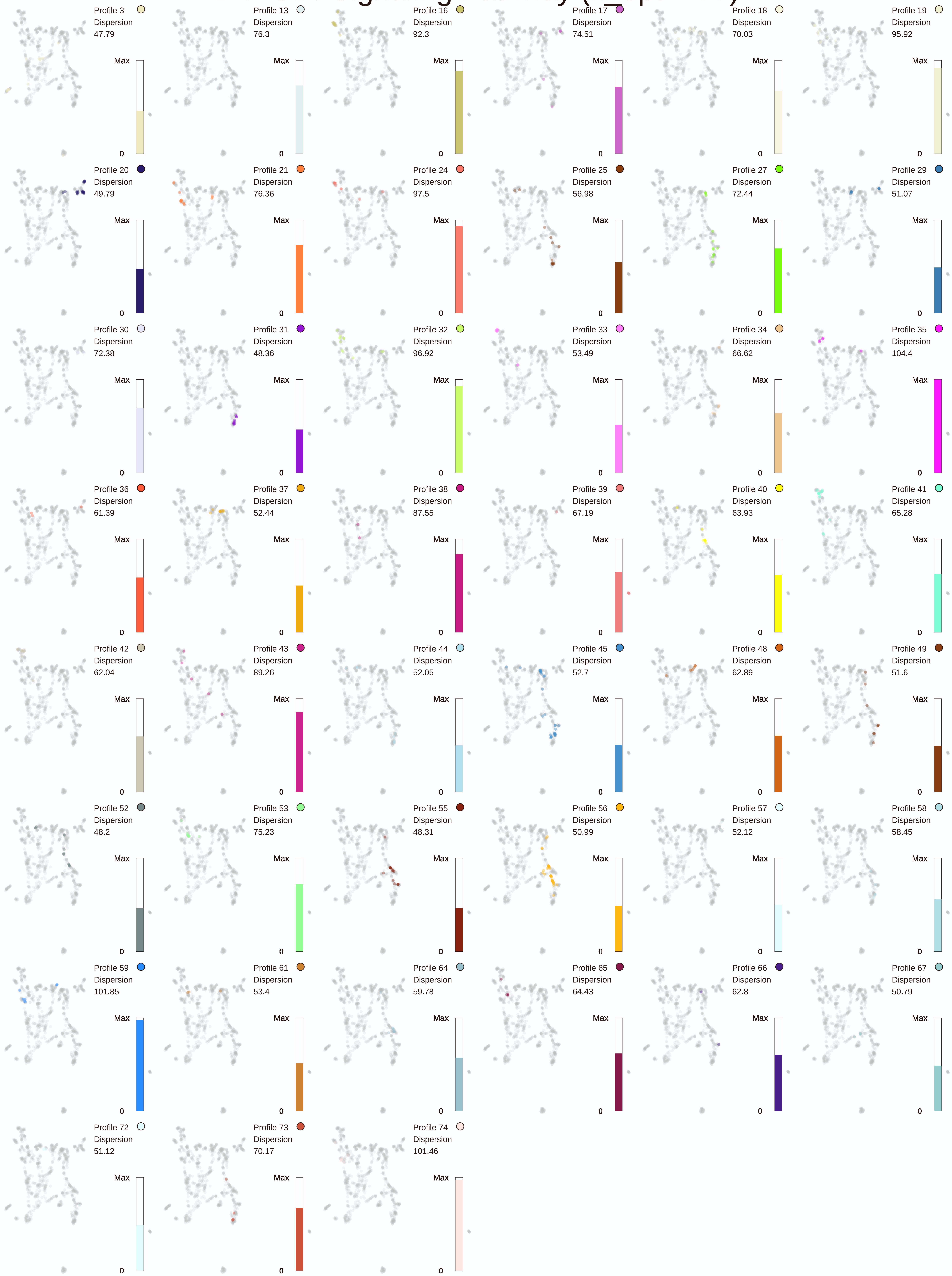

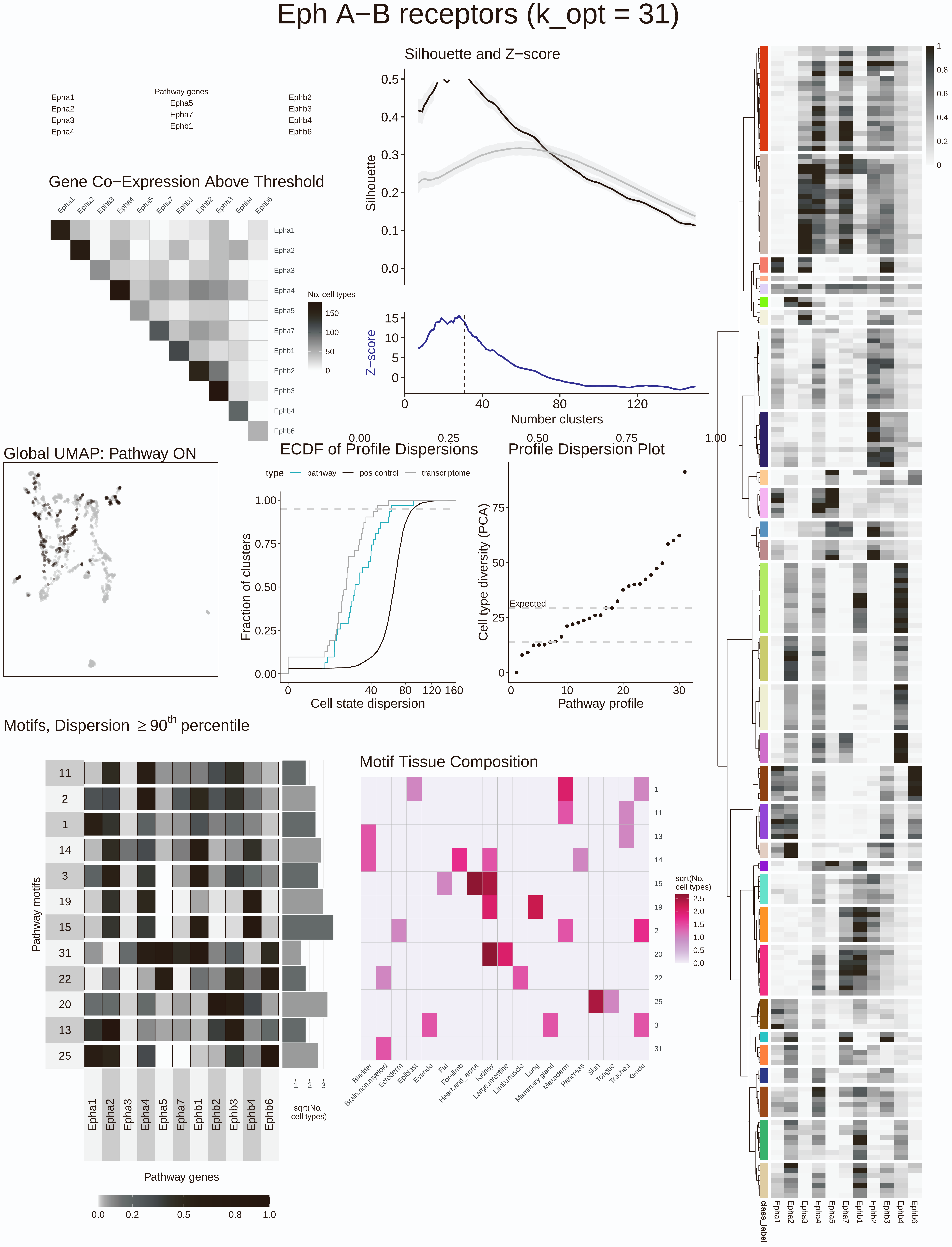

Eph A−B receptors (k_opt = 31)
Pathway genes
Epha1
Epha2
Epha3
Epha4
Epha5
Epha7
Ephb1
Ephb2
Ephb3
Ephb4
Ephb6
Gene Co−Expression Above Threshold
No. cell types
Silhouette and Z−score
Silhouette
Z−score
Number clusters
Global UMAP: Pathway ON
ECDF of Profile Dispersions
type
pathway
pos control
transcriptome
Fraction of clusters
Cell state dispersion
Profile Dispersion Plot
Cell type diversity (PCA)
Expected
Pathway profile
Motifs, Dispersion ≥ 90th percentile
Pathway motifs
sqrt(No. cell types)
Motif Tissue Composition
Bladder
Brain.non.myeloid
Ectoderm
Epiblast
Evendo
Fat
Forelimb
Heart.and_aorta
Kidney
Large.intestine
Limb.muscle
Lung
Mammary.gland
Mesoderm
Pancreas
Skin
Tongue
Trachea
Xendo
class_label

Eph A−B receptors (k_opt = 31)

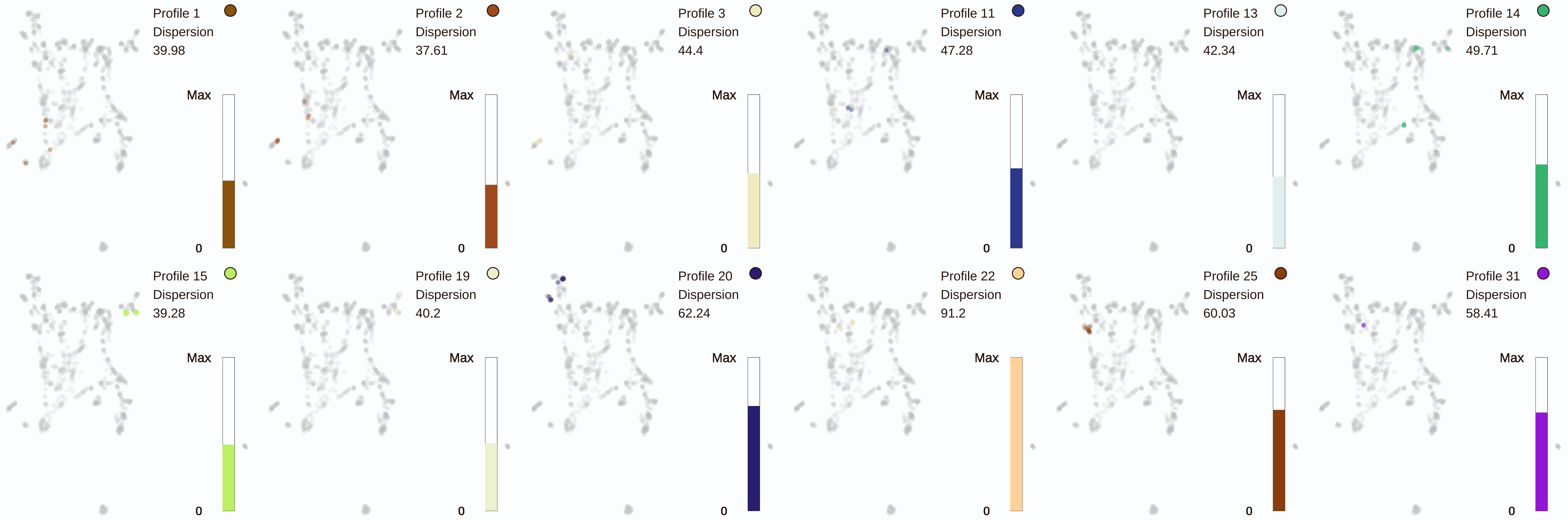

Eph receptors and ligands (k_opt = 52)

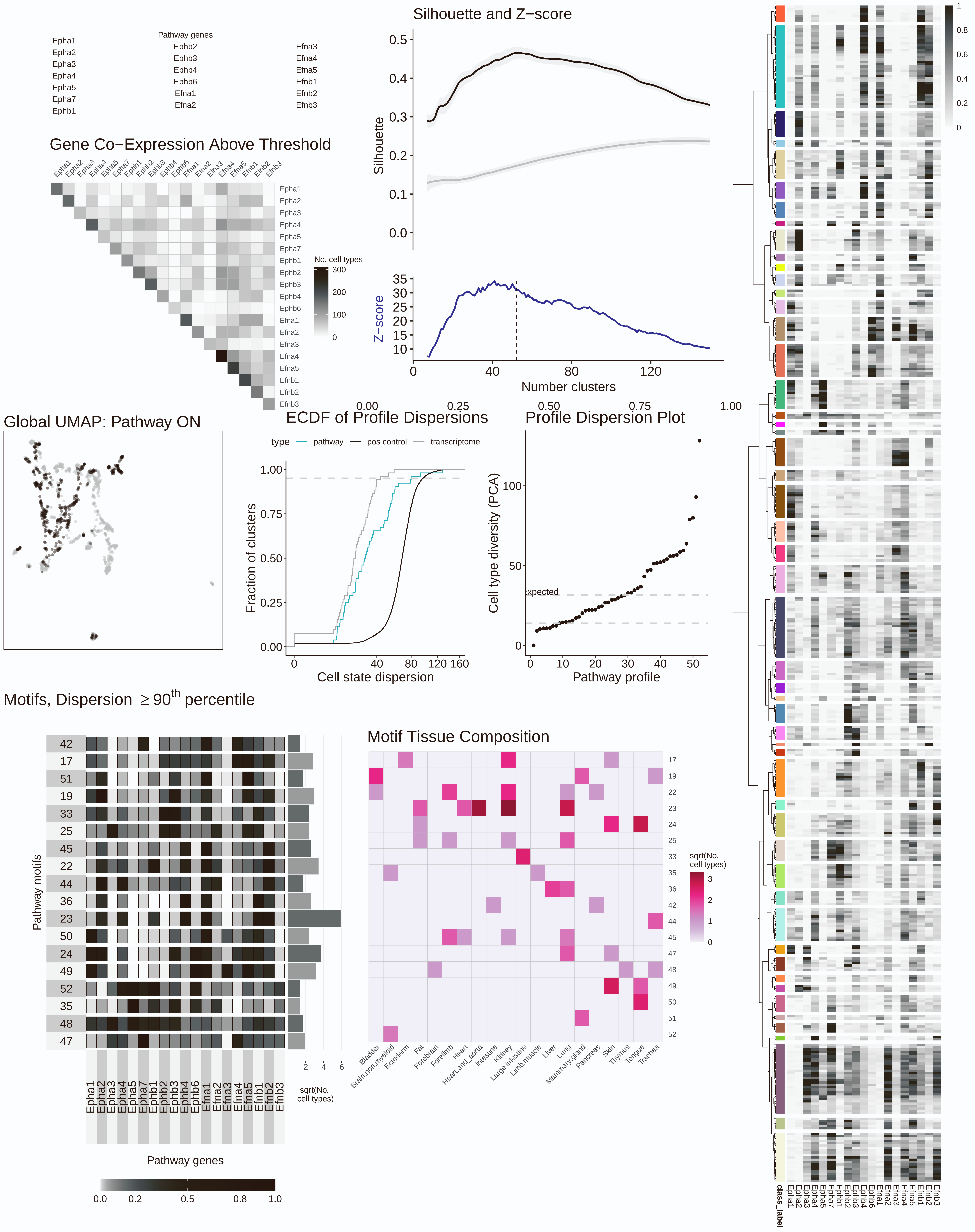

Eph receptors and ligands (k_opt = 52)

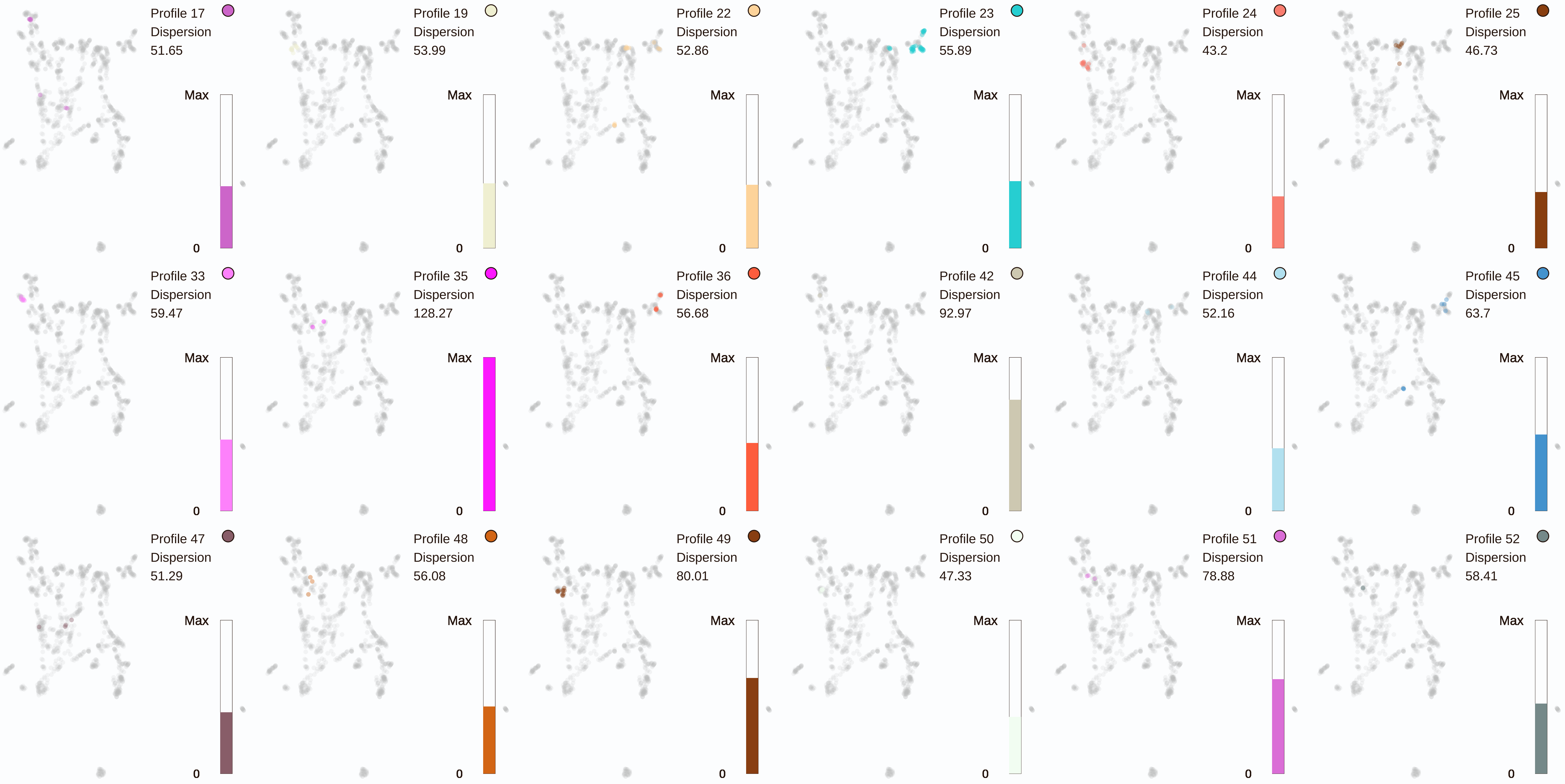

Eph_I (k_opt = 16)

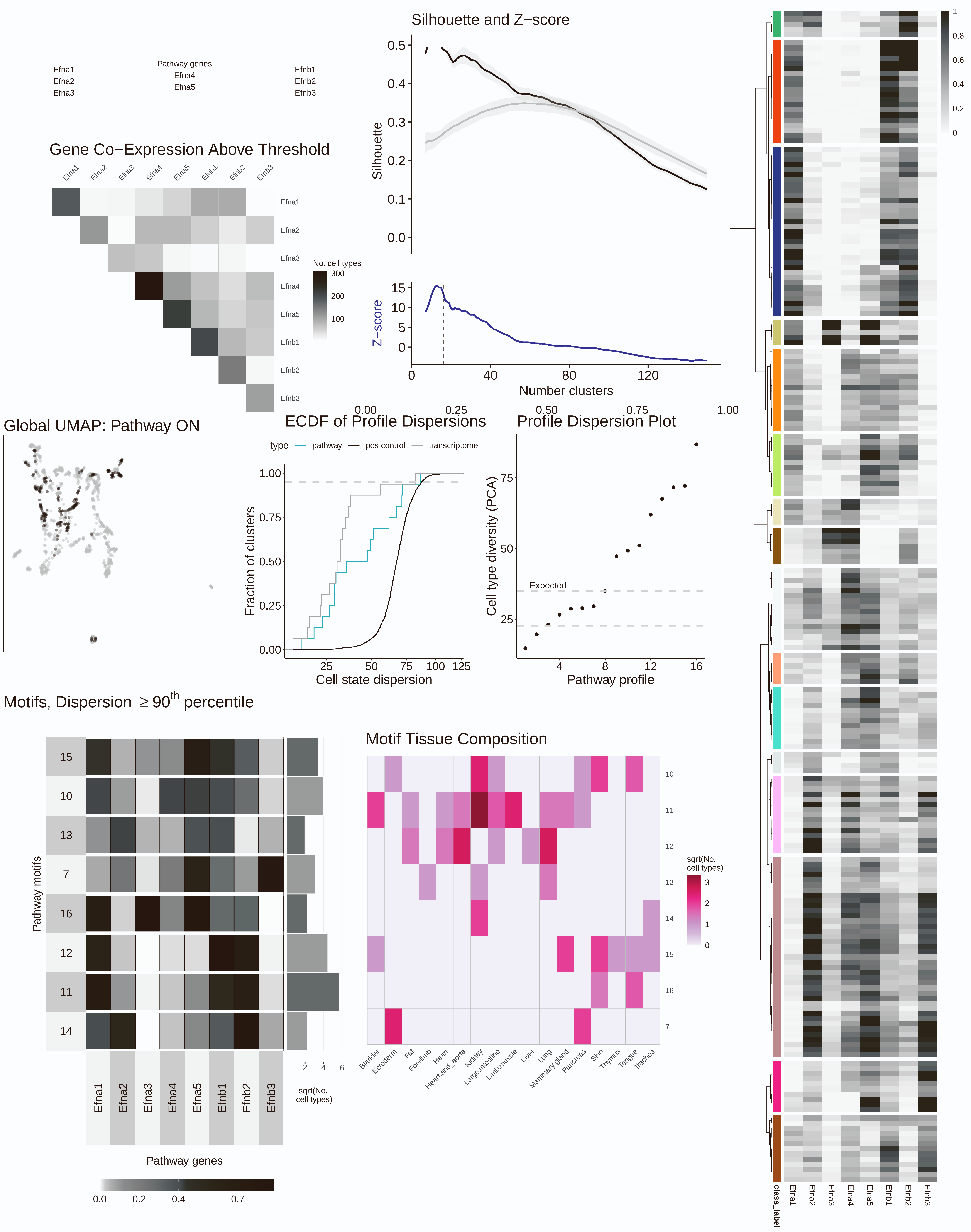

Silhouette and Z−score
Silhouette
Z−score
Number clusters
Pathway genes
Efna1
Efna2
Efna3
Efna4
Efna5
Efnb1
Efnb2
Efnb3
Gene Co−Expression Above Threshold
No. cell types
Global UMAP: Pathway ON
ECDF of Profile Dispersions
type
pathway
pos control
transcriptome
Fraction of clusters
Cell state dispersion
Profile Dispersion Plot
Expected
Cell type diversity (PCA)
Pathway profile
Motifs, Dispersion ≥ 90th percentile
Pathway motifs
sqrt(No. cell types)
Motif Tissue Composition
Bladder
Ectoderm
Fat
Forelimb
Heart
Heart_and_aorta
Kidney
Large.intestine
Limb.muscle
Liver
Lung
Mammary.gland
Pancreas
Skin
Thymus
Tongue
Trachea
class_label

Eph_I (k_opt = 16)

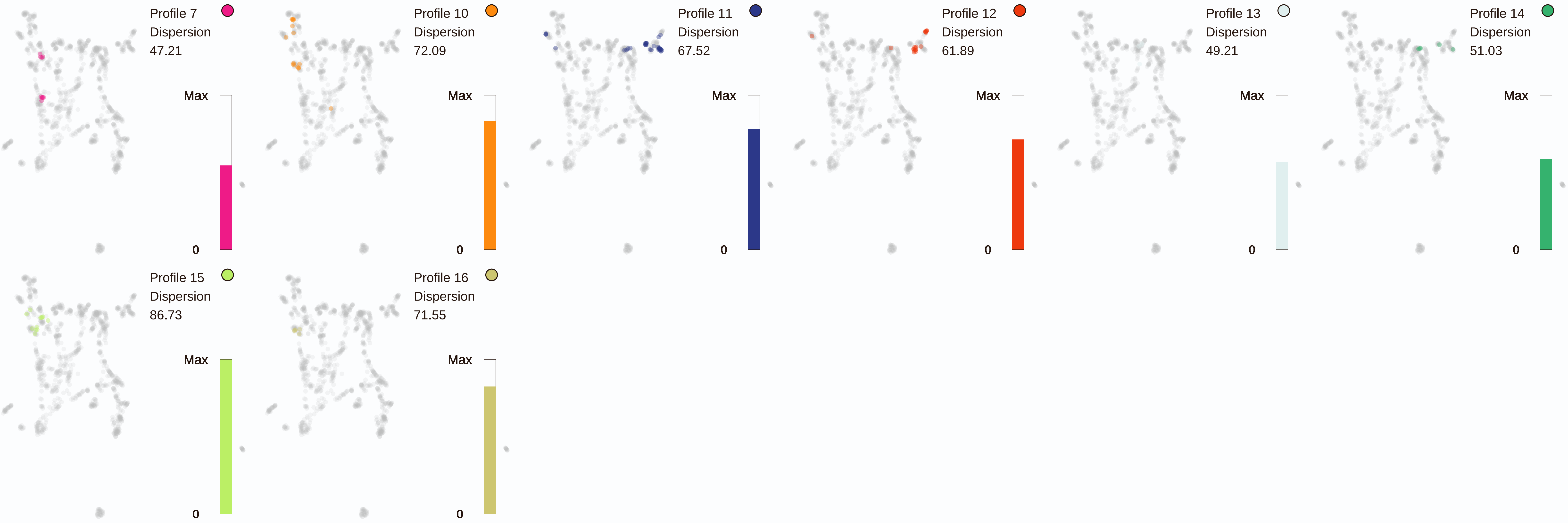
Profile 7
Dispersion
47.21
Max
0
Profile 10
Dispersion
72.09
Max
0
Profile 11
Dispersion
67.52
Max
0
Profile 12
Dispersion
61.89
Max
0
Profile 13
Dispersion
49.21
Max
0
Profile 14
Dispersion
51.03
Max
0
Profile 15
Dispersion
86.73
Max
0
Profile 16
Dispersion
71.55
Max
0

FGF cell signaling proteins (k_opt = 80)

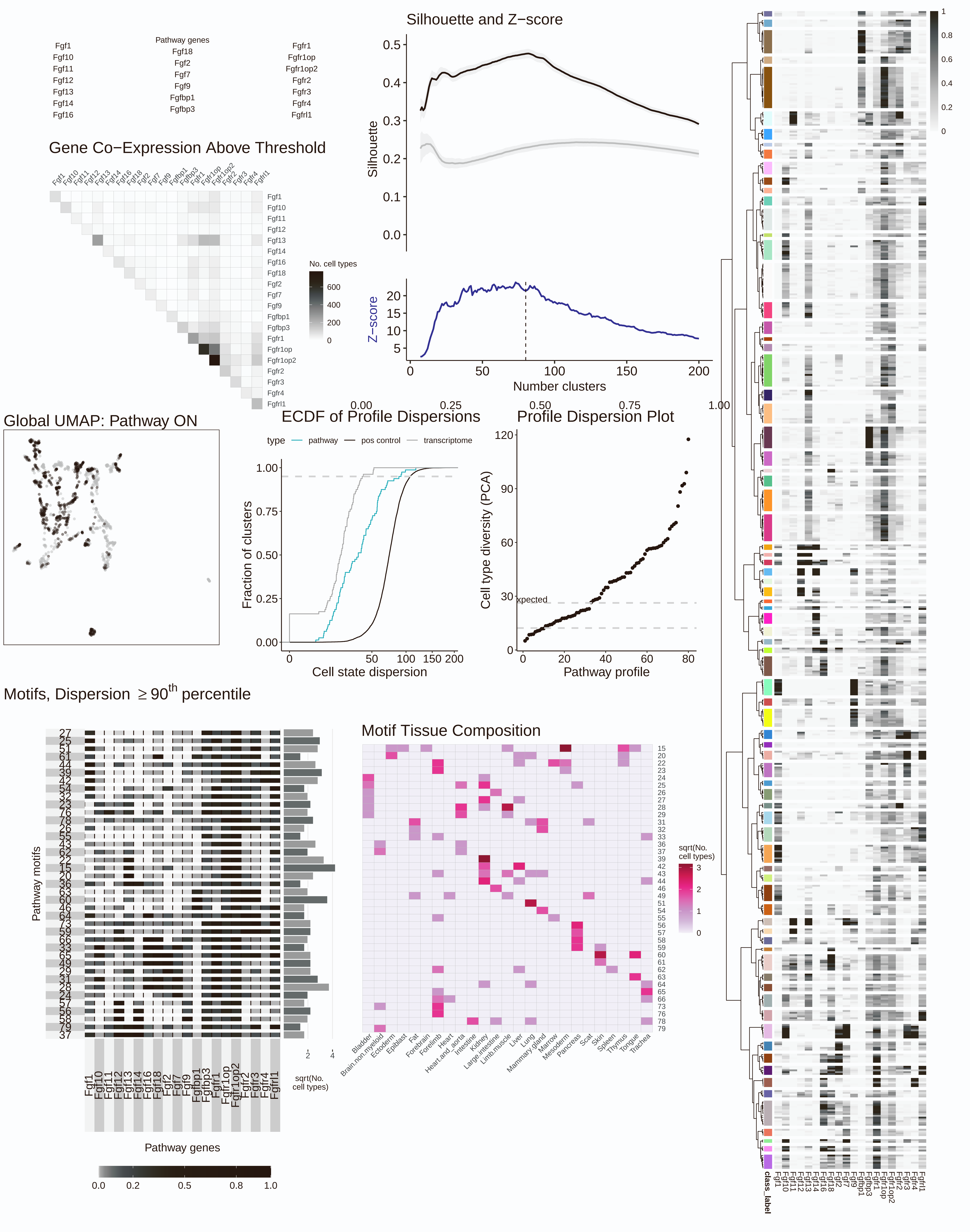

FGF cell signaling proteins (k_opt = 80)

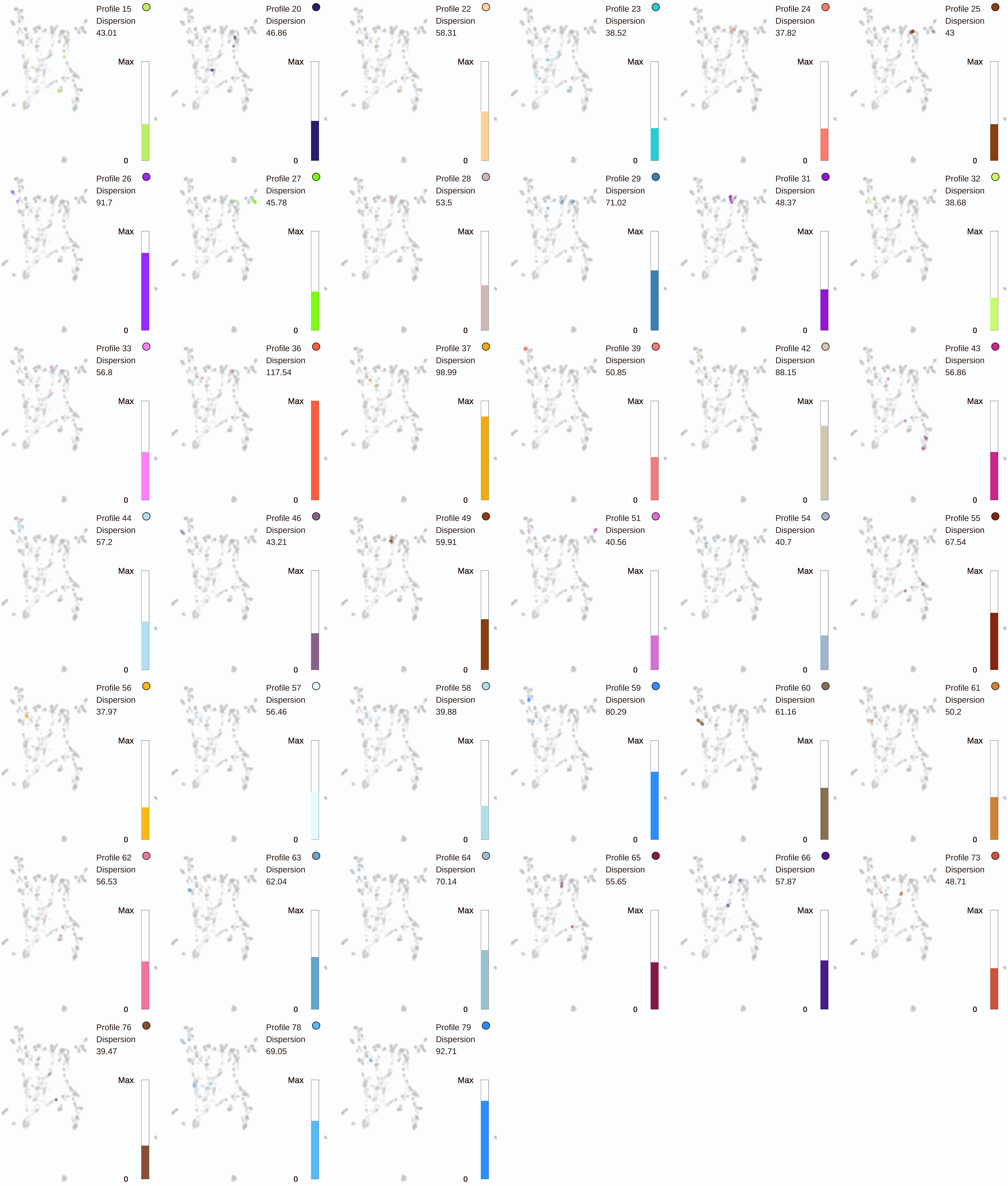

Frizzled and Lrp5 6 receptors for Wnt B Catenin Signaling (k_opt = 37)

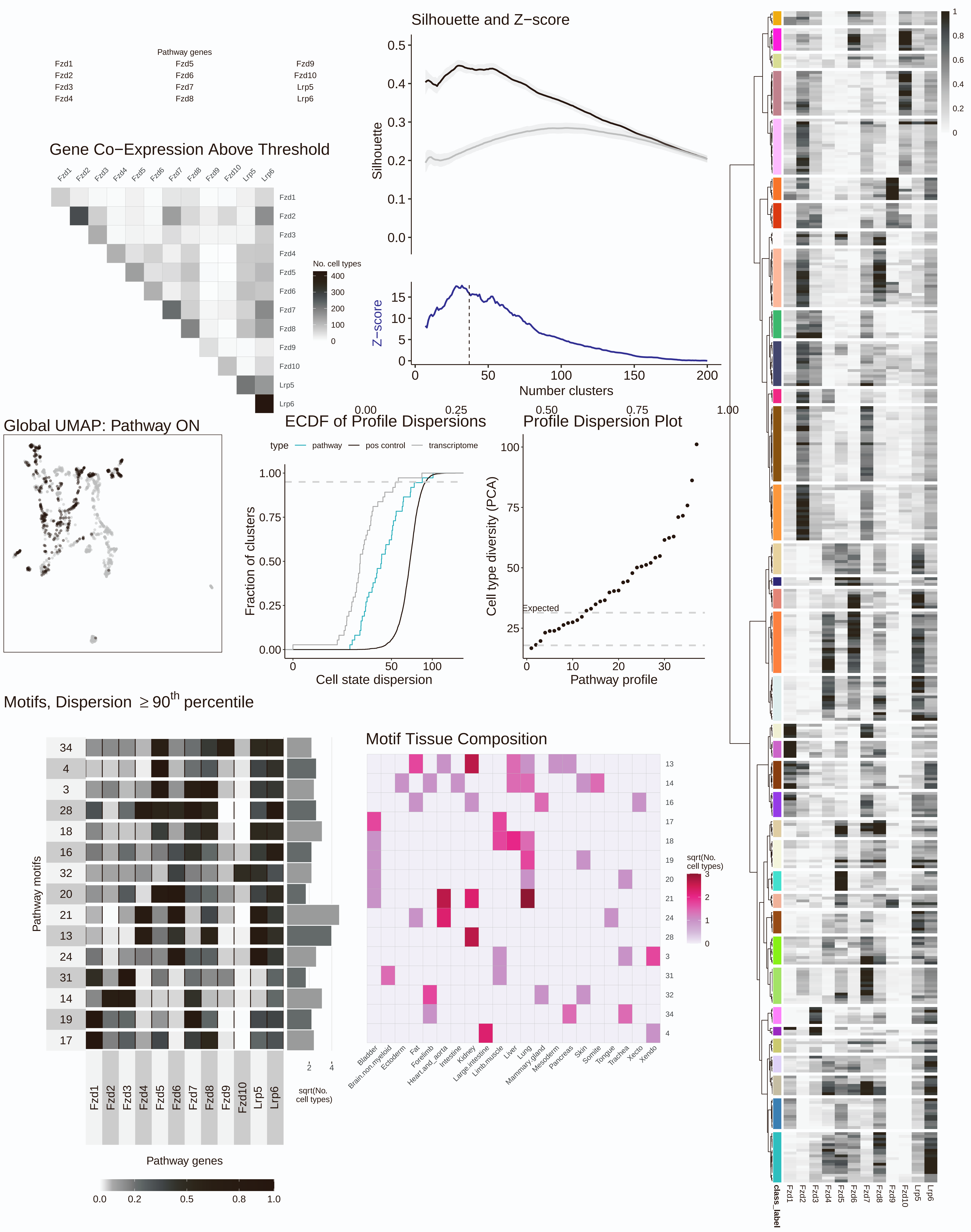

Frizzled and Lrp5 6 receptors for Wnt B Catenin Signaling (k_opt = 37)

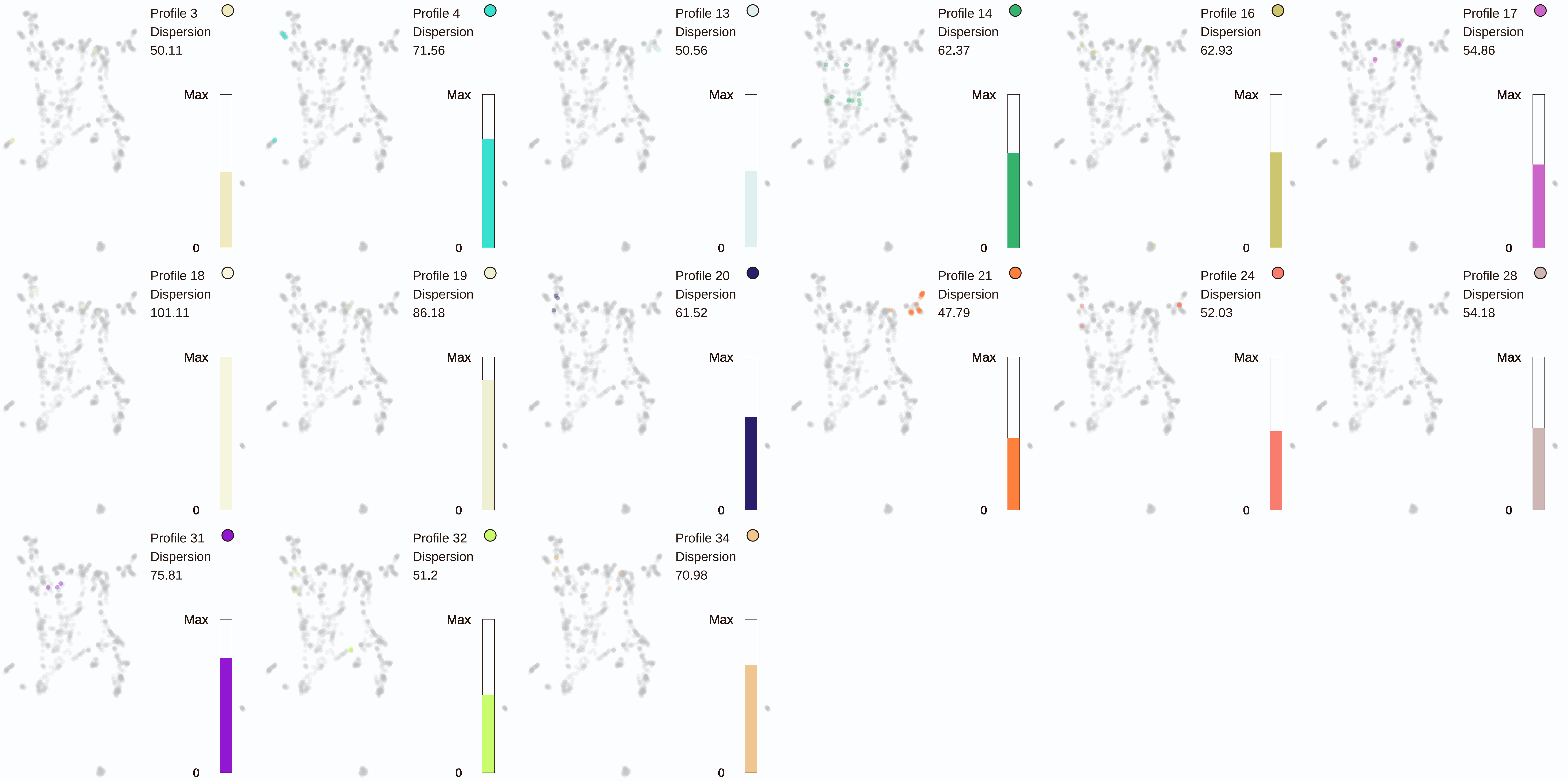

acellular Signalling Through Adenosine Receptor A2a and Adenosine (k_opt =

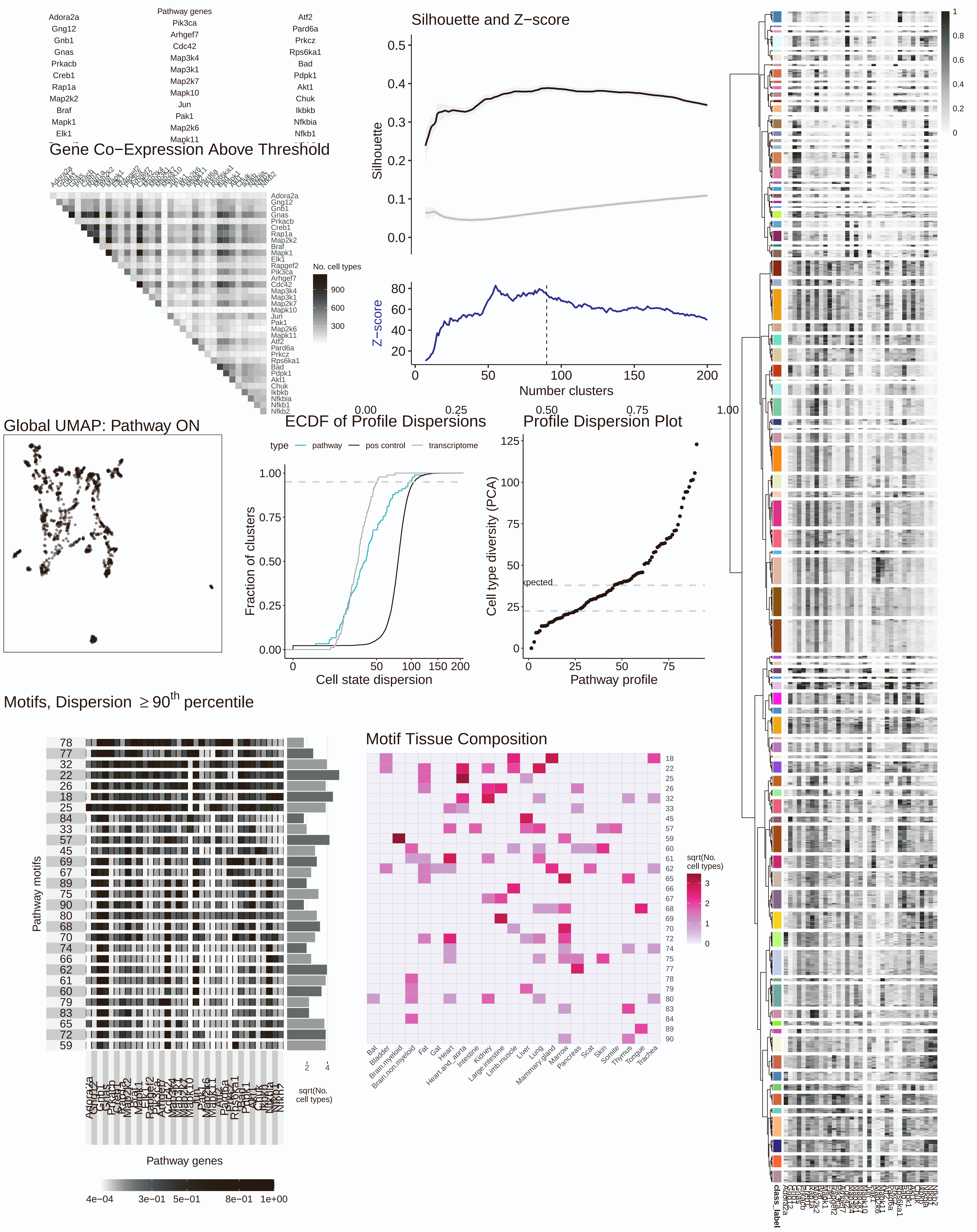

cellular Signalling Through Adenosine Receptor A2a and Adenosine (k_opt =

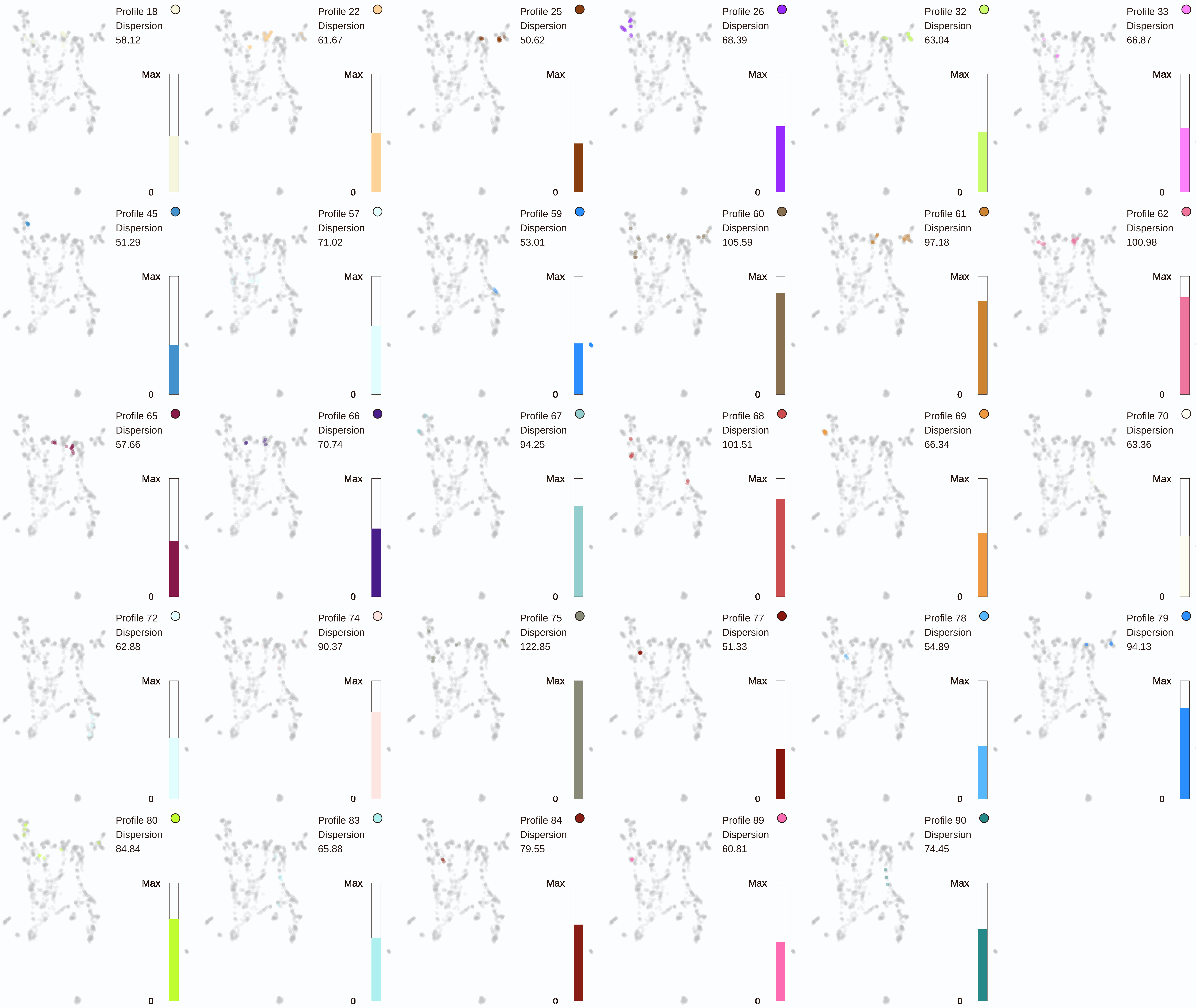

Ion Channels and Their Functional Role in Vascular Endothelium (k_opt = 32)

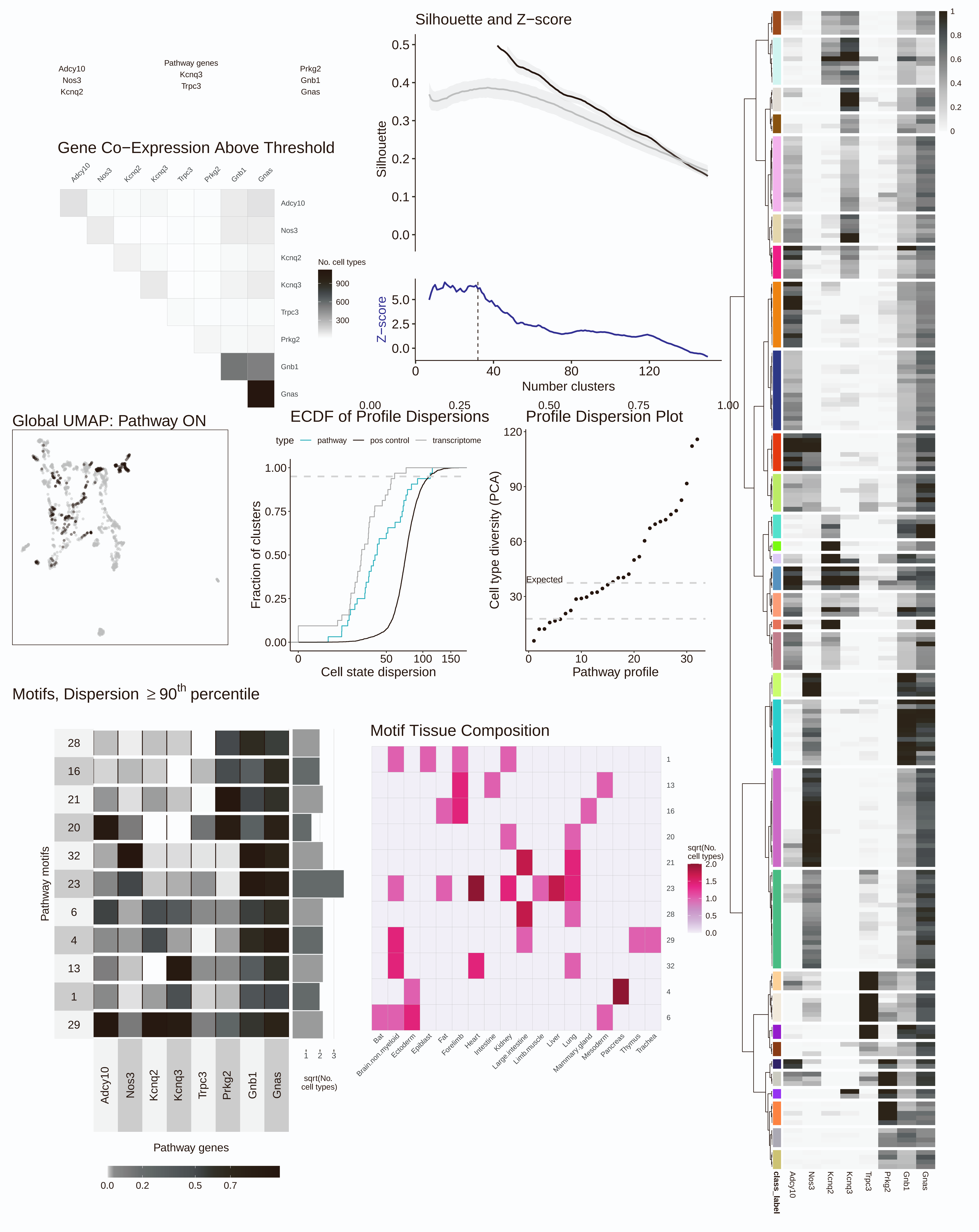

Ion Channels and Their Functional Role in Vascular Endothelium (k_opt = 32)

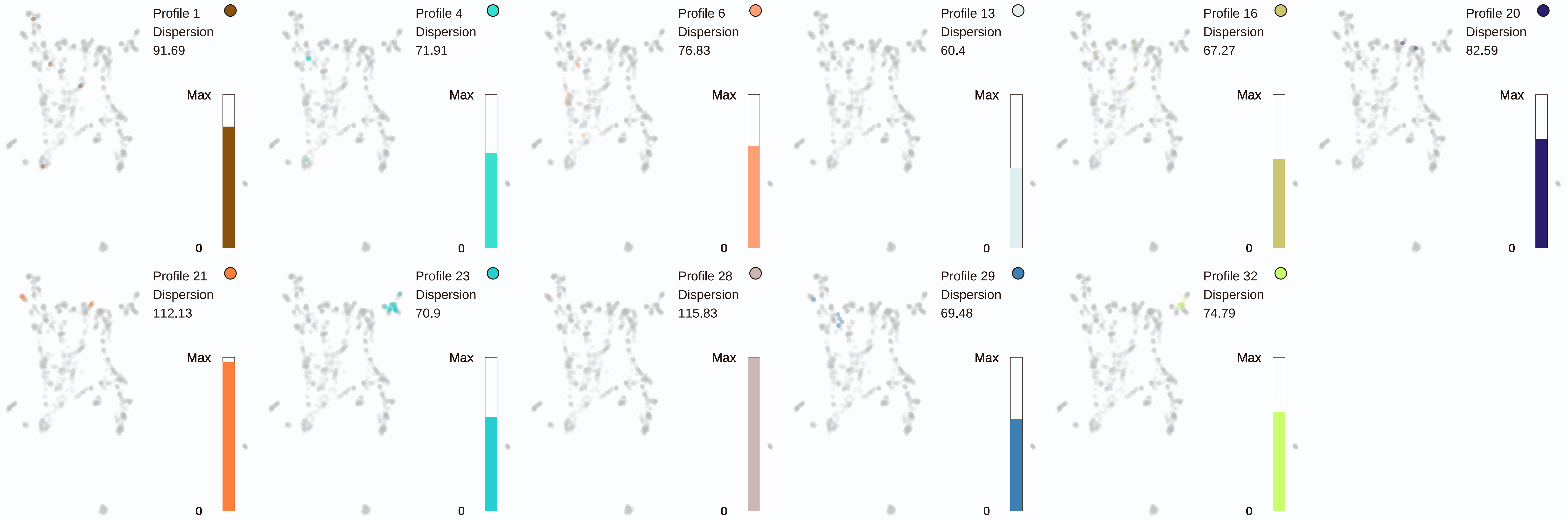

Leucine Stimulation on Insulin Signaling (k_opt = 34)

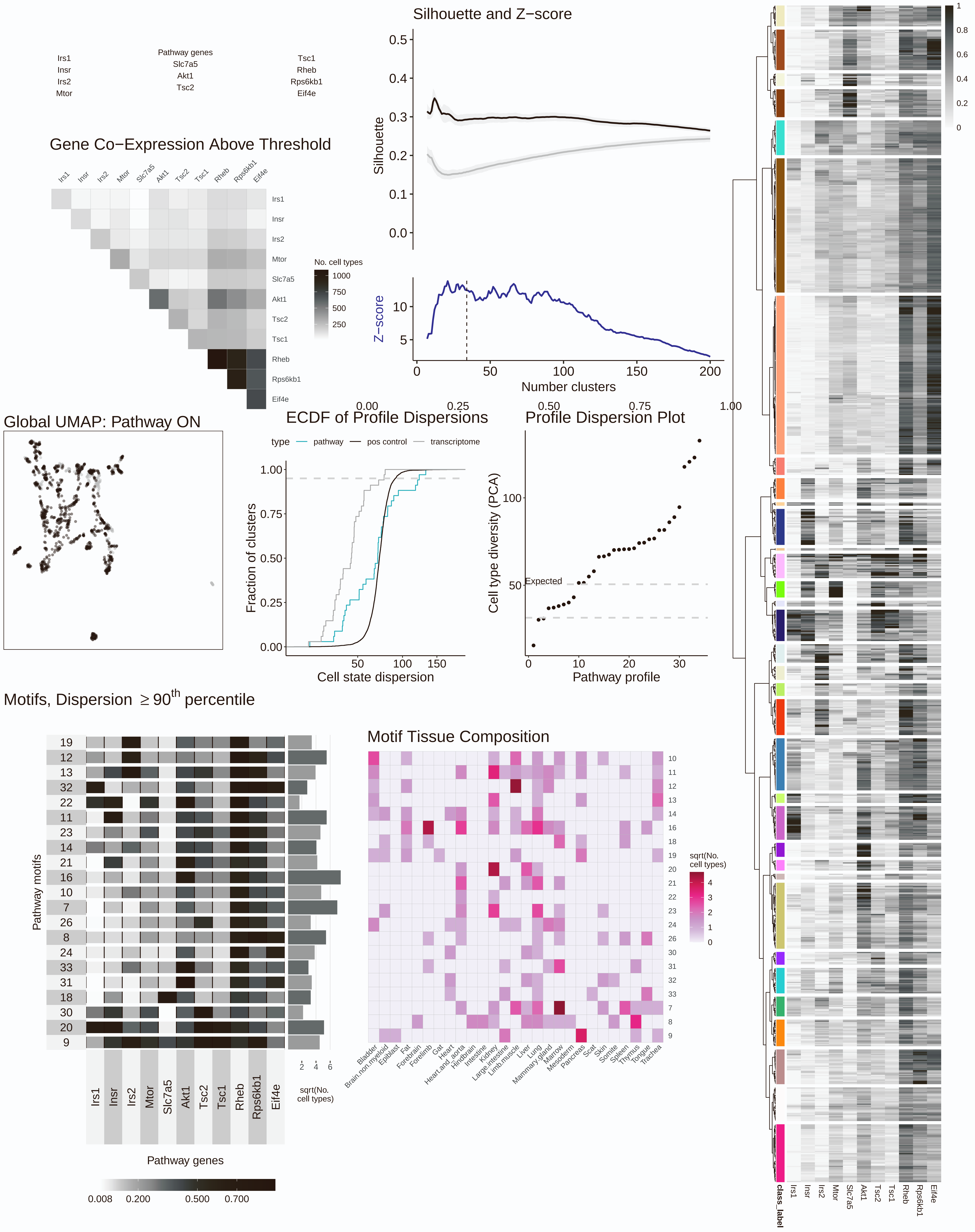

Leucine Stimulation on Insulin Signaling (k_opt = 34)

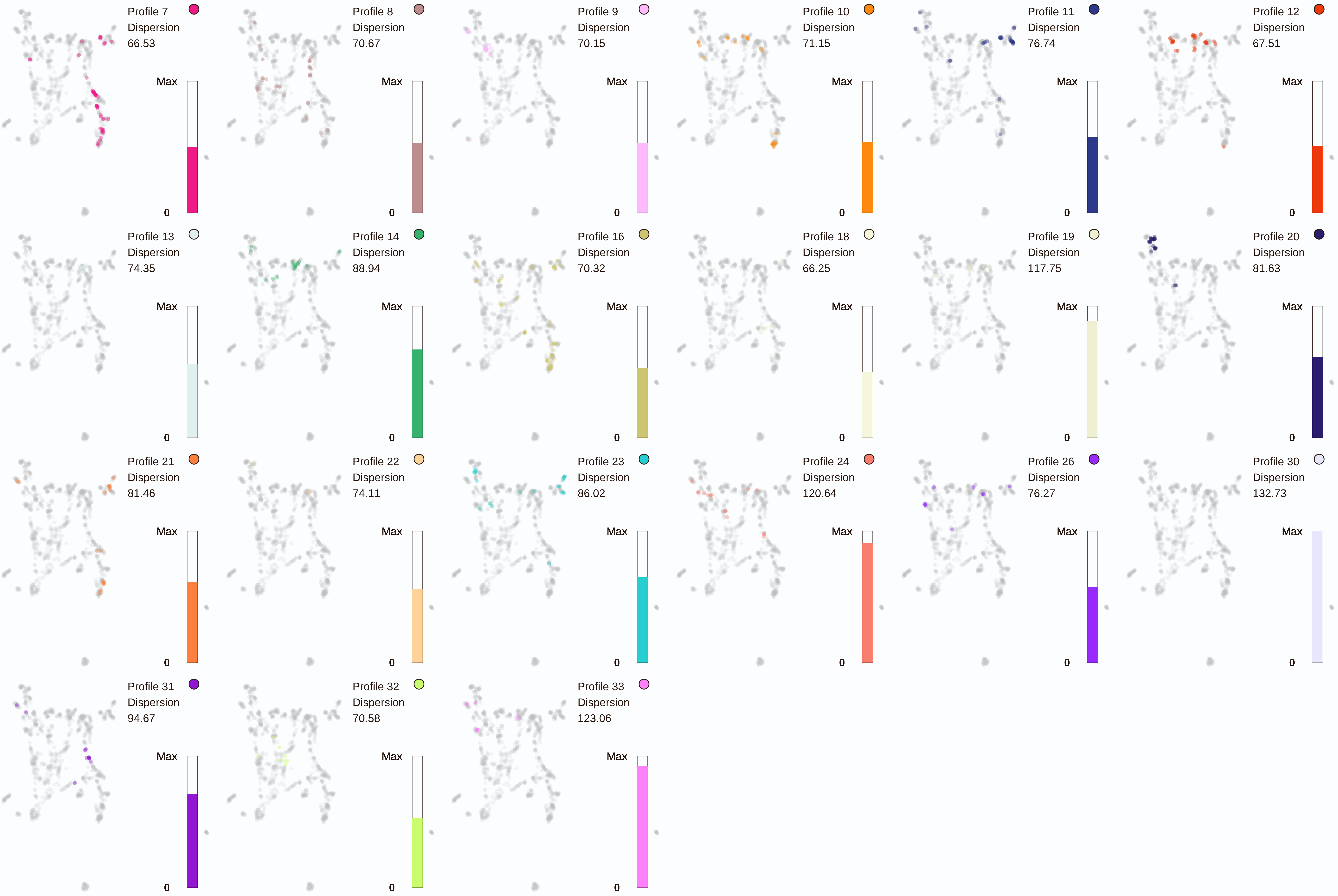

LPS and Citrate Signaling and Inflammation (k_opt = 112)

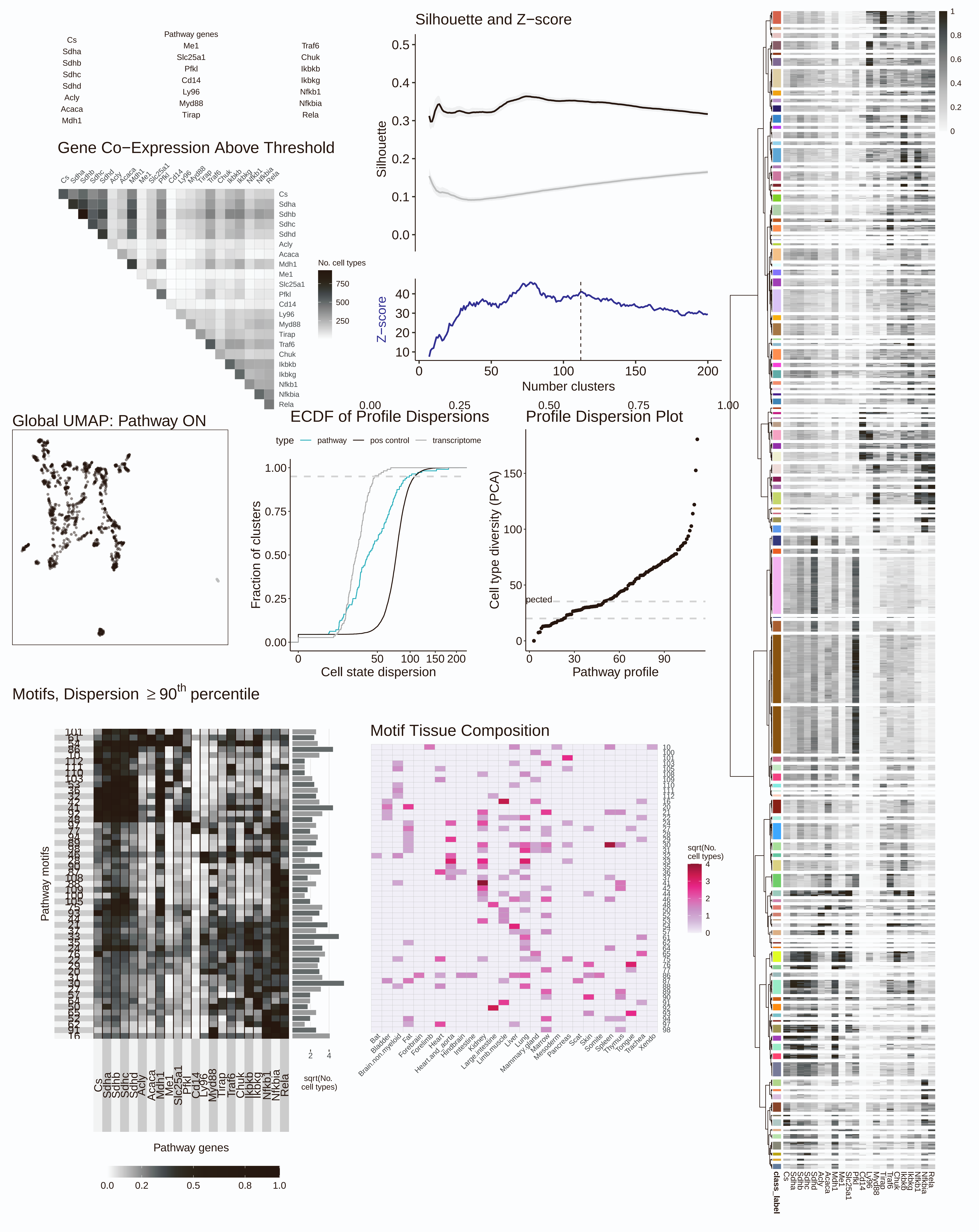

LPS and Citrate Signaling and Inflammation (k_opt = 112)

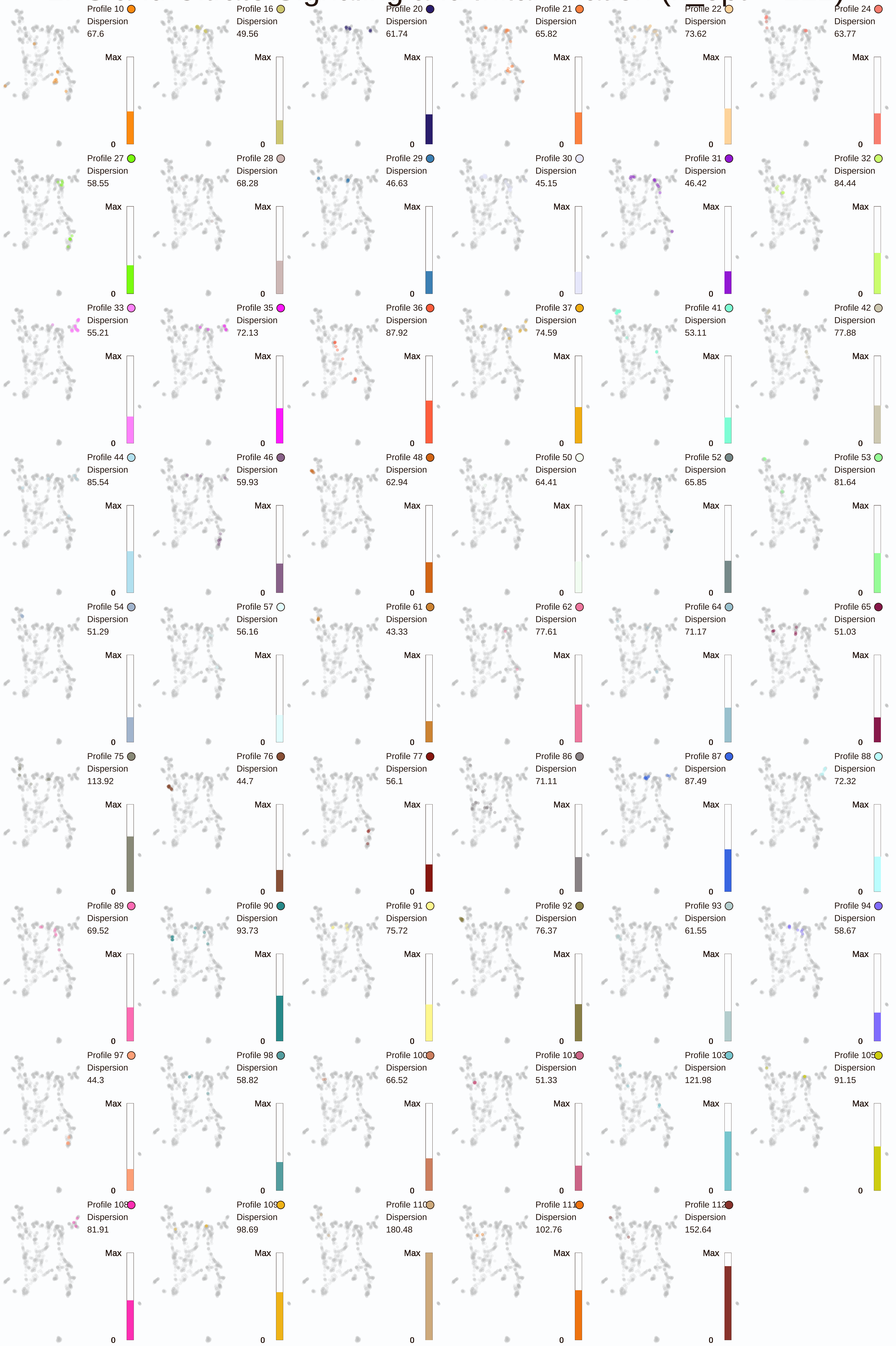

Lysophosphatidic Acid LPA2 Signalling (k_opt = 31)

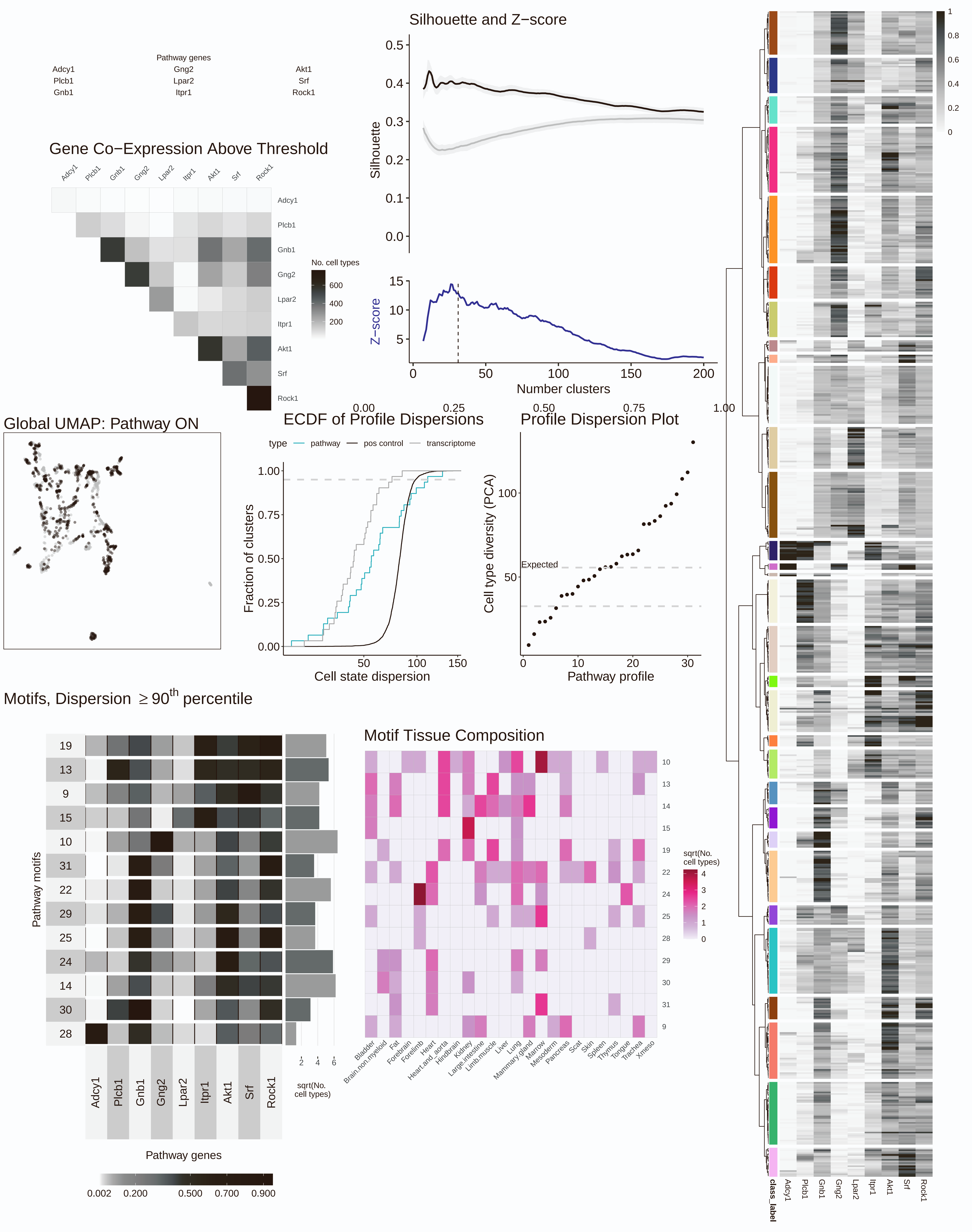

Lysophosphatidic Acid LPA2 Signalling (k_opt = 31)

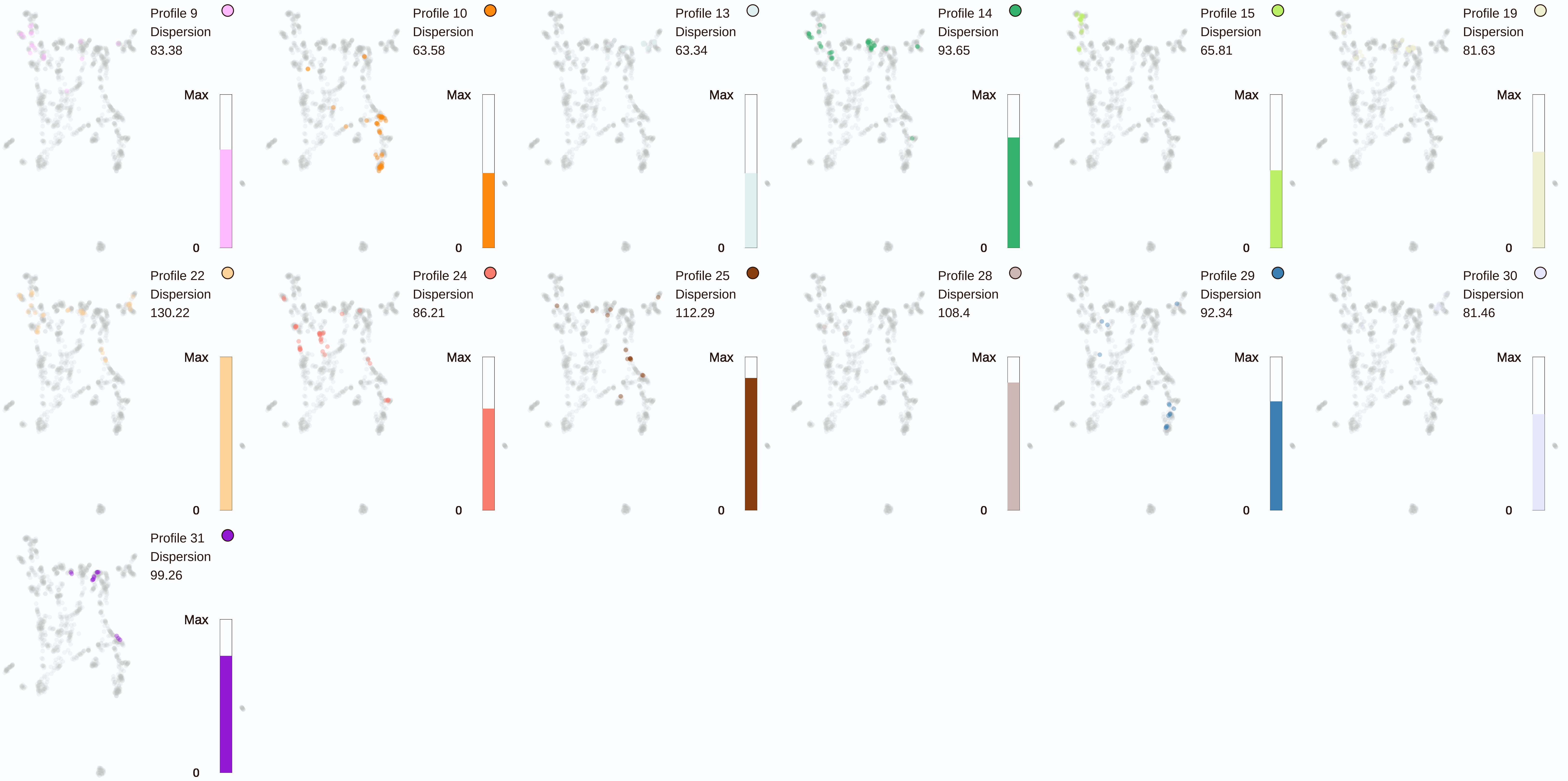

Mitochondrial Electron Transport Chain (k_opt = 43)

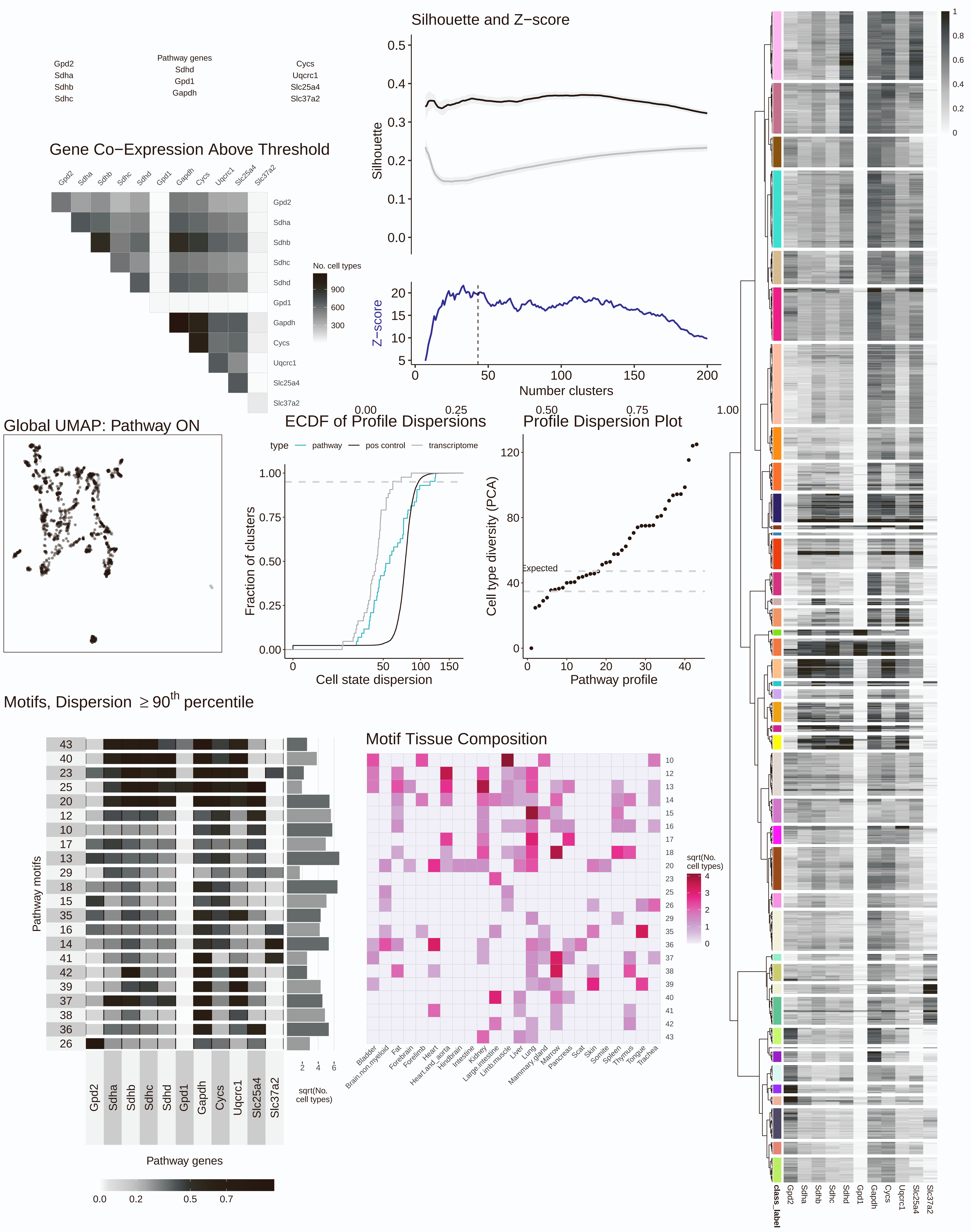

Mitochondrial Electron Transport Chain (k_opt = 43)

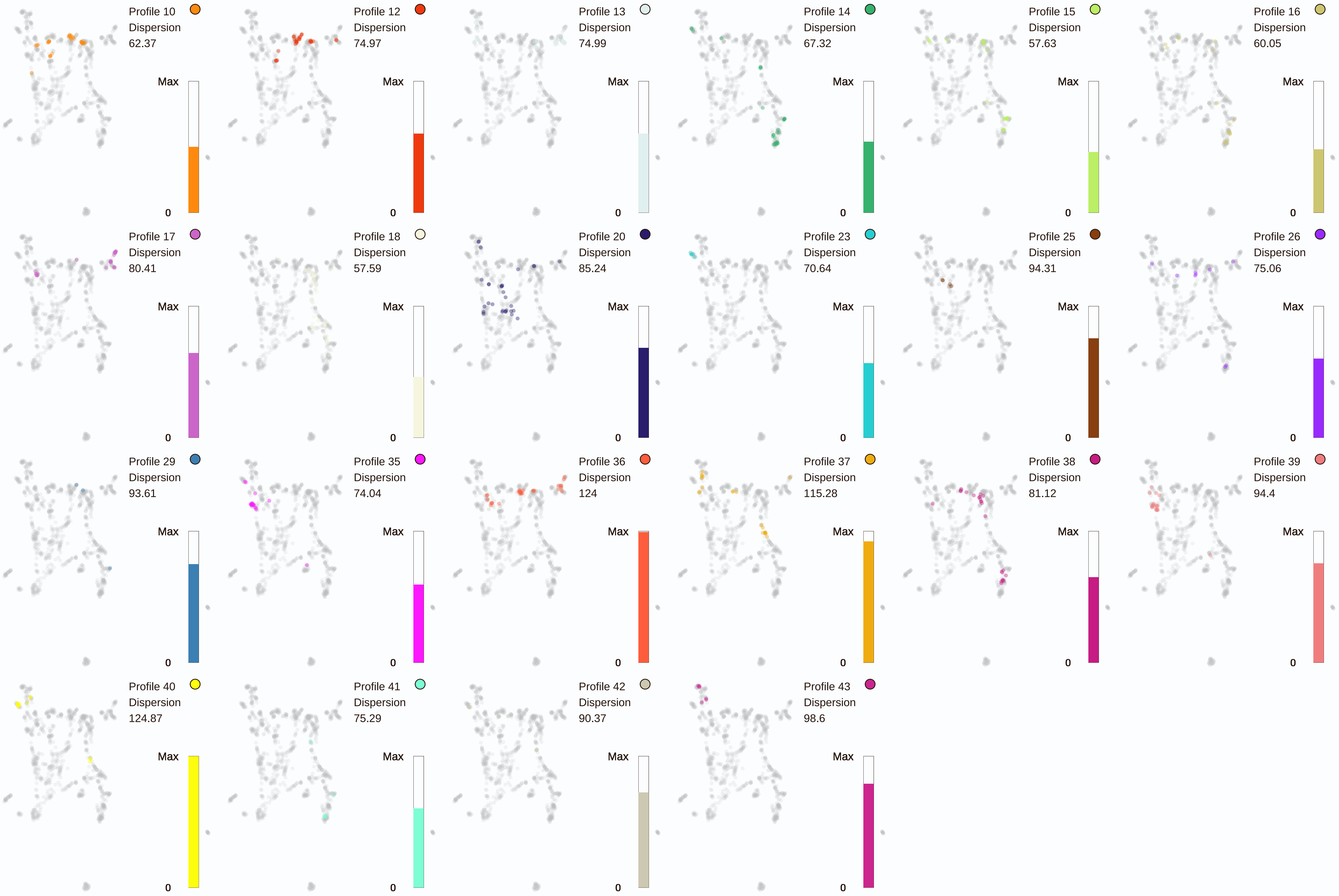

NF−kB Signaling Pathway (k_opt = 113)

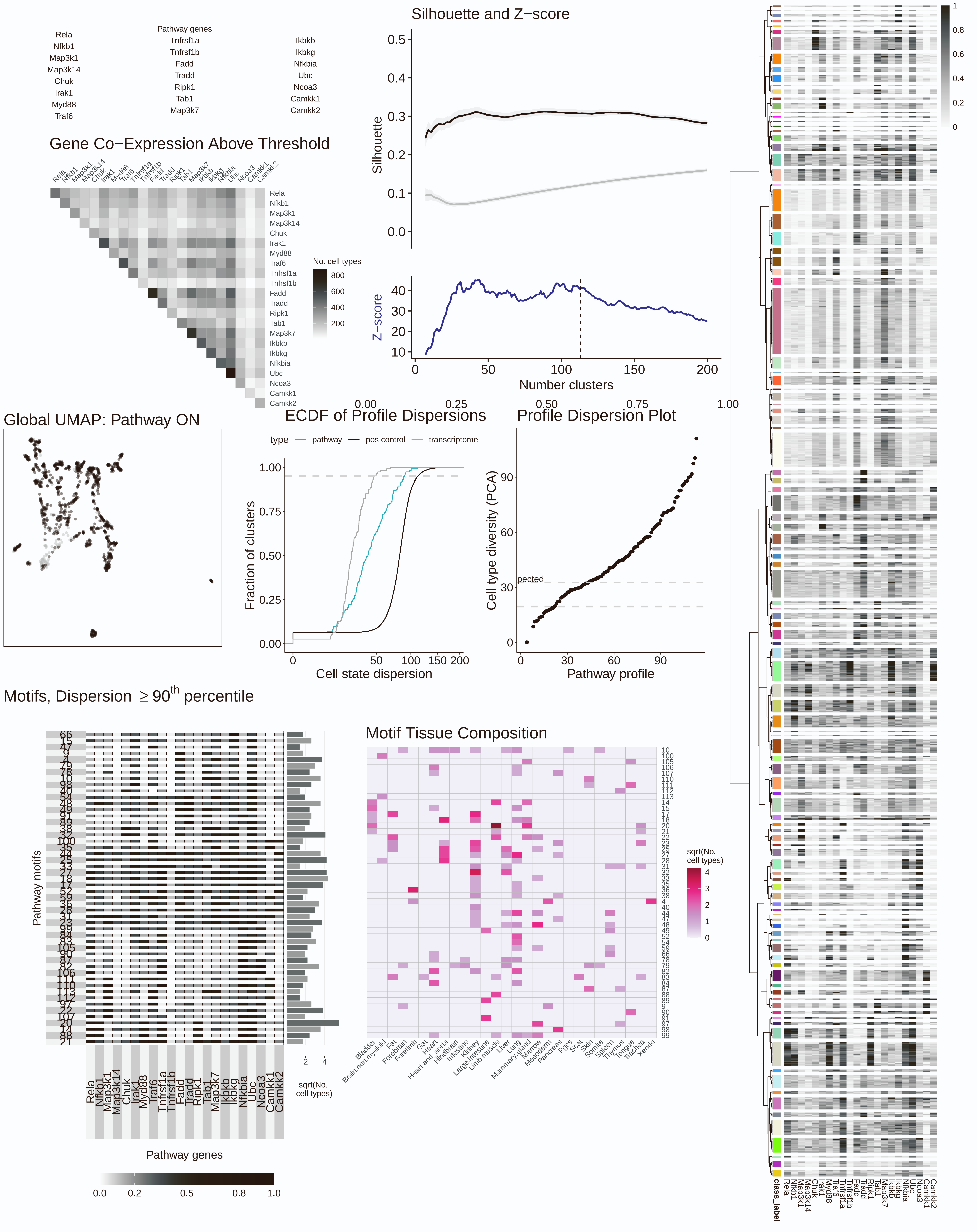

NF−kB Signaling Pathway (k_opt = 113)

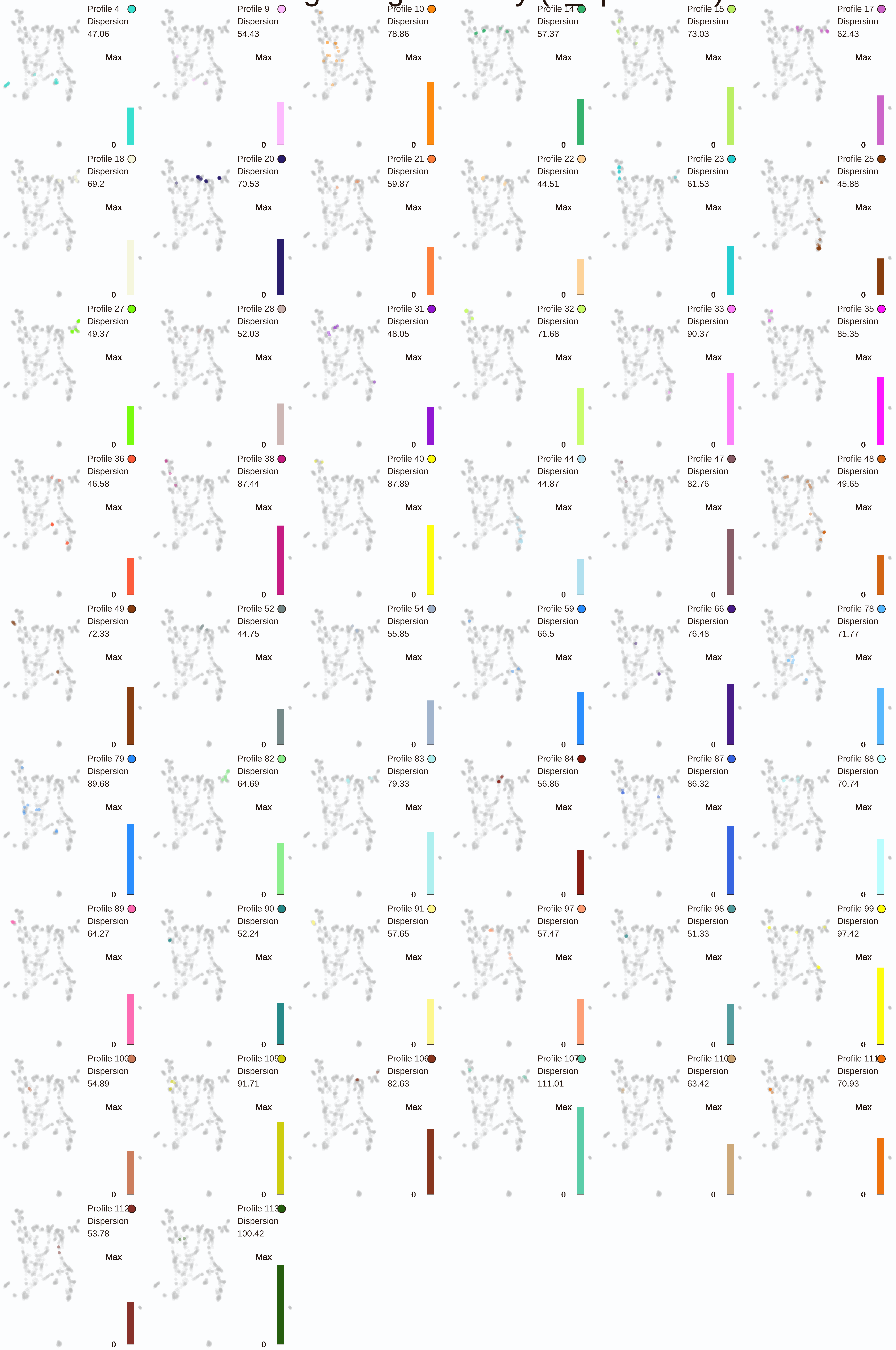

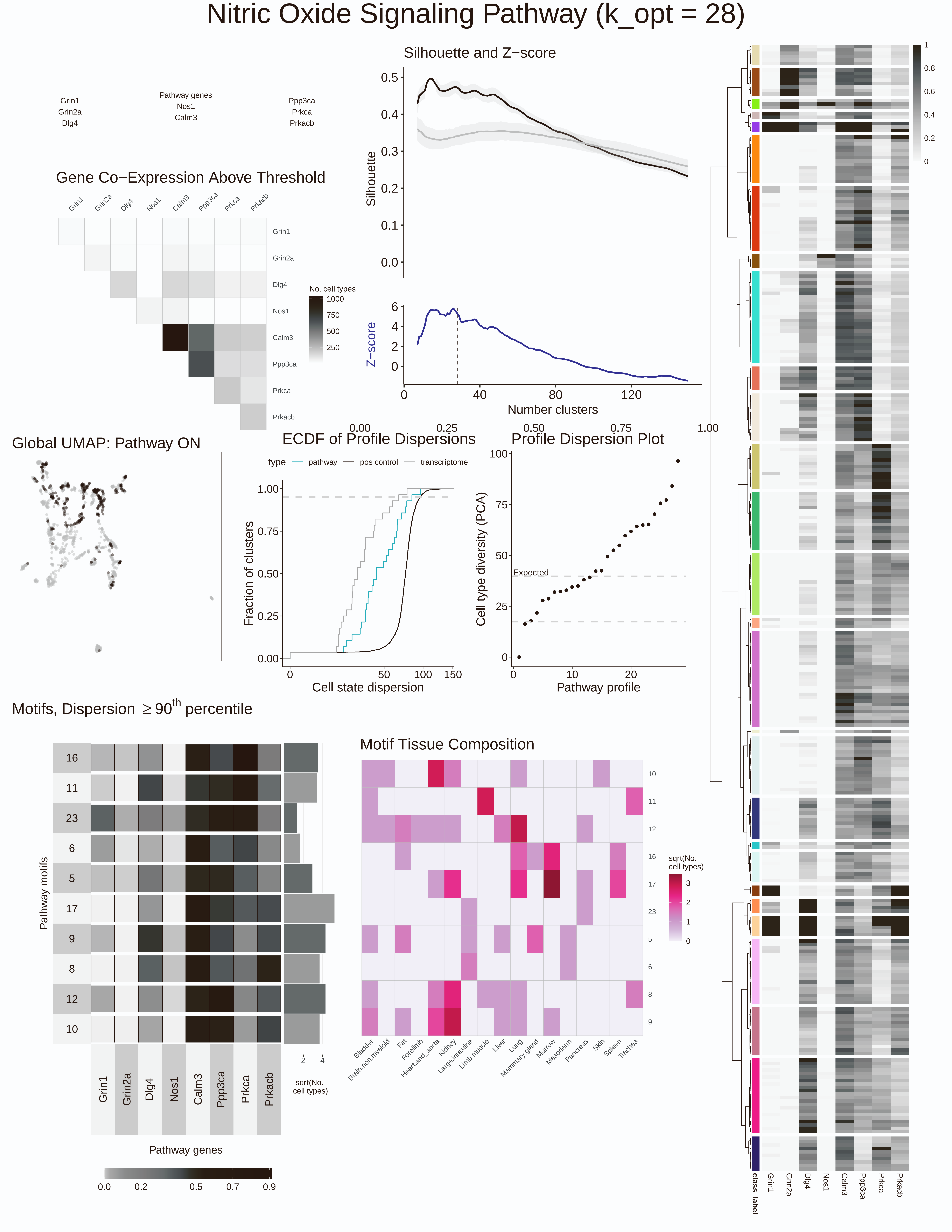
Nitric Oxide Signaling Pathway (k_opt = 28)
Grin1
Grin2a
Dlg4
Pathway genes
Nos1
Calm3
Ppp3ca
Prkca
Prkacb
Gene Co−Expression Above Threshold
No. cell types
1000
750
500
250
Silhouette and Z−score
Silhouette
Z−score
Number clusters
Global UMAP: Pathway ON
ECDF of Profile Dispersions
type
pathway
pos control
transcriptome
Fraction of clusters
Cell state dispersion
Profile Dispersion Plot
Expected
Cell type diversity (PCA)
Pathway profile
Motifs, Dispersion ≥ 90th percentile
Pathway motifs
sqrt(No. cell types)
Pathway genes
Motif Tissue Composition
Bladder
Brain.non.myeloid
Fat
Forelimb
Heart.and_aorta
Kidney
Large.intestine
Limb.muscle
Liver
Lung
Mammary.gland
Marrow
Mesoderm
Pancreas
Skin
Spleen
Trachea
sqrt(No. cell types)
class_label

Nitric Oxide Signaling Pathway (k_opt = 28)

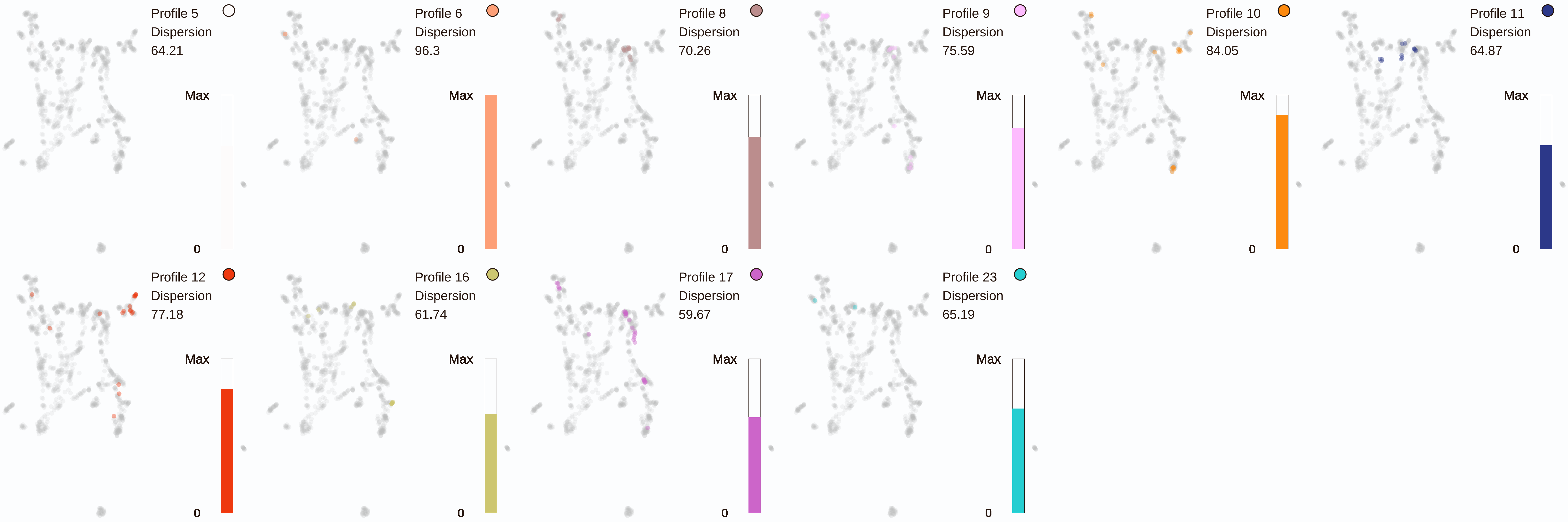

Notch receptors, Dll ligands and Fringe proteins (k_opt = 30)

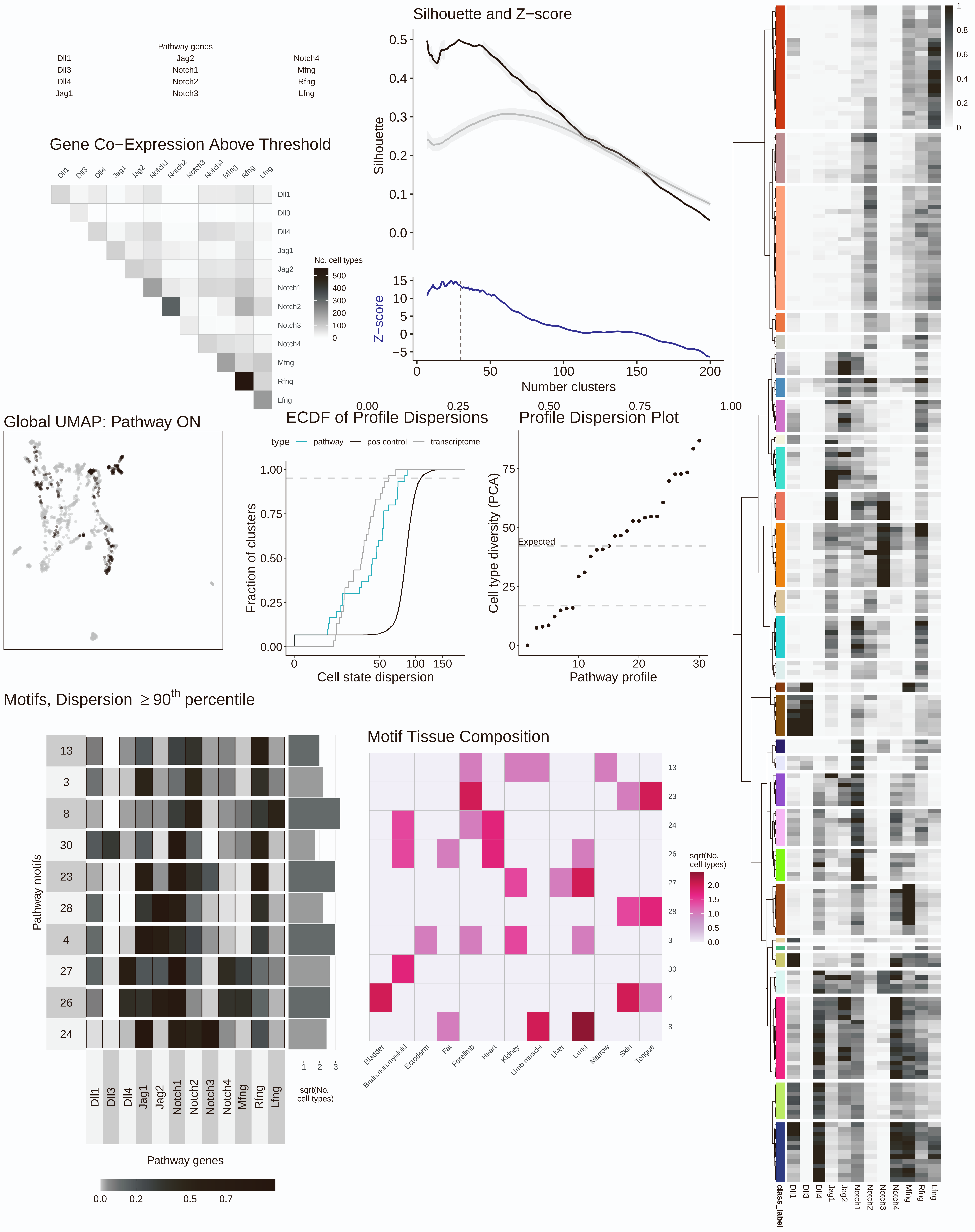

Notch receptors, Dll ligands and Fringe proteins (k_opt = 30)

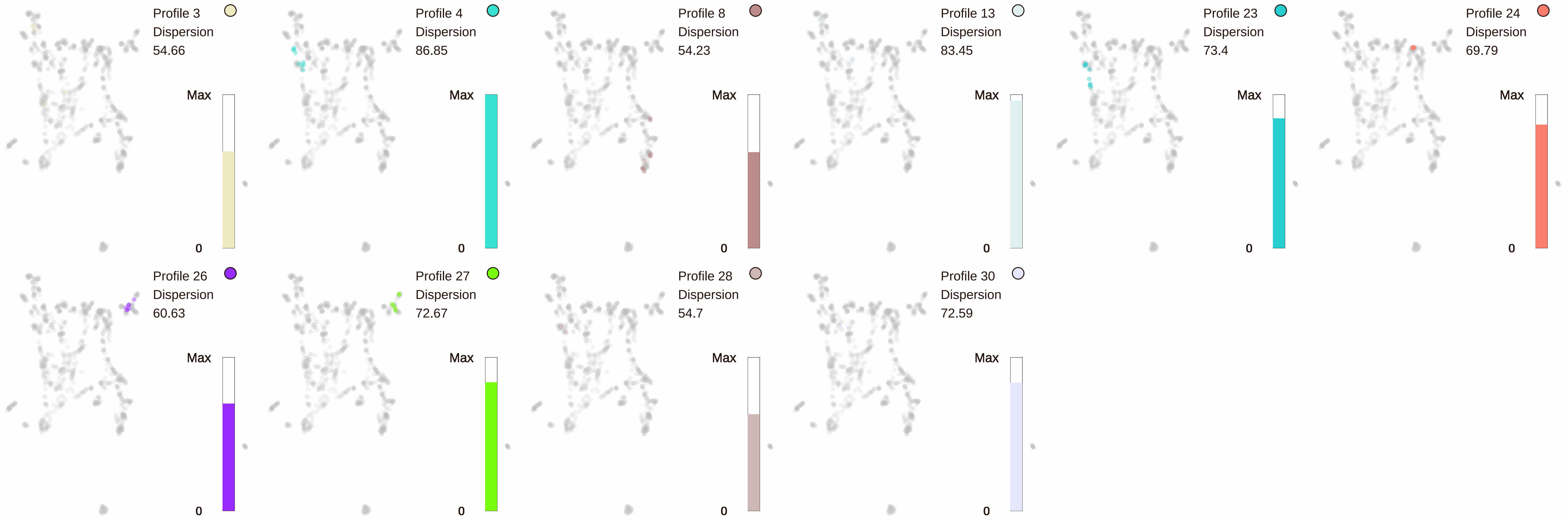

Ras Signaling Pathway (k_opt = 83)

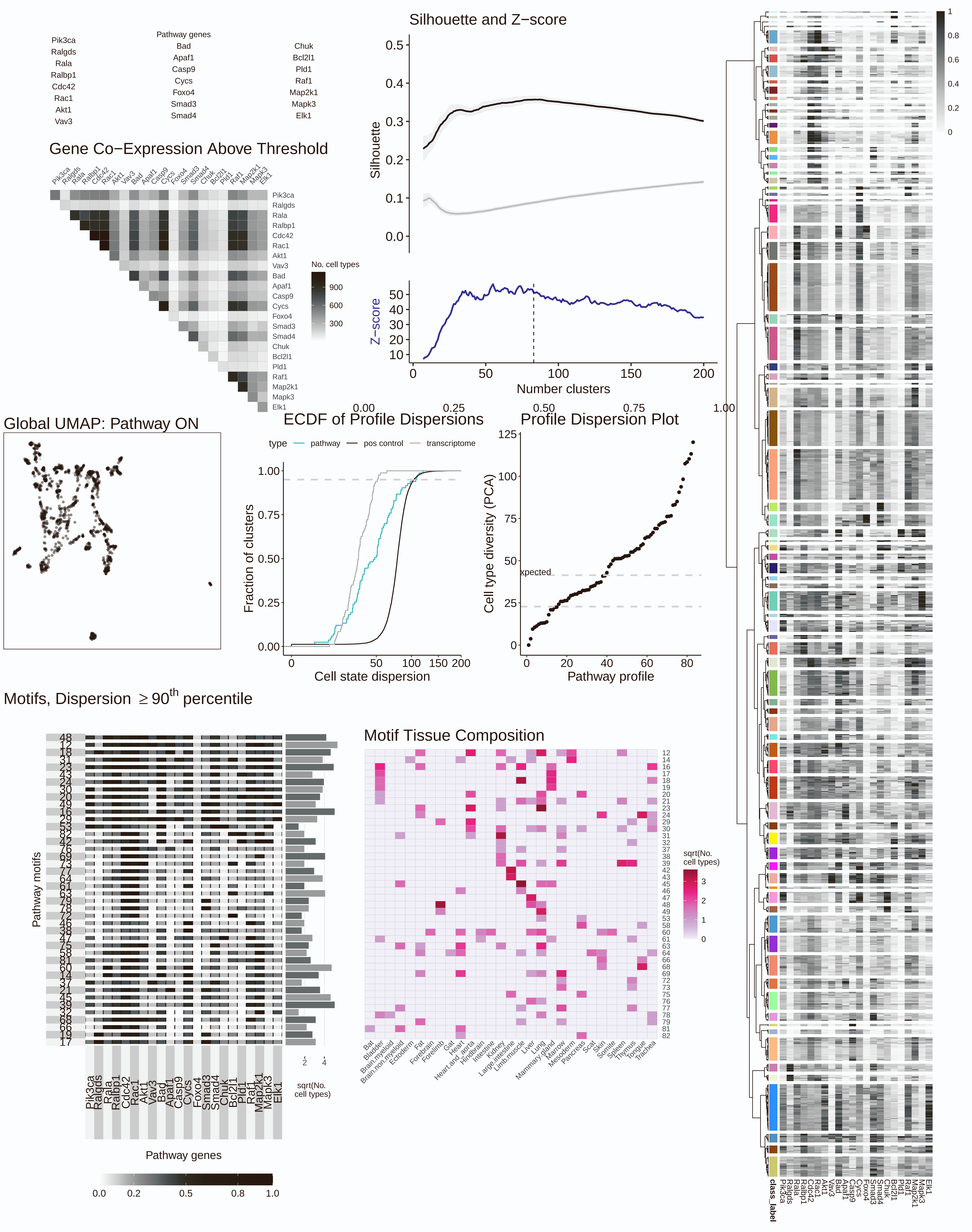

Ras Signaling Pathway (k_opt = 83)

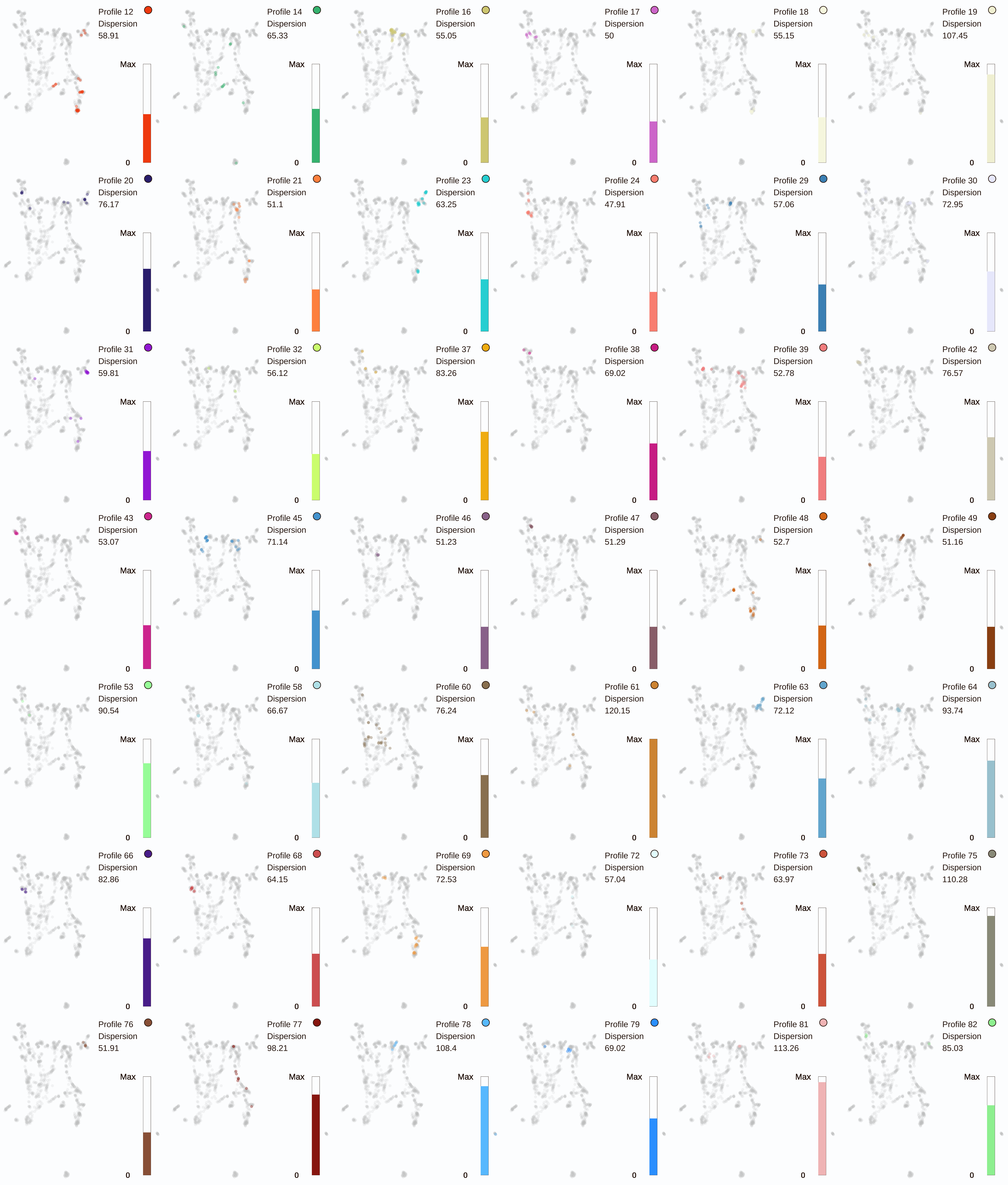

RNA−splicing by SR protein family (k_opt = 19)

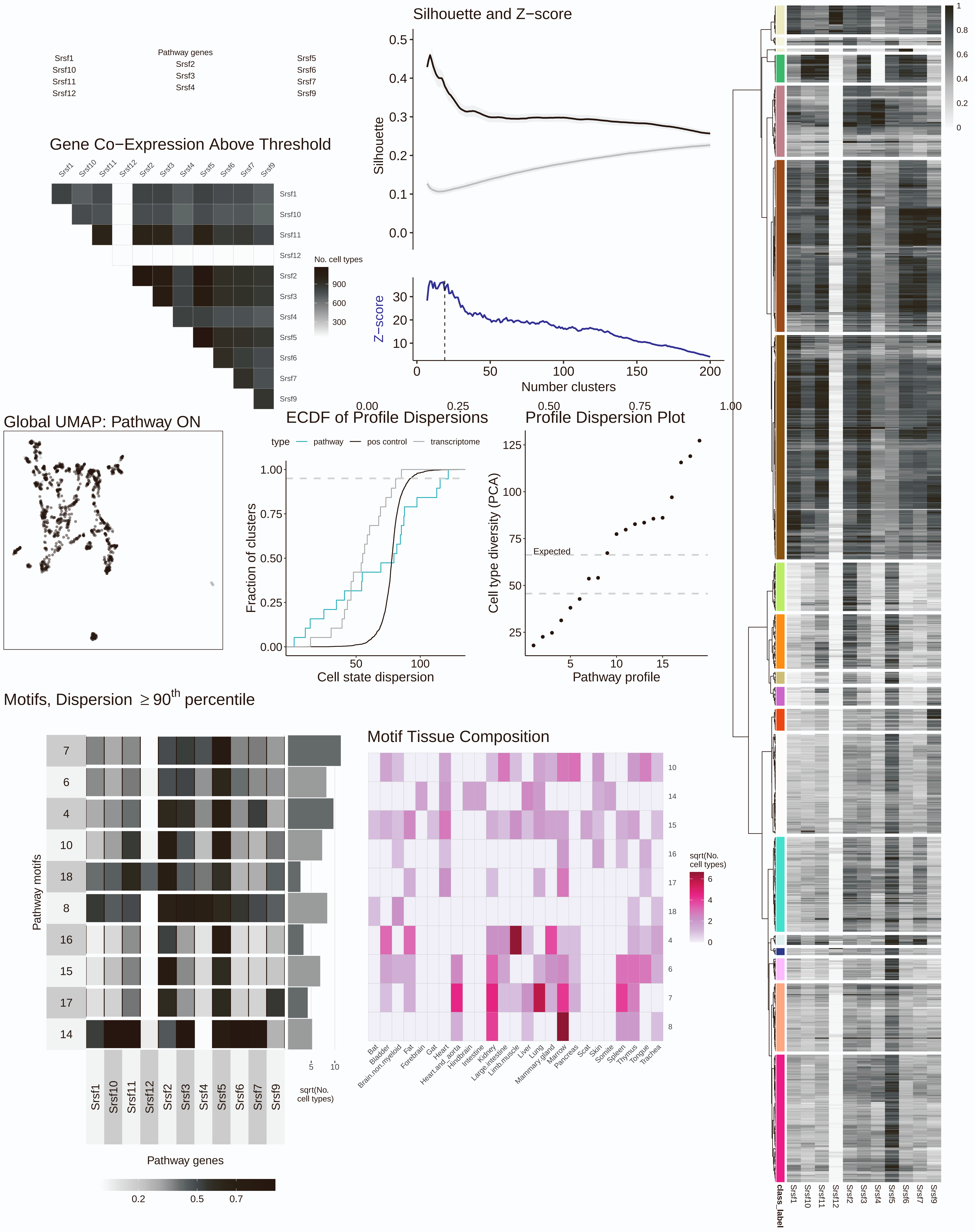

RNA−splicing by SR protein family (k_opt = 19)

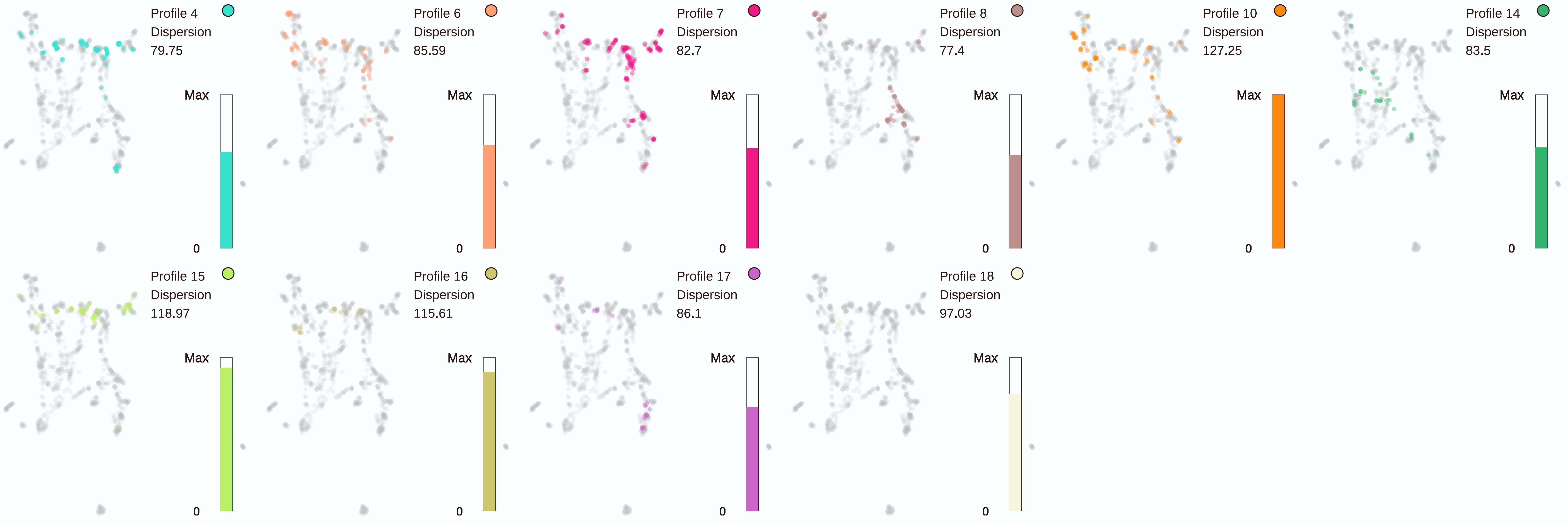

Tgf−beta family receptors (k_opt = 25)

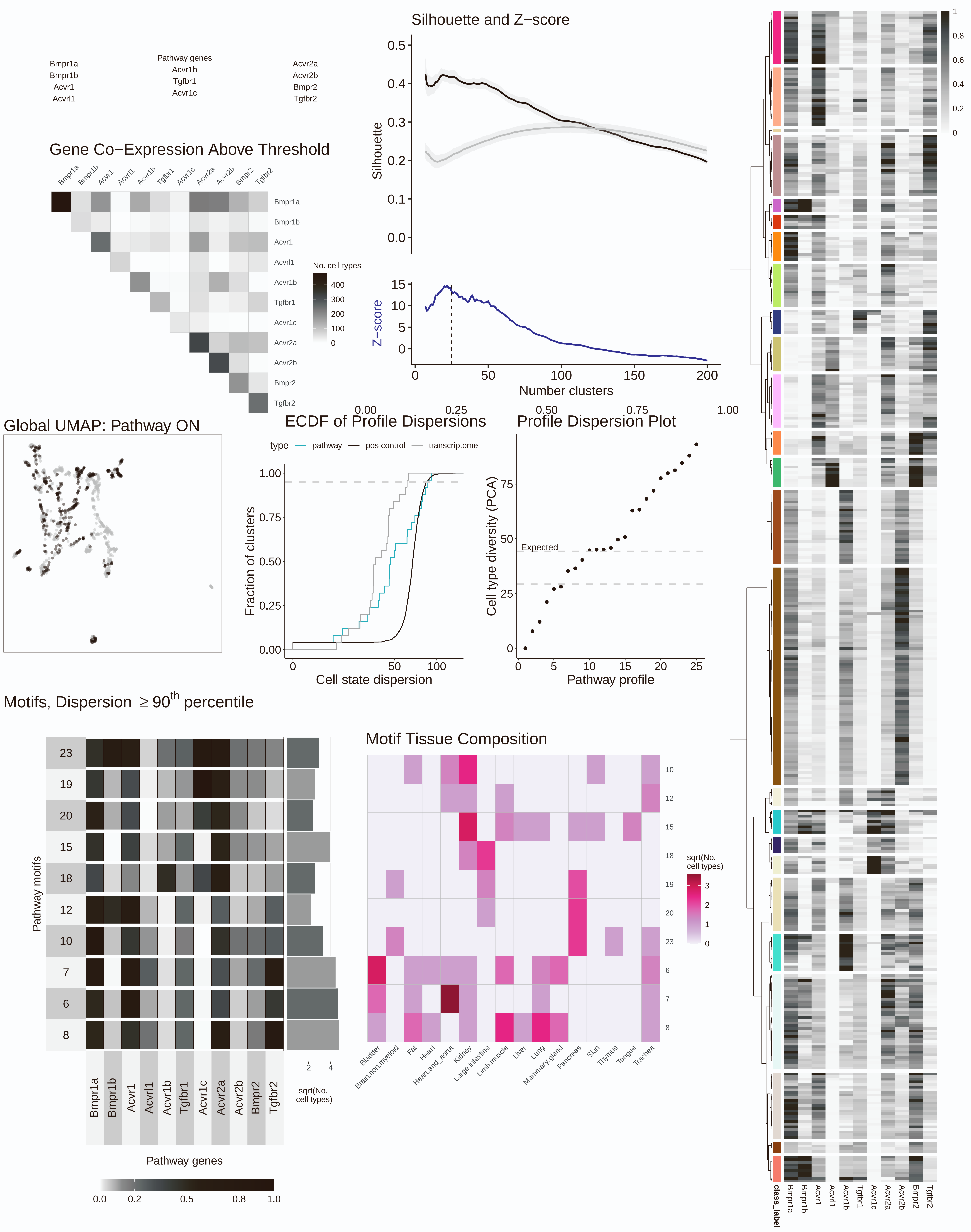

Tgf−beta family receptors (k_opt = 25)

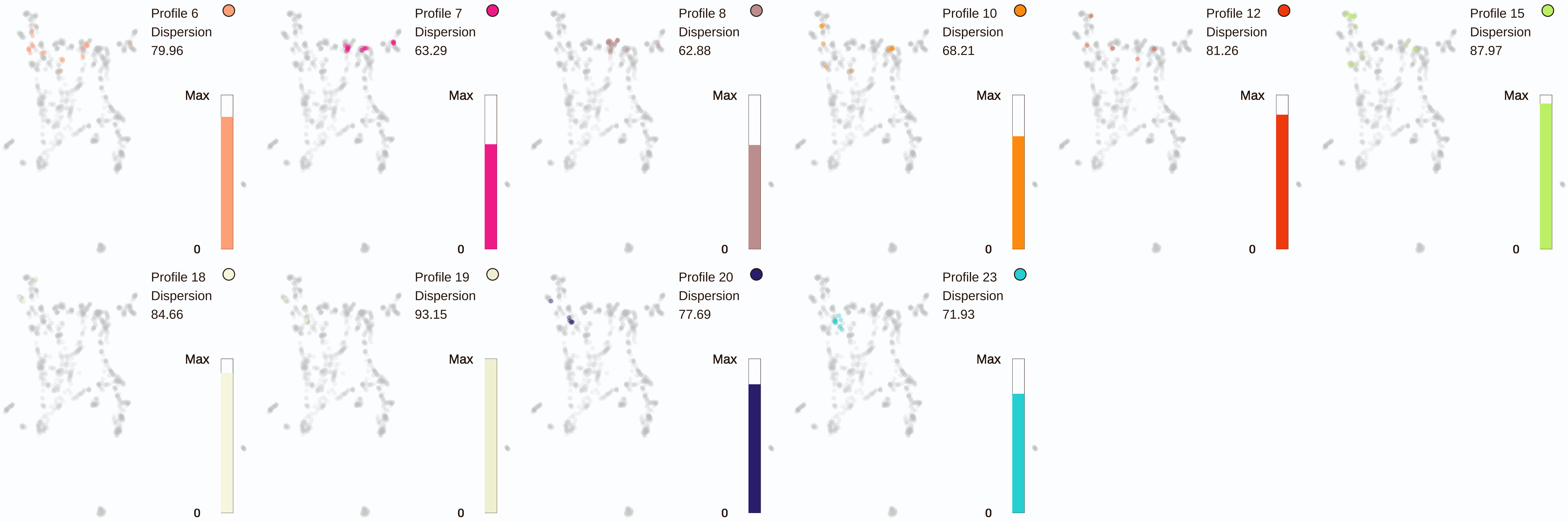

Supplement: Document S2. Article plus supplemental information [file mmc5.pdf]
